# Organic Dye-Sensitized Nitrene Generation: Intermolecular Aziridination of Unactivated Alkenes

**DOI:** 10.1021/acs.joc.3c02709

**Published:** 2024-02-15

**Authors:** Dennis Dam, Nathan R. Lagerweij, Katharina M. Janmaat, Ken Kok, Elisabeth Bouwman, Jeroen D. C. Codée

**Affiliations:** Leiden Institute of Chemistry, Universiteit Leiden, Leiden 2333 CC, The Netherlands

## Abstract

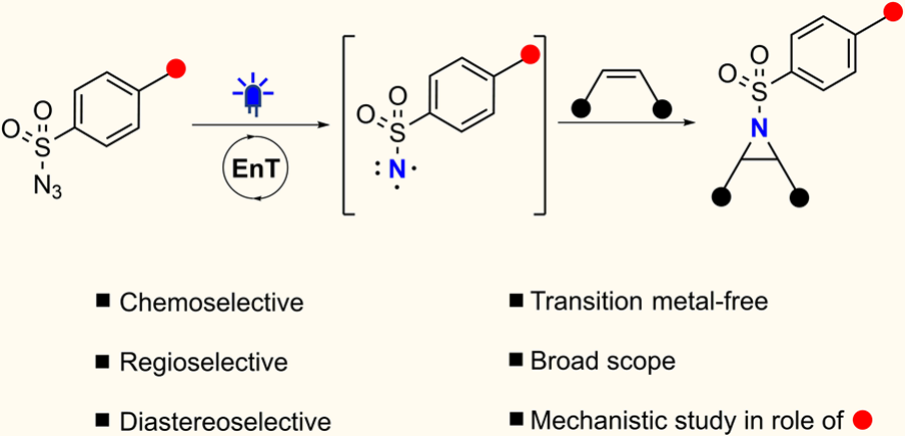

Aziridines are important structural motifs and intermediates, and several synthetic strategies for the direct aziridination of alkenes have been introduced. However, many of these strategies require an excess of activated alkene, suffer from competing side-reactions, have limited functional group tolerance, or involve precious transition metal-based catalysts. Herein, we demonstrate the direct aziridination of alkenes by combining sulfonyl azides as a triplet nitrene source with a catalytic amount of an organic dye functioning as photosensitizer. We show how the nature of the sulfonyl azide, in combination with the triplet-excited state energy of the photosensitizer, affects the aziridination yield and provide a mechanistic rationale to account for the observed dependence of the reaction yield on the nature of the organic dye and sulfonyl azide reagents. The optimized reaction conditions enable the aziridination of structurally diverse and complex alkenes, carrying various functional groups, with the alkene as the limiting reagent.

## Introduction

Aziridines, three-membered heterocycles containing one nitrogen atom, are important structural motifs in biologically active molecules either of natural or synthetic origin.^1^ They serve as useful synthetic intermediates en route to amine-containing products by virtue of their diverse ring-opening reactions.^2^ Especially *N*-sulfonylated aziridines find many applications in organic synthesis and as useful mononmers in polymer chemistry.^3^ Despite the plethora of applications of aziridine-based chemistry, the installment of aziridines on complex molecules remains challenging. In the past few decades, many efforts have been undertaken to develop synthetic methods to transform alkenes to the corresponding aziridines.

A useful approach for the direct transformation of an alkene to an aziridine is the direct insertion of a nitrene. Traditionally, these nitrenes were generated by α-elimination of *N*-sulfonylated urethanes,^4^ by UV-light irradiation of azides,^5^ or with the aid of transition-metal catalysts that activate a nitrene source, such as a haloamine, azide, or hypervalent iodine reagent.^6^ More recently, photoactivation of iminoiodinanes has been established for the generation of nitrenes and nitrene radical anions that produce aziridines.^7^ In addition, efforts have been undertaken to replace metal-based catalysts with organocatalysts with the aim of lowering costs, toxicity, and environmental impact.^8^ All of these methods are quite effective on activated substrates, such as styrene, but typically fail to give synthetically useful yields on more complex alkenes. Moreover, these strategies usually require an excess of alkene for productive yields, which is unattractive when the substrate alkene is not readily available but is the product of a multistep synthetic route. Furthermore, most of the reported methods have limited functional-group tolerance, or the reported conditions lead to competing side-reactions, such as allylic C–H amination.

**Scheme 1:**
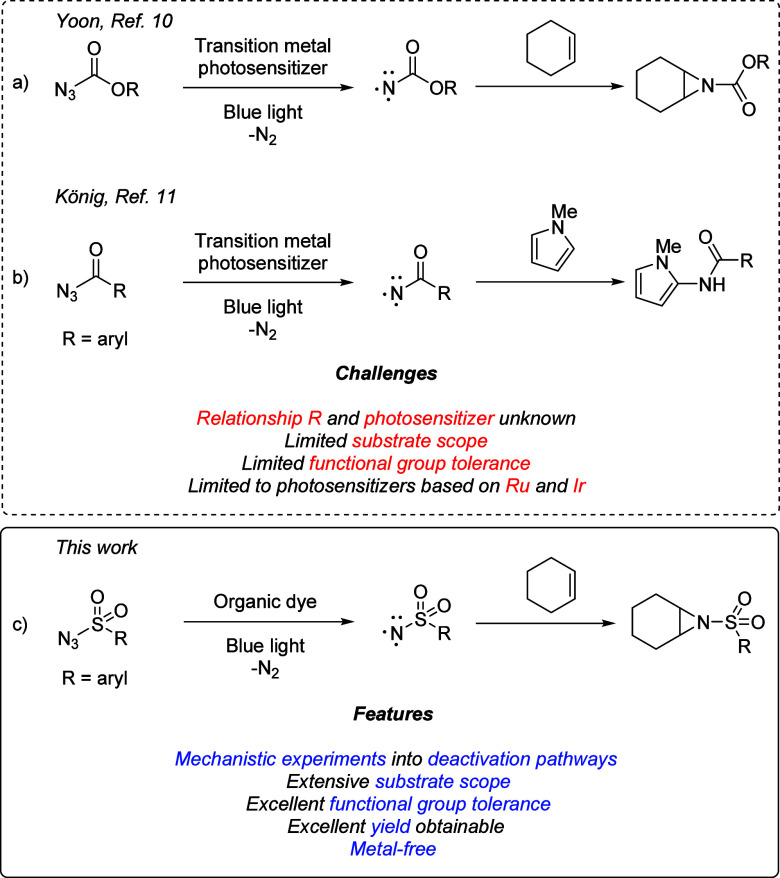
Photosensitization of Azides for Generation of Triplet Nitrenes

A promising alternative strategy was reported by Scholz et al. who showed that triplet sensitization^9^ of azidoformates leads to the formation of triplet nitrenes and subsequent alkene aziridination using the alkene as the limiting reagent (Scheme 1a).^10^ In addition, Brachet et al. have pioneered triplet sensitization of benzoyl azide for C(sp^2^)-H amidation (Scheme 1b),^11^ followed by a formal cycloaddition on alkenes to afford oxazolines.^12^ Despite these encouraging results, azide photosensitization remains underexplored and limited toward transition metal-based photosensitizers. The reactions have been found to be somewhat capricious to the nature of the azide substituent, and the mechanistic intricacies are not well understood, which has sparked endeavors toward the use of alternative nitrene precursors.^13^ To better understand the energy-transfer from photosensitizers to sulfonyl azides, the azide triplet-excited state energies (*E*_T_) have been computationally studied. However, significantly varying values have been determined, as exemplified by the *E*_T_ values reported for *para*-toluenesulfonylazide, varying between ∼49,^14^ ∼ 79,^15^ and ∼91 kcal mol^–1^.^16^ Additionally, endergonic energy transfers have been reported, further complicating mechanistic understanding.^17^

Herein, we describe our systematic studies to the aziridination of alkenes utilizing benzenesulfonyl azides as nitrene precursors. We demonstrate that by tuning the electronic effect of the substituent of the benzenesulfonyl azides, they can be ‘matched’ to the triplet excited-state energy of the photosensitizers. This prevents catalyst deactivation pathways, which otherwise lead to diminished yields. This has allowed us to effectively utilize organic dyes in a triplet-sensitized nitrene-transfer reaction, and we show that the generated triplet sulfonyl nitrenes can be used in the chemo-, regio-, and diastereoselective alkene aziridination of structurally complex alkenes. Importantly, we provide conditions in which the alkenes can be used as limiting reagents, which is attractive in the late-stage functionalization of expensive alkene substrates.

## Results and Discussion

Our investigation started with the evaluation of the aziridination reaction of cyclohexene (**2**), using *para*-methoxybenzenesulfonyl azide (**3-OMe**) in the presence of catalytic amounts of different cyanoaryl photosensitizers (**1a**–**f**) having *E*_T_ values ranging from 56.0 to 65.5 kcal mol^–1^ (Table 1).^18^ These organic photosensitizers are attractive because of their ease of synthesis, their highly energetic triplet-excited states that can be tuned by changing the aryl substituents, and their ability to absorb visible light.^19^ Furthermore, cyanoarenes do not initiate hydrogen-atom transfer reactions of weak C–H bonds, in contrast to photosensitizers based on aromatic ketones or quinones.^20^ A 5-fold excess of azide with respect to the alkene was required for the optimal yield (see Supporting Information for the full optimization table).

**Table 1:**
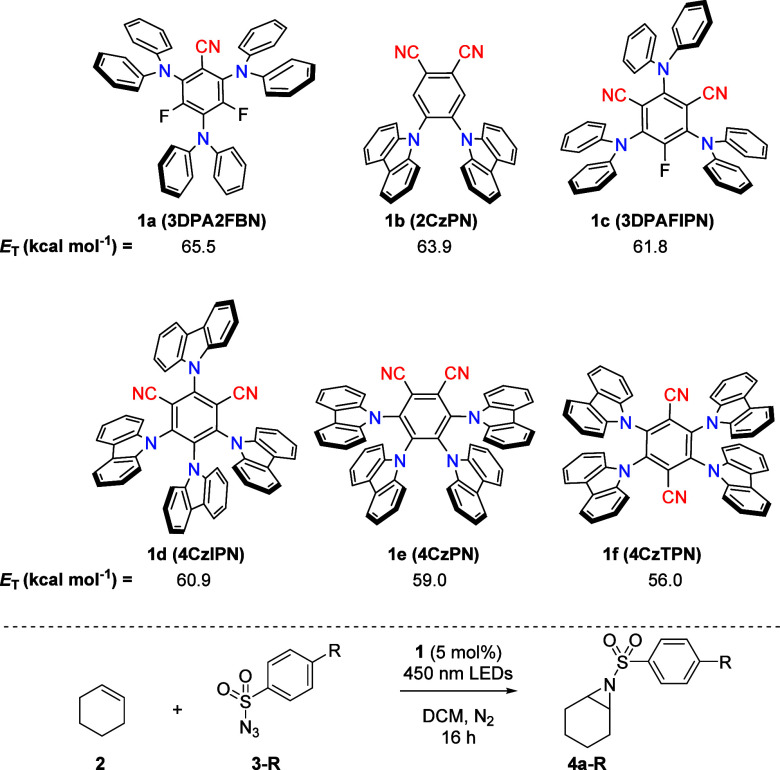
Initial Screening of Cyanoarene-Based Photosensitizers for Aziridination of Cyclohexene^a^

entry	photosensitizer	azide	yield (%)^b^
1	**1a**	**3-OMe**	15
2	**1b**	**3-OMe**	9
3	**1c**	**3-OMe**	48
4	**1d**	**3-OMe**	54
5	**1e**	**3-OMe**	60
6	**1f**	**3-OMe**	25
7^c^	**1e**	**3-OMe**	0
8	none	**3-OMe**	0
9	**1e**	**3-Me**	63
10	**1e**	**3-Cl**	53
11	**1e**	**3-CF**_**3**_	39

a All reactions were performed using 0.20 mmol **2**, 1.0 mmol **3-R** and under a dinitrogen atmosphere. For details, see Supporting Information.

b Yields were determined by ^1^H NMR spectroscopy using 1,3,5-trimethoxybenzene as internal standard.

c Performed in the dark in refluxing DCM. *E*_T_ values were obtained from the literature.^18^

While we expected high *E*_T_ values to be favorable for the reaction, as this should lead to increased rates of the energy-transfer event,^21^ we found that photosensitizers **1a** and **1b** (entries 1 and 2) gave low yields of aziridine and showed rapid discoloration of the reaction mixture from bright yellowish green to brown. To exclude that limited absorption of the blue light was an issue in these reactions, the reaction with **1a** (entry 1) was repeated in an NMR tube (see Supporting Information). This experiment showed that our irradiation wavelength was sufficient, and catalyst degradation likely limits the conversion of cyclohexene. Interestingly, **1c**, **1d**, and **1e** (entries 3, 4, and 5), which harbor lower *E*_T_ values than **1a** and **1b**, were more effective in catalyzing the formation of aziridine **4a-OMe**. This observation is in line with the reports from Scholz et al.^10^ and Brachet et al.^11^ who showed similar unexpected outcomes for azidoformates and benzoyl azides. Photosensitizer **1f** (entry 6) on the other hand provides the aziridine in lower yields than **1c**, **1d**, and **1e**. Control experiments in the absence of light or photosensitizer (entries 7 and 8) indicated the necessity for the excited state of the sensitizers to drive the reaction.

We elected **1e** for further studies in combination with different sulfonyl azides, carrying *para*-substituents with increasingly strong electron-withdrawing capacity (entries 9, 10, and 11). Interestingly, increasing the electron-withdrawing power of the azides significantly decreased the yield of aziridine. The optimal photosensitizer-azide pair for the highest yield was determined to be **1e** in combination with **3-Me**.

With the optimal photosensitizer-nitrene precursor pair defined, we explored the substrate scope of the aziridination reaction (Table 2). We started out by examining different simple aliphatic alkenes and styrenes to find that aziridines **4a**–**m** are readily obtained. The stereoablation found when *cis*- and *trans*-4-octene were used as substrates, yielding the same diastereomeric mixture of aziridines **4c**, is consistent with stepwise triplet nitrene insertion. The substituted styrenes that were evaluated all smoothly afforded the target aziridines (**4e**–**m**). Notably, even the strongly electron-withdrawing trifluoromethyl group did not have an adverse effect on the yield of the reaction.

**Table 2:**
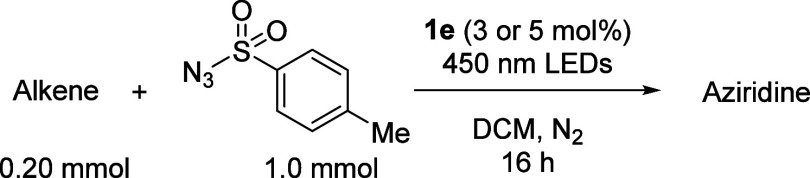

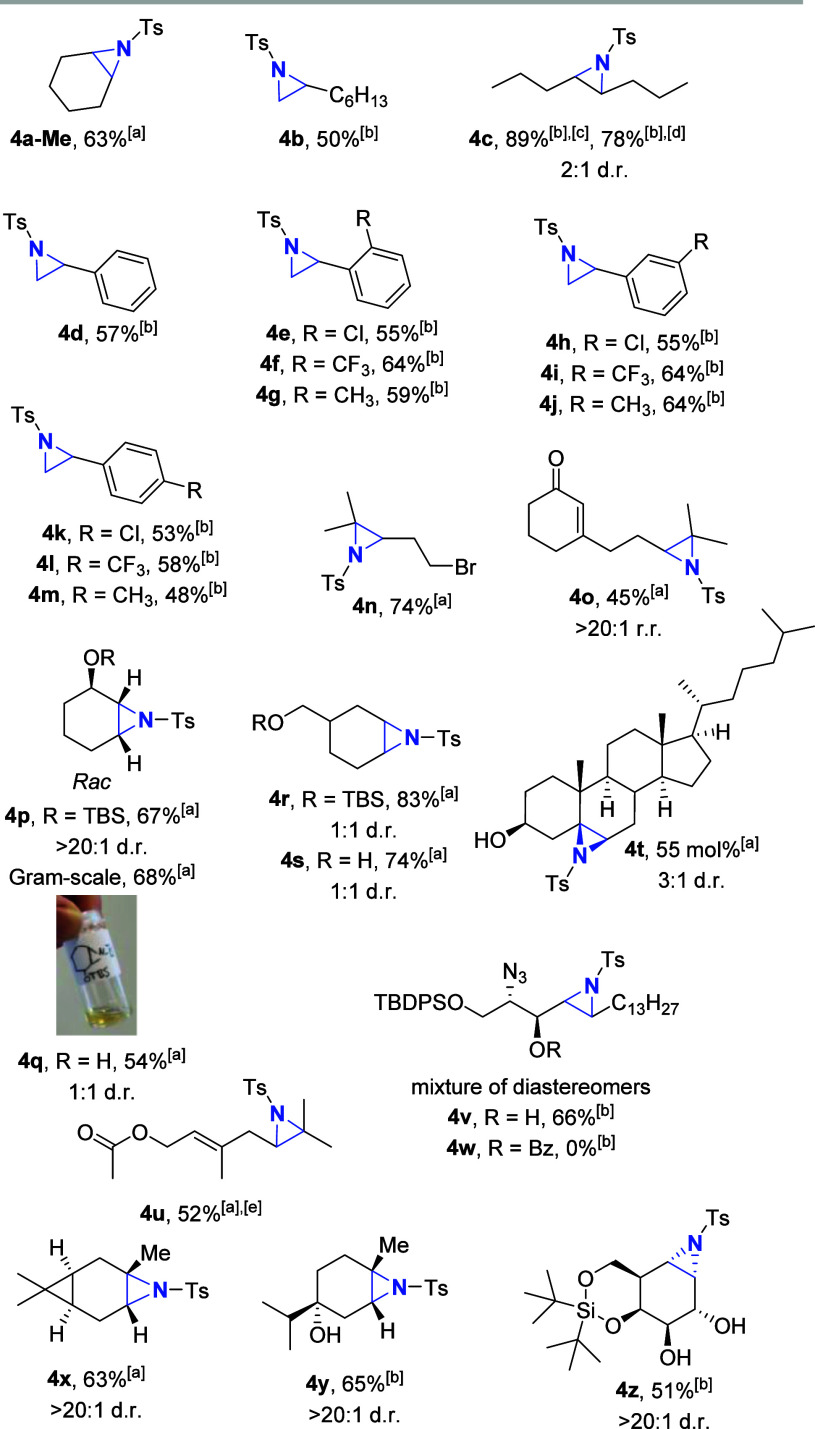
Scope of the Photocatalytic Aziridination Reaction^1^

1 ^a^ 3 mol% **1e** used. ^b^ 5 mol% **1e** used. ^c^ From cis-4-octene. ^d^ From trans-4-octene. ^e^ Some diaziridinated product observed in the ^1^H NMR spectrum of the crude product.

Encouraged by the successful aziridination of simple alkenes, we evaluated more complex substrates. The trisubstituted double bond in 1-bromo-4-methyl-3-pentene was readily transformed to the corresponding aziridine **4n**, and the potential for regioselective reactions was demonstrated by the selective aziridination of a substrate bearing an α,β-unsaturated ketone as shown by product **4o**. Highly diastereoselective reactions can be achieved by the use of sterically demanding groups, as shown by the excellent diastereocontrol reached in aziridine **4p**. We scaled up the synthesis of **4p** to gram-scale, to find if the reaction is readily scalable as it provided the same yield as on a 0.20 mmol scale (details in Supporting Information). No stereocontrol was exerted when the bulky silyl ether was positioned farther away from the double bond, delivering aziridine **4r** as a diastereomeric mixture. Removal of the bulky silyl protecting groups also led to loss of stereocontrol, and hydroxy aziridines **4q** and **4s** were isolated as a 1:1 diastereomeric mixture. Interestingly, the latter result illustrates compatibility of the reaction with hydroxyl functionalities, a feature that to the best of our knowledge has not yet been described for other triplet-sensitized nitrene-transfer reactions.

Next, several biomolecules and derivatives thereof were subjected to the methodology. Cholesterol afforded aziridine **4t** with moderate diastereoselectivity. The most electron-rich alkene of geranyl acetate reacted selectively to give aziridine **4u**. With a catalyst loading of 5 mol % **1e**, increased amounts of the diaziridine product were observed in the^1^H NMR spectrum of the crude product. A protected sphingosine smoothly afforded a mixture of aziridines **4v**. Noteworthy is the presence of the azide in the substrate, which was not detrimental to the reaction, likely because its activation requires photosensitizers having higher *E*_T_ values.^22^ When the alcohol was protected as a benzoate ester, the formation of aziridine **4w** could not be observed, and the starting material was recovered. This outcome highlights that, despite their high reactivity, triplet sulfonyl nitrenes prefer electron-rich alkenes in the aziridination reaction. The monoterpenes (+)-3-carene and (−) terpinen-4-ol (as a ∼ 2:1 mixture of enantiomers) provided excellent diastereocontrol, delivering aziridine products **4x** and **4y**.

Finally, we constructed a d-*galacto*-configured cyclophellitol analog that serves as a proxy for an intermediate toward a retaining galactosidase inhibitor.^23^ The aziridination of this type of alkenes generally involves multiple steps,^24^ and established methods for the direct alkene aziridination fail to give good yields and diastereocontrol.^25^ To achieve diastereoselective aziridination, we reasoned that the use of Kiso’s di-*tert*-butylsilylene protecting group could be a powerful strategy, as it is generally used to achieve α-selective galactosylation reactions in oligosaccharide synthesis.^26^ Gratifyingly, aziridine **4z** was obtained in good yield with excellent diastereocontrol.

**Figure 1:**
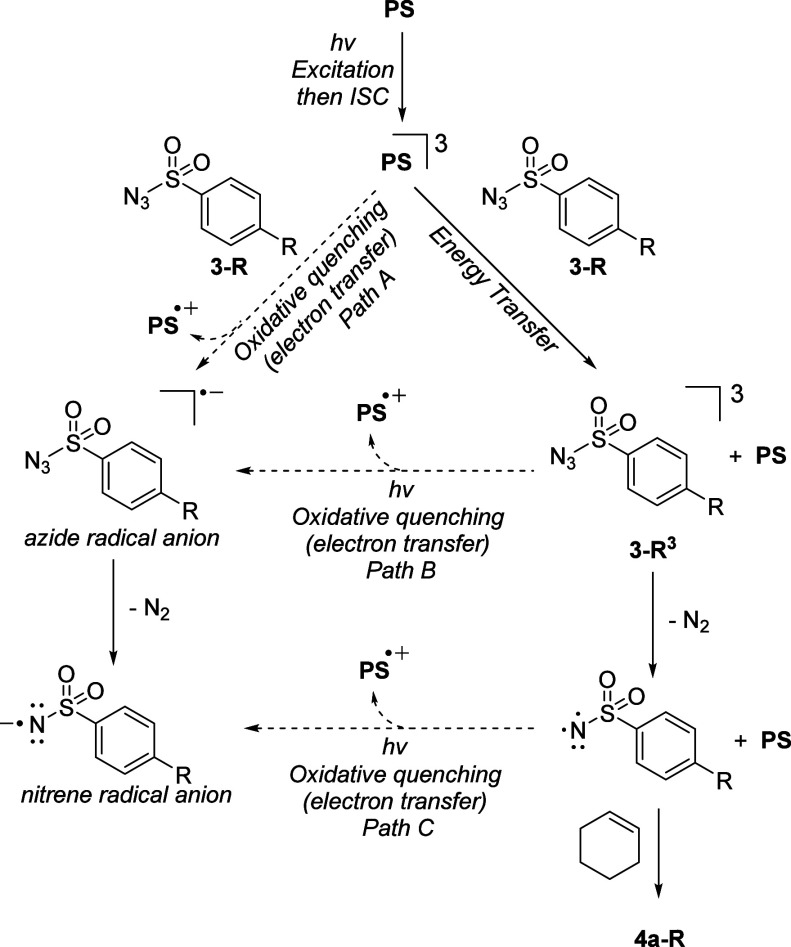
Mechanistic pathways considered. ISC = intersystem crossing, PS = photosensitizer.

**Figure 2:**
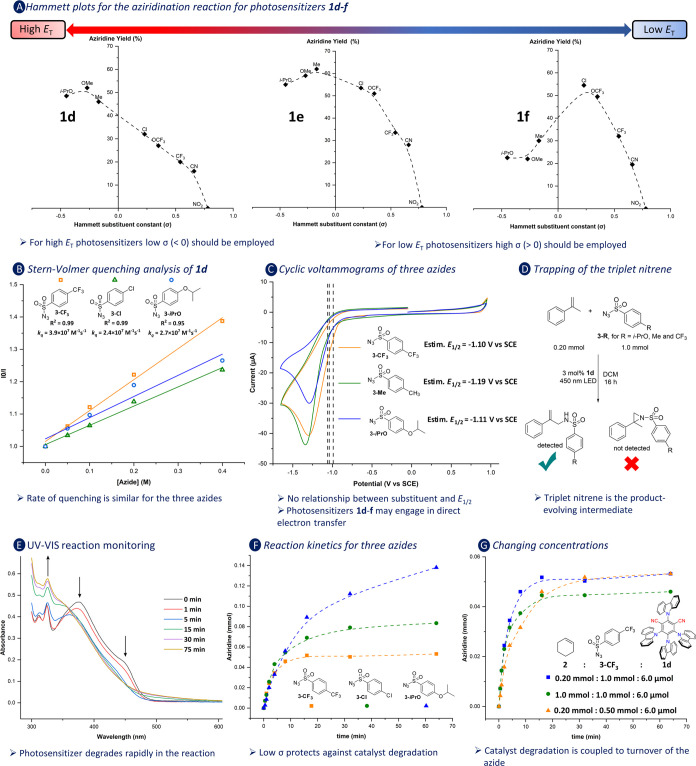
Results of selected mechanistic experiments. (A) Hammett plots for the aziridination reaction. Reaction conditions: 0.20 mmol cyclohexene, 1.0 mmol azide, and 3.0 mol % of the photosensitizer in 0.50 mL of DCM under 450 nm irradiation for 16 h. Yields were determined with ^1^H NMR spectroscopy using 1,3,5-trimethoxybenzene as internal standard. For each azide, the average yield of two experiments has been plotted. (B) Stern–Volmer quenching analysis for the quenching of **1d** by **3-CF**_**3**_, **3-**C**l**, and **3-***i***PrO** in DCM. (C) Cyclic voltammograms of **3-CF**_**3**_, **3-**M**e**, and **3-***i***PrO** (1 mM) in 0.1 M tetrabutylammonium hexafluorophosphate in MeCN plotted according to the IUPAC convention. Dashed lines indicate *E*_ox_*/V vs SCE values for **1d–1f**.^18^ recorded using a 0.07 cm^2^ glassy carbon working electrode, Pt wire auxiliary electrode, and Ag/AgCl reference electrode (sat. aq. KCl). Scanned first in anodic direction starting from −0.38 V vs SCE, with a scan rate of 50 mV/s. (D) selective triplet nitrene trapping using α-methylstyrene. (E) monitoring reaction over time with UV–vis. Reaction conditions: 0.20 mmol of cyclohexene, 1.0 mmol of **3-CF**_**3**_, and 3.0 mol % **1d** in DCM under 450 nm irradiation. (F) Kinetic profile of the reactions involving different azides. Reaction conditions: as in Table 1, but 3.0 mol % of **1d** was used in CD_2_Cl_2_ with 10 μL of CH_2_Br_2_ as internal standard. (G) Kinetic profile of reactions involving **3-CF**_**3**_ with differing [**2**] and [**3-CF**_**3**_]. Reaction conditions are as in Figure 2F.

Having established the applicability of the method for alkene aziridination, we set out to probe the mechanism of photosensitized nitrene transfer. Figure 1 provides an overview of plausible mechanistic pathways involving either triplet nitrene species or nitrene radical anions. As we observed an optimum in *E*_T_ of our photosensitizers for the activation of the different azides (Table 1), we reasoned that electron-transfer reactions might be able to explain this unexpected outcome. To understand the role of the substituent on the benzenesulfonylazide and its relation to the photophysical properties of the photosensitizer, the studies summarized in Figure 2 were undertaken.

We commenced by investigating the yield of the reactions as a function of the used photosensitizers (**1d**–**f**) in combination with eight sulfonyl azides differing in their *para*-substituents (−0.45 ≤ σ ≤ 0.78)^27^ as shown in Figure 2A, in which the yield of the reactions is plotted as a function of the Hammett parameter of the sulfonyl azide substituent. The three photosensitizers were chosen for their structural similarity, which should minimize differences in aziridine yield due to different chemical reactivity. Interestingly, for all photosensitizers the plots revealed an optimum in yield for a different azide, and the plots indicate that an optimum yield can be obtained by balancing two parameters: the electronic properties (σ) of the substituent on the azide and the *E*_T_ of the photosensitizer. Photosensitizers having a high *E*_T_ require azides with a low σ (<0), while photosensitizers with a low *E*_T_ can be combined with a sulfonyl azide, carrying a substituent having a high σ (>0) to obtain the best aziridine yield. Subsequent efforts focused on the origin of this unexpected finding. We elected **1d** for this purpose as it has the highest solubility in DCM, which facilitated our NMR studies reported in Figure 2.

To establish that the benzenesulfonyl azides did not affect the light absorbance by the photosensitizer in our reaction mixture, we evaluated the absorbance of **1d** and of three benzenesulfonyl azides (**3-***i***PrO**, **3-Cl**, **3-CF**_**3**_) (see Supporting Information). We found that **1d** is the sole absorbing species at 450 nm. Mixing the azides with **1d** did not lead to changes in the absorbance spectra, indicating that there is no ground-state association of the photosensitizer and azide. The absence of ground-state interactions was further corroborated by evaluating the Stern–Volmer plots of the luminescence quenching of **1d** (Figure 2B) by the **3-CF**_**3**_, **3-Cl**, and **3-***i***PrO** azides, which show that the three azides exhibit dynamic luminescence quenching of **1d**. Despite the difference in electronic properties of the azides, the quenching rate constants (*k*_q_) are in the same order of magnitude for the three azides. This indicates that the energy-transfer rates are similar for the three sulfonyl azides and that the optimum observed in the Hammett plot of **1d** does not originate from different kinetics in the energy-transfer step for the different azides. Furthermore, the outcome of the Stern–Volmer plots suggests that the *E*_T_ values of the three electronically different azides are similar.

To probe the possibility of electron transfer in our system (path A in Figure 1), we recorded the cyclic voltammograms of three electronically different azides, **3-***i***PrO**, **3-Me**, and **3-CF**_**3**_ (Figure 2C). Oxidative quenching of the photosensitizer by the azide can generate sulfonyl-nitrene radical anions, which have recently been shown to engage in the aziridination of alkenes through a photoredox catalytic cycle.^7c^ In all cases, irreversible waves were obtained in the voltammograms, which indicates consumption of the azides after reduction, which is consistent with loss of dinitrogen from an azide radical anion providing a nitrene radical anion (Figure 1). Because of the irreversibility of the reduction event, the *E*_1/2_ values were estimated at half the maximum current of the wave as described by Roth et al.^28^ Relatively small differences between the estimated *E*_1/2_ values of the azides were established, indicating that the sulfonyl azide substituents only have a minor effect in the electron transfer step. The *E*_1/2_ values also indicate that the photosensitizers **1d**–**f**, probed in Figure 2A, would be capable of electron transfer to the azides (*E*_ox_*/V vs SCE = −1.04 (**1d**), – 1.06 (**1e**), and −0.99 (**1f**)).^18^ Overall, based on these cyclic voltammetry experiments, we cannot exclude the occurrence of the nitrene radical anion pathway in the production of the aziridines.

Guo et al. recently established that α-methylstyrene can be used as a probe to distinguish between the intermediacy of triplet *para*-toluenesulfonyl nitrenes and *para*-toluenesulfonyl nitrene radical anions as product-forming species.^7c^ They established that α-methylstyrene reacts with a triplet nitrene to selectively give the C–H amination product, while the reaction with a nitrene radical anion exclusively leads to alkene aziridination. Therefore, we subjected α-methylstyrene to our reaction conditions using photosensitizer **1d** and the azides **3-***i***PrO**, **3-Me**, and **3-CF**_**3**_ (Figure 2D). In these experiments, we exclusively observed the C–H amination product, showing that triplet nitrenes are the product-evolving species in our reactions.

Having established that triplet nitrenes are the product-evolving transient intermediates in our aziridination reactions and that the energy transfer kinetics of the different sulfonyl azides do not significantly diverge (as deduced from the similar slopes in Figure 2B), we reasoned that the observed differences in aziridination yield as a function of the photosensitizer-nitrene precursor pair could be caused by consumption of the photosensitizer under the reaction conditions. Three possible oxidative quenching pathways (paths A–C, Figure 1) can lead to the consumption of the photosensitizer through the formation of the corresponding radical cation (**1d**^•+^ when **1d** is used) and consequently lower reaction yields.

To study degradation of the photosensitizer, we monitored the aziridination reaction of cyclohexene with **1d**/**3-CF**_**3**_ by UV–vis spectroscopy, showing that the absorption bands of **1d** rapidly disappeared (<15 min, Figure 2E). By recording aziridine formation over time by ^1^H NMR spectroscopy, we found that product formation stopped after 15 min (Figure 2F), in line with the degradation time of the photosensitizer, indicating that aziridine formation is limited by degradation of **1d**. Moreover, the ^1^H NMR spectra clearly showed the disappearance of the photosensitizer (see Supporting Information).

When **3-Cl** and **3-***i***PrO** were combined with **1d** in analogous NMR experiments (Figure 2F), we established similar initial rates for aziridine formation, which is consistent with the similar rates of energy-transfer of **1d** to the different azides (Figure 2B). However, Figure 2F shows that the more electron-donating substituents on the benzenesulfonyl azide (**3-***i***PrO** > **3-Cl** > **3-CF**_**3**_) lead to prolonged reaction times, during which the aziridine product is formed, and consequently a higher yield is obtained. These results suggest that the rate of catalyst degradation is related to the electronic properties of the benzenesulfonyl azide substituent, with the substituents having a higher σ, leading to faster degradation of **1d**. This substantiates the notion that an oxidative quenching pathway, in which an electron is transferred from the photosensitizer to the benzenesulfonyl azide or nitrene, abrogates the reaction.

Finally, we studied the effects of the relative concentration of the benzenesulfonyl azide and alkene on the yield of the reaction (Figure 2G). When increasing the concentration of cyclohexene **2** 5-fold compared to the standard reaction conditions, we observed no difference in the time point at which product evolution halts. On the other hand, when the concentration of the azide was halved, aziridine formation became observably slower, but the effective reaction time increased, indicating that the rate of sensitizer degradation decreased. Of note, the aziridine yield, and thus the turnover number (TON ≈ 1) of the photosensitizer, in all cases was similar. To rule out that the aziridine product is involved in sensitizer degradation, we investigated the consumption of the aziridine in the presence of the photosensitizer and blue light irradiation. In these experiments, we could not observe degradation of either component. Overall, these results show that sensitizer degradation coincides with turnover of the azide and can be rationalized by electron transfer from the photocatalyst to the benzenesulfonylazide or nitrene. As there is no significant anodic shift in the *E*_1/2_ values for the benzenesulfonyl azides, as judged from the voltammograms (Figure 2C), nor an increased rate of quenching with increased electron-withdrawing power (as measured by the Stern–Volmer plots in Figure 2B), the difference in reaction yield obtained with the different azides, cannot be readily reconciled with electron transfer path A (Figure 1). It is therefore more likely that oxidative quenching occurs after the energy-transfer event, following pathways B and/or C. This allows for rationalization of the Hammett plots for azides bearing σ > 0: as the azides quench the excited state of the photosensitizer at similar rates, the rate of formation of triplet azide and thereby the rate of triplet nitrene formation is dependent on the *E*_T_ of the photosensitizer. The triplet nitrenes generated from the benzenesulfonyl azides having stronger electron withdrawing groups (higher σ-values) are likely stronger oxidants than their electron-donating counterparts (lower σ-values) and, therefore, show enhanced oxidative quenching and degradation of the photosensitizer.

## Conclusions

We have successfully expanded the scope of photosensitizers in photosensitized nitrene-transfer reactions from transition metal-based sensitizers to organic dyes. We have shown that the produced triplet sulfonyl nitrenes are highly efficient and selective species for alkene aziridination. This has allowed us to perform late-stage installation of *N*-sulfonylated aziridines on complex (bio)molecules harboring various functional groups, including alcohols, azides, α,β-unsaturated ketones, and halides. Diastereoselective, regioselective, and chemoselective reactions can be achieved.

In addition to the synthetic utility demonstrated, we introduced the concept of ‘matching’ the electronic effect on the azide to the *E*_T_ of the photosensitizer. This concept establishes that in photosensitized sulfonyl nitrene transfer, the photosensitizer can engage in competing oxidative quenching processes toward transient species, leading to loss of photocatalytic activity. Efficient reactions can be conceived by utilizing the highly energetic *E*_T_ states of the photosensitizers and electron-donating groups on the sulfonyl azide. This finding will aid future research in photosensitized nitrene-transfer reactions and provides an explanation for the sensitive nature of previously reported reactions to the nature of the used nitrene precursors and the observations that high *E*_T_ photosensitizers do not as such lead to efficient reactions.

## Data Availability

The data underlying this study are available in the published article and its Supporting Information.

## Supporting Information Available

Catalytic and synthetic procedures, procedures for mechanistic studies and characterization of compounds (PDF)
