# Supplementary material for: Organic Dye-Sensitized Nitrene Generation: Intermolecular Aziridination of Unactivated Alkenes
Source: J Org Chem. 2024 Feb 15;89(5):3251–8. doi: 10.1021/acs.joc.3c02709 (PMC10913034; doi:10.1021/acs.joc.3c02709)

Supporting information for

Organic Dye-Sensitized Nitrene Generation: Intermolecular Aziridination of Unactivated alkenes

Dennis Dam, Nathan R. Lagerweij, Katharina M. Janmaat, Ken Kok, Elisabeth Bouwman*, and Jeroen D. C. Codée*

Table of Contents

1. General Experimental Details S2
2. Synthesis Of New Starting Materials S4
3. Reaction optimization S5
4. NMR reaction of 1a with 3-OMe on cyclohexene *(cf. Table 1)* S6
5. Substrate Scope *(cf. Table 2)* S7
6. Scale-up reaction *(cf. Table 2)* S19
7. Hammett Plots *(cf. Figure 2a)* S20
8. Stern-Volmer luminescence quenching studies *(cf. Figure 2b)* S21
9. Cyclic Voltammetry *(cf. Figure 2c)* S22
10. Triplet nitrene trapping *(cf. Figure 2d)* S24
11. UV-Vis experiments *(cf. Figure 2e)* S25
12. Reaction kinetics by ^{1}H NMR spectroscopy *(cf. Figures 2f and 2g)* S28
13. Limitations S29
14. References S30
15. NMR Spectra of new compounds and compounds isolated from catalytic reactions S32

1. General Experimental Details

All commercial chemicals were used directly without purification unless stated otherwise. Solvents were dried over flame-dried molecular sieves of the appropriate size or obtained from a Pure-Solv 400 solvent purification system. Air and water sensitive reactions were performed under a dry nitrogen atmosphere using standard Schlenk techniques. TLC analysis was performed using TLC Silica gel (Kieselgel 60 F_{254}, Merck) with UV detection at 254 nm and by spraying a solution of $(NH_4)_6Mo_7O_{24}\cdot H_2O$ (25 g/L) and $(NH_4)_4Ce(SO_4)_4\cdot H_2O$ (10 g/mL) in 10% sulfuric acid and subsequent charring at ~300 °C using a hot plate. Flash column chromatography was performed manually using silica gel 60Å (40-63 µm) from Screening Devices. All mixtures of solvents are reported as v/v solutions. High-resolution mass spectra were recorded on a Thermo Finnigan LTQ Orbitrap mass spectrometer equipped with an electrospray ion source in positive mode (source voltage 3.5 kV, sheath gas flow 10, capillary temperature 275 °C) with resolution R=60.000 at m/z=400 (mass range = 150-4000). ^{1}H NMR, ^{13}C{^{1}H}-APT NMR and ^{19}F-NMR spectra were recorded on a Bruker AV-500 (500/126/471 MHz), or a Bruker AV-400 (400/101/376 MHz) spectrometer. Chemical shifts are given in ppm relative to tetramethyl silane (TMS), or chloroform for ^{1}H NMR and ^{13}C{^{1}H}-APT NMR spectra. ^{19}F NMR spectra are referenced against $CFCl_3$ (external reference). 2D NMR experiments (HSQC, COSY, NOESY) were carried out to assign protons and carbons and the stereochemistry of the diastereoselective reactions. Photocatalytic reactions were irradiated using the EvoluChem PhotoRedOx box™ equipped with a 30 W blue LED (λ_{max} = 450 nm, HCK1012-01-002, EvoluChem™). The temperature was maintained between 27 °C and 29 °C by air cooling using the built-in fan. A custom-made holder was fitted to allow the use of small glass vials (Figure S1).

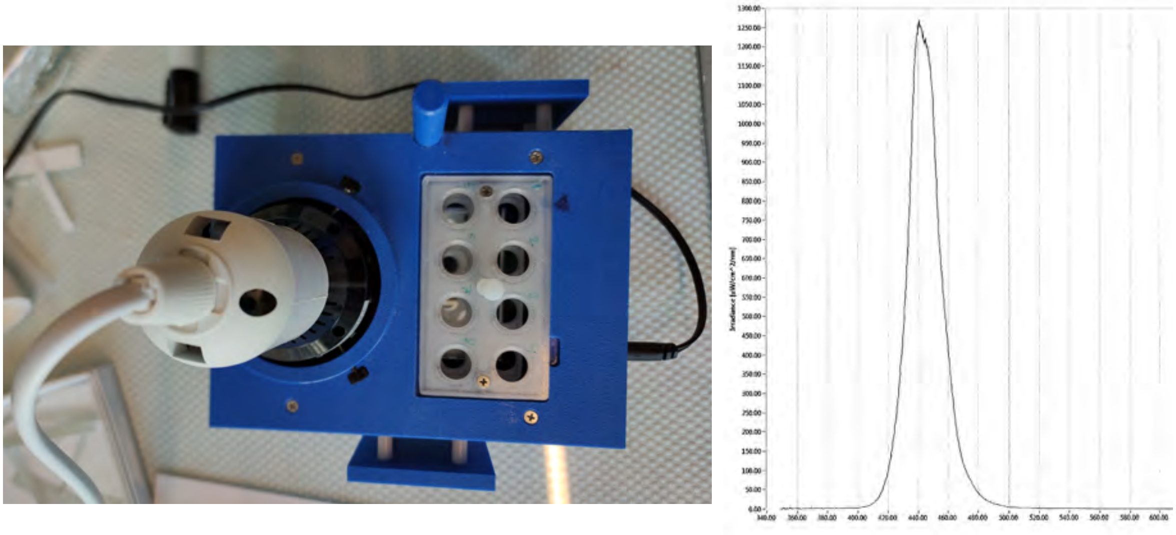

Figure S1. Left: Photochemical setup using the EvoluChem PhotoRedOx box™ including the custum-made holder. **Right:** Emission spectrum of the 450 nm LED lamp.

All photocatalysts (**1a-f**) were synthesized from the according fluorinated benzenecarbonitriles using the protocols reported by Zeitler and co-workers.[1] All benzenesulfonyl azides (**3-R**) were prepared by reacting the respective sulfonylchlorides with sodium azide according to a procedure by Laughlin and co-workers.[2] The precursor alkenes to aziridine products **4o**,[3] **4p**,[4] **4r**,[4] and **4v**[5] were prepared as previously described. The synthesis of the precursor alkene to aziridine **4z** is partly known and is outlined in Figure S2 below. From D-xylose, intermediate S1[6] could be obtained and transformed to S2[7]. Lewis acid-mediated debenzylation, followed by silyl protection afforded S3.

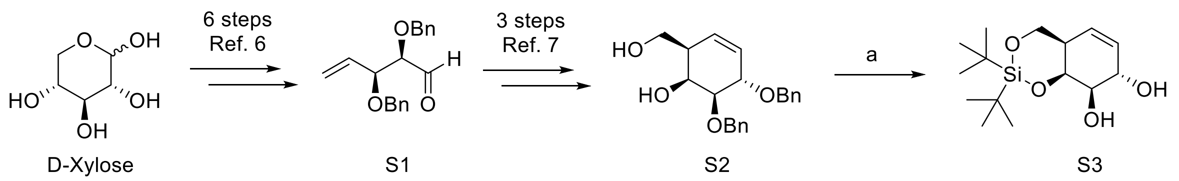

Figure S2. Synthesis of precursor alkene S3. a) i. BCl_3, DCM, -78 °C. ii. di-*tert*-butylsilanediyl bis(trifluoromethanesulfonate), pyridine, 66%, two steps.

2. Synthesis Of New Starting Materials

4-isopropoxybenzenesulfonyl azide (3-*i*-PrO)

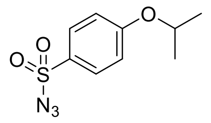

Prepared according to the procedure by Laughlin and co-workers.[2]

^{1}H NMR (400 MHz, $CDCl_3$) δ 7.91 – 7.83 (m, 2H), 7.05 – 6.97 (m, 2H,), 4.68 (hept, J = 6.0 Hz, 1H), 1.39 (d, J = 6.1 Hz, 6H).

^{13}C{^{1}H}-APT NMR (101 MHz, $CDCl_3$) δ 163.3, 129.9, 129.0, 116.0, 70.9, 21.7.

HRMS: Not detected.

(4aR,7S,8R,8aS)-2,2-di-tert-butyl-4a,7,8,8a-tetrahydro-4H-benzo[d][1,3,2]dioxasiline-7,8-diol (S3)

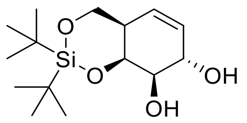

Compound S2 (Figure S2, 197 mg, 0.580 mmol, 1.0 eq.) was dissolved in dry DCM (4 mL). The reaction mixture was cooled to -78 °C and a BCl_3 solution in DCM (5.8 mL, 1 M, 10 eq.) was added dropwise. The reaction mixture was stirred at -78 °C for 3h and then quenched with the addition of methanol. The reaction mixture was concentrated and the crude product was filtered over a short silica plug, and washed with ethyl acetate. The product was then eluted by treating the silica plug with a 1:1 mixture of ethyl acetate:methanol. The solution was concentrated to afford a crude oil which was coevaporated thrice with toluene to remove traces of water. The crude was then dissolved in dry pyridine (11.6 mL). The solution was cooled to 0 °C with an ice bath and di-*tert*-butylsilanediyl bis(trifluoromethanesulfonate) (285 µL, 0.87 mmol, 1.5 eq.) was added. The reaction mixture was stirred at room temperature for 45 minutes and then quenched with the addition of methanol. Water was added and the mixture was extracted three times with ethyl acetate. The combined organic layers were washed two times with 1 M HCl, brine and then dried with $MgSO_4$, filtered and concentrated. The crude was purified by flash column chromatography (ethyl acetate/pentane; 1.0:4.0 → 2.3:1.0) to afford **S3** as a white solid (115 mg, 0.383 mmol, 66%).

^{1}H NMR (400 MHz, $CDCl_3$, H–H COSY, HSQC) δ 6.02 – 5.74 (m, 1H, H-7), 5.58 (dq, J = 10.1, 2.0 Hz, 1H, H-1), 4.65 – 4.58 (m, 1H, H-4 or H-2), 4.40 – 4.31 (m, 2H, H-2 or H-4, H-6), 4.09 (dd, J = 11.6, 1.8 Hz, 1H, H-6), 3.58 – 3.51 (m, 1H, H-3), 2.97 (s, 1H, OH), 2.85 (s, 1H, OH), 2.46 – 2.37 (m, 1H, H-5), 1.06 (s, 9H, CH_3), 0.93 (s, 9H, CH_3).

^{13}C{^{1}H}-APT NMR (101 MHz, $CDCl_3$, HSQC) δ 130.7 (C-7), 129.2 (C-1), 77.0 (C-3), 75.0 (C-2 or C-4), 71.2 (C-2 or C-4), 67.2 (C-6), 40.8 (C-5), 28.2 (CH_3), 27.3 (CH_3), 23.4 (C_q), 20.5 (C_q).

HRMS: $[M + H]^+$ calcd. for $C_{15}H_{29}O_4Si^+$ 301.1830; found 301.1830.

3. Reaction optimization

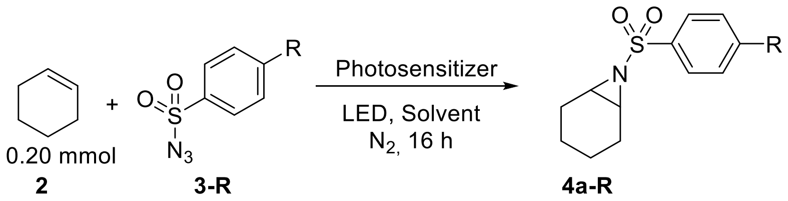

Table S1. Reaction optimization.[a]

Entry	Solvent	Photosensitizer (mol%)	LED	Azide (eq.)	Yield (%)[b]
1	MeCN (0.50 mL)	**1b** (5 mol%)	450 nm	**3-Me** (5.0)	9
2	Toluene (0.50 mL)	**1b** (5 mol%)	450 nm	**3-Me** (5.0)	9
3	DMF (0.50 mL)	**Eosin Y** (5 mol%)	525 nm	**3-Me** (5.0)	0
4	MeCN (0.50 mL)	**1b** (5 mol%)	450 nm	**3-OMe** (5.0)	9
5	MeCN (5.5 mL)[c]	**1a** (5 mol%)	450 nm	**3-OMe** (5.0)	17
6	MeCN (5.5 mL)[c]	**1d** (5 mol%)	450 nm	**3-OMe** (5.0)	27
7	DCM (0.50 mL)	**1a** (5 mol%)	450 nm	**3-OMe** (5.0)	15
8	DCM (0.50 mL)	**1d** (5 mol%)	450 nm	**3-OMe** (5.0)	54
9	DCM (0.50 mL)	**1c** (5 mol%)	450 nm	**3-OMe** (5.0)	48
10	DCM (0.50 mL)	**1e** (5 mol%)	450 nm	**3-OMe** (5.0)	60
11	DCM (0.50 mL)	**1f** (5 mol%)	450 nm	**3-OMe** (5.0)	25
12[d]	DCM (0.50 mL)	**1e** (5 mol%)	-	**3-OMe** (5.0)	0
13	DCM (0.50 mL)	**none**	450 nm	**3-OMe** (5.0)	0
14[e]	DCM (0.50 mL)	**1e** (5 mol%)	450 nm	**3-OMe** (5.0)	34
15	DCM (0.50 mL)	**1d** (5 mol%)	450 nm	**3-OMe** (2.5)	42
16	DCM (0.50 mL)	**1d** (5 mol%)	450 nm	**3-OMe** (7.5)	41
17	DCM (0.50 mL)	**1d** (5 mol%)	450 nm	**3-OMe** (1.0)	32
18	DCM (0.50 mL)	**1e** (5 mol%)	450 nm	**3-Me** (5.0)	62
18	DCM (1.0 mL)	**1e** (5 mol%)	450 nm	**3-Me** (5.0)	63
19	DCM (3.0 mL)	**1e** (5 mol%)	450 nm	**3-Me** (5.0)	61
20	DCM (1.0 mL)	**1e** (5 mol%)	450 nm	**3-Cl** (5.0)	53
21	DCM (1.0 mL)	**1e** (5 mol%)	450 nm	**3-CF_3** (5.0)	39

[a] Reactions performed as per GP1. [b] Yields were determined by ^{1}H NMR spectroscopy using 1,3,5-trimethoxybenzene as internal standard. [c] More solvent used to ensure homogeneity of the reaction mixture. [d] Performed in the dark in refluxing DCM. [e] Reaction done in air.

4. NMR reaction of 1a with 3-OMe on cyclohexene *(cf. Table 1)*

This reaction was performed as described in section 12, with the modification that no CH_2Br_2 was added. The stacked ^1H NMR spectra are displayed below in figure S3, including the observed color change of the reaction mixture. The figure shows that the aziridine forms but after around 150 minutes the reaction halts and no more aziridine is formed. Moreover we observe a clear discoloration of the reaction mixture as indicated in the figure. We attribute this to degradation of the photosensitizer. This experiment shows that the wavelength we use is sufficient to excite even the highest E_T photosensitizer.

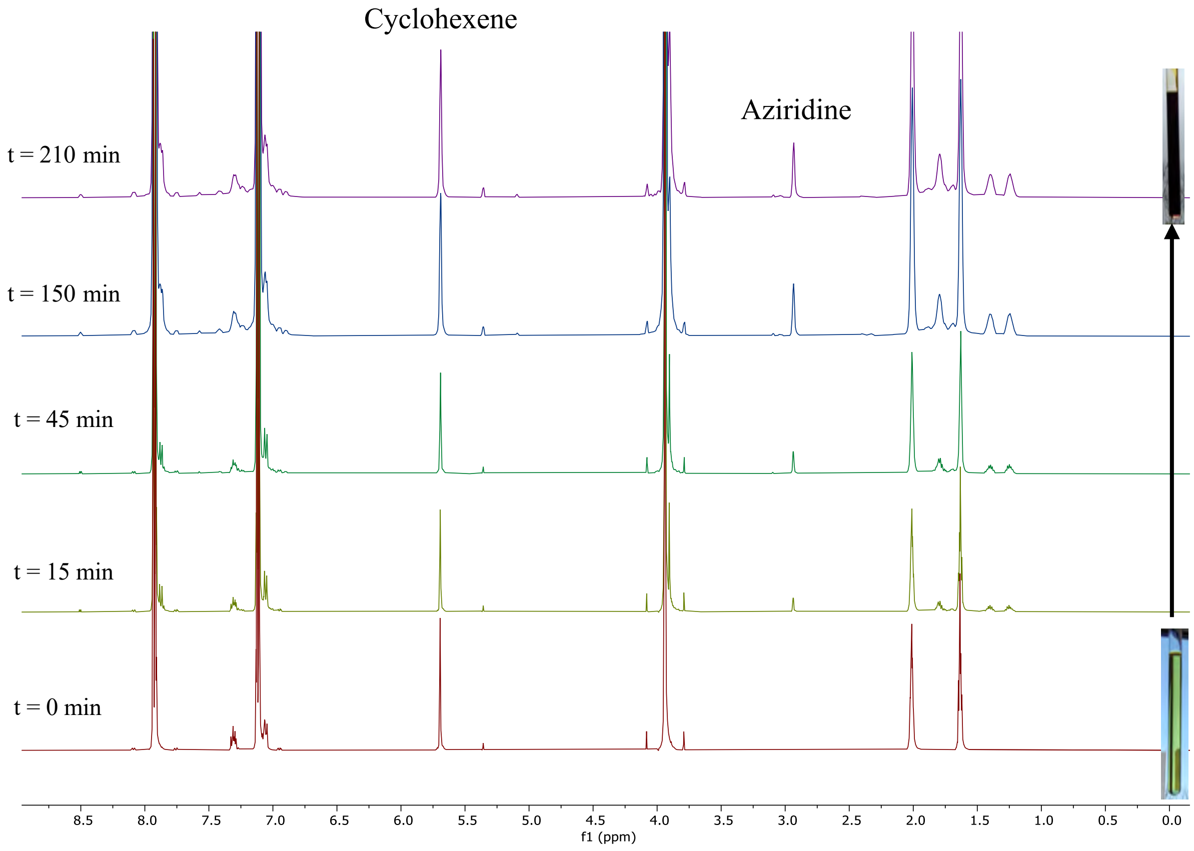

Figure S3. NMR reaction of 0.20 mmol **2**, 1.0 mmol **3-OMe** with 5 mol% **1a** in 0.50 mL CD_2Cl_2 followed over time with ^1H NMR spectroscopy. The indicated timepoints refer to cumulative irradiation times.

5. Substrate Scope *(cf. Table 2)*

GP1-General Procedure for the substrate scope of the catalytic aziridination reaction

A vial was charged with a stir bar and 153 µL (1.0 mmol) of tosyl azide (**3-Me**). In the case of a solid alkene, the alkene (0.20 mmol) was added at this point. The vial was entered in a Schlenk tube and the tube was deoxygenated by subjection to three vacuum and dinitrogen cycles ending on dinitrogen. In a separate Schlenk tube, a stock solution was made of 4CzPN (**1f**) in dry DCM (10 mM for a 5 mol% reaction and 6.0 mM for a 3 mol% reaction). This stock solution was deoxygenated by three freeze-pump-thaw cycles, ending on dinitrogen. Using standard syringe and needle techniques, 1.0 mL of the stock solution was transferred to the vial containing the azide. In the case of a liquid alkene, the alkene was passed over a short alumina pad and added to the vial at this point (0.20 mmol). The vial was closed with a screw cap, introduced in the photoreactor, and irradiated with blue LED lights for 16 h. The reaction mixture was then concentrated *in vacuo* and the crude product was analyzed by ^{1}H NMR spectroscopy. The product(s) were isolated by flash column chromatography.

7-tosyl-7-azabicyclo[4.1.0]heptane (4a-Me)

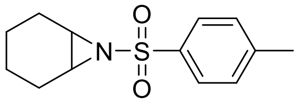

Prepared by **GP1** using 3 mol% 4CzPN. Crude purified by flash column chromatography (diethyl ether/pentane; 0.0:1.0 → 1.0:4.0) to afford **4a-Me** as a colorless oil (32 mg, 0.13 mmol, 63%).

^{1}H NMR (400 MHz, $CDCl_3$) δ 7.85 – 7.79 (m, 2H), 7.34 – 7.31 (m, 2H), 3.00 – 2.95 (m, 2H), 2.44 (s, 3H), 1.82 – 1.76 (m, 4H), 1.45 – 1.35 (m, 2H), 1.28 – 1.16 (m, 2H).

^{13}C{^{1}H}-APT NMR (101 MHz, $CDCl_3$) δ 144.1, 136.0, 129.7, 127.7, 39.9, 22.9, 21.7, 19.5.

Data in accordance with the literature.[8]

2-hexyl-1-tosylaziridine (4b)

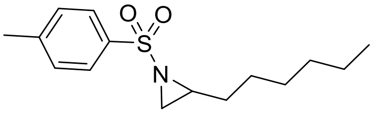

Prepared by **GP1** using 5 mol% 4CzPN. Crude purified by flash column chromatography (diethyl ether/pentane; 0.0:1.0 → 1.0:4.0) to afford **4b** as a colorless oil (28 mg, 0.10 mmol, 50%).

^{1}H NMR (400 MHz, $CDCl_3$, H−H COSY, HSQC) δ 7.87 – 7.79 (m, 2H, Ts), 7.36 – 7.32 (m, 2H, Ts), 2.71 (tt, J = 7.3, 4.7 Hz, 1H, CHN), 2.64 (d, J = 7.0 Hz, 1H, CH_2N), 2.45 (s, 3H, $PhCH_3$), 2.06 (d, J = 4.6 Hz, 1H, CH_2N), 1.58 – 1.49 (m, 1H, $NCHCH_2$), 1.36 – 1.28 (m, 1H, $NCHCH_2$), 1.24 – 1.10 (m, 8H), 0.85 (t, J = 7.0 Hz, 3H, CH_3 alkyl chain).

^{13}C{^{1}H}-APT NMR (101 MHz, $CDCl_3$, HSQC) δ 144.5 ($C_{q\text{-}arom}SO_2$), 135.3 ($C_{q\text{-}arom}CH_3$), 129.7 (CH_{arom}), 128.1 (CH_{arom}), 40.6 (CHN), 33.9 (CH_2N), 31.7 (CH_2 alkyl chain), 31.4 ($NCHCH_2$), 28.8 (CH_2 alkyl chain), 26.8 (CH_2 alkyl chain), 22.5 (CH_2 alkyl chain), 21.7 ($PhCH_3$), 14.2 (CH_3 alkyl chain).

Data in accordance with the literature.[9]

2,3-dipropyl-1-tosylaziridine (4c)

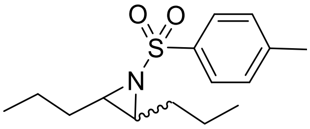

Prepared by **GP1** using 5 mol% 4CzPN. Crude purified by flash column chromatography (diethyl ether/pentane; 0.0:1.0 → 1.0:9.0) to afford aziridines **4c** as a colorless oil (50 mg, 0.18 mmol, 89%, 2:1 d.r.; *trans*:*cis*).

^{1}H NMR (400 MHz, $CDCl_3$, H−H COSY, HSQC) δ 7.86 – 7.80 (m, 2H, Ts), 7.35 – 7.28 (m, 2H, Ts), 2.84 – 2.73 (m, 2H, CHN), 2.68 – 2.61 (m, 2H, CHN), 2.44 (s, 3H, PhCH_3), 2.43 (s, 3H, PhCH_3), 1.81 – 1.16 (m, 8H), 0.93 – 0.85 (m, 6H, CH_3 alkyl tail).

^{13}C{^{1}H}-APT NMR (101 MHz, $CDCl_3$, HSQC) δ 144.3 ($C_{q\text{-arom}}SO_2$), 143.8 ($C_{q\text{-arom}}SO_2$), 138.1 ($C_{q\text{-arom}}CH_3$), 135.5 ($C_{q\text{-arom}}CH_3$), 129.6 (CH_{arom}), 129.5 (CH_{arom}), 128.1 (CH_{arom}), 127.5 (CH_{arom}), 49.8 (CHN), 45.1 (CHN), 32.0 (CH_2), 28.9 (CH_2), 21.7 (Ph*C*H_3), 21.7 (Ph*C*H_3), 20.9 (CH_2), 20.7 (CH_2), 13.9 (CH_3 alkyl chain), 13.8 (CH_3 alkyl chain).

Data in accordance with the literature.[9]

2-phenyl-1-tosylaziridine (4d)

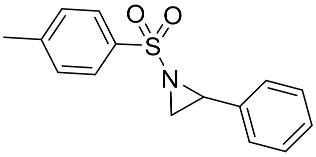

Prepared by **GP1** using 5 mol% 4CzPN. Crude purified by flash column chromatography (diethyl ether/pentane; 0.0:1.0 → 1.0:4.0) to afford **4d** as a colorless solid (31 mg, 0.11 mmol, 57%).

^{1}H NMR (400 MHz, $CDCl_3$) δ 7.90 – 7.84 (m, 2H), 7.34 – 7.31 (m, 2H), 7.30 – 7.24 (m, 3H), 7.23 – 7.17 (m, 2H), 3.77 (dd, J = 7.2, 4.5 Hz, 1H), 2.98 (d, J = 7.2 Hz, 1H), 2.43 (s, 3H), 2.38 (d, J = 4.4 Hz, 1H).

^{13}C{^{1}H}-APT NMR (101 MHz, $CDCl_3$) δ 144.8, 135.2, 135.1, 129.9, 128.7, 128.4, 128.1, 126.7, 41.1, 36.1, 21.8.

Data in accordance with the literature.[10]

2-(2-chlorophenyl)-1-tosylaziridine (4e)

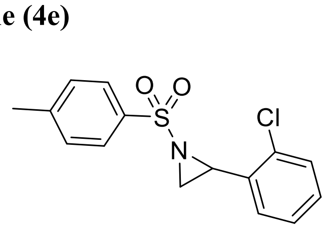

Prepared by **GP1** using 5 mol% 4CzPN. Crude purified by flash column chromatography (diethyl ether/pentane; 0.0:1.0 → 1.0:4.0) to afford **4e** as a colorless oil (34 mg, 0.11 mmol, 55%).

^{1}**H NMR** (400 MHz, $CDCl_3$, H−H COSY, HSQC) δ 7.94 – 7.86 (m, 2H, Ts), 7.38 – 7.34 (m, 2H, Ts), 7.32 (dt, J = 7.3, 1.2 Hz, 1H, Ph), 7.25 – 7.12 (m, 3H, Ph), 4.04 (dd, J = 7.2, 4.4 Hz, 1H, CHN), 3.03 (d, J = 7.2 Hz, 1H, CH_2N), 2.45 (s, 3H, CH_3), 2.29 (d, J = 4.3 Hz, 1H, CH_2N).

^{13}**C{^{1}H}-APT NMR** (101 MHz, $CDCl_3$, HSQC) δ 145.0 ($C_{q\text{-arom}}SO_2$), 134.8 ($C_{q\text{-arom}}$), 133.9 ($C_{q\text{-arom}}$), 133.2 ($C_{q\text{-arom}}$), 129.9 (CH_{arom} Ts), 129.4 (CH_{arom} Ph), 129.3 (CH_{arom} Ph), 128.2 (CH_{arom} Ts), 127.6 (CH_{arom} Ph), 127.1 (CH_{arom} Ph), 39.1 (CHN), 35.7 (CH_2N), 21.8 (CH_3).

Data in accordance with the literature.[11]

1-tosyl-2-(2-(trifluoromethyl)phenyl)aziridine (4f)

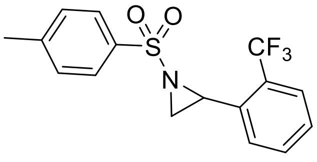

Prepared by **GP1** using 5 mol% 4CzPN. Crude purified by flash column chromatography (diethyl ether/pentane; 0.0:1.0 → 1.0:9.0) to afford **4f** as a colorless oil (44 mg, 0.13 mmol, 64%).

^{1}**H NMR** (400 MHz, $CDCl_3$, H−H COSY, HSQC) δ 7.93 – 7.86 (m, 2H, Ts), 7.61 (d, J = 7.7 Hz, 1H, Ph), 7.50 – 7.32 (m, 5H, Ts + Ph), 4.04 (ddt, J = 6.1, 4.6, 1.8 Hz, 1H, CHN), 3.02 (d, J = 7.3 Hz, 1H, CH_2N), 2.45 (s, 3H, CH_3), 2.29 (d, J = 4.4 Hz, 1H, CH_2N).

^{13}**C{^{1}H}-APT NMR** (101 MHz, $CDCl_3$, HSQC) δ 145.1 ($C_{q\text{-arom}}SO_2$), 134.5 ($C_{q\text{-arom}}$), 133.8 ($C_{q\text{-arom}}$), 132.4 (CH_{arom}), 130.0 (CH_{arom}), 128.8 (q appd, $^{2}J_{CF}$ = 31.1 Hz, $C_{q\text{-arom}}CF_3$), 128.2 (CH_{arom}), 128.2 (CH_{arom}), 127.8 (CH_{arom}), 125.81 (q, $^{3}J_{CF}$ = 5.5 Hz, CH_{arom} *ortho* to CF_3), 124.2 (q appd, $^{1}J_{CF}$ = 273.7 Hz, CF_3), 38.2 (q, $^{4}J_{CF}$ = 2.7 Hz, CHN), 36.5 (CH_2N), 21.8 (CH_3).

^{19}**F NMR** (376 MHz, $CDCl_3$) δ -60.1 (s, 3F).

HRMS: $[M + H]^+$ calcd. for $C_{16}H_{15}F_3NO_2S^+$ 342.0770; found 342.0772.

2-(o-tolyl)-1-tosylaziridine (4g)

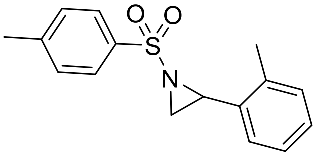

Prepared by **GP1** using 5 mol% 4CzPN. Crude purified by flash column chromatography (diethyl ether/pentane; 0.0:1.0 → 1.0:4.0) to afford **4g** as a colorless oil (34 mg, 0.12 mmol, 59%).

^{1}**H NMR** (400 MHz, $CDCl_3$, H−H COSY, HSQC) δ 7.93 – 7.86 (m, 2H, Ts), 7.38 – 7.31 (m, 2H, Ts), 7.22 – 7.06 (m, 4H, Ph), 3.86 (dd, J = 7.2, 4.5 Hz, 1H, C*H*N), 2.98 (d, J = 7.2 Hz, 1H, CH_2N), 2.44 (s, 3H, CH_3 Ts), 2.38 (s, 3H, PhCH_3), 2.31 (d, J = 4.5 Hz, 1H, CH_2N).

^{13}**C{^{1}H}-APT NMR** (101 MHz, $CDCl_3$, HSQC) δ 144.8 ($C_{q\text{-arom}}SO_2$), 136.9 ($C_{q\text{-arom}}$), 135.1 ($C_{q\text{-arom}}$), 133.3 ($C_{q\text{-arom}}$), 130.1 (CH_{arom} *o*-MePh), 129.9 (CH_{arom} Ts), 128.2 (CH_{arom} *o*-MePh), 128.1 (CH_{arom} Ts), 126.2 (CH_{arom} *o*-MePh), 126.0 (CH_{arom} *o*-MePh), 39.6 (CHN), 35.2 (CH_2N), 21.8 (CH_3 tosyl), 19.2 (CH_3 *o*-MePh).

Data in accordance with the literature.[10]

2-(3-chlorophenyl)-1-tosylaziridine (4h)

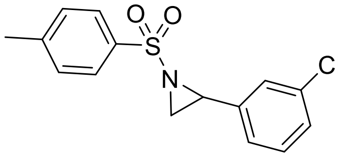

Prepared by **GP1** using 5 mol% 4CzPN. Crude purified by flash column chromatography (diethyl ether/pentane; 0.0:1.0 → 1.0:4.0) to afford **4h** as a colorless oil (34 mg, 0.11 mmol, 55%).

^{1}H NMR (400 MHz, $CDCl_3$, H−H COSY, HSQC) δ 7.90 – 7.83 (m, 2H, Ts), 7.38 – 7.31 (m, 2H, Ts), 7.28 – 7.16 (m, 3H, Ph), 7.12 (dt, J = 6.7, 1.9 Hz, 1H, Ph), 3.73 (dd, J = 7.1, 4.3 Hz, 1H, CHN), 2.97 (d, J = 7.1 Hz, 1H, CH_2N), 2.44 (s, 3H, CH_3), 2.35 (d, J = 4.4 Hz, 1H, CH_2N).

^{13}C{^{1}H}-APT NMR (101 MHz, $CDCl_3$, HSQC) δ 145.0 ($C_{q\text{-arom}}SO_2$), 137.3 ($C_{q\text{-arom}}$), 134.8 ($C_{q\text{-arom}}$), 134.7 ($C_{q\text{-arom}}$), 130.0 (CH_{arom}), 130.0 (CH_{arom}), 128.6 (CH_{arom}), 128.1 (CH_{arom}), 126.7 (CH_{arom}), 125.0 (CH_{arom}), 40.2 (CHN), 36.3 (CH_2N), 21.8 (CH_3).

Data in accordance with the literature.[11]

1-tosyl-2-(3-(trifluoromethyl)phenyl)aziridine (4i)

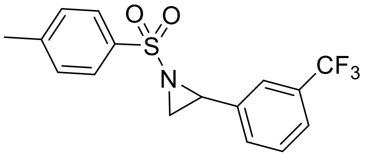

Prepared by **GP1** using 5 mol% 4CzPN. Crude purified by flash column chromatography (diethyl ether/pentane; 0.0:1.0 → 1.0:4.0) to afford **4i** as a colorless oil (44 mg, 0.13 mmol, 64%).

^{1}H NMR (400 MHz, $CDCl_3$, H−H COSY, HSQC) δ 7.90 – 7.86 (m, 2H, Ts), 7.53 (td, J = 4.6, 3.8, 2.2 Hz, 1H, CH_{arom}), 7.46 – 7.41 (m, 3H, CH_{arom}), 7.35 (d, J = 8.1 Hz, 2H, Ts), 3.81 (dd, J = 7.2, 4.3 Hz, 1H, CHN), 3.01 (d, J = 7.2 Hz, 1H, CH_2N), 2.44 (s, 3H, CH_3), 2.38 (d, J = 4.3 Hz, 1H, CH_2N).

^{13}C{^{1}H}-APT NMR (101 MHz, $CDCl_3$, HSQC) δ 145.1 ($C_{q\text{-arom}}SO_2$), 136.4 ($C_{q\text{-arom}}$), 134.7 ($C_{q\text{-arom}}$), 131.2 (q, $^{2}J_{CF}$ = 32.4 Hz, $C_{q\text{-arom}}CF_3$), 130.1 (CH_{arom} Ph), 130.0 (CH_{arom} Ts), 129.3 (CH_{arom} Ph), 128.1 (CH_{arom} Ts), 125.3 (q, $^{3}J_{CF}$ = 3.8 Hz, CH_{arom}), 123.5 (q, $^{3}J_{CF}$ = 3.8 Hz, CH_{arom}), 121.2 (q appd, $^{1}J_{CF}$ = 272.2 Hz, CF_3), 40.2 (CHN), 36.3 (CH_2N), 21.8 (CH_3).

^{19}F NMR (471 MHz, $CDCl_3$) δ -63.0 (s, 3F).

HRMS: $[M + H]^+$ calcd. for $C_{16}H_{15}F_3NO_2S^+$ 342.0770; found 342.0770.

2-(m-tolyl)-1-tosylaziridine (4j)

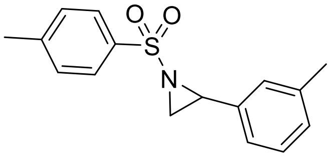

Prepared by **GP1** using 5 mol% 4CzPN. Crude purified by flash column chromatography (diethyl ether/pentane; 0.0:1.0 → 1.0:4.0) to afford **4j** as a colorless oil (37 mg, 0.13 mmol, 64%).

^{1}H NMR (400 MHz, $CDCl_3$, H–H COSY, HSQC) δ 7.91 – 7.83 (m, 2H, Ts), 7.37 – 7.29 (m, 2H, Ts), 7.17 (t, J = 7.8 Hz, 1H, Ph), 7.08 (d, J = 7.2 Hz, 1H, Ph), 7.04 – 6.98 (m, 2H, Ph), 3.74 (dd, J = 7.1, 4.5 Hz, 1H, CHN), 2.96 (d, J = 7.2 Hz, 1H, CH_2N), 2.43 (s, 3H, CH_3 Ts), 2.38 (d, J = 4.5 Hz, 1H, CH_2N), 2.30 (s, 3H, CH_3 Ph).

^{13}C{^{1}H}-APT NMR (101 MHz, $CDCl_3$, HSQC) δ 144.7 ($C_{q\text{-arom}}SO_2$), 138.4 ($C_{q\text{-arom}}$), 135.1 ($C_{q\text{-arom}}$), 135.0 ($C_{q\text{-arom}}$), 129.9 (CH_{arom} tosyl), 129.2 (CH_{arom} *m*-MePh), 128.6 (CH_{arom} *m*-MePh), 128.1 (CH_{arom} tosyl), 127.3 (CH_{arom} *m*-MePh), 123.8 (CH_{arom} *m*-MePh), 41.2 (CHN), 36.0 (CH_2N), 21.8 (CH_3 tosyl), 21.4 (CH_3 *m*-MePh).

Data in accordance with the literature.[10]

2-(4-chlorophenyl)-1-tosylaziridine (4k)

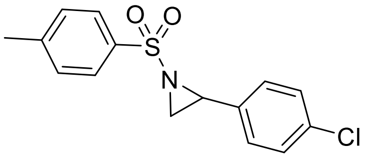

Prepared by **GP1** using 5 mol% 4CzPN. Crude purified by flash column chromatography (diethyl ether/pentane; 0.0:1.0 → 1.0:4.0) to afford **4k** as a pale yellow oil (33 mg, 0.11 mmol, 53%).

^{1}H NMR (400 MHz, $CDCl_3$, H–H COSY, HSQC) δ 7.89 – 7.82 (m, 2H, Ts), 7.37 – 7.30 (m, 2H, Ts), 7.30 – 7.22 (m, 2H, Ph), 7.19 – 7.11 (m, 2H, Ph), 3.73 (dd, J = 7.2, 4.4 Hz, 1H, CHN), 2.98 (d, J = 7.2 Hz, 1H, CH_2N), 2.43 (s, 3H, CH_3), 2.34 (d, J = 4.4 Hz, 1H, CH_2N).

^{13}C{^{1}H}-APT NMR (101 MHz, $CDCl_3$, HSQC) δ 144.9 ($C_{q\text{-arom}}SO_2$), 134.9 ($C_{q\text{-arom}}$), 134.3 ($C_{q\text{-arom}}$), 133.8 ($C_{q\text{-arom}}$), 129.9 (CH_{arom} Ts), 128.9 (CH_{arom} Ph), 128.1 (CH_{arom} Ts or Ph), 128.0 (CH_{arom} Ts or Ph), 40.4 (CHN), 36.2 (CH_2N), 21.8 (CH_3).

Data in accordance with the literature.[12]

1-tosyl-2-(4-(trifluoromethyl)phenyl)aziridine (4l)

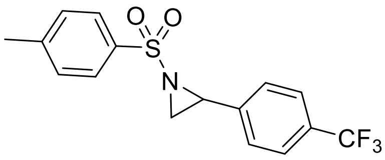

Prepared by **GP1** using 5 mol% 4CzPN. Crude purified by flash column chromatography (diethyl ether/pentane; 0.0:1.0 → 1.0:4.0) to afford **4l** as a colorless oil (40 mg, 0.12 mmol, 58%).

^{1}H NMR (400 MHz, $CDCl_3$, H–H COSY, HSQC) δ 7.91 – 7.83 (m, 2H, Ts), 7.55 (d, J = 8.0 Hz, 2H, Ts), 7.38 – 7.31 (m, 4H, Ph), 3.81 (dd, J = 7.2, 4.3 Hz, 1H, CHN), 3.02 (d, J = 7.2 Hz, 1H, CH_2N), 2.44 (s, 3H, CH_3), 2.37 (d, J = 4.3 Hz, 1H, CH_2N).

^{13}C{^{1}H}-APT NMR (101 MHz, $CDCl_3$, HSQC) δ 145.1 ($C_{q\text{-arom}}SO_2$), 139.3 ($C_{q\text{-arom}}$), 134.7 ($C_{q\text{-arom}}$), 130.6 (q appd, $^{2}J_{CF}$ = 32.5 Hz), 130.0 (CH_{arom}), 128.1 (CH_{arom}), 127.0 (CH_{arom}), 125.7 (q, $^{3}J_{CF}$ = 3.8 Hz, CH_{arom}), 124.0 (q appd, $^{1}J_{CF}$ = 272.1 Hz), 40.2 (CHN), 36.3 (CH_2N), 21.8 (CH_3).

^{19}F NMR (376 MHz, $CDCl_3$) δ -62.9 (s, 3F).

Data in accordance with the literature.[13]

2-(p-tolyl)-1-tosylaziridine (4m)

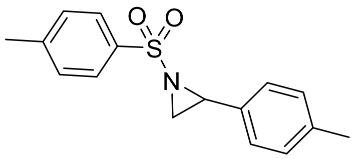

Prepared by **GP1** using 5 mol% 4CzPN. Crude purified by flash column chromatography (diethyl ether/pentane; 0.0:1.0 → 1.0:4.0) to afford **4m** as a colorless oil (28 mg, 0.10 mmol, 48%).

^{1}H NMR (400 MHz, $CDCl_3$, H−H COSY, HSQC) δ 7.90 – 7.82 (m, 2H, Ts), 7.37 – 7.26 (m, 2H, Ts), 7.10 (s, 4H, Ph), 3.74 (dd, J = 7.2, 4.5 Hz, 1H, CHN), 2.97 (d, J = 7.2 Hz, 1H, CH_2N), 2.43 (s, 3H, CH_3 Ts), 2.38 (d, J = 4.5 Hz, 1H, CH_2N), 2.31 (s, 3H, $C\mathit{H_3}Ph$).

^{13}C{^{1}H}-APT NMR (101 MHz, $CDCl_3$, HSQC) δ 144.7 ($C_{q\text{-arom}}SO_2$), 138.3 ($C_{q\text{-arom}}$), 135.2 ($C_{q\text{-arom}}$), 132.1 ($C_{q\text{-arom}}$), 129.9 (CH_{arom} Ts), 129.4 (CH_{arom} Ph), 128.1 (CH_{arom} Ts), 126.6 (CH_{arom} Ph), 41.2 (CHN), 35.9 (CH_2N), 21.8 (CH_3 Ts), 21.3 (CH_3 Ph).

Data in accordance with the literature.[10]

3-(2-bromoethyl)-2,2-dimethyl-1-tosylaziridine (4n)

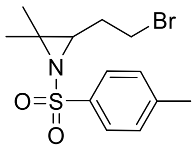

Prepared by **GP1** using 3 mol% 4CzPN. Crude purified by flash column chromatography (diethyl ether/pentane; 0.0:1.0 → 1.0:4.0) to afford **4n** as a colorless solid (49 mg, 0.15 mmol, 74%).

^{1}H NMR (500 MHz, $CDCl_3$, H−H COSY, HSQC) δ 7.89 – 7.84 (m, 2H, Ts), 7.35 – 7.31 (m, 2H, Ts), 3.24 (ddd, J = 10.0, 6.6, 4.9 Hz, 1H, CH_2Br), 3.07 – 3.00 (m, 2H, CH_2Br, CHN), 2.46 (s, 3H, CH_3Ph), 2.10 – 2.01 (m, 1H, $CHC\mathit{H_2}$), 1.87 (dddd, J = 14.5, 7.9, 6.1, 4.9 Hz, 1H, $CHC\mathit{H_2}$), 1.76 (s, 3H, CH_3C_qN), 1.33 (s, 3H, CH_3C_qN).

^{13}C{^{1}H}-APT NMR (126 MHz, $CDCl_3$, HSQC) δ 144.1 ($C_{q\text{-arom}}SO_2$), 138.0 ($\mathit{C_{q\text{-arom}}}CH_3$), 129.6 ($CH_{arom}$), 127.6 ($CH_{arom}$), 51.9 ($C_qN$), 50.8 (CHN), 31.3 ($CH\mathit{C}H_2$), 30.1 ($CH_2Br$), 21.7 ($CH_3$), 21.7 ($CH_3$), 21.2 ($CH_3$).

HRMS: $[M + H]^+$ calcd. For $C_{13}H_{19}BrNO_2S^+$ 332.0314 (^{79}Br) and 334.0293 (^{81}Br); found 332.0315 (^{79}Br) and 334.0292 (^{81}Br).

3-(2-(3,3-dimethyl-1-tosylaziridin-2-yl)ethyl)cyclohex-2-en-1-one (4o)

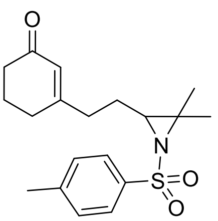

Prepared by **GP1** using 3 mol% 4CzPN. Crude purified by flash column chromatography (diethyl ether/pentane; 1.0:1.5 → 1.0:0.0) to afford **4o** as a colorless oil (34 mg, 0.10 mmol, 45%).

^{1}H NMR (400 MHz, $CDCl_3$, H–H COSY, HSQC) δ 7.83 – 7.79 (m, 2H, Ts), 7.32 – 7.28 (m, 2H, Ts), 2.82 (dd, *J* = 8.2, 5.1 Hz, 1H, CHN), 2.42 (s, 3H, CH_3Ph), 2.31 (dd, *J* = 7.6, 5.8 Hz, 2H, endocyclic $CH_2C_{q,alkene}$), 2.15 – 2.05 (m, 3H, CH_2C=O + $CH_2C_{q,alkene}$ exocyclic), 2.00 – 1.90 (m, 3H, $CH_2\mathit{CH_2}CH_2$ + $CH_2C_{q,alkene}$ exocyclic), 1.75 – 1.64 (m, 4H, CH_3 + $\mathit{CH_2}$CHN), 1.50 – 1.42 (m, 1H, $\mathit{CH_2}$CHN), 1.27 (s, 3H, CH_3).

^{13}C{^{1}H}-APT NMR (101 MHz, $CDCl_3$, HSQC) δ 199.7 (C_q=O), 164.5 (C_q enone), 144.0 ($C_{q\text{-}arom}SO_2$), 138.1 ($\mathit{C_{q\text{-}arom}}CH_3$), 129.6 ($CH_{arom}$), 127.5 ($CH_{arom}$), 125.7 (CH enone), 52.0 (C_qN), 51.7 (CHN), 37.3 (endocyclic $\mathit{CH_2}C_{q,alkene}$), 35.5 (exocyclic $\mathit{CH_2}C_{q,alkene}$), 29.8 ($\mathit{CH_2}C_q$=O), 25.5 ($\mathit{CH_2}$CHN), 22.6 ($CH_2\mathit{CH_2}CH_2$), 21.7 ($\mathit{CH_3}$Ph), 21.3 ($CH_3$), 21.2 ($CH_3$).

HRMS: $[M + H]^+$ calcd. for $C_{19}H_{26}NO_3S^+$ 348.1628; found 348.1629.

***Trans*-2-((tert-butyldimethylsilyl)oxy)-7-tosyl-7-azabicyclo[4.1.0]heptane (4p)**

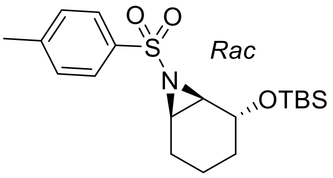

Prepared by **GP1** using 3 mol% 4CzPN. Crude purified by flash column chromatography (diethyl ether/pentane; 0.0:1.0 → 1.0:9.0) to afford **4p** as a pale yellow oil (51 mg, 0.13 mmol, 67%).

^{1}H NMR (400 MHz, $CDCl_3$, H–H COSY, HSQC) δ 7.86 – 7.77 (m, 2H, Ts), 7.37 – 7.29 (m, 2H, Ts), 3.82 (dd, *J* = 7.5, 5.2 Hz, 1H, CHOTBS), 3.07 (ddd, *J* = 6.9, 4.1, 1.4 Hz, 1H, $CH_2C\mathit{H}N$), 2.81 (dd, *J* = 6.9, 0.9 Hz, 1H, $CHC\mathit{H}N$), 2.44 (s, 3H, $PhC\mathit{H_3}$), 1.87 – 1.78 (m, 1H, *H*-CH), 1.77 – 1.63 (m, 2H, CH_2), 1.49 – 1.41 (m, 1H, *H*-CH_2), 1.23 – 1.10 (m, 2H, CH_2), 0.85 (s, 9H, *t*-Bu), -0.01 (s, 3H, $SiCH_3$), -0.02 (s, 3H, $SiCH_3$).

^{13}C{^{1}H}-APT NMR (101 MHz, $CDCl_3$, HSQC) δ 144.4 ($C_{q\text{-}arom}SO_2$), 135.5 ($\mathit{C_{q\text{-}arom}}CH_3$), 129.8 ($CH_{arom}$), 128.0 ($CH_{arom}$), 66.2 (*C*HOTBS), 44.9 (CH*C*HN), 40.8 ($CH_2\mathit{C}HN$), 30.4 (CH_2), 25.9 (*C*H_3 *t*-Bu), 22.7 (CH_2), 21.8 (*C*H_3Ph), 18.2 (C_q *t*-Bu), 15.1 (CH_2), -4.8 (CH_3Si), -4.9 (CH_3Si).

HRMS: $[M + H]^+$ calcd. for $C_{19}H_{32}NO_3SSi^+$ 382.1867; found 382.1866.

7-tosyl-7-azabicyclo[4.1.0]heptan-2-ol (4q)

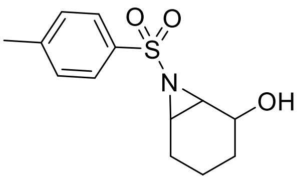

Prepared by **GP1** using 3 mol% 4CzPN. Crude purified by flash column chromatography (diethyl ether/pentane; 1.0:1.5 → 4.0:1.0) to afford aziridines **4q** as a colorless oil (29 mg, 0.11 mmol, 54%, syn:anti = 1.4:1.0).

^{1}H NMR (400 MHz, $CDCl_3$, H–H COSY, HSQC) δ 7.86 – 7.79 (m, 2H, Ts, *syn*), 7.83 – 7.76 (m, 2H, Ts, *anti*), 7.38 – 7.29 (m, 2H, Ts), 3.98 – 3.90 (m, 1H, C*H*OH), 3.23 – 3.13 (m, 2H, C*H*NC*H*, *syn*), 3.04

(ddd, J = 6.9, 4.2, 1.5 Hz, 1H, $CH_2C\mathit{H}N$, *anti*), 2.94 (dd, J = 6.8, 0.9 Hz, 1H, $OCHC\mathit{H}N$, *anti*), 2.44 (s, 3H, CH_3), 1.85 – 1.64 (m, 3H, $C\mathit{H_2}$), 1.60 – 1.43 (m, 1H, $C\mathit{H_2}$), 1.40 – 1.31 (m, 1H, $C\mathit{H_2}$), 1.23 – 1.12 (m, 1H, $C\mathit{H_2}$,).

^{13}C{^{1}H}-APT NMR (101 MHz, $CDCl_3$, HSQC) δ 144.8 ($C_{q\text{-arom}}SO_2$, *syn*), 144.5 ($C_{q\text{-arom}}SO_2$, *anti*), 135.2 ($\mathit{C_{q\text{-}arom}}CH_3$, *anti*), 134.9 ($\mathit{C_{q\text{-}arom}}CH_3$, *syn*), 129.9 ($CH_{arom}$, *syn*), 129.8 ($CH_{arom}$, *anti*), 128.0 ($CH_{arom}$, *syn*), 127.9 ($CH_{arom}$, *anti*), 65.3 (CHOH, *anti*) 64.8 (CHOH, *syn*), 44.5 (*C*HCHOH, *syn*), 44.0 (*C*HCHOH, *anti*), 42.7 (N*C*HCH$_2$, *syn*), 40.5 (N*C*HCH$_2$, *anti*), 29.6 (CH_2, *anti*), 29.2 (CH_2, *syn*), 22.6 (CH_2, *anti*), 21.9 (CH_2, *syn*), 21.8 (CH_3, *syn*), 21.8 (CH_3, *anti*), 18.5 (CH_2, *syn*), 14.9 (CH_2, *anti*).

HRMS: $[M + H]^+$ calcd. for $C_{13}H_{18}NO_3S^+$ 268.1002; found 268.1002.

3-(((tert-butyldimethylsilyl)oxy)methyl)-7-tosyl-7-azabicyclo[4.1.0]heptane (4r)

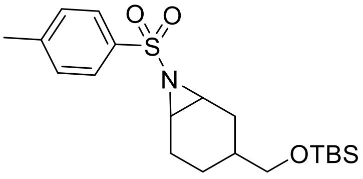

Prepared by **GP1** using 3 mol% 4CzPN. Crude purified by flash column chromatography (diethyl ether/pentane; 0.0:1.0 → 1.0:9.0) to afford **4r** as a colorless oil (66 mg, 0.17 mmol, 83%, syn:anti=1.0:1.0).

^{1}H NMR (400 MHz, $CDCl_3$, H−H COSY, HSQC) δ 7.84 – 7.77 (m, 2H, Ts), 7.31 (d, J = 8.0 Hz, 2H, Ts), 3.41 – 3.26 (m, 2H, CH_2OTBS), 3.03 (ddd, J = 7.2, 3.4, 1.6 Hz, 1H, NCH, one isomer), 3.01 – 2.92 (m, 3H, NCH, *syn* + *anti*), 2.43 (s, 3H, $PhC\mathit{H_3}$), 2.09 – 1.87 (m, 2H), 1.75 – 1.55 (m, 2H), 1.52 – 1.44 (m, 1H, one isomer) 1.42 – 1.28 (m, 2H), 1.13 – 1.00 (m, 1H), 1.00 – 0.90 (m, 1H) 0.84 (s, 9H, *t*-Bu), -0.02 (s, 6H, $Si(CH_3)_2$).

^{13}C{^{1}H}-APT NMR (101 MHz, $CDCl_3$, HSQC) δ 144.1 ($C_{q\text{-arom}}SO_2$, *syn* + *anti*), 135.9 ($\mathit{C_{q\text{-}arom}}CH_3$, one isomer), 135.9 ($\mathit{C_{q\text{-}arom}}CH_3$, one isomer), 129.7 ($CH_{arom}$, *syn* + *anti*), 127.7 (CH_{arom}, *syn* + *anti*), 67.6 (*C*H$_2$OTBS, one isomer), 67.3 (*C*H$_2$OTBS, one isomer), 41.0 (NCH, one isomer), 40.7 (NCH, one isomer), 39.6 (NCH, one isomer), 39.2 (NCH, one isomer), 35.6 (*C*HCH$_2$OH, one isomer), 32.6 (*C*HCH$_2$OH, one isomer), 26.4 (CH_2, one isomer), 26.0 (*C*H$_3$ *t*-Bu), 25.7 (CH_2, one isomer), 23.8 (CH_2, one isomer), 23.5 (CH_2, one isomer), 21.8 (CH_2, one isomer), 21.7 (*C*H$_3$Ph, *syn* + *anti*), 21.3 (CH_2, one isomer), 18.4 (C_q *t*-Bu, *syn* + *anti*), -5.3 (CH_3Si, *syn* + *anti*), -5.4 (CH_3Si, *syn* + *anti*).

HRMS: $[M + H]^+$ calcd. for $C_{20}H_{34}NO_3SSi^+$ 396.2023; found 396.2023.

(7-tosyl-7-azabicyclo[4.1.0]heptan-3-yl)methanol (4s)

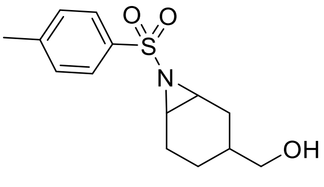

Prepared by **GP1** using 3 mol% 4CzPN. Crude purified by flash column chromatography (diethyl ether/pentane; 1.0:1.5 → 1.0:0.0) to afford aziridines **4s** as a colorless oil (41 mg, 0.15 mmol, 74%, syn:anti = 1.0:1.0).

^{1}H NMR (400 MHz, $CDCl_3$, H−H COSY, HSQC) δ 7.83 – 7.74 (m, 2H, Ts), 7.35 – 7.27 (m, 2H, Ts), 3.46 – 3.28 (m, 2H, C*H*2OH), 3.04 (ddd, *J* = 7.1, 3.4, 1.6 Hz, 1H, NC*H*, one isomer), 3.01 – 2.95 (m, 2H, NC*H*), 2.98 – 2.92 (m, 1H, NC*H*, one isomer), 2.43 (s, 3H, CH_3), 2.08 – 1.93 (m, 2H, CH_2), 1.75 – 1.58 (m, 2H, CH_2, *syn* + *anti*, CH_2C*H*CH_2, one isomer), 1.58 – 1.47 (m, 1H, *H*-CH, one isomer), 1.46 – 1.42 (m, 1H, *H*-CH, one isomer), 1.41 – 1.29 (m, 1H, CH_2C*H*CH_2), 1.09 (qd, *J* = 13.8, 13.0, 4.4 Hz, 1H, *H*-CH, one isomer), 0.93 (dtd, *J* = 13.2, 11.6, 6.6 Hz, 1H, *H*-CH, one isomer).

^{13}C{^{1}H}-APT NMR (101 MHz, $CDCl_3$, HSQC) δ 144.3 ($C_{q\text{-}arom}SO_2$, *syn* + *anti*), 135.7 ($C_{q\text{-}arom}CH_3$, one isomer), 135.7 ($C_{q\text{-}arom}CH_3$, one isomer), 129.7 (CH_{arom}, *syn* + *anti*), 127.7 (CH_{arom}, *syn* + *anti*), 67.4 (CH_2OH, one isomer), 67.2 (CH_2OH, one isomer), 40.8 (NCH, one isomer), 40.6 (NCH, one isomer), 39.4 (NCH, one isomer), 39.0 (NCH, one isomer), 35.4 (*C*HCH_2OH, one isomer), 32.7 (*C*HCH_2OH, one isomer), 26.5 (CH_2, one isomer), 25.6 (CH_2, one isomer), 23.7 (CH_2, one isomer), 23.4 (CH_2, one isomer), 21.7 (CH_3, *syn* + *anti*), 21.7 (CH_2, one isomer), 21.3 (CH_2, one isomer).

HRMS: $[M + H]^+$ calcd. for $C_{14}H_{20}NO_3S^+$ 282.1158; found 282.1159.

(3S,4aS,5aR,6bS,9R,9aR,11aS,11bR)-9a,11b-dimethyl-9-((R)-6-methylheptan-2-yl)-5-tosylhexadecahydro-2H-cyclopenta[1,2]phenanthro[8a,9-b]azirin-3-ol (4t)

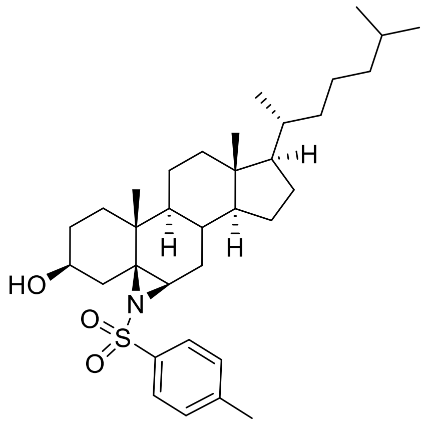

Prepared by **GP1** using 3 mol% 4CzPN. Crude purified by flash column chromatography (diethyl ether/pentane; 1.0:1.0 → 2.3:1.0) to afford **4t** as a colorless oil (61 mg, 0.11 mmol, 41%). The other diastereomer was detected in the crude ^{1}H NMR spectrum but was not isolated and based on the relative integrals (3:1) should amount to 14% product making the total yield 55%.

^{1}H NMR (400 MHz, $CDCl_3$, H−H COSY, H−H NOESY, HSQC) δ 7.82 – 7.75 (m, 2H, Ts), 7.31 – 7.24 (m, 2H, Ts), 3.87 (dq, *J* = 10.7, 5.7 Hz, 1H, CHO), 3.17 – 3.11 (m, 1H, CHN), 2.44 – 2.39 (m, 4H), 2.40 – 2.24 (m, 1H), 1.93 (dt, *J* = 12.4, 3.3 Hz, 1H), 1.87 – 1.69 (m, 4H), 1.54 – 1.41 (m, 1H), 1.37 – 1.22 (m, 3H), 1.21 – 1.03 (m, 2H), 1.03 – 0.97 (m, 3H), 0.91 – 0.82 (m, 10H), 0.81 – 0.75 (m, 1H).

^{13}C{^{1}H}-APT NMR (101 MHz, $CDCl_3$, HSQC) δ 143.5 ($C_{q\text{-}arom}SO_2$), 139.2 ($C_{q\text{-}arom}CH_3$), 129.4 (CH_{arom}), 127.0 (CH_{arom}), 68.5 (CHOH), 58.0, 56.2, 56.1, 50.2 (CHN), 49.0, 42.3, 39.9, 39.6, 36.8, 36.2, 36.2, 35.8, 34.7, 30.7, 30.1, 29.8, 28.2, 28.1, 24.2, 23.9, 22.9, 22.7, 22.2, 21.7 (CH_3Ph), 20.6, 18.7, 11.8.

HRMS: $[M + H]^+$ calcd. for $C_{34}H_{54}NO_3S^+$ 556.3819; found 556.3819.

(E)-5-(3,3-dimethyl-1-tosylaziridin-2-yl)-3-methylpent-2-en-1-yl acetate (4u)

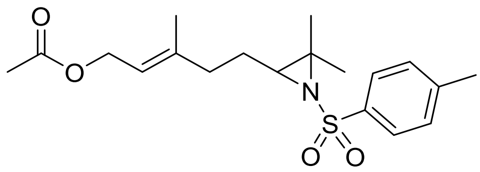

Prepared by **GP1** using 3 mol% 4CzPN. Crude purified by flash column chromatography (diethyl ether/pentane; 1.0:9.0 → 1.0:2.3) to afford **4u** as a colorless oil with a geranyl-related impurity that was challenging to remove. The addition of CH_2Br_2 as internal standard allowed for accurate quantification of the yield of **4u** (38 mg, 0.10 mmol, 52%).

^{1}H NMR (500 MHz, $CDCl_3$, H–H COSY, HSQC) δ 7.85 – 7.80 (m, 2H, Ts), 7.33 – 7.29 (m, 2H, Ts), 5.22 (tq, J = 7.1, 1.3 Hz, 1H, CH alkene), 4.54 (d, J = 7.1 Hz, 2H, CH_2OAc), 2.81 (dd, J = 7.6, 5.8 Hz, 1H, CHN), 2.43 (s, 3H, PhCH_3), 2.05 (s, 3H, CH_3 OAc), 2.01 – 1.89 (m, 1H, $C_{q\text{-alkene}}CH_2$), 1.90 – 1.76 (m, 1H, $C_{q\text{-alkene}}CH_2$), 1.70 (s, 3H, CH_3), 1.63 – 1.55 (m, 4H, NCHCH_2 + CH_3), 1.49 – 1.42 (m, 1H, NCHCH_2), 1.28 (s, 3H, CH_3).

^{13}C{^{1}H}-APT NMR (126 MHz, $CDCl_3$, HSQC) δ 171.2 (C_q=O), 143.8 ($C_{q\text{-arom}}SO_2$), 140.9 ($C_{q\text{-alkene}}$), 138.5 ($C_{q\text{-arom}}$$CH_3$), 129.5 ($CH_{arom}$), 127.5 ($CH_{arom}$), 119.1 (CH alkene), 61.3 (CH_2 OAc), 52.3 (CHN), 52.0 (C_qN), 37.0 $C_{q\text{-alkene}}$$CH_2$), 26.2 (NCH$CH_2$), 21.7 ($CH_3$Ph), 21.4 ($CH_3$), 21.3 ($CH_3$), 16.6 ($CH_3$).

Data in accordance with the literature.[12]

(1S,2S)-2-azido-3-((tert-butyldiphenylsilyl)oxy)-1-(1-tosyl-3-tridecylaziridin-2-yl)propan-1-ol (4v)

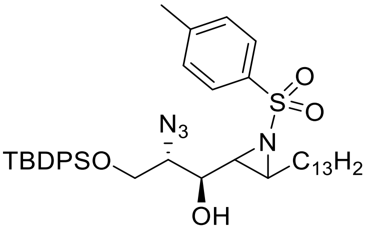

Prepared by **GP1** using 5 mol% 4CzPN. Crude purified by flash column chromatography (diethyl ether/pentane; 0.0:1.0 → 1.0:4.0) to afford mixtures of diastereomers **4v** as colorless oils (48 mg, 0.10 mmol, 51%). This reaction potentially yields 4 diastereomeric products. It was challenging to determine the absolute stereochemistry from the product mixtures due to overlapping signals in the ^{1}H NMR spectra. Two major diastereomers were formed of which the characterization data can be found below.

First product eluting from the column (one diastereomer):

^{1}H NMR (400 MHz, $CDCl_3$, H–H COSY, HSQC) δ 7.89 – 7.81 (m, 2H, Ts), 7.71 – 7.62 (m, 4H, Ph TBDPS), 7.49 – 7.35 (m, 6H, Ph TBDPS), 7.36 – 7.30 (m, 2H, Ts), 3.92 (dd, J = 10.8, 3.9 Hz, 1H, CH_2O), 3.84 (dt, J = 7.3, 3.8 Hz, 1H, CHOH), 3.79 (dd, J = 10.8, 6.8 Hz, 1H, CH_2O), 3.44 – 3.35 (m, 1H, CHN_3), 2.96 – 2.87 (m, 2H, CHN 2x), 2.42 (s, 3H, CH_3Ph), 1.72 – 1.62 (m, 1H, CH_2CHN), 1.58 – 1.50 (m, 1H, CH_2CHN), 1.35 – 1.14 (m, 22H, CH_2 alkyl chain), 1.08 (s, 9H, CH_3 *t*-Bu), 0.90 – 0.86 (m, 3H, CH_3 alkyl chain).

^{13}C{^{1}H}-APT NMR (101 MHz, $CDCl_3$, HSQC) δ 144.6 ($C_{q\text{-arom}}SO_2$), 137.0 ($C_{q\text{-arom}}$), 135.7 (CH_{arom} TBDPS), 135.7 (CH_{arom} TBDPS), 132.9 ($C_{q\text{-arom}}$), 132.8 ($C_{q\text{-arom}}$), 130.0 (CH_{arom}), 129.7 (CH_{arom}), 127.9

(CH_{arom}), 127.7 (CH_{arom}), 68.9 (CHOH), 65.9 (CHN_3), 64.4 (CH_2O), 52.2 (CHN), 47.8 (CHN), 32.0 (CH_2 alkyl chain), 29.8 (CH_2 alkyl chain), 29.8 (CH_2 alkyl chain), 29.8 (CH_2 alkyl chain), 29.6 (CH_2 alkyl chain), 29.5 (CH_2 alkyl chain), 29.5 (CH_2 alkyl chain), 29.2 (CH_2 alkyl chain), 27.3 (CH_2 alkyl chain), 26.8 (CH_3 *t*-Bu), 22.8 (CH_2 alkyl chain), 21.8 (*C*H_3Ph), 19.2 (C_q *t*-Bu), 14.3 (CH_3 alkyl chain).

HRMS: $[M + H – 2N]^+$ calcd. for $C_{41}H_{61}N_2O_4SSi^+$ 705.4116; found 705.4109.

Second product eluting from the column (mixture of two diastereomers, major one reported here):

^{1}H NMR (400 MHz, $CDCl_3$, H–H COSY, HSQC) δ 7.82 – 7.74 (m, 2H, Ts), 7.70 – 7.63 (m, 4H, Ph TBDPS), 7.48 – 7.36 (m, 6H, Ph TBDPS), 7.28 (d, *J* = 8.2 Hz, 2H, Ts), 3.87 – 3.80 (m, 3H, CH_2O + CHO), 3.43 (td, *J* = 6.3, 4.3 Hz, 1H, CHN_3), 3.01 (dd, *J* = 4.7, 3.3 Hz, 1H, OCHC*H*N), 2.88 (dt, *J* = 8.0, 5.2 Hz, 1H, CH_2C*H*N), 2.41 (s, 3H, CH_3Ph), 1.99 – 1.89 (m, 1H, C*H*$_2$CHN), 1.85 – 1.74 (m, 1H, C*H*$_2$CHN), 1.35 – 1.19 (m, 22H, CH_2 alkyl chain), 1.07 (s, 9H, *t*-Bu), 0.88 (t, *J* = 6.8 Hz, 3H, CH_3 alkyl chain).

^{13}C{^{1}H}-APT NMR (101 MHz, $CDCl_3$, HSQC) δ 144.5 ($C_{q\text{-}arom}SO_2$), 137.3 ($C_{q\text{-}arom}$), 135.7 (CH_{arom} TBDPS), 132.6 ($C_{q\text{-}arom}$), 130.1 (CH_{arom}), 129.8 (CH_{arom}), 128.0 (CH_{arom}), 127.5 (CH_{arom}), 67.6 (CHO), 65.1 (CHN_3), 64.1 (CH_2O), 48.9 (OCH*C*HN), 46.6 (CH_2*C*HN), 32.0 (CH_2 alkyl chain), 29.8 (CH_2 alkyl chain), 29.8 (CH_2 alkyl chain), 29.6 (CH_2 alkyl chain), 29.5 (CH_2 alkyl chain), 29.5 (CH_2 alkyl chain), 29.3 (CH_2 alkyl chain), 28.4 (CH_2 alkyl chain), 27.8 (CH_2 alkyl chain), 26.8 (CH_3 *t*-Bu), 22.8 (CH_2 alkyl chain), 21.7 (*C*H_3Ph), 19.2 (C_q *t*-Bu), 14.3 (CH_3 alkyl chain).

(1S,3S,5R,7R)-3,8,8-trimethyl-4-tosyl-4-azatricyclo[5.1.0.03,5]octane (4x)

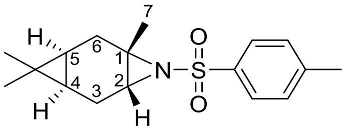

Prepared by **GP1** using 5 mol% 4CzPN. Crude purified by flash column chromatography (diethyl ether/pentane; 0.0:1.0 → 1.0:4.0) to afford **4x** as a colorless solid (38 mg, 0.13 mmol, 63%).

^{1}H NMR (400 MHz, $CDCl_3$, H–H COSY, HSQC) δ 7.86 – 7.79 (m, 2H, Ts), 7.33 – 7.26 (m, 2H, Ts), 2.93 (dd, *J* = 2.8, 2.0 Hz, 1H, H-2), 2.43 (s, 3H, C*H*$_3$Ph), 2.28 (dd, *J* = 15.9, 9.4 Hz, 1H, H-6), 2.02 (ddd, *J* = 16.0, 9.5, 2.0 Hz, 1H, H-3), 1.65 (s, 3H, CH_3-7), 1.45 (dt, *J* = 16.0, 3.0 Hz, 1H, H-3), 1.26 (dd, *J* = 16.0, 3.2 Hz, 1H, H-6), 0.97 (s, 3H, CH_3), 0.69 (s, 3H, CH_3), 0.52 (td, *J* = 9.3, 3.2 Hz, 1H, H-5), 0.33 (td, *J* = 9.4, 3.2 Hz, 1H, H-4).

^{13}C{^{1}H}-APT NMR (101 MHz, $CDCl_3$, HSQC) δ 143.4 ($C_{q\text{-}arom}SO_2$), 139.1 (*C*$_{q\text{-}arom}$$CH_3$), 129.5 ($CH_{arom}$), 127.0 ($CH_{arom}$), 48.7 (C-1), 46.0 (C-2), 27.7 (CH_3), 25.3 (C-6), 21.7 (CH_3Ph), 19.6 (CH_3), 17.7 (C-3), 16.5 (C_q, cyclopropyl), 16.2 (C-5), 15.2 (CH_3), 13.7 (C-4).

HRMS: $[M + H]^+$ calcd. for $C_{17}H_{24}NO_2S^+$ 306.1522; found 306.1522.

(1R,3R,6S)-3-isopropyl-6-methyl-7-tosyl-7-azabicyclo[4.1.0]heptan-3-ol (4y)

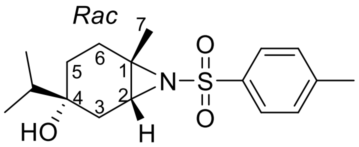

Prepared by **GP1** using 5 mol% 4CzPN. Crude purified by flash column chromatography (diethyl ether/pentane; 1.0:9.0 → 4.0:1.0) to afford **4y** as a colorless oil (42 mg, 0.13 mmol, 65%).

^{1}H NMR (500 MHz, $CDCl_3$, H−H COSY, H−H NOESY, HSQC) δ 7.81 – 7.77 (m, 2H, Ts), 7.34 – 7.30 (m, 2H, Ts), 3.26 (dd, *J* = 3.4, 1.8 Hz, 1H, H-2), 3.10 (s, 1H, OH), 2.43 (s, 3H, PhC*H*$_3$), 2.24 (ddd, *J* = 15.5, 12.1, 6.3 Hz, 1H, H-5/H-6), 1.82 (ddd, *J* = 15.5, 6.7, 2.8 Hz, 1H, H-5/H-6), 1.77 (s, 3H, H-7) 1.75 (d, *J* = 3.5 Hz, 1H, H-3), 1.68 (dt, *J* = 15.1, 2.0 Hz, 1H, H-3), 1.54 – 1.48 (m, 2H, CH *i*-Pr + H-5/H-6), 1.34 – 1.25 (m, 1H, H-5/H-6), 0.83 (d, *J* = 1.2 Hz, 3H, CH_3 *i*-Pr), 0.82 (d, *J* = 1.2 Hz, 3H, CH_3 *i*-Pr).

^{13}C{^{1}H}-APT NMR (126 MHz, $CDCl_3$, HSQC) δ 144.3 ($C_{q\text{-}arom}SO_2$), 138.1 ($C_{q\text{-}arom}CH_3$), 130.0 (CH_{arom}), 127.1 (CH_{arom}), 71.7 (C-4), 51.6 (C-1), 49.1 (C-2), 37.0 (CH *i*-Pr), 31.1 (C-3), 29.2 (C-5/C-6), 28.7 (C-5/C-6), 21.8 (Ph*C*H$_3$), 20.9 (C-7), 16.9 (CH_3 *i*-Pr), 16.5 (CH_3 *i*-Pr).

HRMS: [M + H]$^+$ calcd. for $C_{17}H_{26}NO_3S^+$ 324.1628; found 324.1629, [M + Na]$^+$ calc. for $C_{17}H_{25}NNaO_3S^+$ 346.1447; found 346.1449.

Galactose-cyclphellitol aziridine (4z)

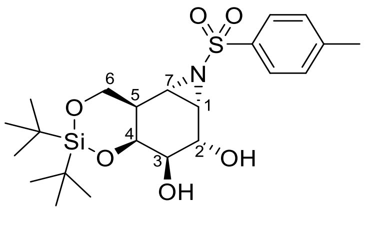

Prepared by **GP1** using 5 mol% 4CzPN. Crude purified by flash column chromatography (ethyl acetate/pentane; 1.0:2.3 → 1.0:1.0) to afford **4z** as a colorless solid (48 mg, 0.10 mmol, 51%).

^{1}H NMR (400 MHz, $CDCl_3$, H−H COSY, HSQC) δ 7.87 – 7.80 (m, 2H, Ts), 7.34 (d, *J* = 7.7 Hz, 2H, Ts), 4.41 – 4.33 (m, 2H, H-4, H-6), 4.19 – 4.09 (m, 2H, H-2, H-6), 3.37 – 3.29 (m, 2H, H-1, H-3), 3.16 (dt, *J* = 7.0, 1.3 Hz, 1H, H-7), 2.54 (br s, 1H, OH), 2.44 (s, 3H, C*H*$_3$Ph), 2.28 (br s, 1H, OH), 2.00 – 1.97 (m, 1H, H-5), 1.05 (s, 9H, C*H*$_3$ *t*-Bu), 1.00 (s, 9H, C*H*$_3$ *t*-Bu).

^{13}C{^{1}H}-APT NMR (101 MHz, $CDCl_3$, HSQC) δ 145.0 ($C_{q\text{-}arom}SO_2$), 134.6 ($C_{q\text{-}arom}CH_3$), 129.9 (CH_{arom}), 128.1 (CH_{arom}), 75.5 (C-4), 74.1 (C-3), 68.3 (C-3), 66.8 (C-6), 46.2 (C-1), 43.7 (C-7), 39.6 (C-5), 28.2 (*C*H$_3$ *t*-Bu), 27.1 (*C*H$_3$ *t*-Bu), 23.4 (C_q *t*-Bu), 21.8 *C*H$_3$Ph, 20.4 (C_q *t*-Bu).

HRMS: [M + H]$^+$ calcd. for $C_{22}H_{36}NO_6SSi^+$ 470.2027; found 470.2027.

6. Scale-up reaction *(cf. Table 2)*

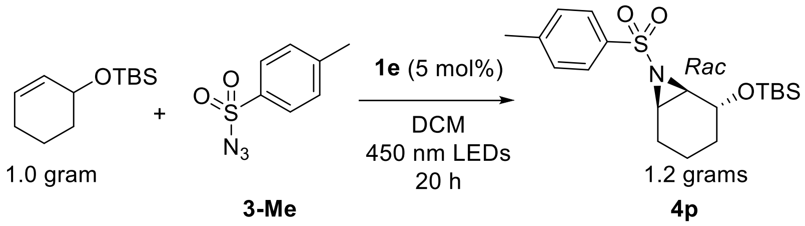

In a similar fashion to GP1, a Schlenk tube was charged with **1e** (0.19 g, 0.24 mmol, 0.050 eq.), 24 mL DCM, **3-Me** (3.6 mL, 24 mmol, 5.0 eq.) and *tert*-butyl(cyclohex-2-en-1-yloxy)dimethylsilane (1.0 g, 4.7 mmol, 1.0 eq.). The mixture was deoxygenated by three freeze-pump-thaw cycles, ending on dinitrogen. Using standard syringe and needle techniques, the mixture was transferred to an elongated reaction tube under dinitrogen containing a stir bar. The tube was irradiated with two 30 W blue LED lights (λ_{max} = 450 nm, HCK1012-01-002, EvoluChem™) and maintained around room temperature with a fan (**CAUTION:** A reaction of this scale will evolve large volumes of dinitrogen; in our case a pressure relief valve on the Schlenk line allowed for safe dinitrogen release). The reaction mixture was concentrated *in vacuo* after 20 h and the crude product was analyzed by ^{1}H NMR spectroscopy. The aziridine was isolated by flash column chromatography (1.2 g, 3.2 mmol, 68%) in the same manner as described in section 5 for compound **4p** and its spectral data was in accordance with the spectral data reported in section 5.

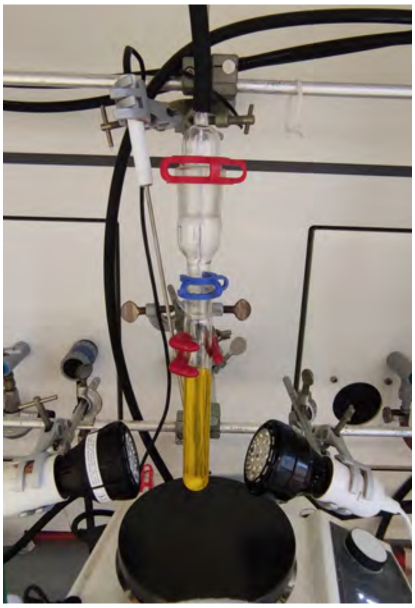

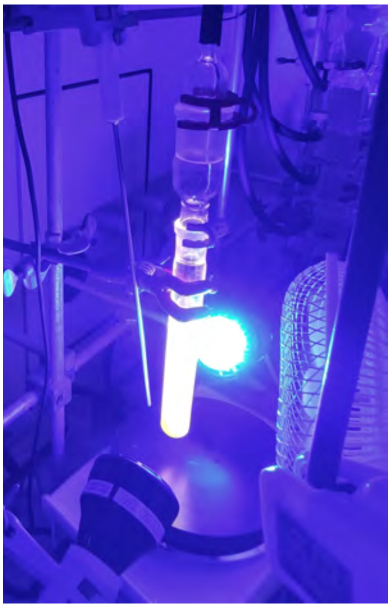

Figure S4. Setup used for gram-scale aziridination. **Left:** The reaction mixture prior to shining light. The tube on top is connected to the Schlenk line including the pressure relief valve. **Right:** Reaction right after switching on the light, here the position of the fan can be seen.

7. Hammett Plots *(cf. Figure 2a)*

GP2-General procedure for the aziridination reaction for constructing the Hammett plots

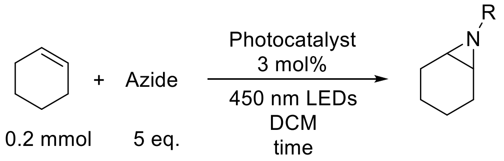

A 1 mL GC vial was charged with a stir bar and 1.0 mmol of azide. The vial was entered in a Schlenk tube and the tube was deoxygenated by subjection to three vacuum and dinitrogen cycles, ending on dinitrogen. In a separate Schlenk tube, a stock solution was made containing the indicated amount of photocatalyst and dichloromethane (table S2). This stock solution was deoxygenated by three freeze-pump-thaw cycles, ending on dinitrogen. The appropriate amount of stock solution was then added to the vial with azide. Cyclohexene (0.20 mmol), freshly passed over alumina, was quickly added, and the vial was closed with a screw cap. The vial was introduced in the photoreactor and irradiated with blue LED lights for the indicated time. After the reaction, the mixture was transferred to a round-bottom flask and concentrated *in vacuo*. The crude was dissolved in $CDCl_3$ in the presence of a known amount (~ 4.00 mg) of 1,3,5-trimethoxybenzene as internal standard to determine the yield with ^{1}H NMR spectroscopy.

It was found in our screening that due to the lower solubility of **1e** and **1f**, these reactions gave irreproducible results when too little solvent was used. By performing these reactions at higher dilution, the reactions were reproducible. It was considered that dilution of reaction mixtures could affect the aziridine yields and thereby the Hammett plots. However, we found that the reactions using either **1d** or **1e** gave the same yields at 2.0 mM, 6.0 mM or 12 mM concentration. At 2.0 mM the reactions became observably slower but extending reaction times to 23 h gave the same yields. Hence the reported reactions with **1f** required extended reaction times. Thus, the differences in yields are purely a consequence of the matching of the azide and photosensitizer, not of dilution.

Table S2. Raw data used for the Hammett plots.

Azide *para* substituent	Hammett substituent constant (σ)	1d (4CzIPN)[a]		1e (4CzPN)[b]		1f (4CzTPN)[c]	
		Yield 1	Yield 2	Yield 1	Yield 2	Yield 1	Yield 2
***i*-PrO**	-0.45	49	48	53	57	23	22
**OMe**	-0.27	52	52	57	61	22	22
**Me**	-0.17	46	46	64	60	30	30
**Cl**	0.23	32	32	51	56	52	57
**OCF_3**	0.35	27	27	51	51	52	47
**CF_3**	0.54	19	21	34	33	31	33
**CN**	0.66	16	16	31	25	19	20
**NO_2**	0.78	0	0	0	0	0	0

[a] Reactions performed according to **GP2** using 3 mol % **1d** in 0.5 mL DCM for 16 h. [b] Reactions performed according to **GP2** using 3 mol % **1e** in 1.0 mL DCM for 16 h. [c] Reactions performed according to **GP2** using 3 mol % **1f** in 3.0 mL DCM for 23 h.

8. Stern-Volmer luminescence quenching studies *(cf. Figure 2b)*

Luminescence spectra were recorded on a HORIBA Aqualog® spectrofluorometer. An excitation wavelength of 450 nm was used and the emission was measured between 211 nm and 620 nm. The emission intensity at the emission maximum of **1d** at 548 nm was used to construct the Stern-Volmer plots. All measurements were performed in the same quartz cuvette from Hellma (1 cm x 1 cm) fitted with a rubber septum containing 2 mL solution.

In a glovebox, a 60 µM solution of **1d**, a 0.10 M solution of **3-R**, a 0.20 M solution of **3-R**, a 0.40 M solution of **3-R** and a 0.80 M solution of **3-R**, in DCM were prepared. To prepare a sample for measurement, a 1.0 mL sample of the **1d** solution was taken and 1.0 mL of the desired **3-R** solution (or DCM in the case of the measurement in absence of quencher) was added to the cuvette. The cuvette was closed with a rubber septum and transported in a closed flask under inert atmosphere to the spectrofluorometer. The cuvette was taken out of the flask and the emission spectrum was recorded as quickly as possible to minimize interference of molecular oxygen.

Cognizant of the lifetime of **1d** in DCM (τ_0 = 24.6 ns)[14] we could calculate the bimolecular quenching constants (k_q) from the slope of the lines of the Stern-Volmer plots (K_{SV}) according to equation 1.

Equation 1: $K_{SV} = k_q \tau_0$

9. Cyclic Voltammetry *(cf. Figure 2c)*

Cyclic voltammetry (CV) was performed using an Autolab Pgstart10 potentiostat controlled by GPES4 software connected to a three-electrode cell containing a 0.07 cm^2 glassy carbon working electrode, Pt wire auxiliary electrode, a Ag/AgCl reference electrode (sat. aq. KCl). Prior to starting each measurement, the working electrode was polished manually in a figure-eight motion using MicroPolish™ alumina 0.3 µm and some Milli-Q® water. Then, the working electrode was polished in the same manner with MicroPolish™ alumina 0.05 µm and some Milli-Q® water. The working electrode was then sonicated in Milli-Q® water and washed with MeCN. All solutions in the electrochemical cell were degassed by bubbling with argon. A scan rate of 50 mV s^{-1} was used and the experiments were performed at room temperature under argon. First, a blank (0.1 M tetrabutylammonium hexafluorophosphate in MeCN) was measured to ensure successful polishing of the electrode. All other CV experiments were performed with a 1 mM concentration of the azide **3-R** in 0.1 M tetrabutylammonium hexafluorophosphate in MeCN. After the measurement, ferrocene was added as internal standard and the spectrum was referenced against the Fc^+/Fc redox couple. Addition of 0.380 V allowed referencing against SCE.[15] The reported spectra were first scanned in anodic direction starting from -0.380 V vs SCE.

Comments on the data

We considered that the strongly reducing excited states of our photosensitizers might result in direct one-electron reduction of the azides. This might either be a pathway towards catalyst inactivation, or the resulting nitrene radical anion might be an effective aziridinating agent. This potential mechanism is outlined in Figure S5. After excitation of the photocatalyst, a single electron transfer from PC* to the azide would yield $PC^{\cdot+}$ and the azide radical anion. This species might lose dinitrogen similar to Liu's azide reduction.[16] The outcome of this process is a nitrene radical anion akin to Koenigs findings.[17] The sulfonyl-substituted radical anion should be capable of reacting with an alkene to form the aziridine and render the process catalytic by reducing $PC^{\cdot+}$ in the process.

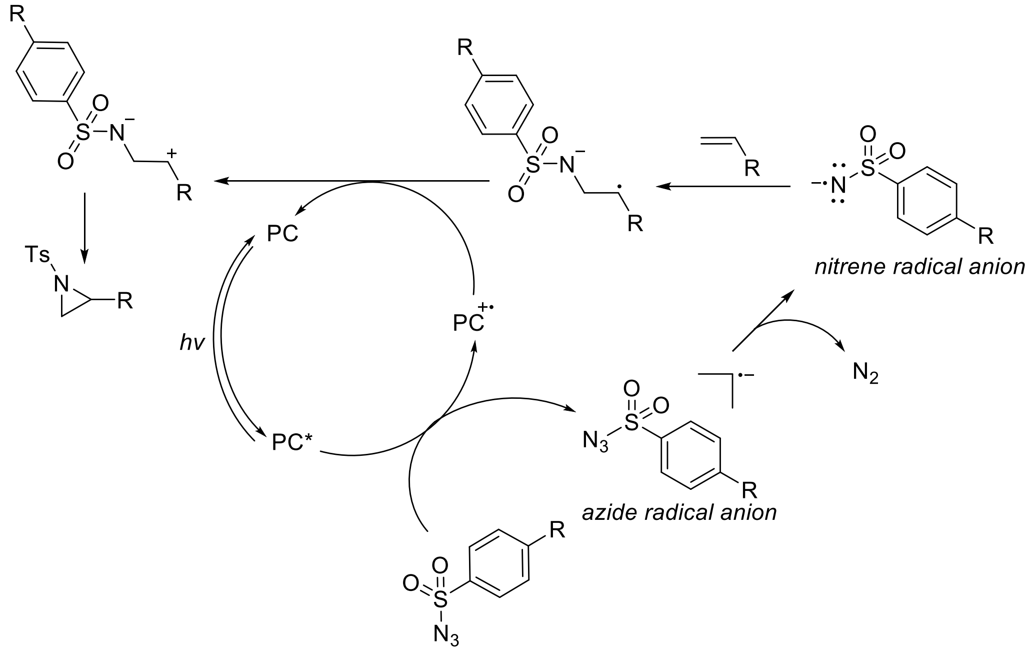

Figure S5. Possible aziridination mechanism initiated by direct one electron reduction of the phenylsulfonyl azide.

From the cyclic voltammograms (Figure 2c) we obtained estimated $E_{1/2}$ values vs SCE for the reduction event of the three azides: -1.10 V (**3-CF₃**), -1.19 V (**3-Me**) and -1.11 V (**3-*i*PrO**). The excited-state

oxidation potentials of cyanoarenes **1d-f** are reducing enough to transfer an electron as they are more reducing than the onset potential and quite close to the estimated $E_{1/2}$ values of the azides (largest difference is E_{ox}*(**1d**) = -0.99 V vs SCE and estimated $E_{1/2}$ (**3-Me**) = -1.19 V vs SCE. The difference is 0.20 V, which corresponds to 4.6 kcal mol^{-1}.

10. Triplet nitrene trapping *(cf. Figure 2d)*

The reaction outlined in Figure 2d was performed as in **GP1**. The crude ^{1}H NMR spectrum showed exclusive formation of the C(sp^{3})-H amination product (Figure S6). The ^{1}H NMR spectrum is in accordance with the literature.[18] Similarly, for **3-CF$_3$** and **3-*i*PrO** exclusively C(sp^{3})-H amination product was observed.

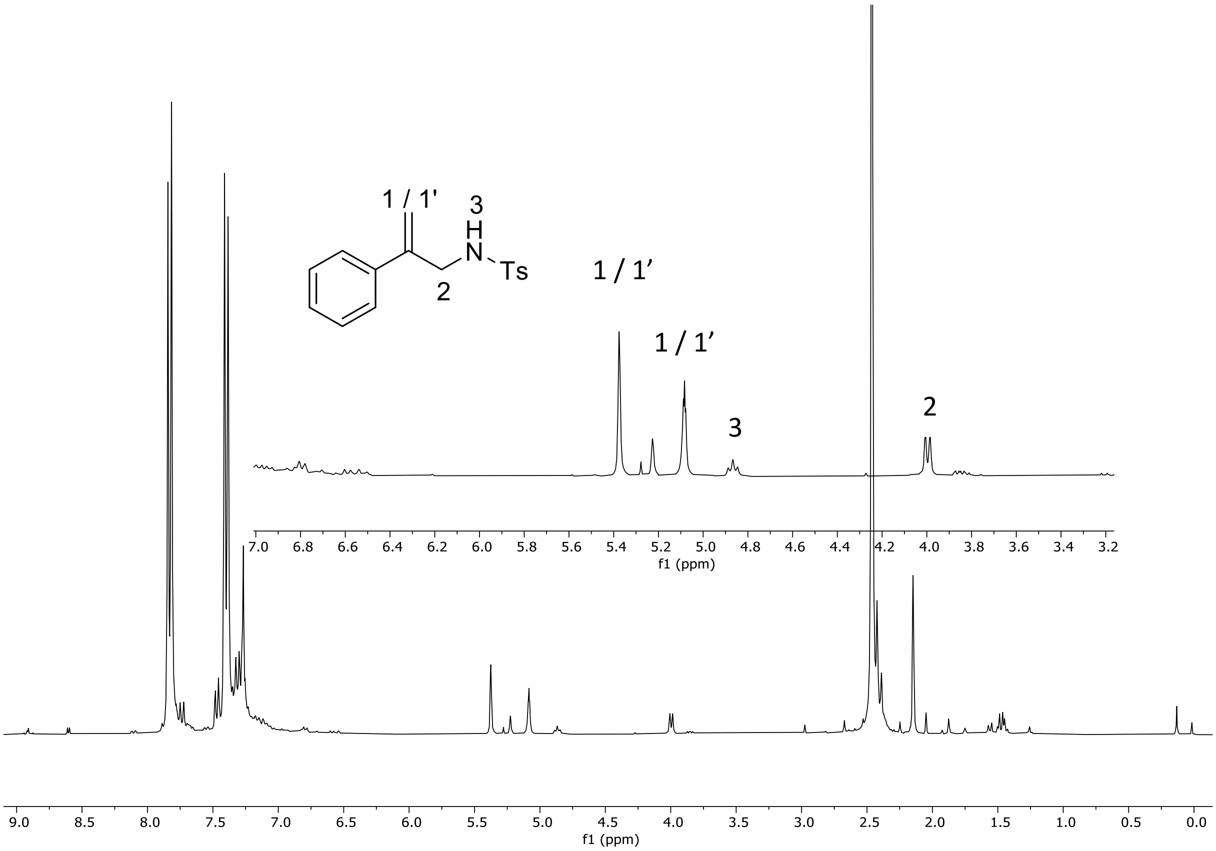

Figure S6. Crude ^{1}H NMR spectrum of **GP1** performed on α-methylstyrene showing exclusive C-H amination product.

11. UV-Vis experiments *(cf. Figure 2e)*

UV-Visible experiments were carried out with a Cary 60 UV-Vis Spectrometer from Agilent in a quartz cuvette from Hellma (1 cm x 1 cm). To monitor the reaction as in Figure 2e, the reaction was commenced as per **GP1**. At the indicated time points, a 10 µL sample was taken and diluted in 3 mL DCM in the quartz cuvette prior to the UV-Vis measurement. The UV-Vis experiments of the photosensitizer and the azides (Figures S7-S10) were measured at the same concentrations as for Figure 2e. Upon addition of azides to the photosensitizer no spectral changes were observed.

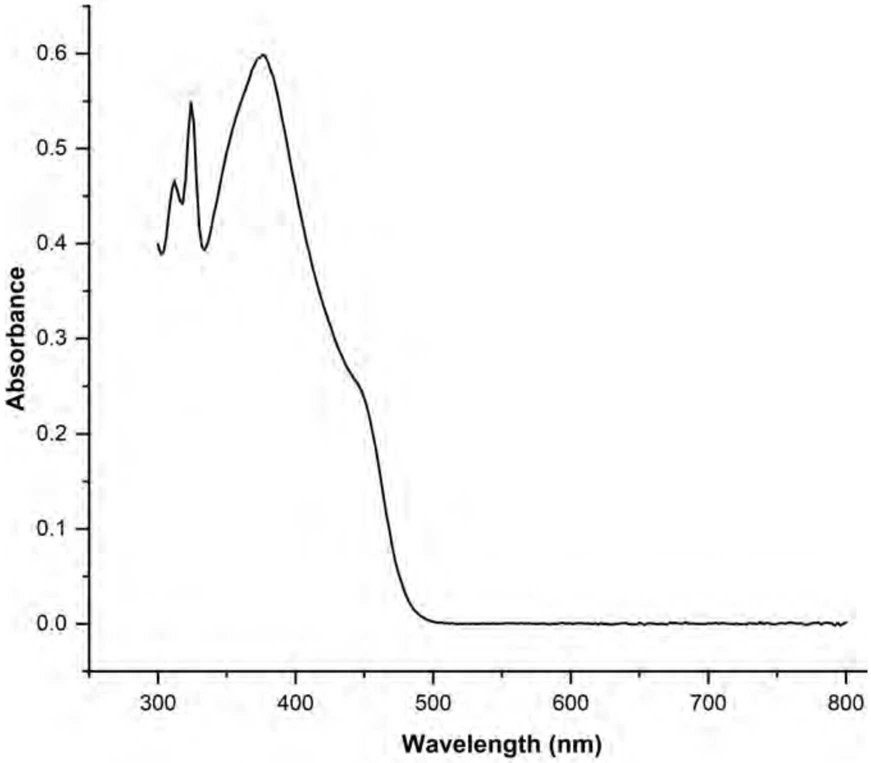

Figure S7. UV-Vis absorption spectrum of **1d** in DCM.

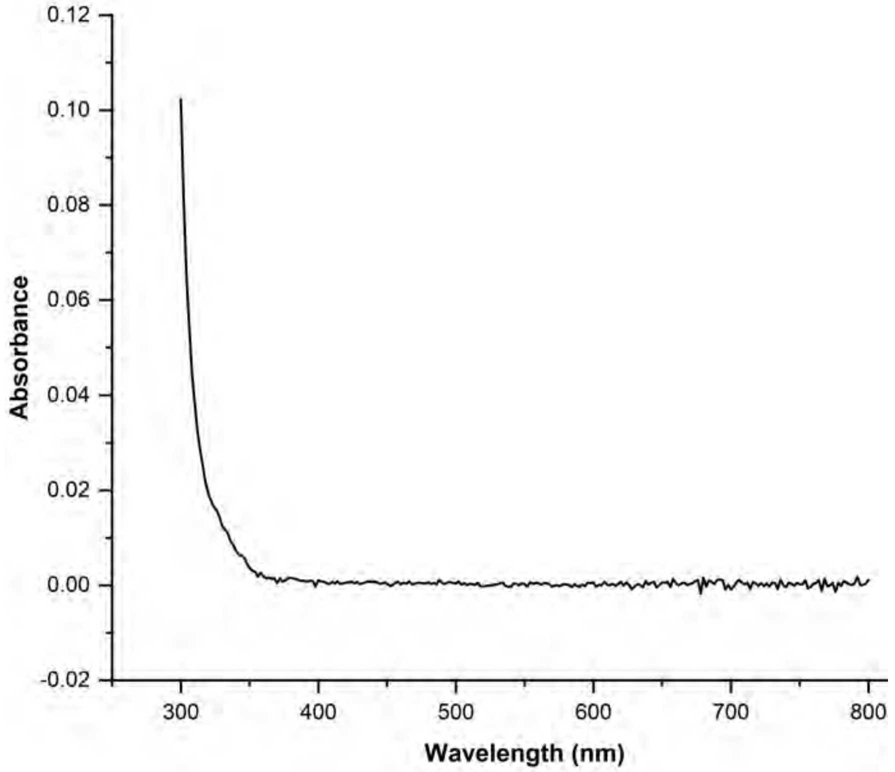

Figure S8. UV-Vis absorption spectrum of **3-CF$_3$** in DCM.

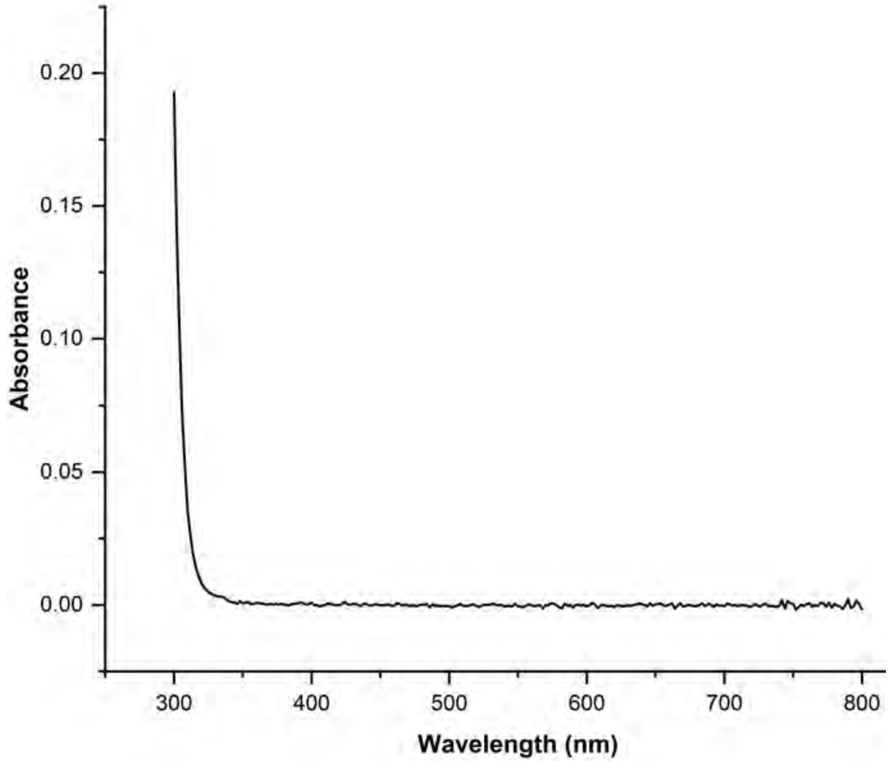

Figure S9. UV-Vis absorption spectrum of **3-Cl** in DCM.

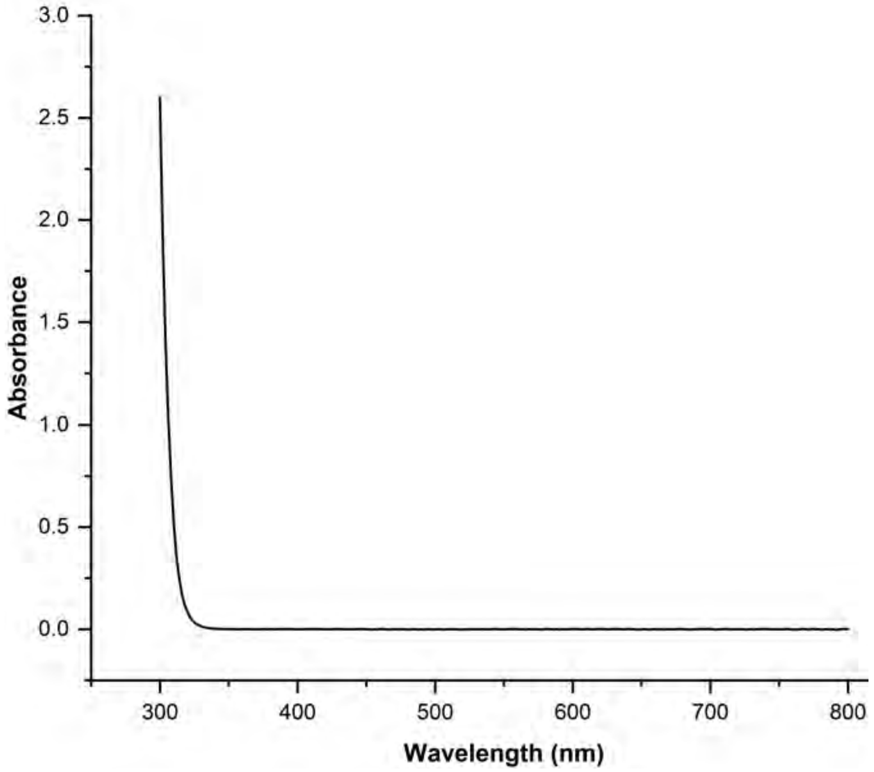

Figure S10. UV-Vis absorption spectrum of **3-*i*PrO** in DCM.

12. Reaction kinetics by ¹H NMR spectroscopy *(cf. Figures 2f and 2g)*

Procedure used for all NMR experiments:

To a J Young NMR tube, **1d** and the azide (**3-R**) were added. The NMR tube was introduced in the glovebox and 0.50 mL CD_2Cl_2 was added. The NMR tube was closed and removed from the glovebox. Then cyclohexene (**2**), passed over a short alumina pad, and 10 µL CH_2Br_2 were added. The NMR tube was closed again and shaken well to ensure homogeneity. The NMR tube was then placed in the photoreactor and irradiated. The reaction was monitored periodically by ¹H NMR spectroscopy using CH_2Br_2 as internal standard.

A zoom of the NMR spectra of the reaction involving **3-Cl** is outlined below in figure S11. These stacked NMR spectra clearly indicate the transformation of **1d** into new species.

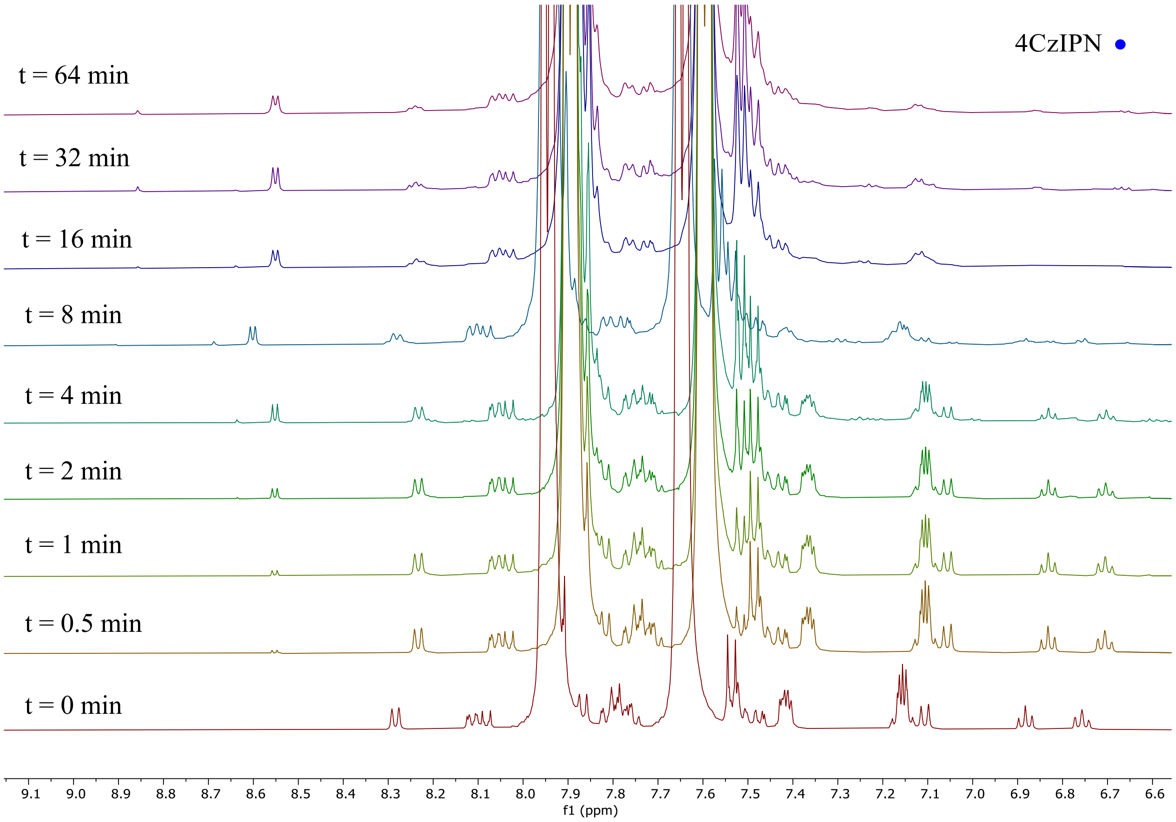

Figure S11. NMR reaction of 0.20 mmol **2**, 1.0 mmol **3-Cl** with 3.0 mol% **1d** in 0.50 mL CD_2Cl_2 followed over time with ¹H NMR spectroscopy. The indicated timepoints refer to cumulative irradiation times with 450 nm LEDs.

13. Limitations

For the substrates below **GP1** was applied but were unsuccessful for the desired transformation. In the case of the terminal alkyne no nitrene insertion nor cycloaddition products were observed. For 1,5-cyclooctadiene and 2-vinylpyridine some product was detected alongside a complex mixture of other products. In the case of the ceramide, the starting material was fully consumed, however no aziridine product could be detected. Both *trans*- and *cis*-stilbene provided traces of aziridine products however the reaction provided a low NMR yield. The origin of this may be competing energy transfer from **1e** to the stilbene substrates.[19]

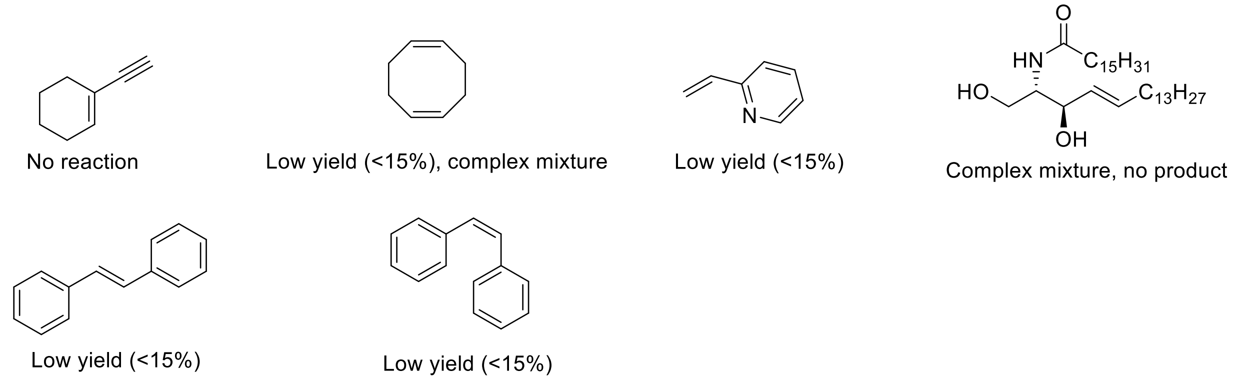

Figure S12. Limitations of the aziridination reaction. Reactions were analyzed by ^{1}H NMR and the yield was determined using CH_2Br_2 as internal standard.

14. References

(1) Speckmeier, E.; Fischer, T. G.; Zeitler, K. A Toolbox Approach To Construct Broadly Applicable Metal-Free Catalysts for Photoredox Chemistry: Deliberate Tuning of Redox Potentials and Importance of Halogens in Donor–Acceptor Cyanoarenes. *J. Am. Chem. Soc.* **2018**, *140*, 15353–15365.

(2) Kumar, P.; Jiang, T.; Li, S.; Zainul, O.; Laughlin, S. T. Caged Cyclopropenes for Controlling Bioorthogonal Reactivity. *Org. Biomol. Chem.* **2018**, *16*, 4081–4085.

(3) Guevel, A.-C.; Hart, D. J. Synthesis of Carbocycles via Intramolecular Conjugate Additions: Total Syntheses of Axane Sesquiterpenoids. *J. Org. Chem.* **1996**, *61*, 473–479.

(4) Trost, B. M.; Shen, H. C.; Horne, D. B.; Toste, F. D.; Steinmetz, B. G.; Koradin, C. Syntheses of Seven-Membered Rings: Ruthenium-Catalyzed Intramolecular [5+2] Cycloadditions. *Chem. – Eur. J.* **2005**, *11*, 2577–2590.

(5) Kim, S.; Lee, S.; Lee, T.; Ko, H.; Kim, D. Efficient Synthesis of D-Erythro-Sphingosine and d-Erythro-Azidosphingosine from d-Ribo-Phytosphingosine via a Cyclic Sulfate Intermediate. *J. Org. Chem.* **2006**, *71*, 8661–8664.

(6) Jiang, J.; Artola, M.; Beenakker, T. J. M.; Schröder, S. P.; Petracca, R.; de Boer, C.; Aerts, J. M. F. G.; van der Marel, G. A.; Codée, J. D. C.; Overkleeft, H. S. The Synthesis of Cyclophellitol-Aziridine and Its Configurational and Functional Isomers. *Eur. J. Org. Chem.* **2016**, *2016*, 3671–3678.

(7) Harrak, Y.; Barra, C. M.; Delgado, A.; Castaño, A. R.; Llebaria, A. Galacto-Configured Aminocyclitol Phytoceramides Are Potent in Vivo Invariant Natural Killer T Cell Stimulators. *J. Am. Chem. Soc.* **2011**, *133*, 12079–12084.

(8) Ng, W.-H.; Hu, R.-B.; Lam, Y.-P.; Yeung, Y.-Y. Zwitterion-Catalyzed Intermolecular Bromoesterifications. *Org. Lett.* **2020**, *22*, 5572–5576.

(9) Liu, M.-S.; Du, H.-W.; Cui, J.-F.; Shu, W. Intermolecular Metal-Free Cyclopropanation and Aziridination of Alkenes with XH2 (X=N, C) by Thianthrenation. *Angew. Chem. Int. Ed.* **2022**, *61*, e202209929.

(10) Kiyokawa, K.; Kosaka, T.; Minakata, S. Metal-Free Aziridination of Styrene Derivatives with Iminoiodinane Catalyzed by a Combination of Iodine and Ammonium Iodide. *Org. Lett.* **2013**, *15*, 4858–4861.

(11) Craig II, R. A.; O'Connor, N. R.; Goldberg, A. F. G.; Stoltz, B. M. Stereoselective Lewis Acid Mediated (3+2) Cycloadditions of N-H- and N-Sulfonylaziridines with Heterocumulenes. *Chem. – Eur. J.* **2014**, *20*, 4806–4813.

(12) Ren, Y.; Cheaib, K.; Jacquet, J.; Vezin, H.; Fensterbank, L.; Orio, M.; Blanchard, S.; Desage-El Murr, M. Copper-Catalyzed Aziridination with Redox-Active Ligands: Molecular Spin Catalysis. *Chem. – Eur. J.* **2018**, *24*, 5086–5090.

(13) Zhao, Q.; Yao, Q.-Y.; Zhang, Y.-J.; Xu, T.; Zhang, J.; Chen, X. Selective Cyclopropanation/Aziridination of Olefins Catalyzed by Bis(Pyrazolyl)Borate Cu(I) Complexes. *Eur. J. Org. Chem.* **2022**, *2022*, e202200790.

(14) Ishimatsu, R.; Matsunami, S.; Shizu, K.; Adachi, C.; Nakano, K.; Imato, T. Solvent Effect on Thermally Activated Delayed Fluorescence by 1,2,3,5-Tetrakis(Carbazol-9-Yl)-4,6-Dicyanobenzene. *J. Phys. Chem. A* **2013**, *117*, 5607–5612.

(15) Pavlishchuk, V. V.; Addison, A. W. Conversion Constants for Redox Potentials Measured versus Different Reference Electrodes in Acetonitrile Solutions at 25°C. *Inorganica Chim. Acta* **2000**, *298*, 97–102.

(16) Chen, Y.; Kamlet, A. S.; Steinman, J. B.; Liu, D. R. A Biomolecule-Compatible Visible-Light-Induced Azide Reduction from a DNA-Encoded Reaction-Discovery System. *Nat. Chem.* **2011**, *3*, 146–153.

(17) Guo, Y.; Pei, C.; Koenigs, R. M. A Combined Experimental and Theoretical Study on the Reactivity of Nitrenes and Nitrene Radical Anions. *Nat. Commun.* **2022**, *13*, 86.

(18) Kiyokawa, K.; Kojima, T.; Hishikawa, Y.; Minakata, S. Iodine-Catalyzed Decarboxylative Amidation of β,γ-Unsaturated Carboxylic Acids with Chloramine Salts Leading to Allylic Amides. *Chem. – Eur. J.* **2015**, *21*, 15548–15552.

(19) Lu, J.; Pattengale, B.; Liu, Q.; Yang, S.; Shi, W.; Li, S.; Huang, J.; Zhang, J. Donor–Acceptor Fluorophores for Energy-Transfer-Mediated Photocatalysis. *J. Am. Chem. Soc.* **2018**, *140*, 13719–13725.

15. NMR Spectra of new compounds and compounds isolated from catalytic reactions

(3-*i*PrO): ^{1}H NMR, 400 MHz in $CDCl_3$

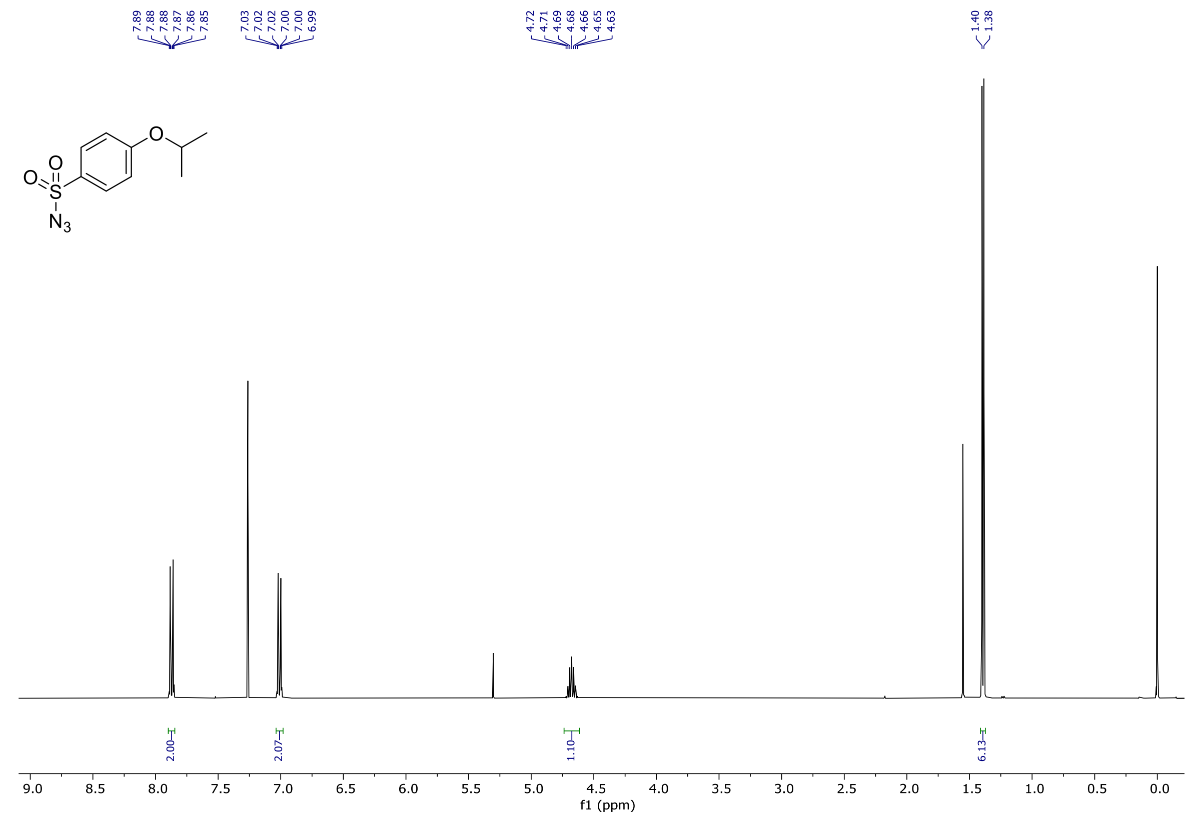

(3-*i*-PrO): ^{13}C{^{1}H}-APT NMR, 101 MHz in $CDCl_3$

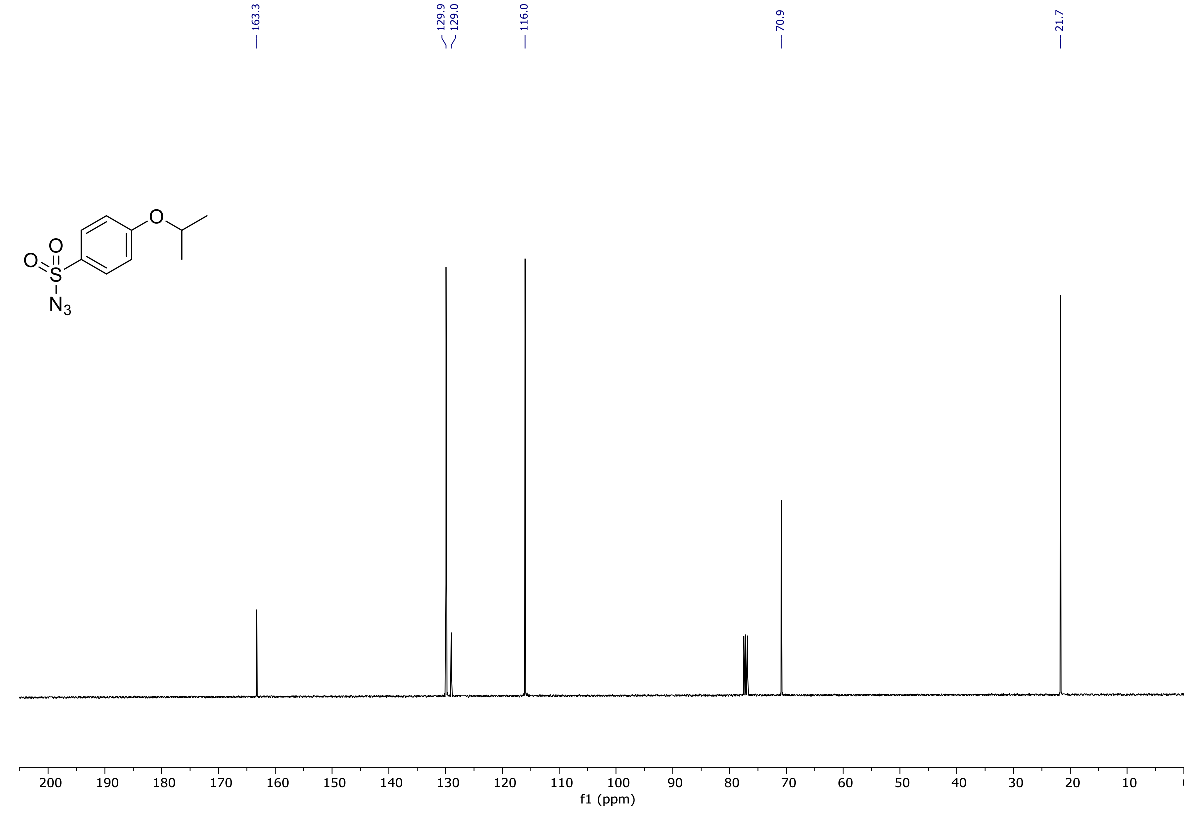

S3: ^{1}H NMR, 400 MHz in $CDCl_3$

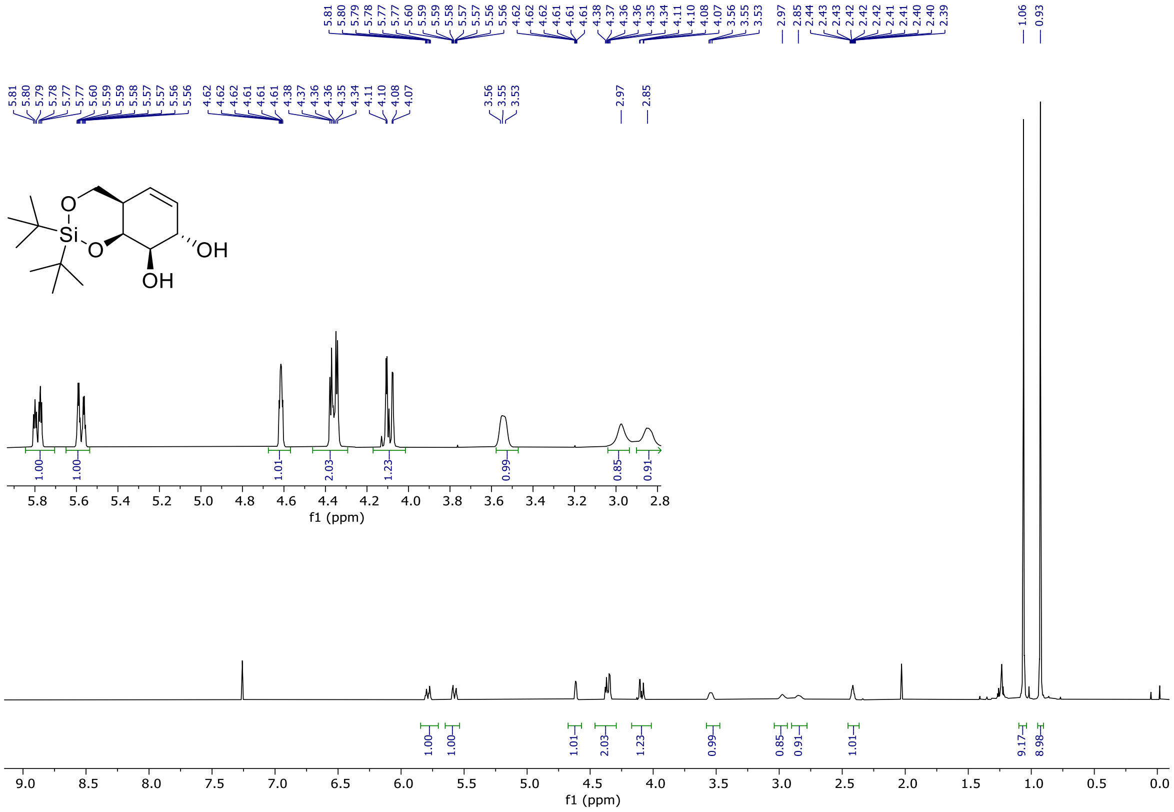

S3:^{13}C{^{1}H}-APT NMR, 101 MHz in $CDCl_3$

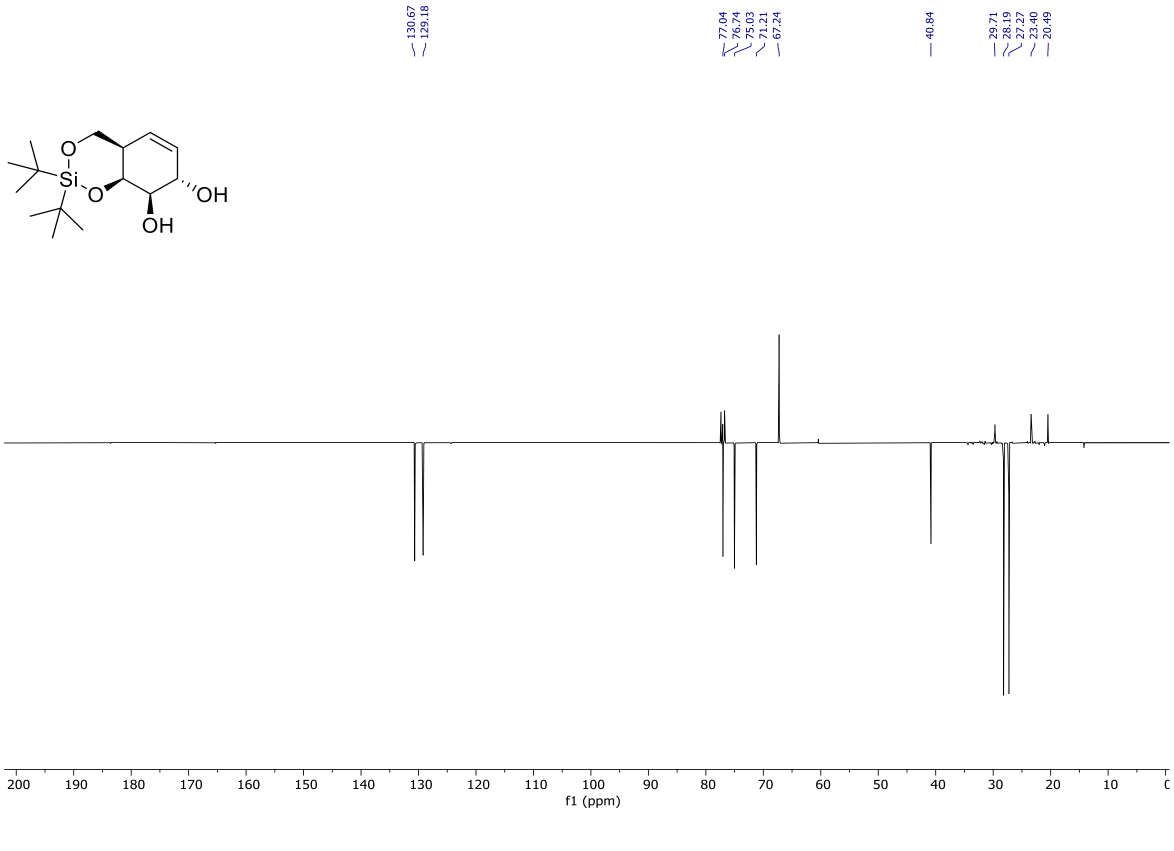

S3: ^{1}H-^{1}H COSY spectrum in $CDCl_3$

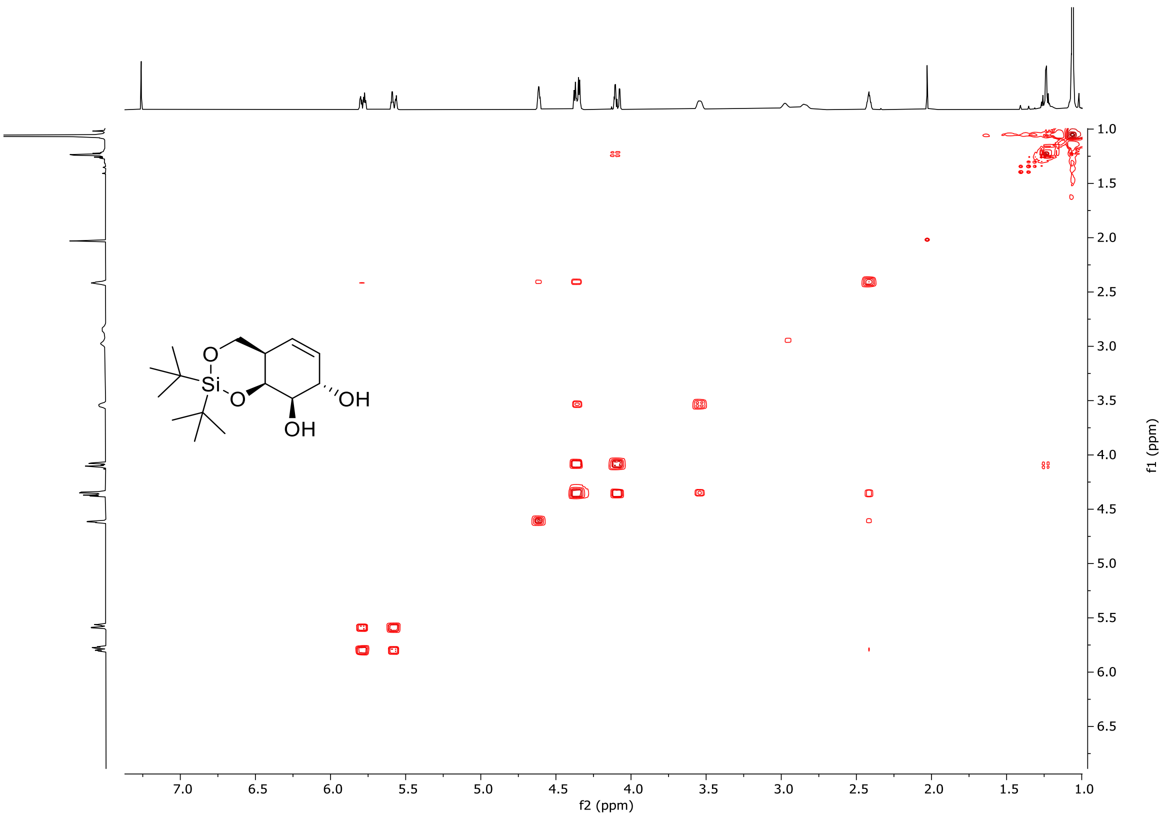

S3: ^{1}H-^{13}C HSQC spectrum in $CDCl_3$

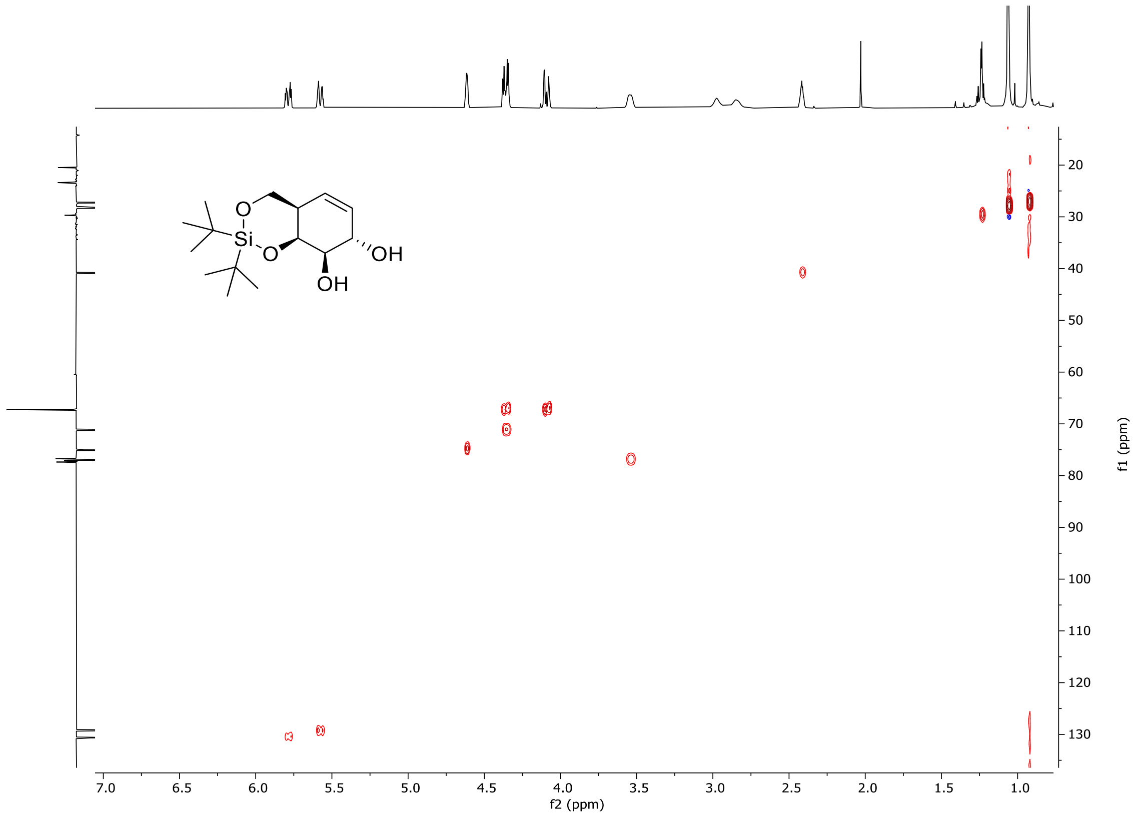

4a-Me:^{1}H NMR, 400 MHz in $CDCl_3$

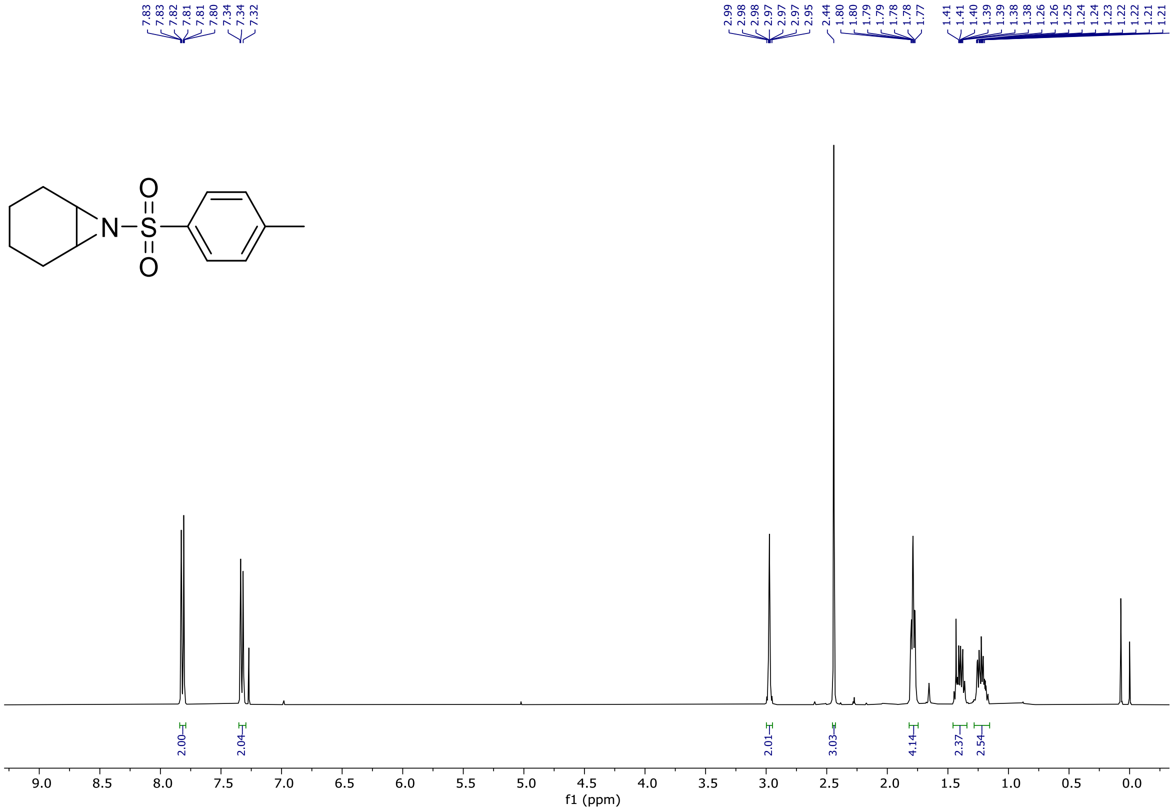

4a-Me:^{13}C{^{1}H}-APT NMR, 101 MHz in $CDCl_3$

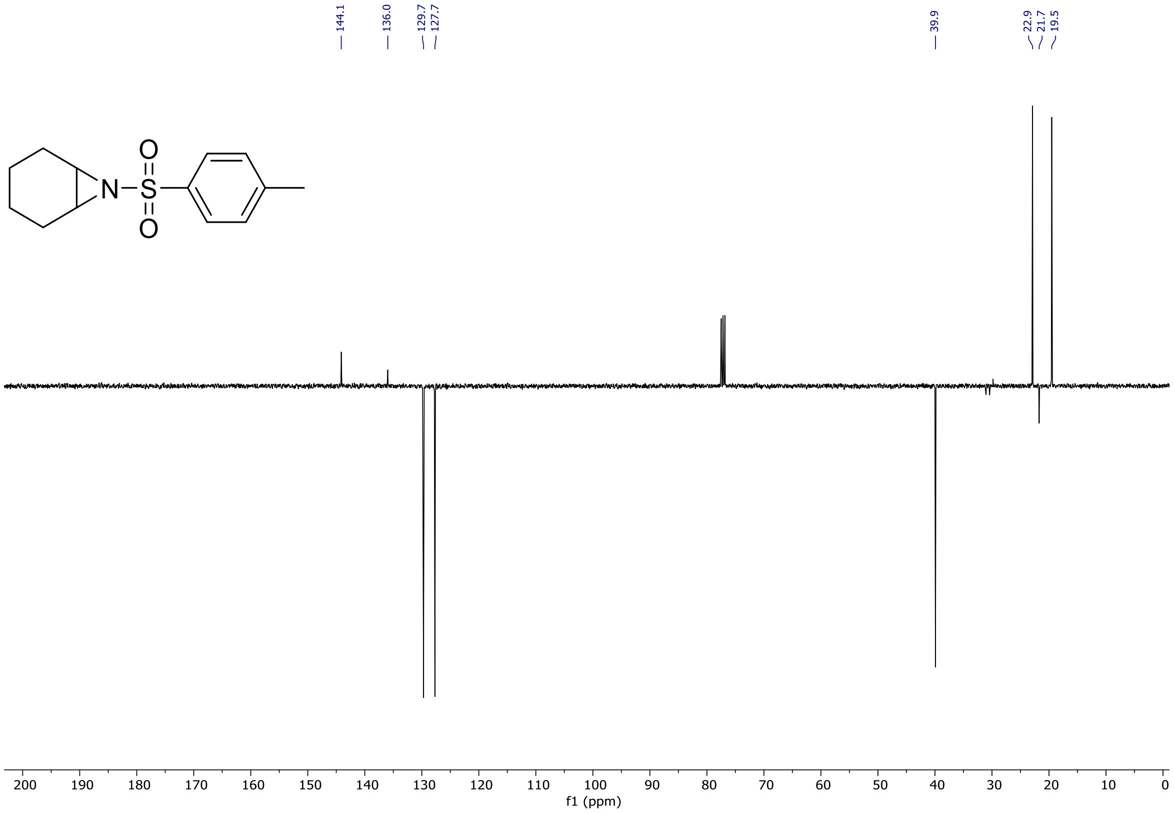

4b: ^{1}H NMR, 400 MHz in $CDCl_3$

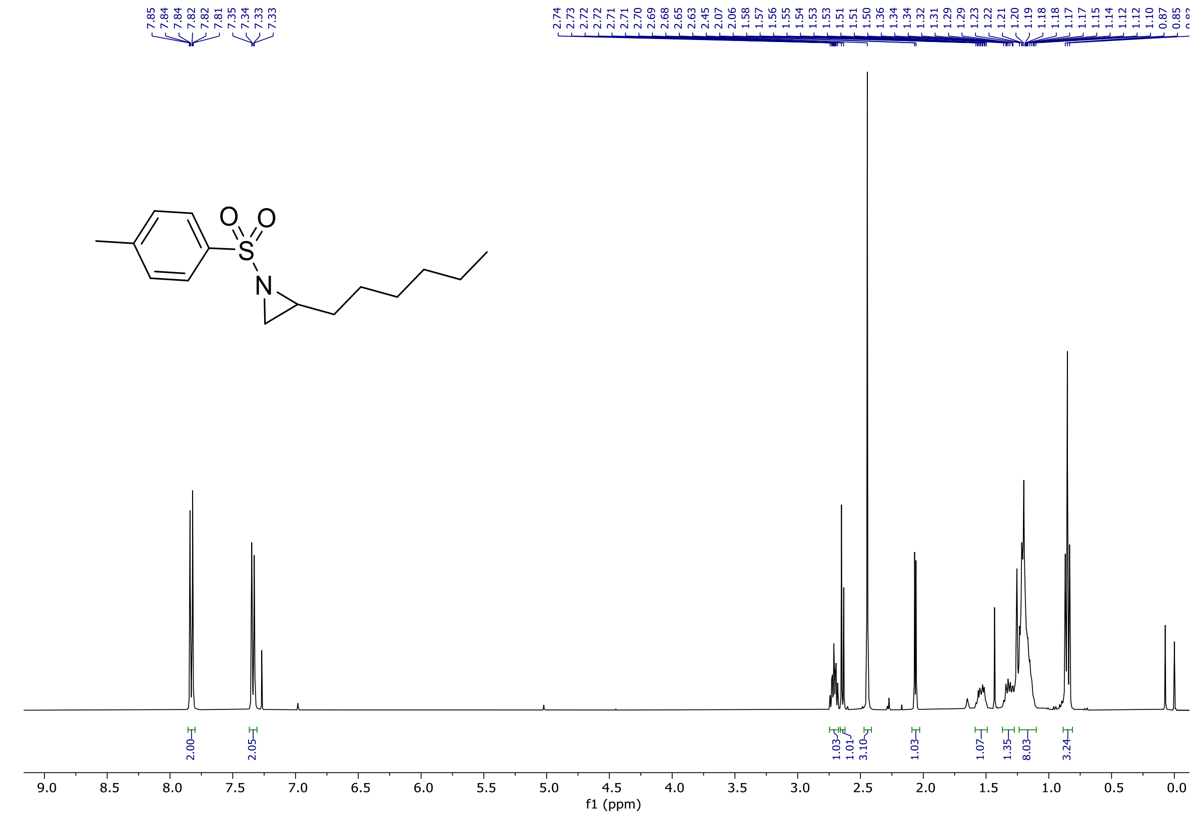

4b:^{13}C{^{1}H}-APT NMR, 101 MHz in $CDCl_3$

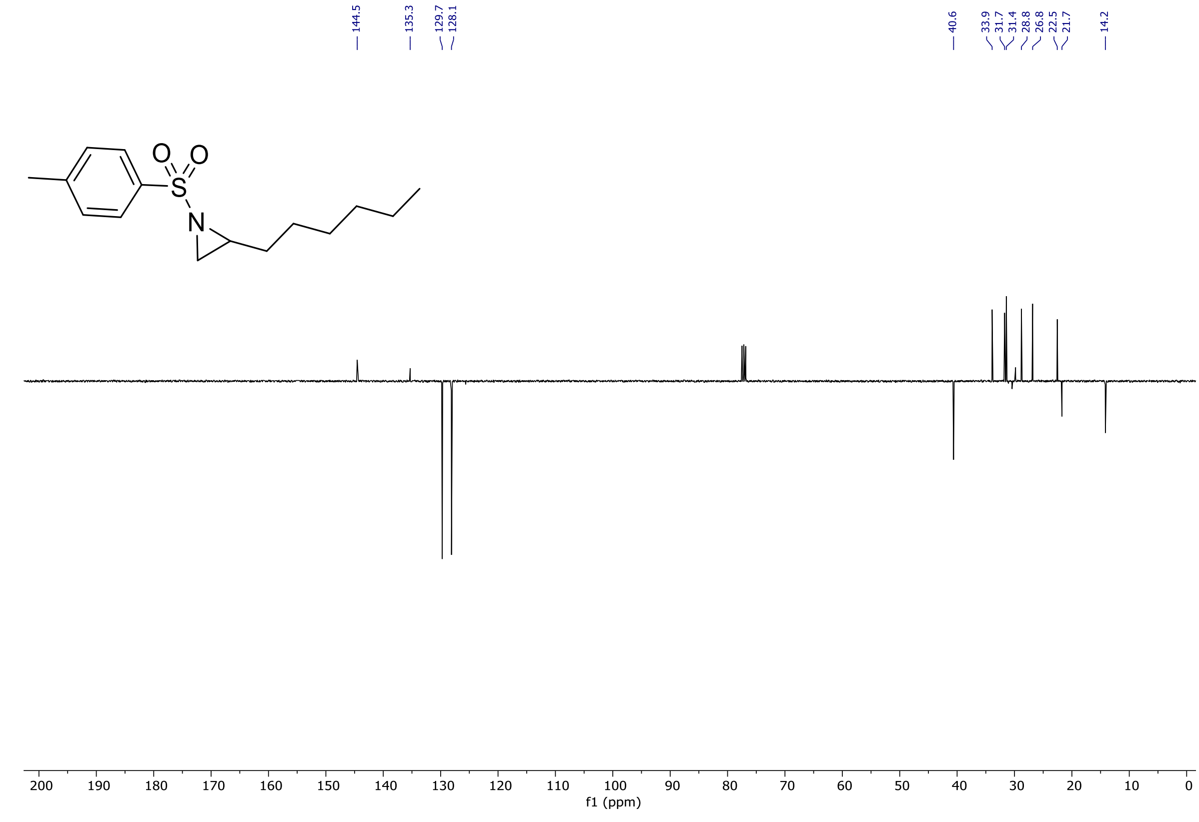

4b: ^{1}H-^{1}H COSY spectrum in $CDCl_3$

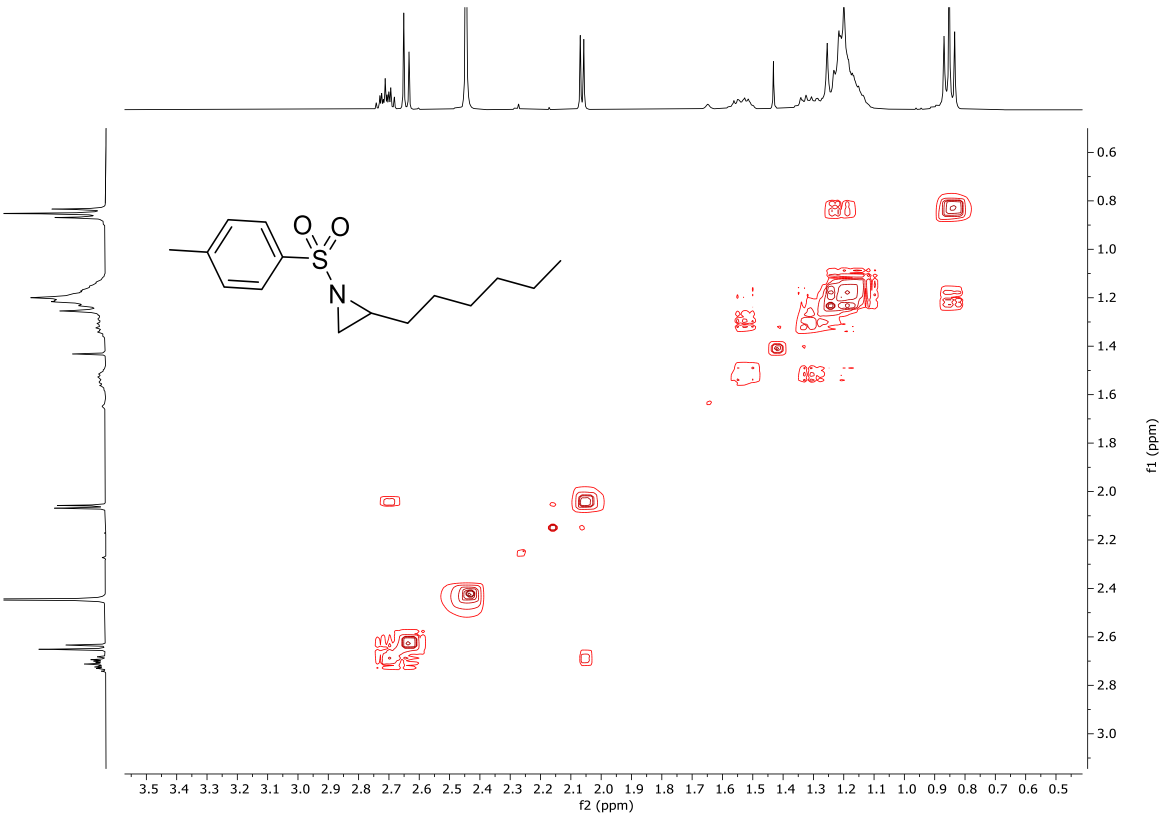

4b: ^{1}H-^{13}C HSQC spectrum in $CDCl_3$

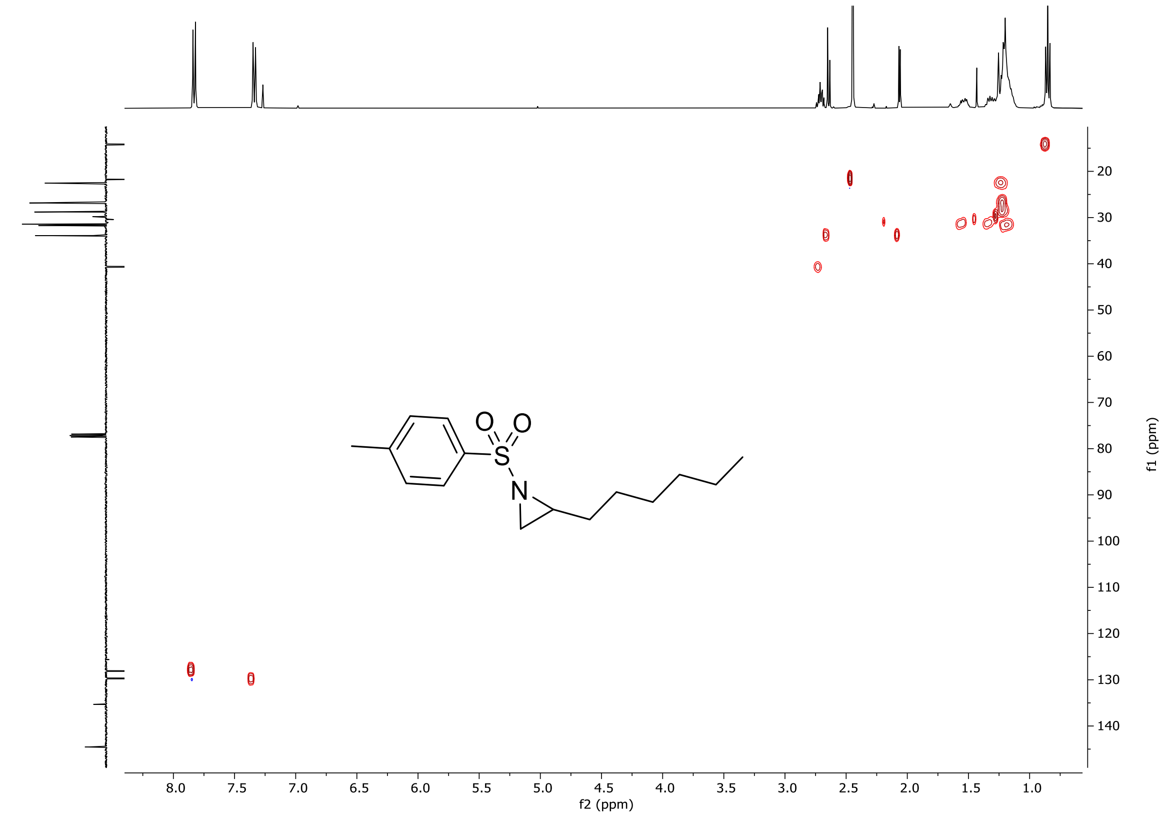

4c: ^{1}H NMR 400 MHz in $CDCl_3$

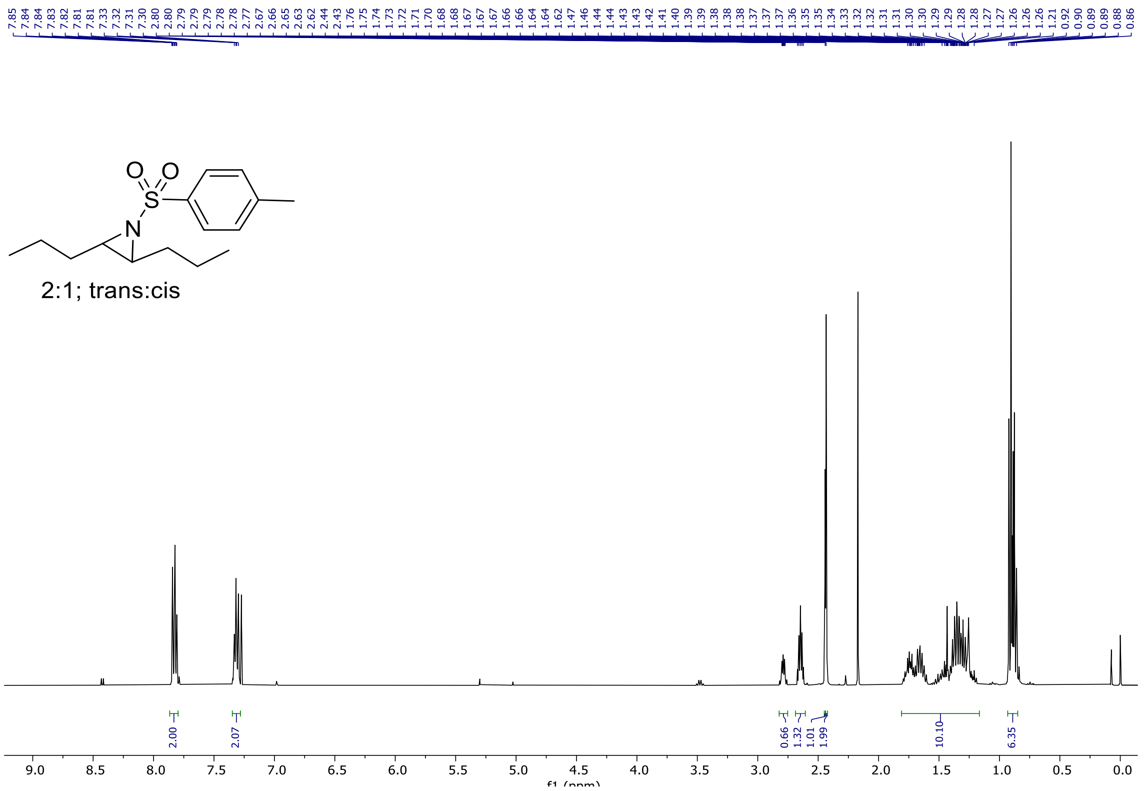

4c: $^{13}C\{^{1}H\}$-APT NMR, 101 MHz in $CDCl_3$

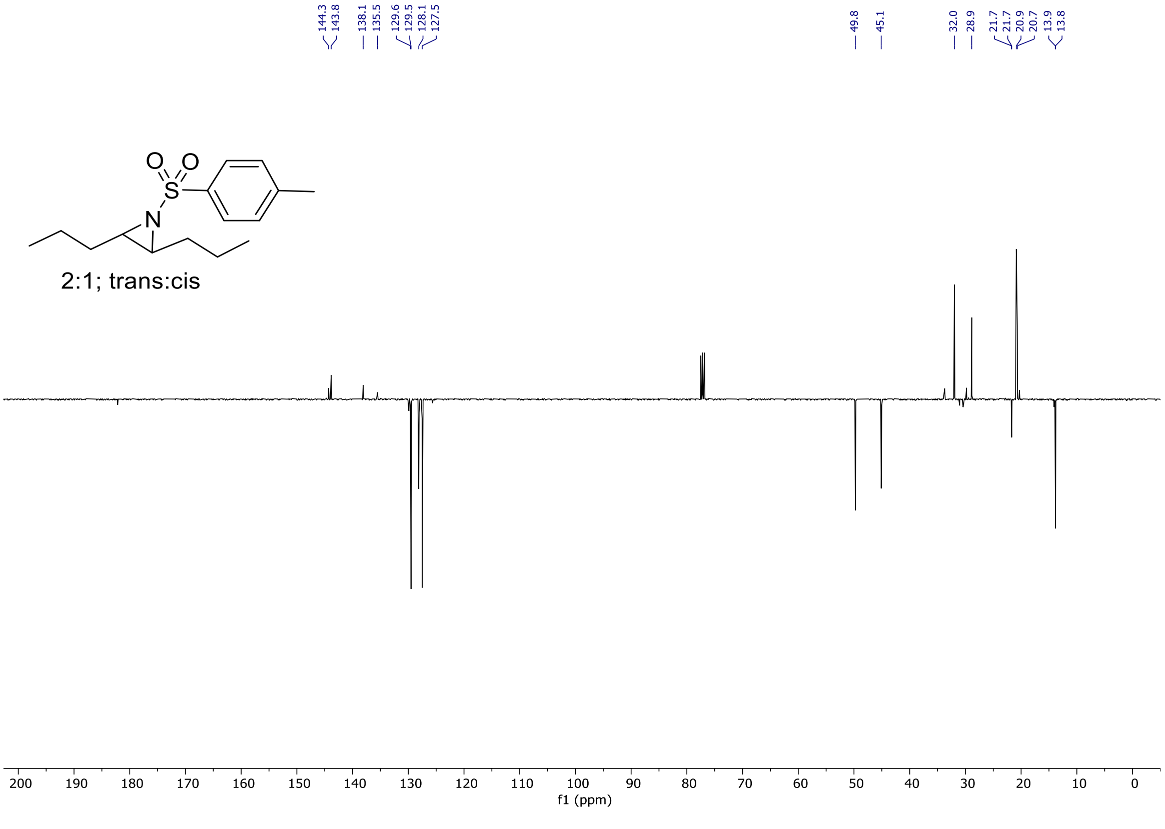

4c: ^{1}H-^{1}H COSY spectrum in $CDCl_3$

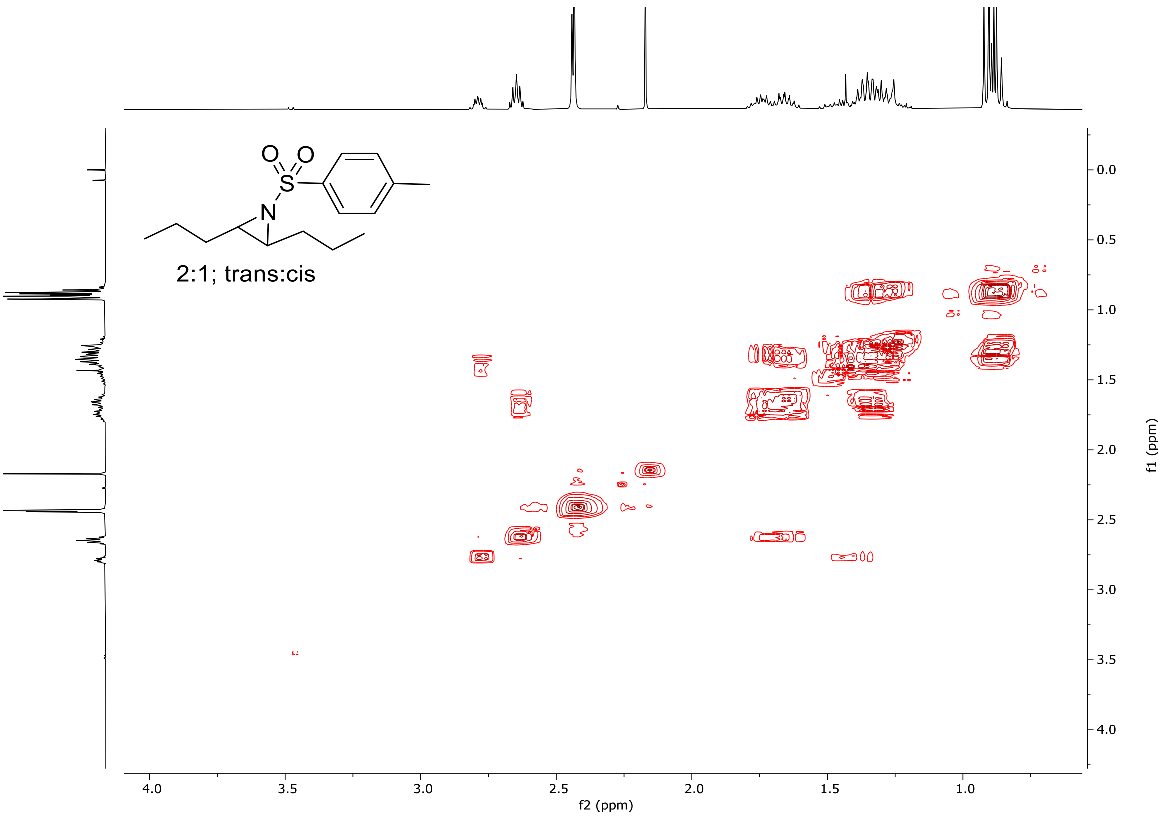

4c: ^{1}H-^{13}C HSQC spectrum in $CDCl_3$

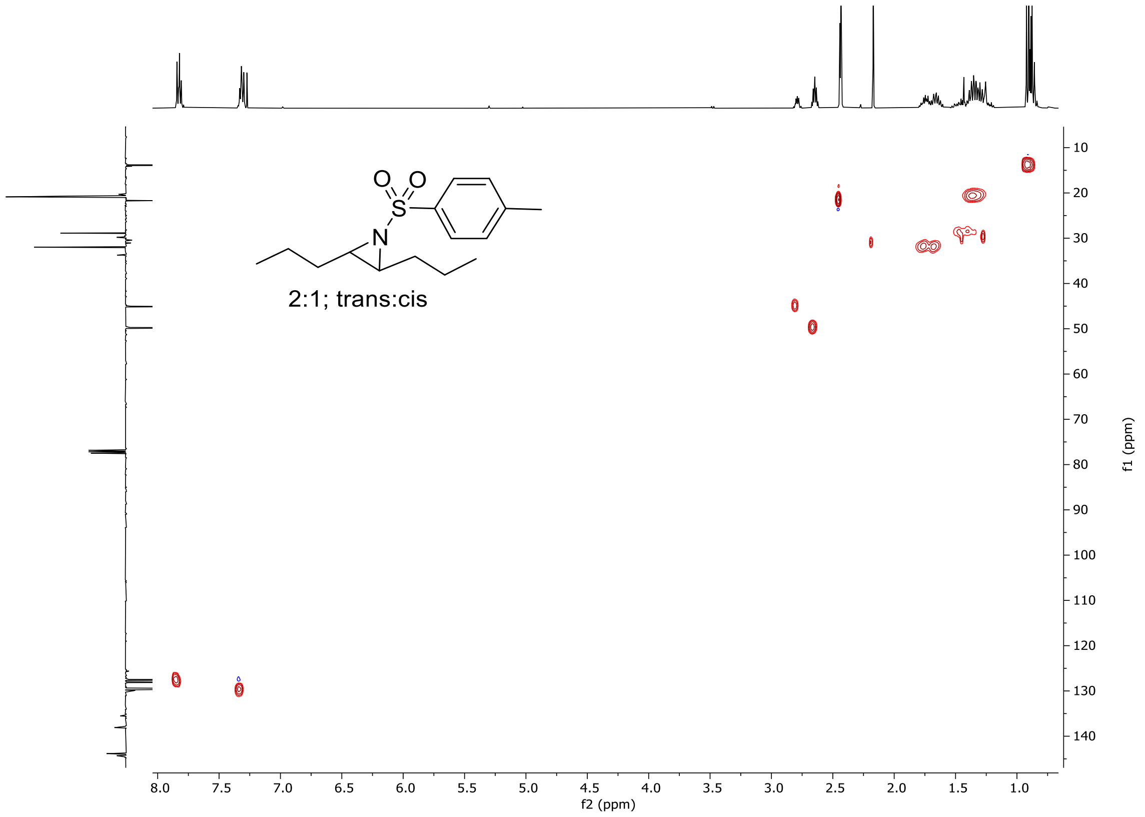

4d: ^{1}H NMR, 400 MHz in $CDCl_3$

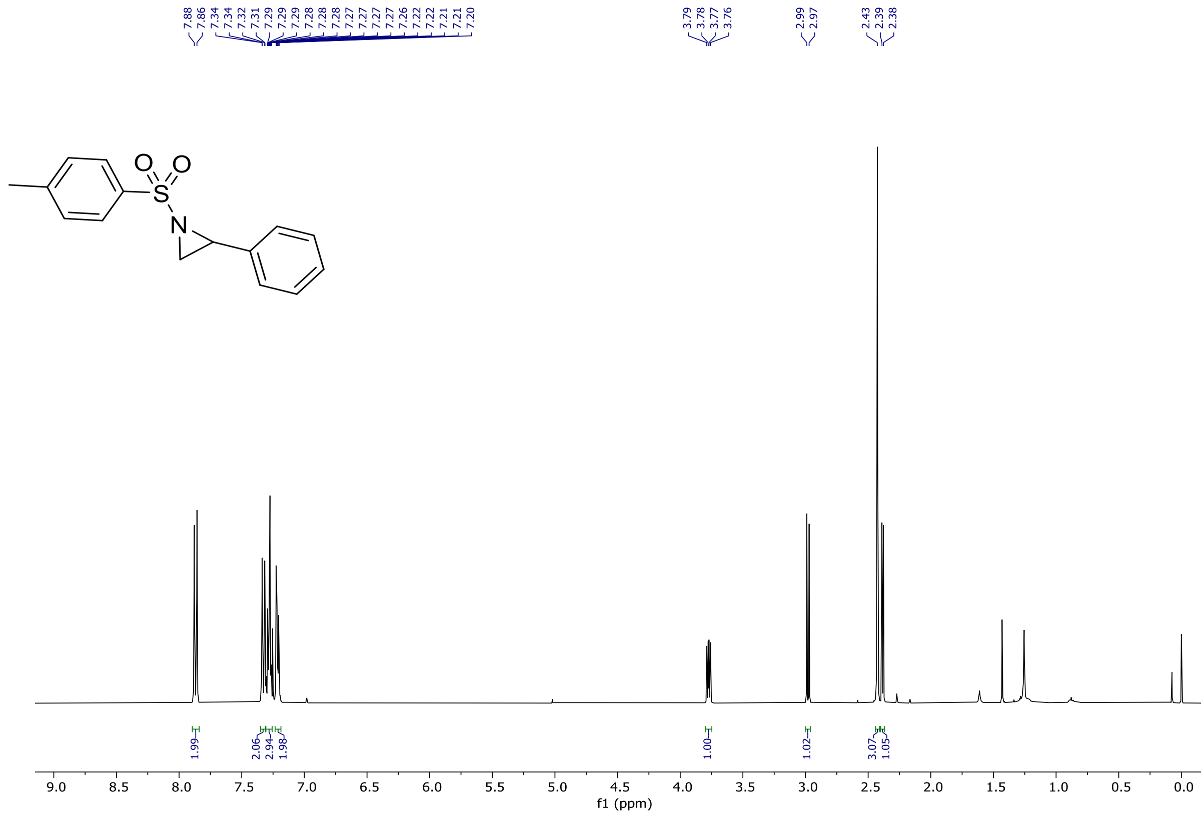

4d: ^{13}C{^{1}H}-APT NMR, 101 MHz in $CDCl_3$

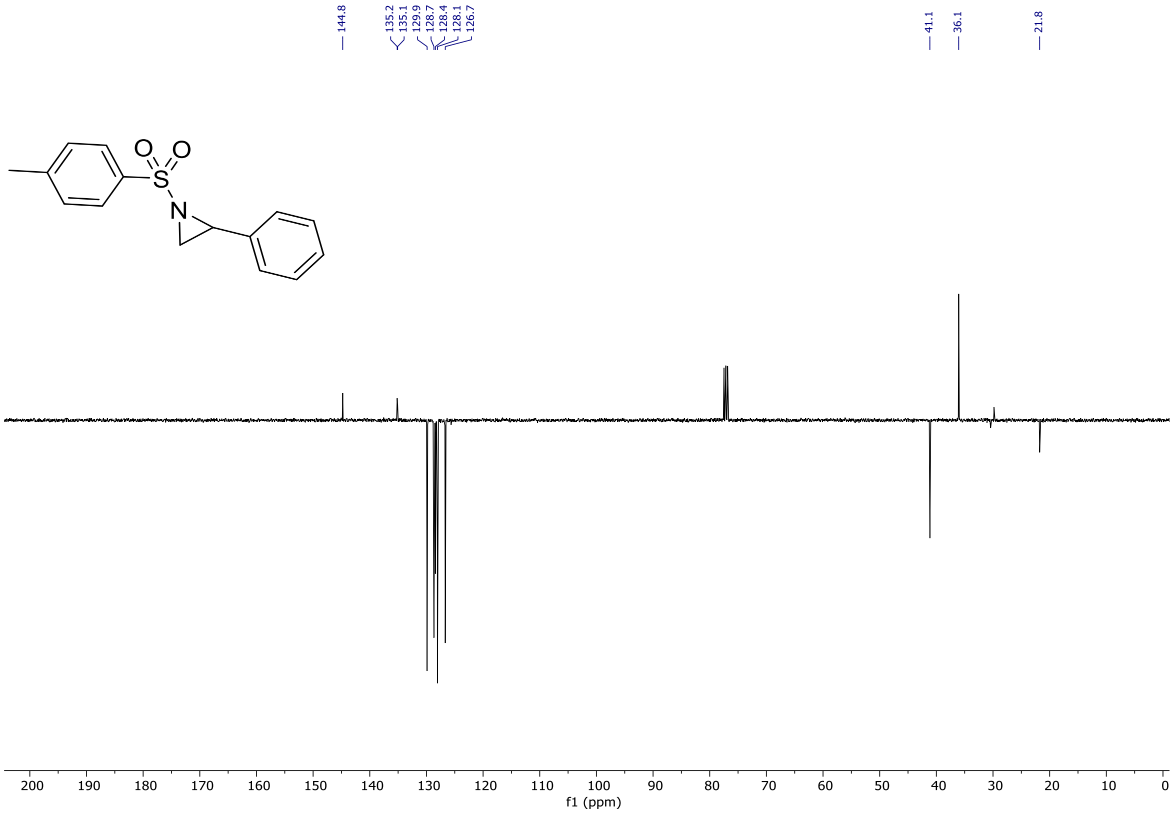

4e: ^{1}H NMR, 400 MHz in $CDCl_3$

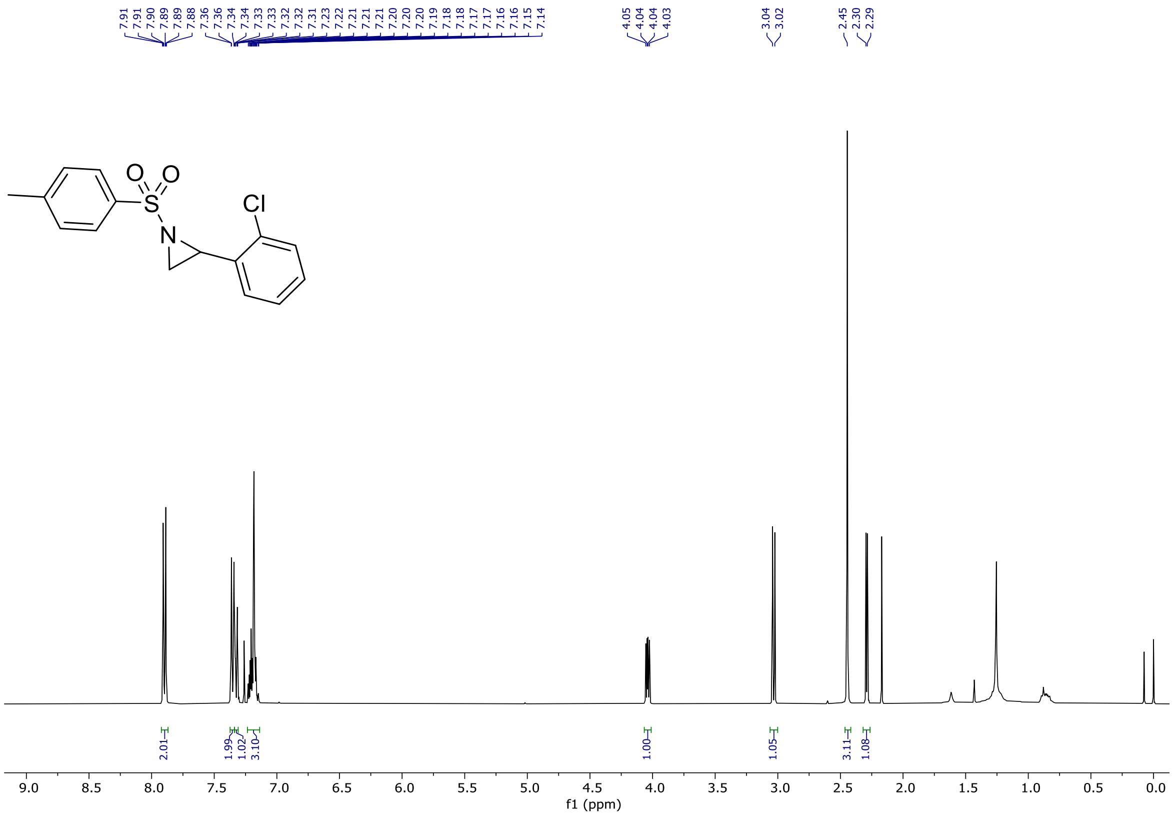

4e: ^{13}C{^{1}H}-APT NMR, 101 MHz in $CDCl_3$

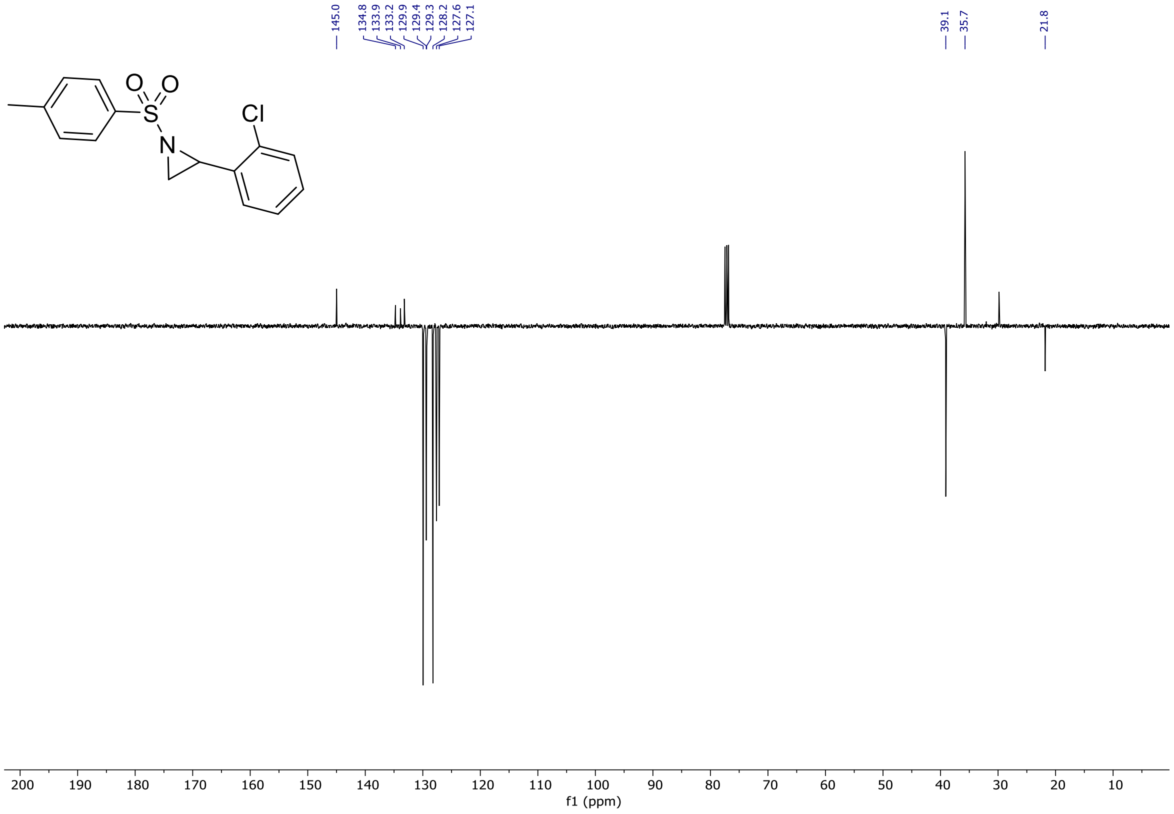

4e: ^{1}H-^{1}H COSY spectrum in $CDCl_3$

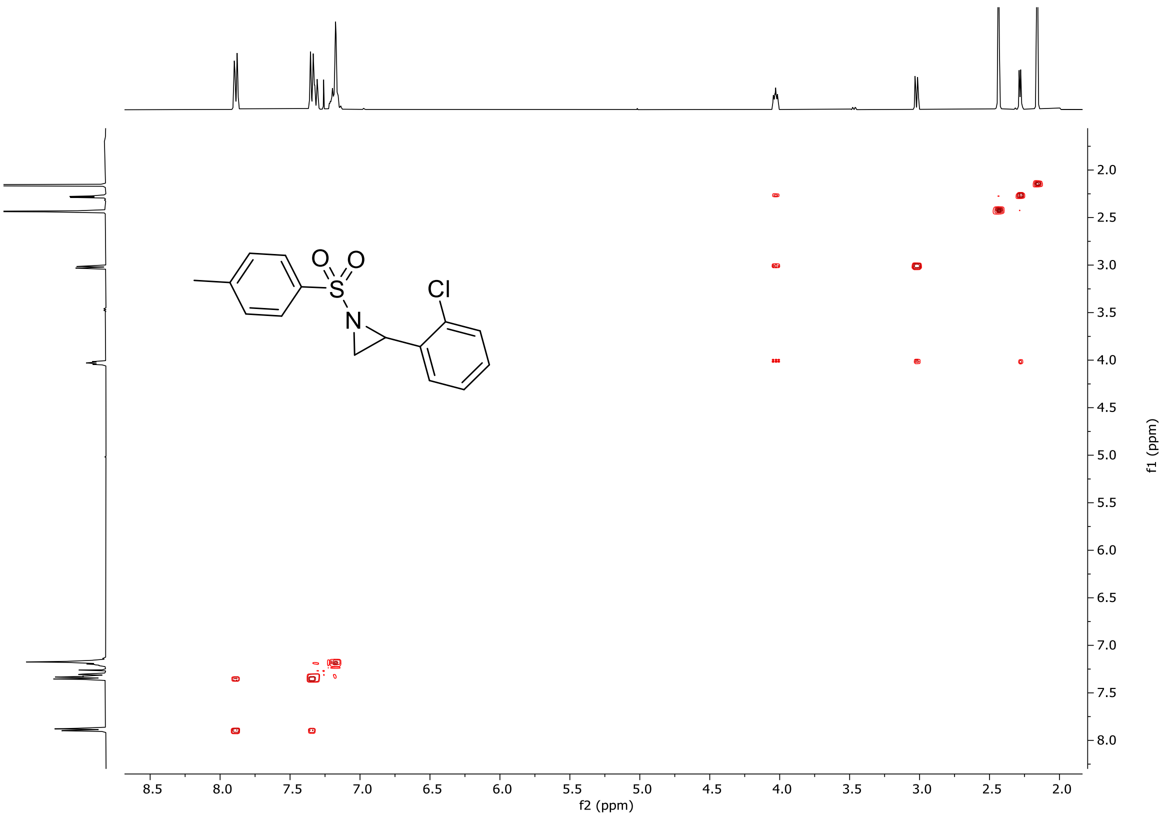

4e: ^{1}H-^{13}C HSQC spectrum in $CDCl_3$

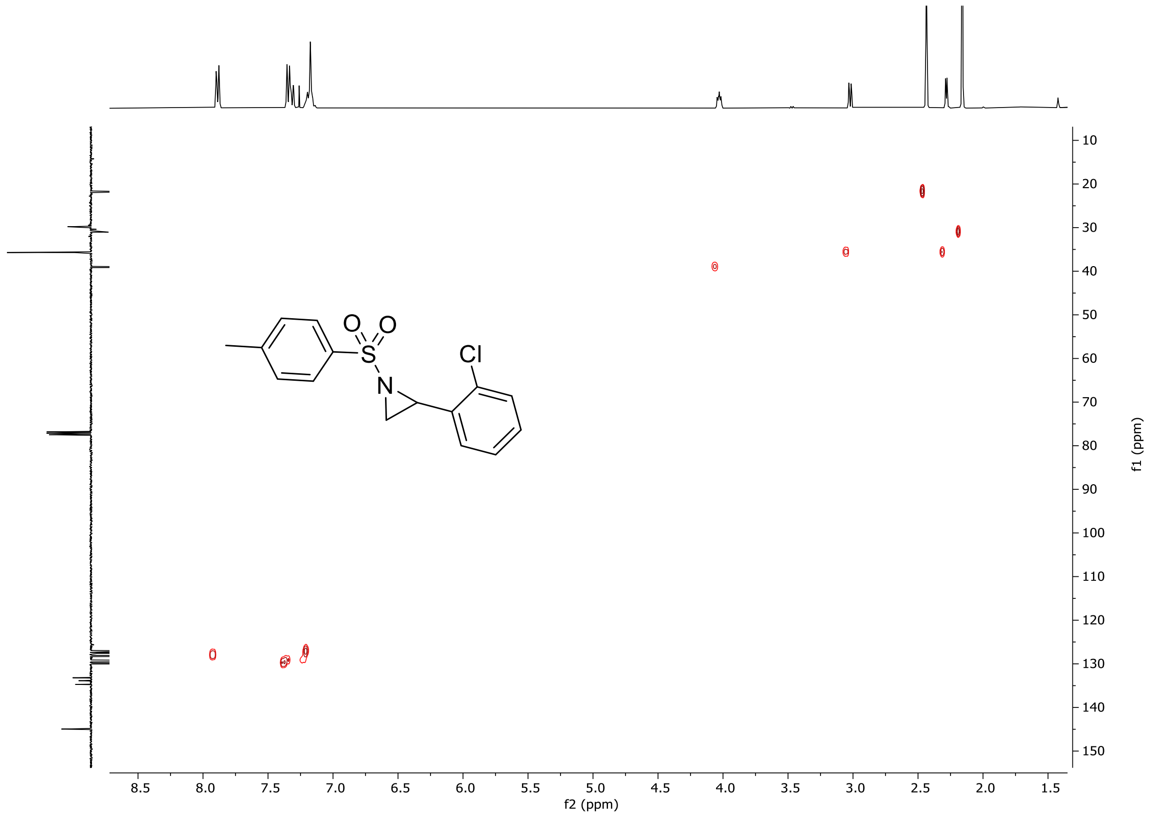

4f: ^{1}H NMR, 400 MHz in $CDCl_3$

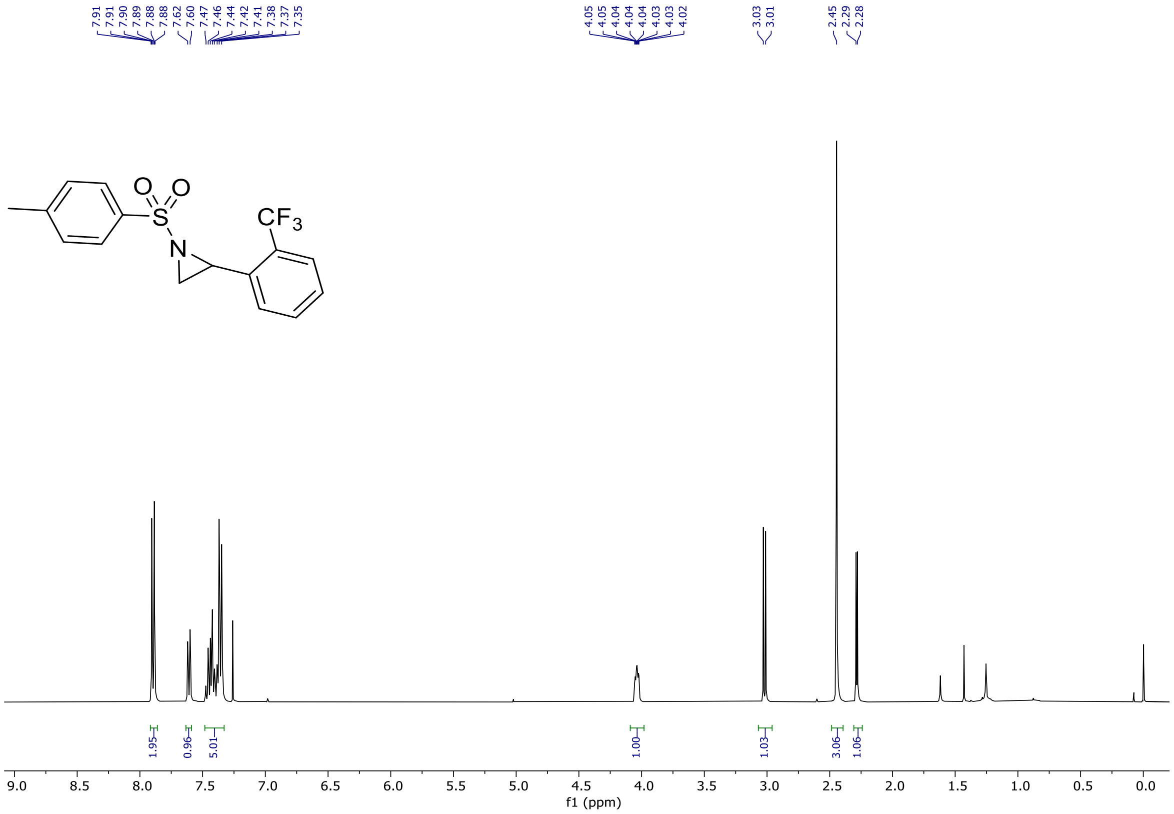

4f: ^{13}C{^{1}H}-APT NMR, 101 MHz in $CDCl_3$

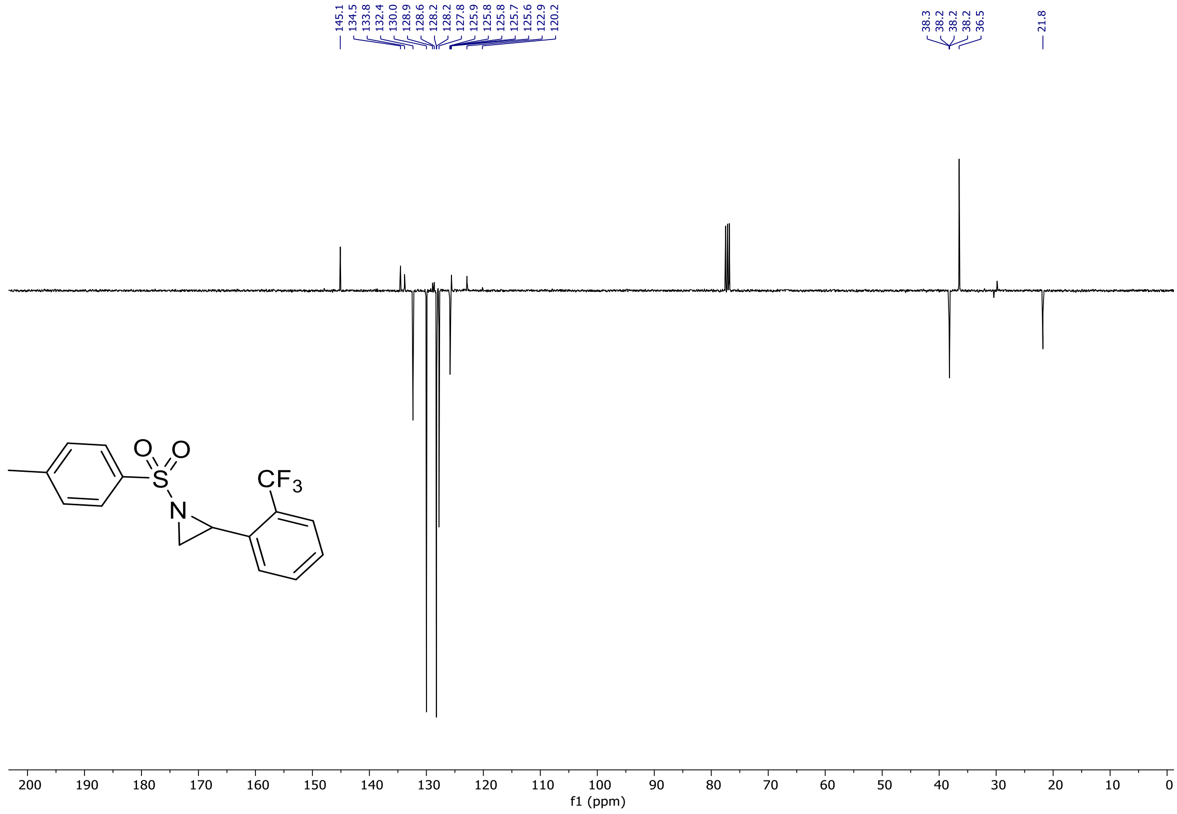

4f: ^{19}F NMR, 376 MHz in $CDCl_3$

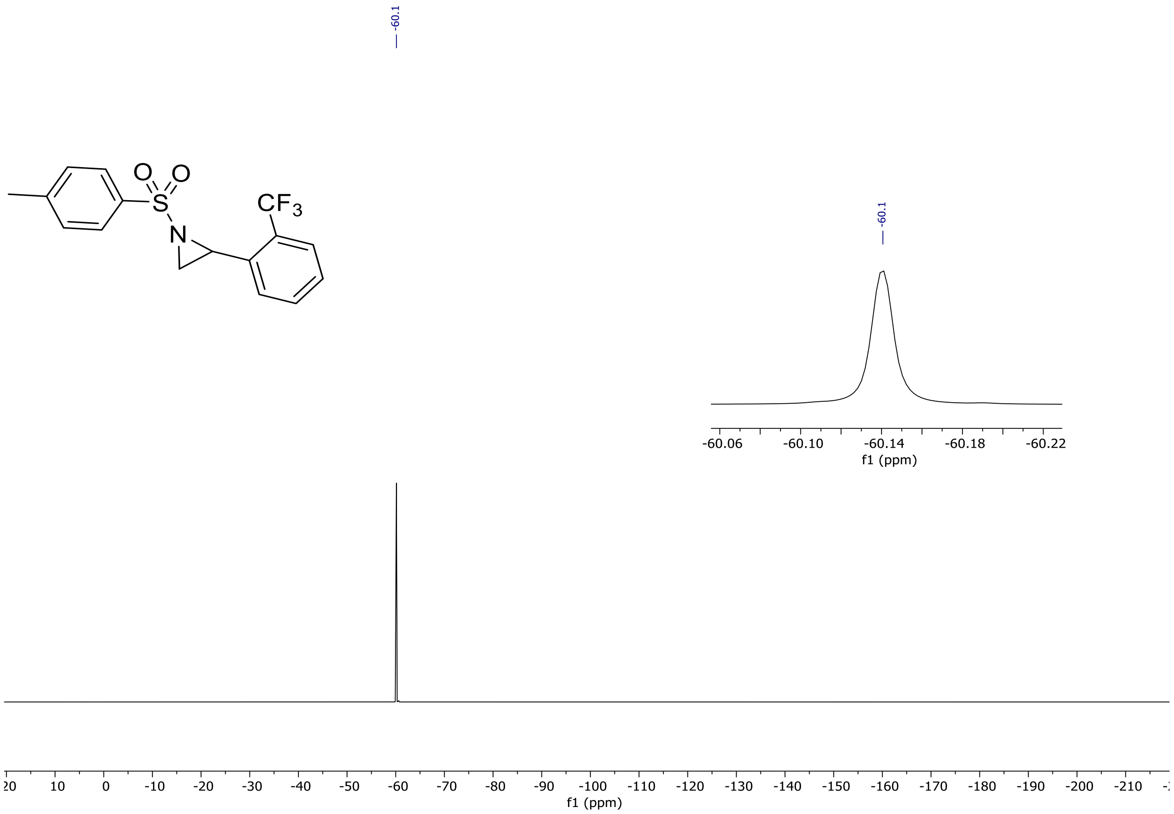

4f: ^{1}H-^{1}H COSY spectrum in $CDCl_3$

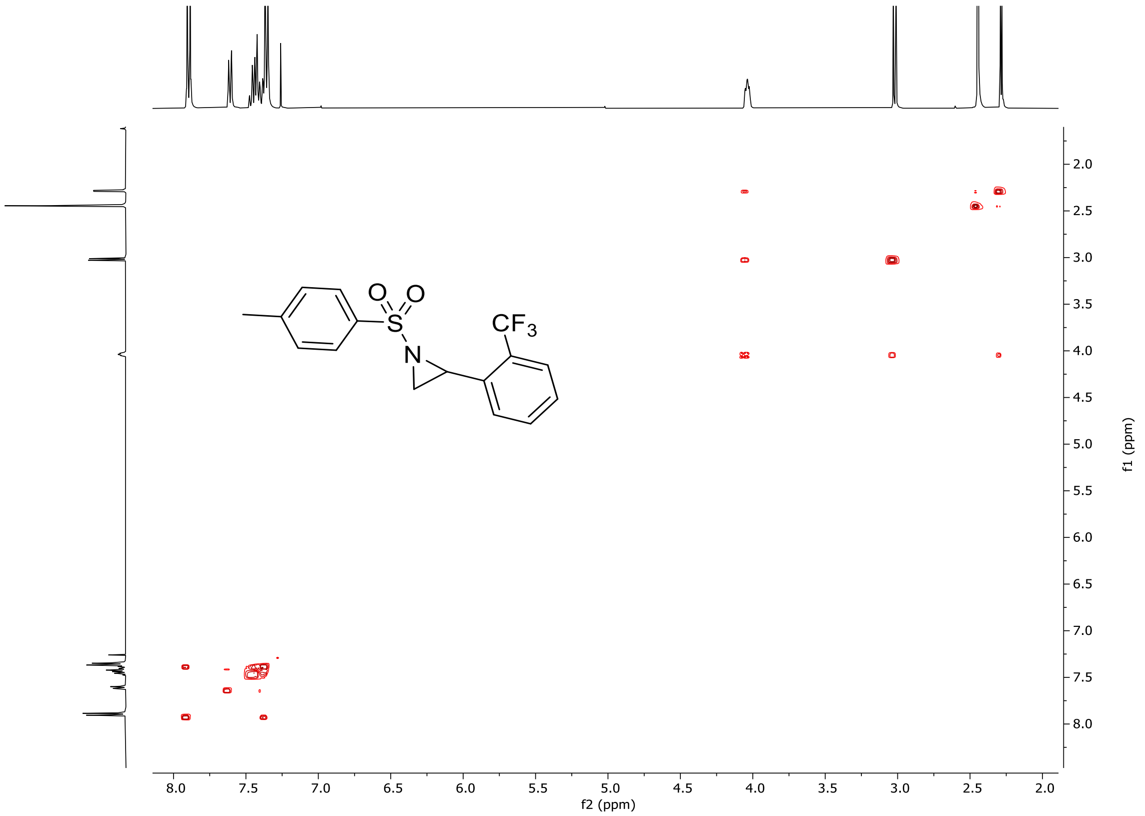

4f: ^{1}H-^{13}C HSQC spectrum in $CDCl_3$

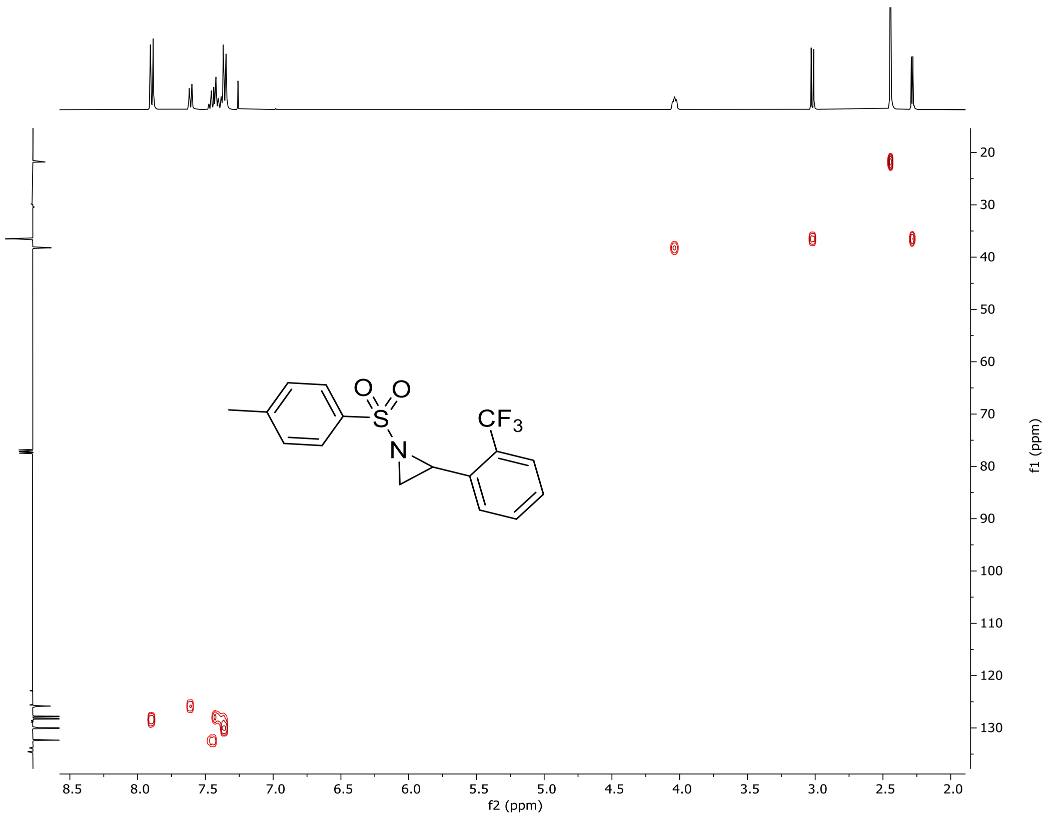

4g: ^{1}H NMR, 400 MHz in $CDCl_3$

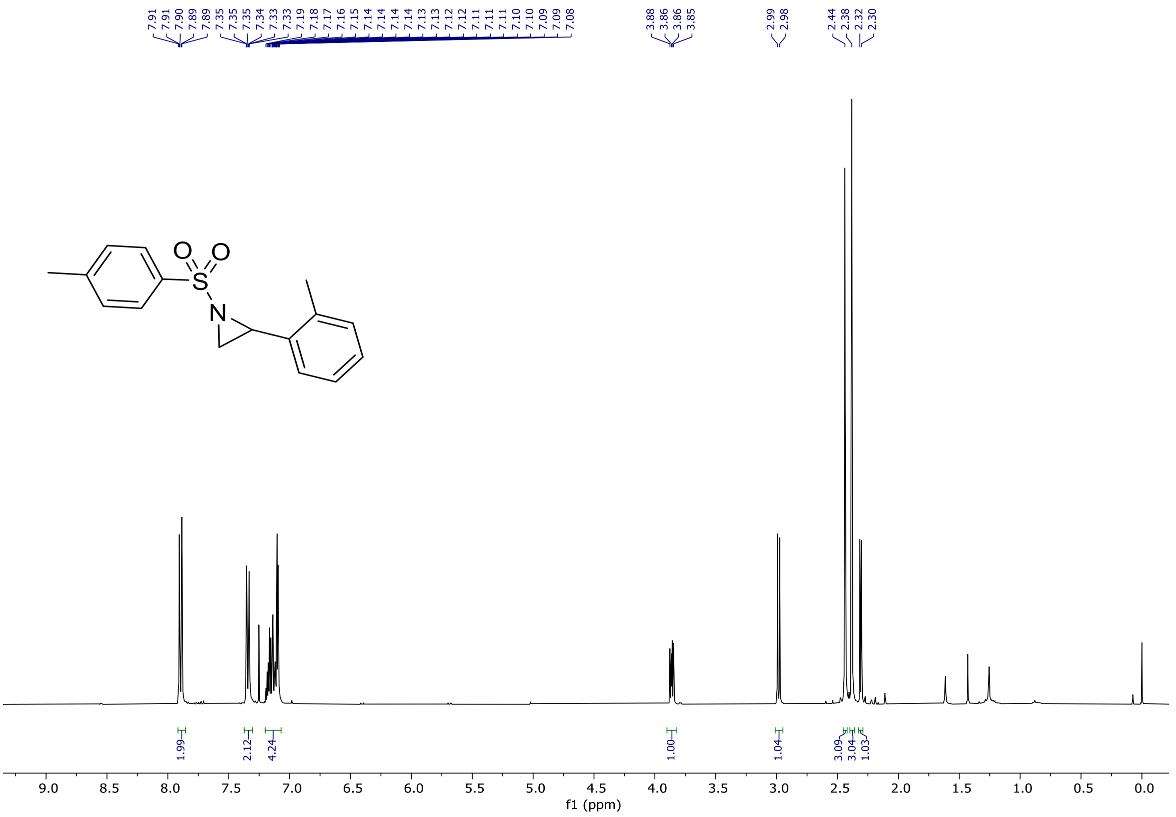

4g: $^{13}C\{^{1}H\}$-APT NMR, 101 MHz in $CDCl_3$

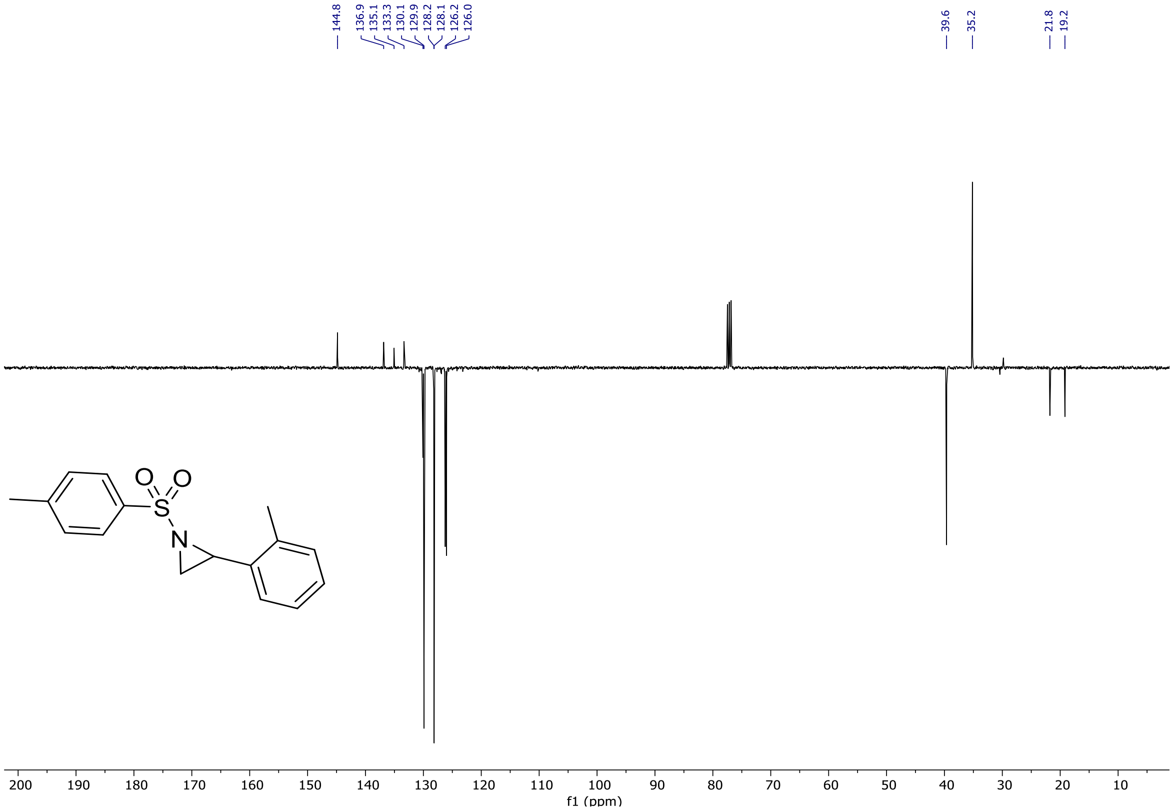

4g: ^{1}H-^{1}H COSY spectrum in $CDCl_3$

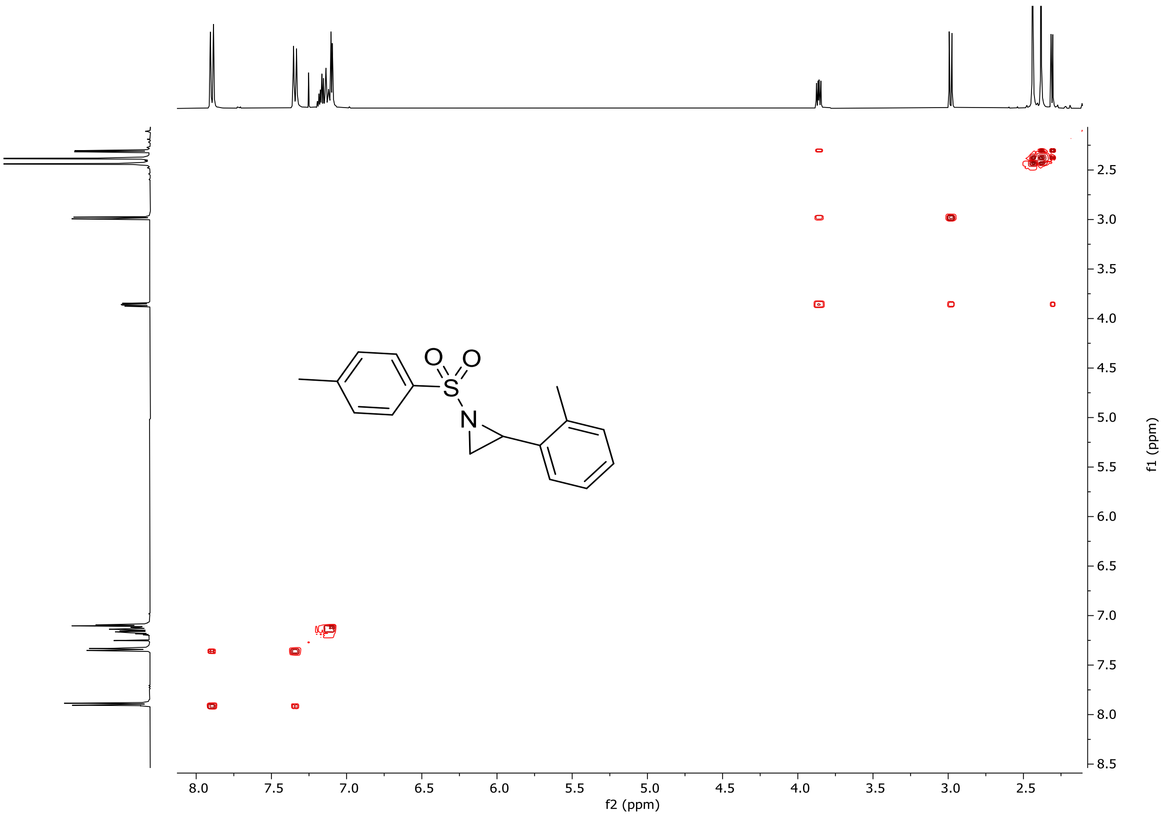

4g: ^{1}H-^{13}C HSQC spectrum in $CDCl_3$

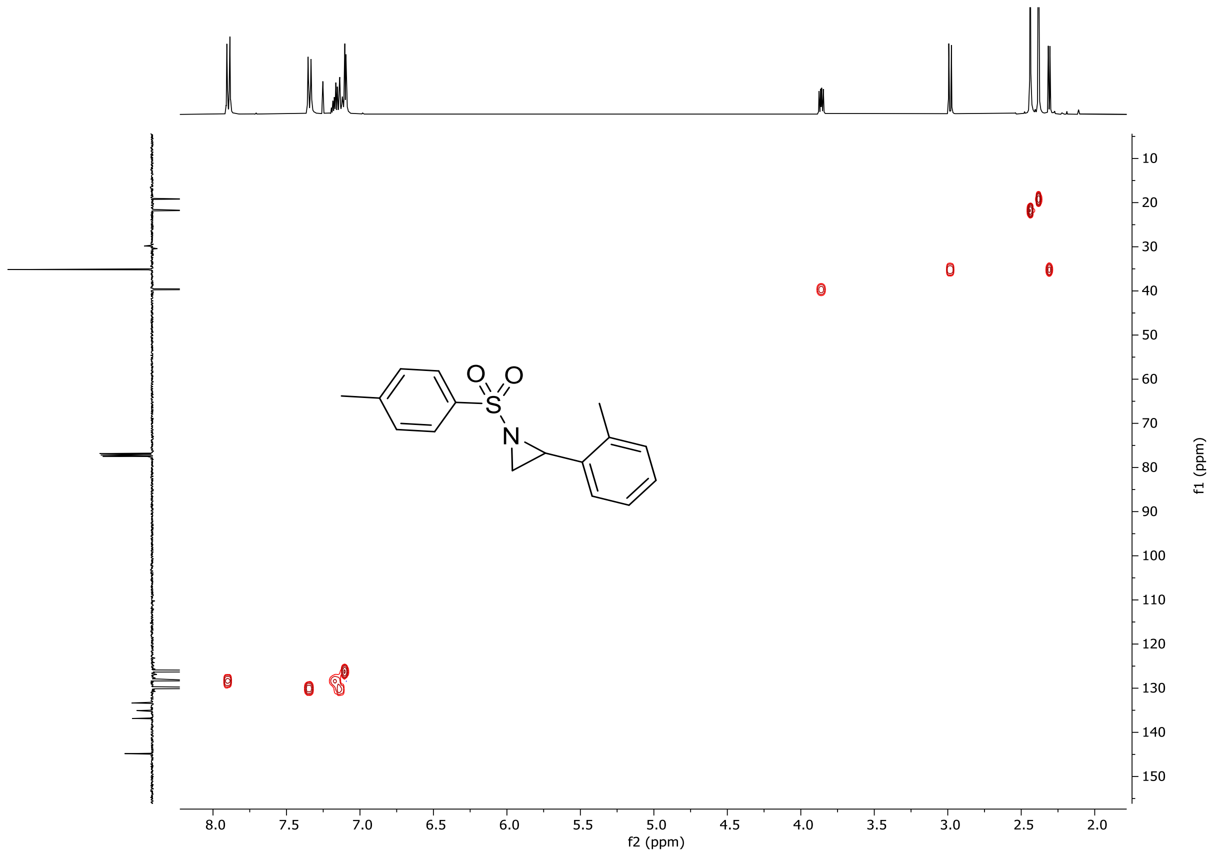

4h: ^{1}H NMR, 400 MHz in $CDCl_3$

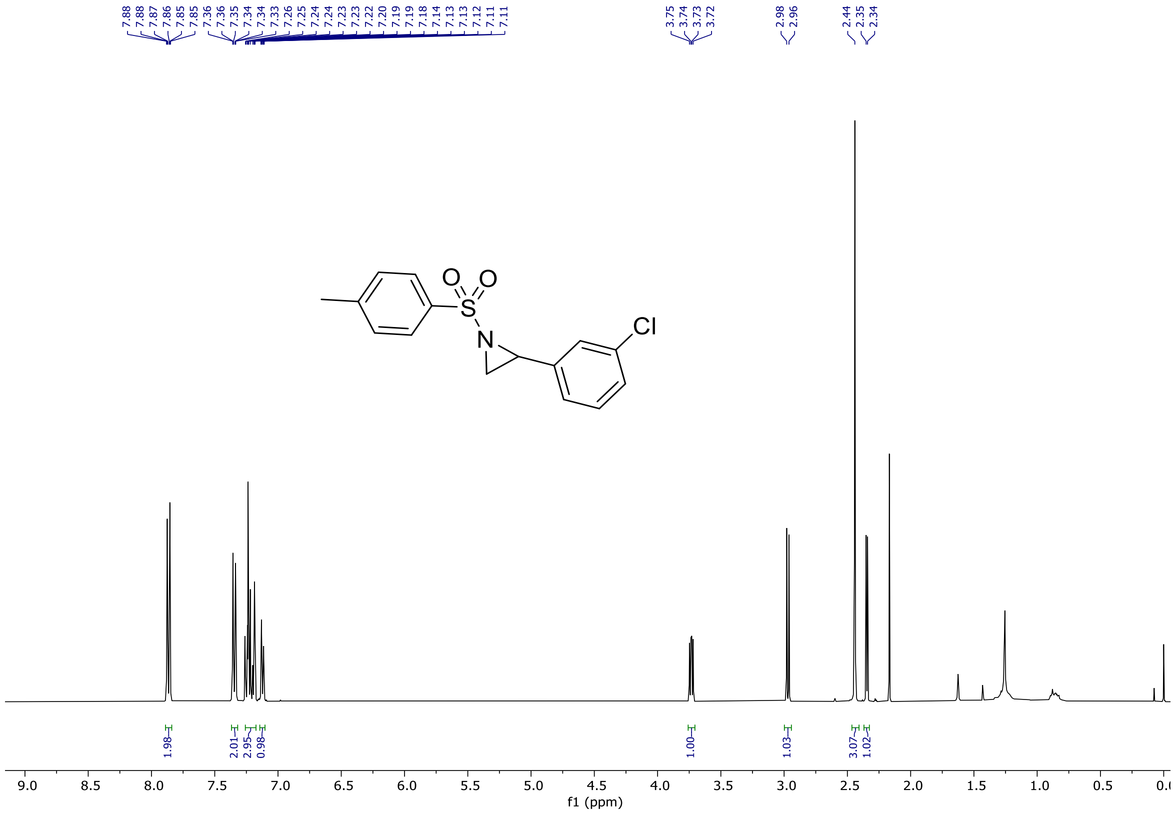

4h: $^{13}C\{^{1}H\}$-APT NMR, 400 MHz in $CDCl_3$

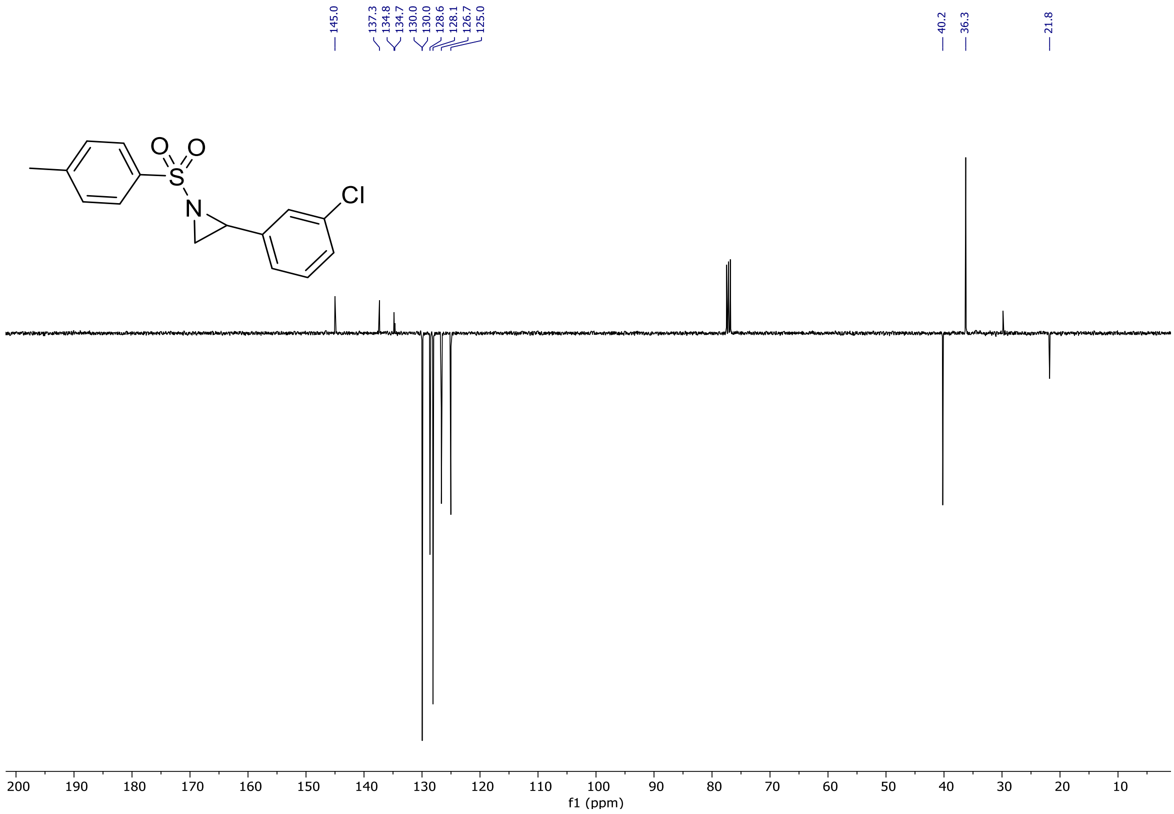

4h: ^{1}H-^{1}H COSY spectrum in $CDCl_3$

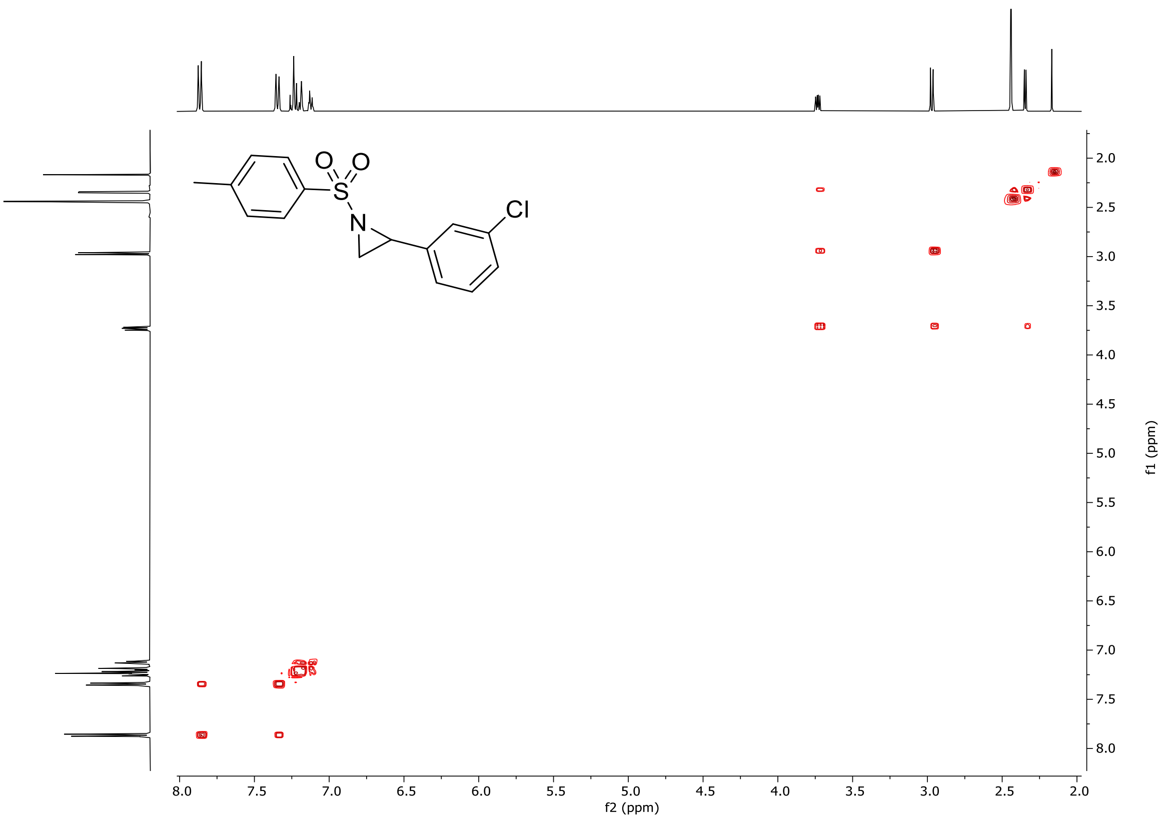

4h: ^{1}H-^{13}C HSQC spectrum in $CDCl_3$

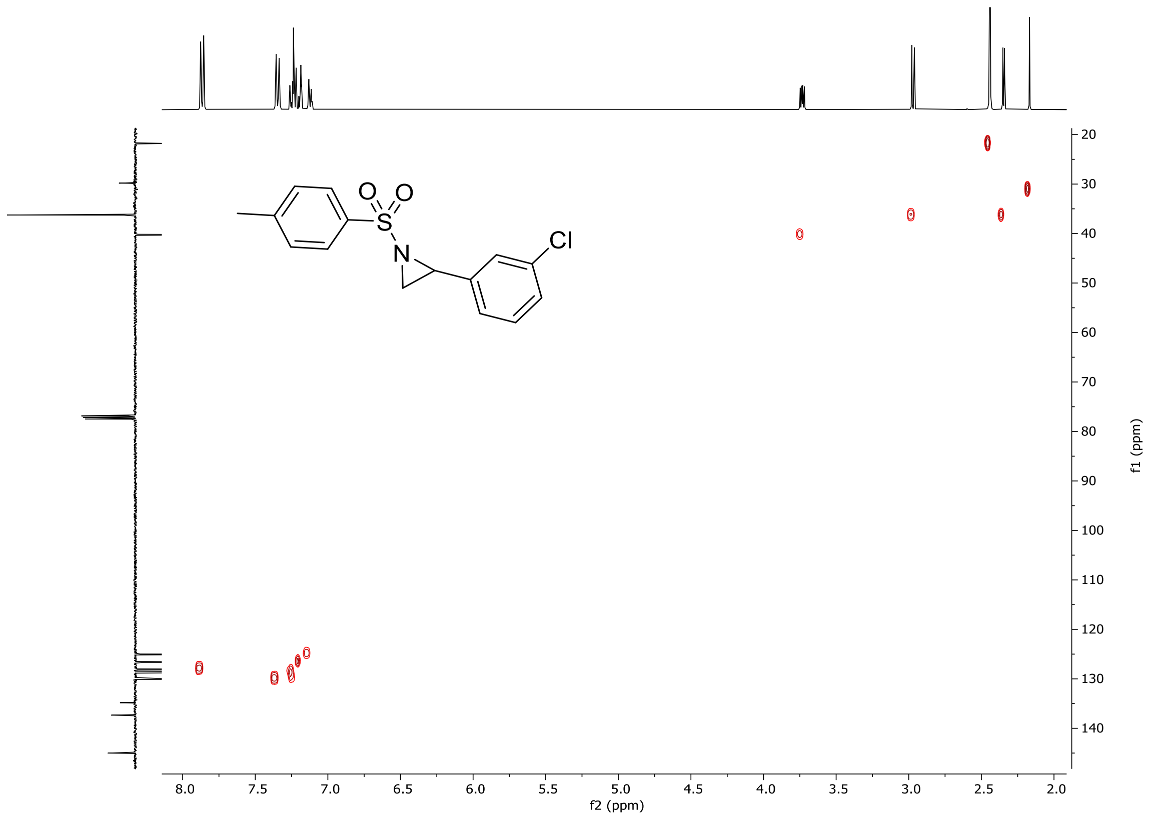

4i: ^{1}H NMR, 400 MHz in $CDCl_3$

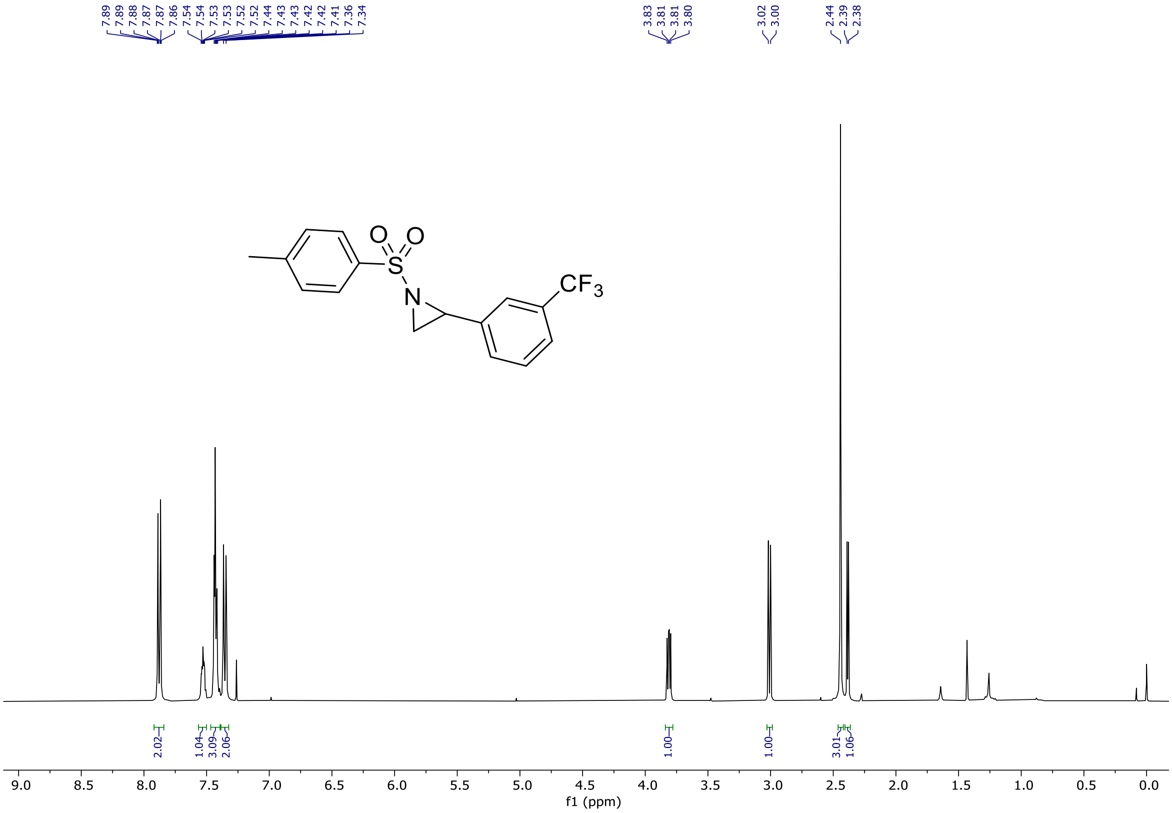

4i: $^{13}C\{^{1}H\}$-APT NMR, 101 MHz in $CDCl_3$

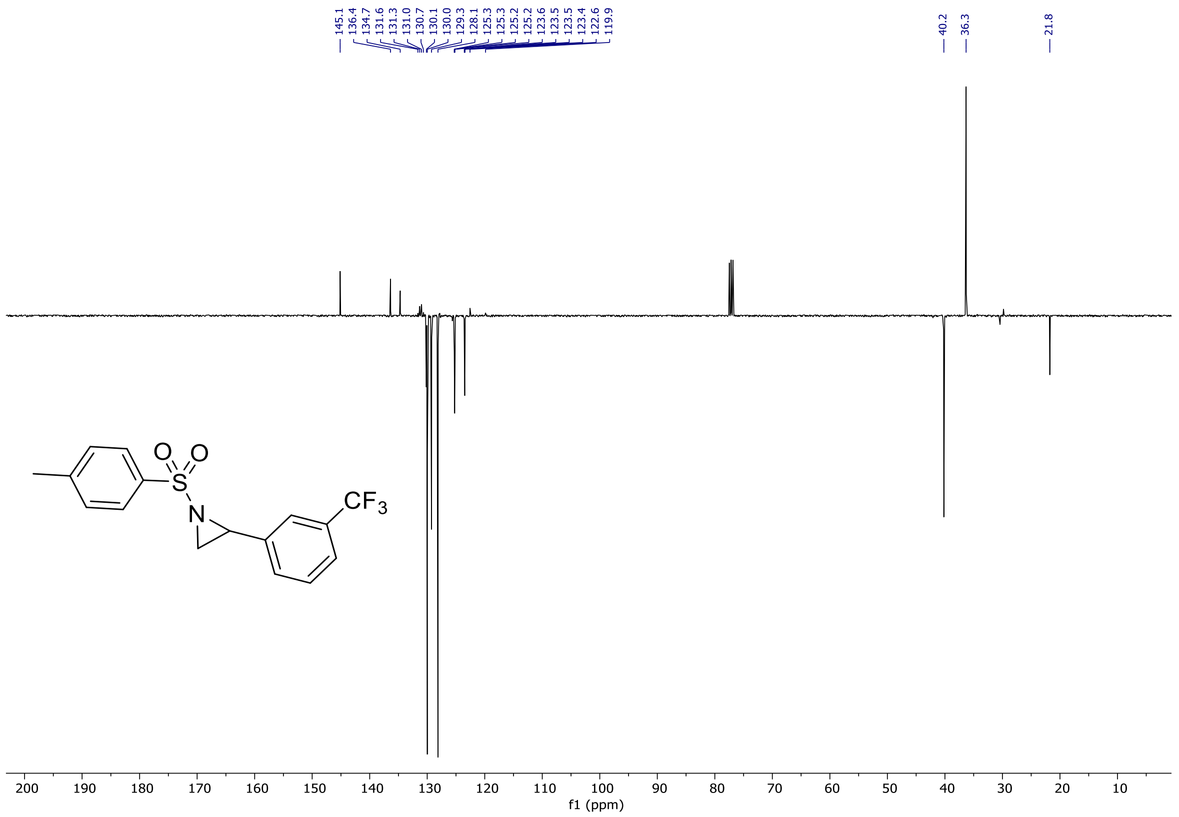

4i: ^{19}F NMR, 376 MHz in $CDCl_3$

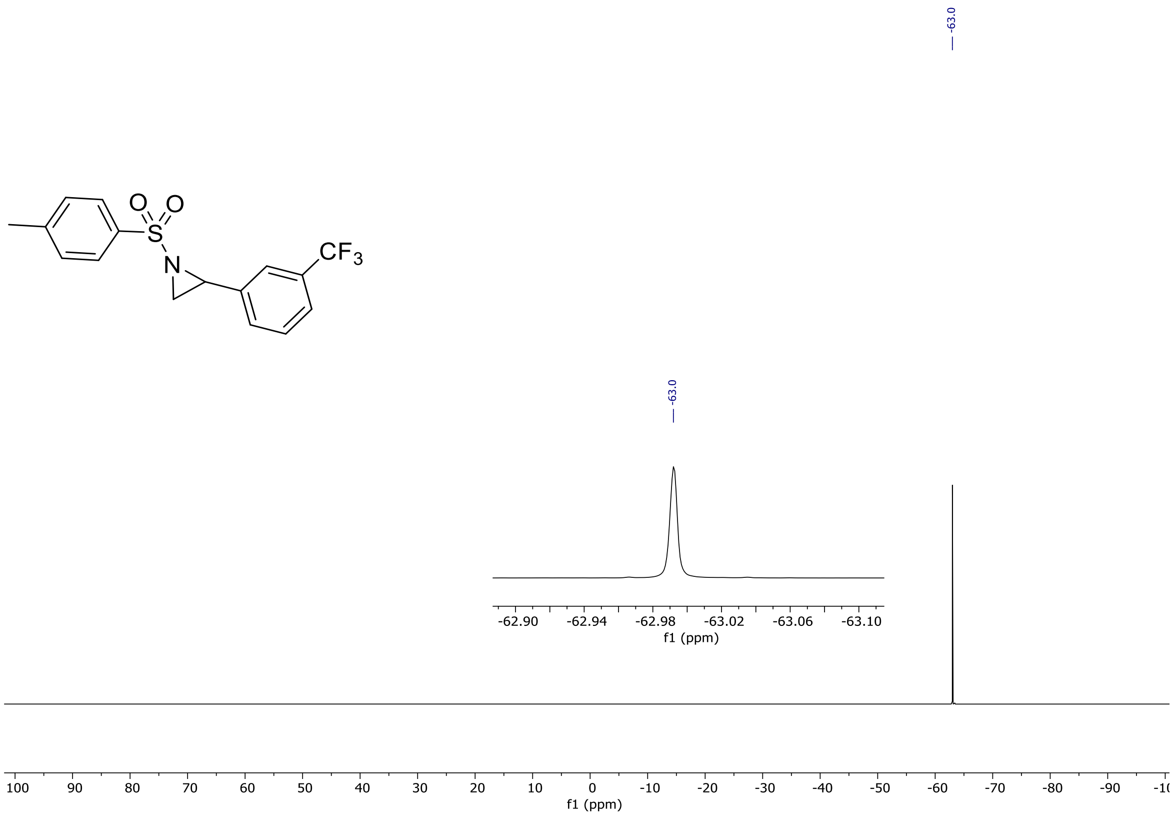

4i: ^{1}H-^{1}H COSY spectrum in $CDCl_3$

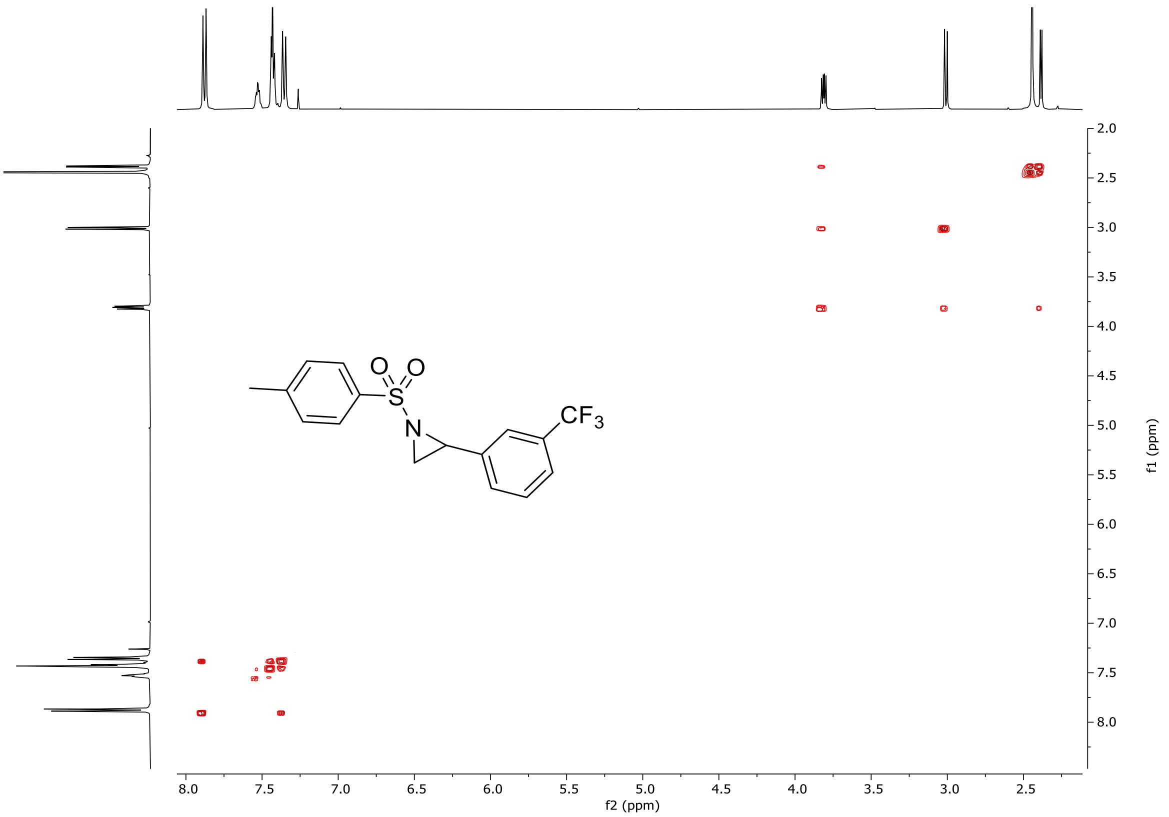

4i: ^{1}H-^{13}C HSQC spectrum in $CDCl_3$

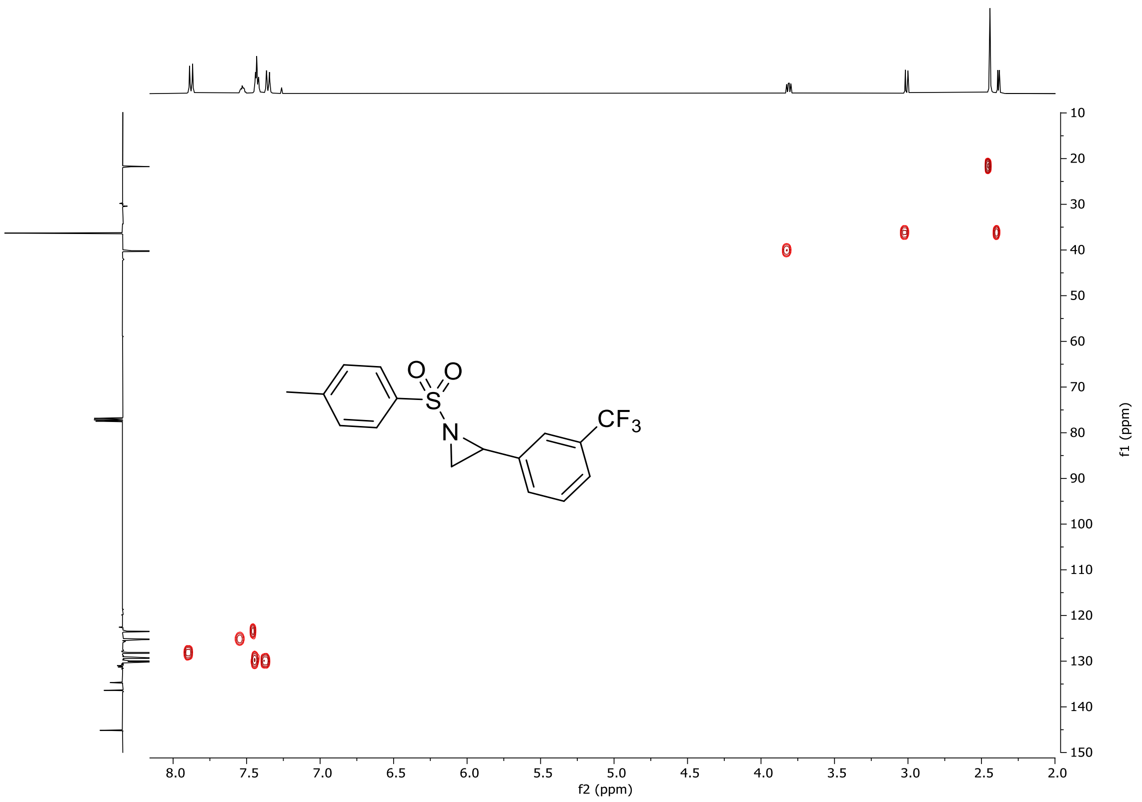

4j: ^{1}H NMR, 400 MHz in $CDCl_3$

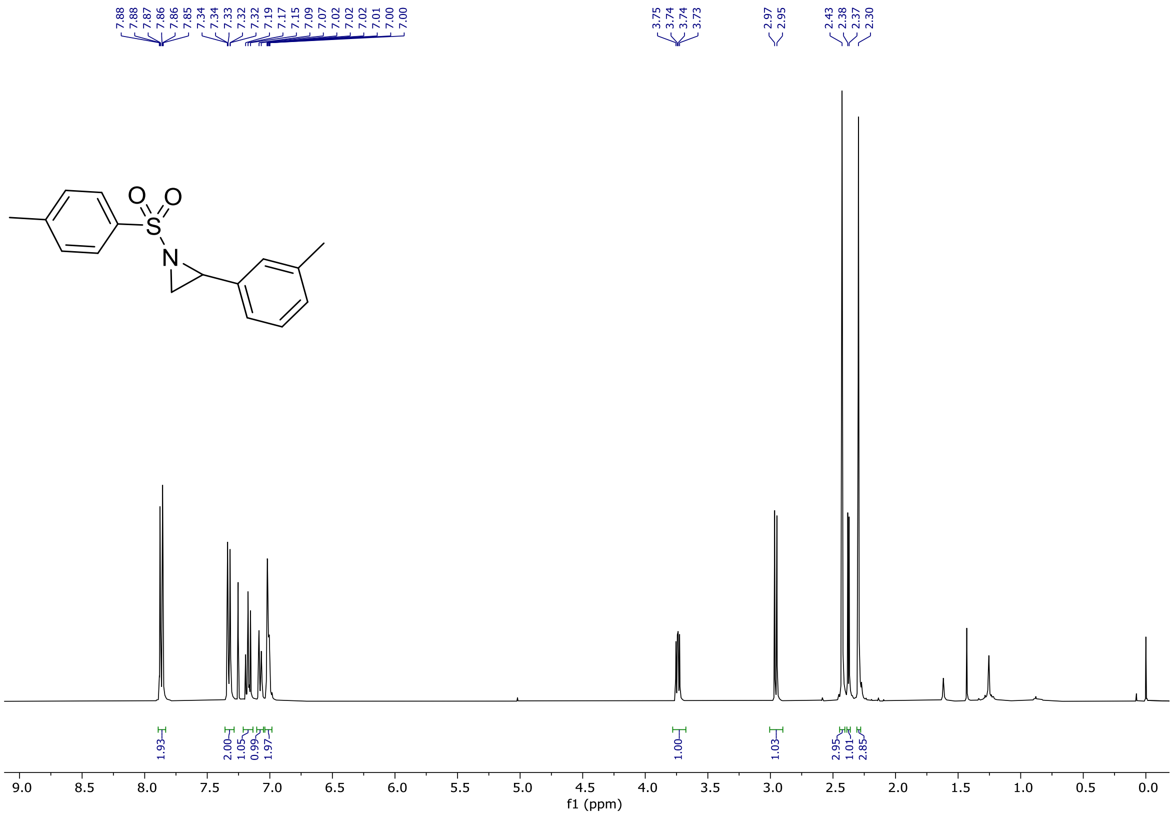

4j: ^{13}C{^{1}H}-APT NMR, 101 MHz in $CDCl_3$

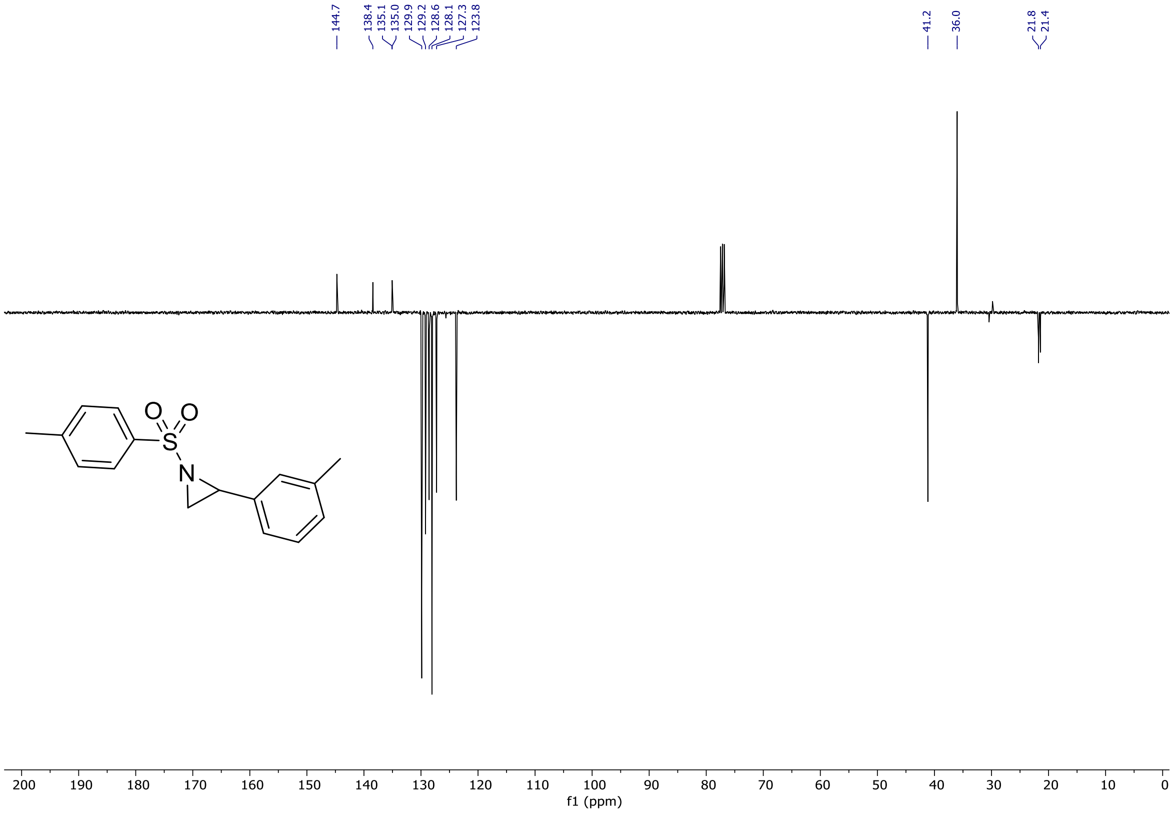

4j: ^{1}H-^{1}H COSY spectrum in $CDCl_3$

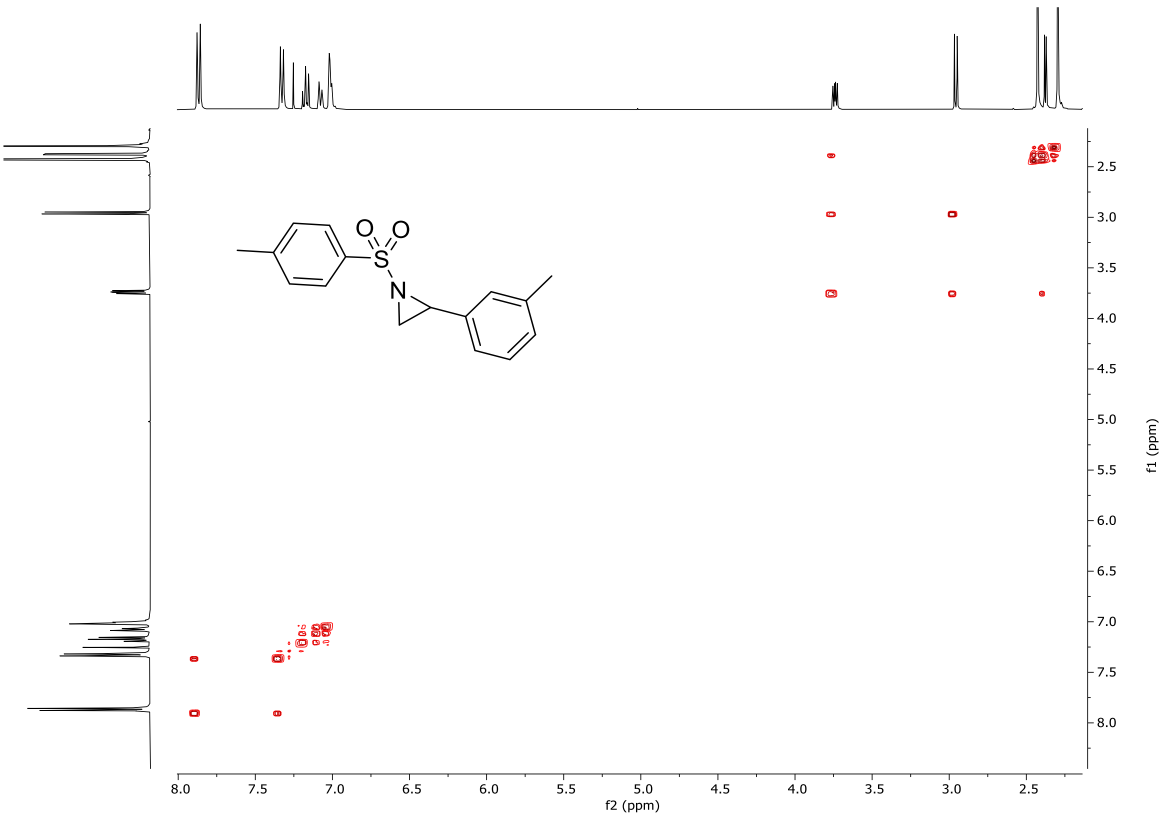

4j: ^{1}H-^{13}C HSQC spectrum in $CDCl_3$

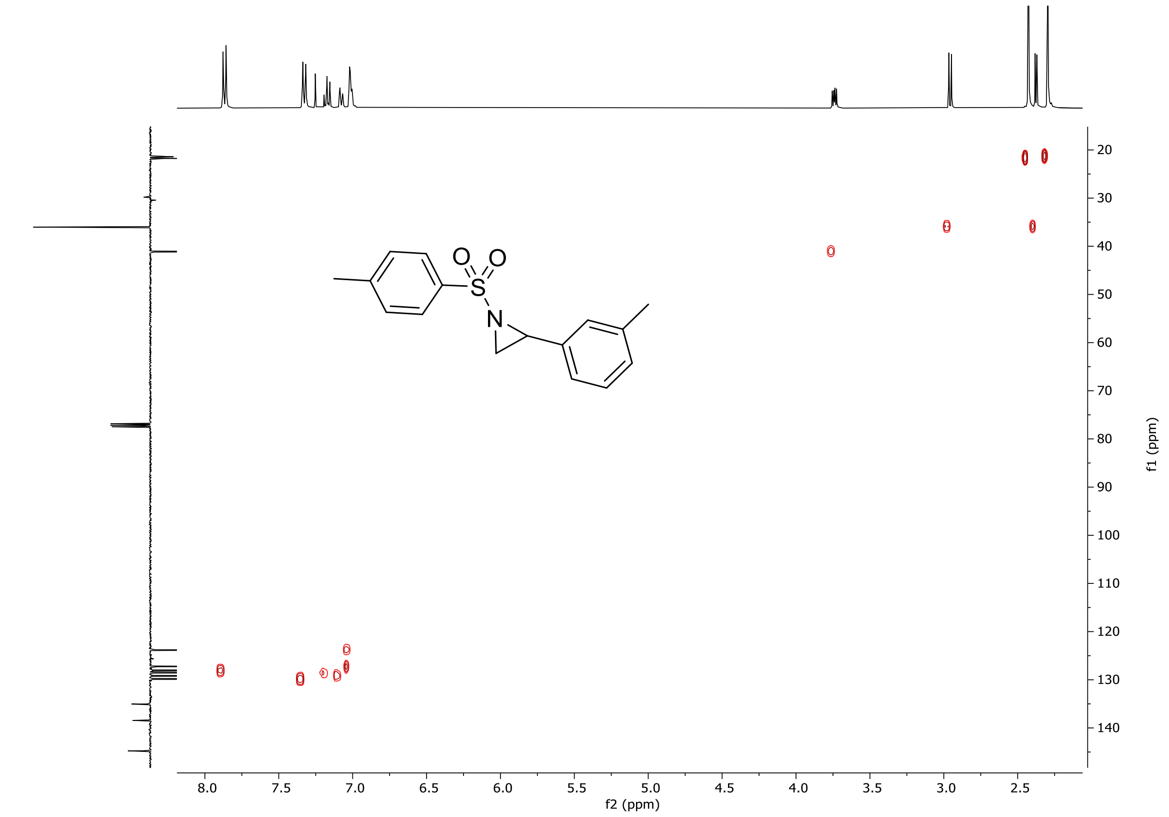

4k: ^{1}H NMR, 400 MHz in $CDCl_3$

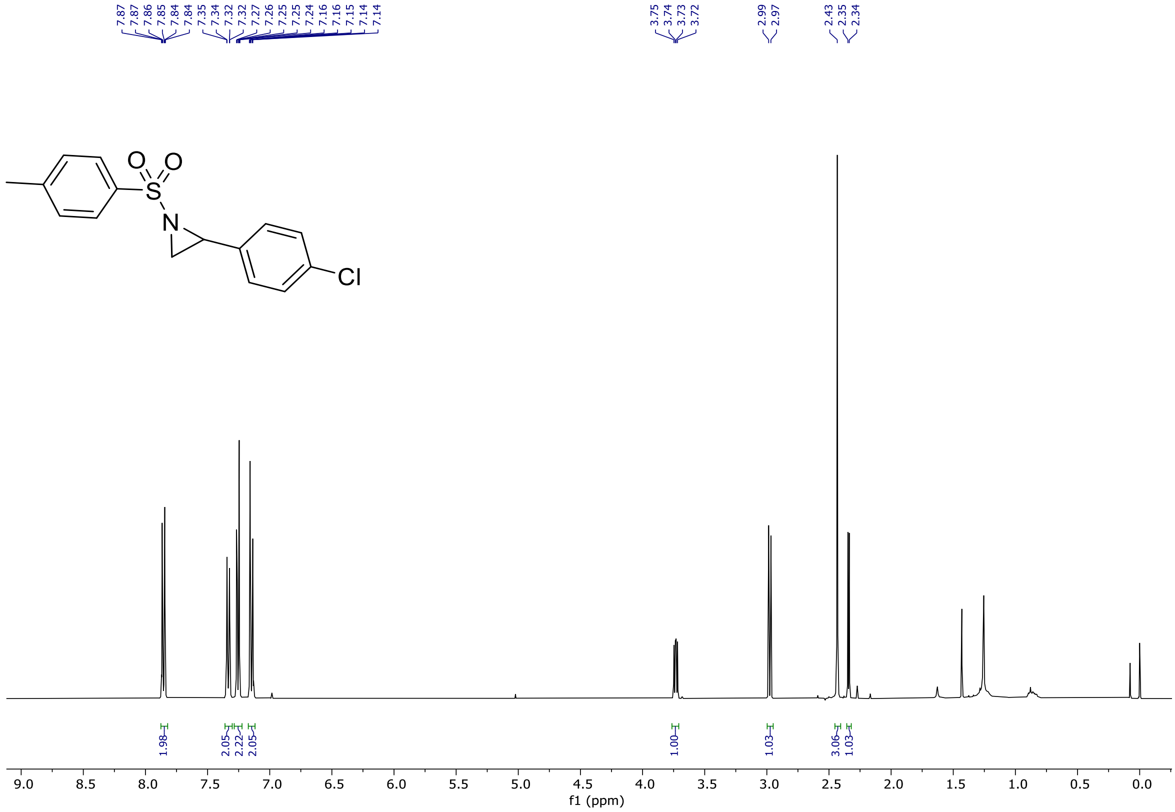

4k: ^{13}C{^{1}H}-APT NMR, 101 MHz in $CDCl_3$

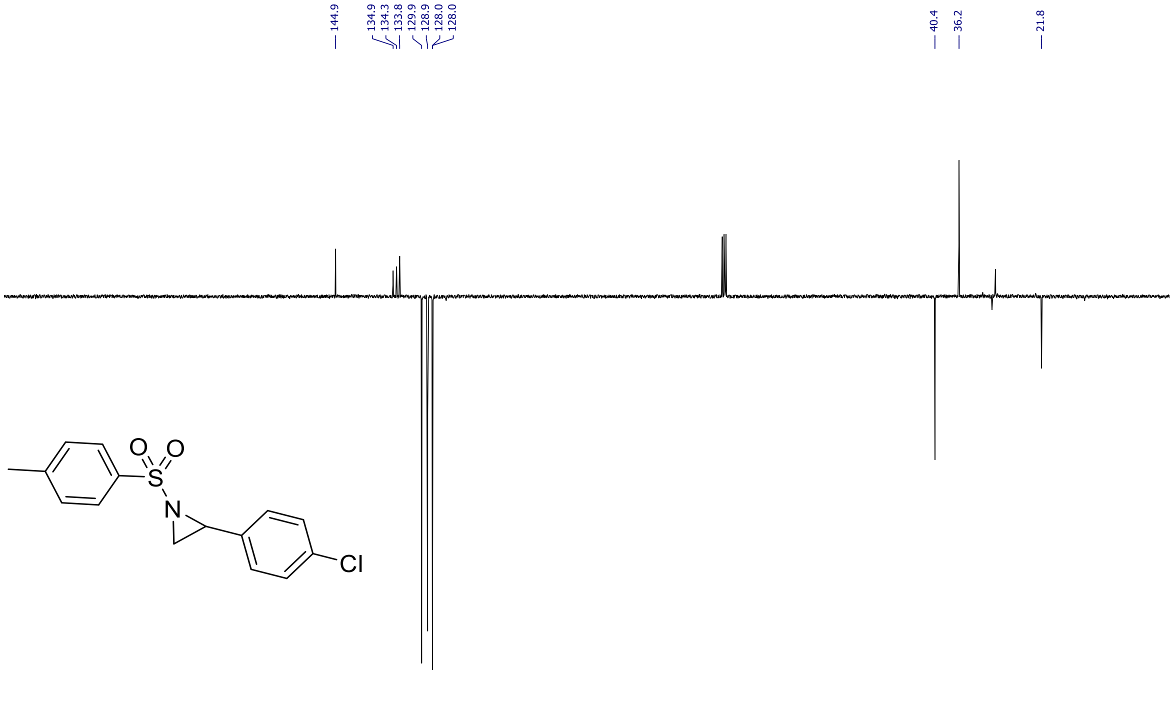

4k: ^{1}H-^{1}H COSY spectrum in $CDCl_3$

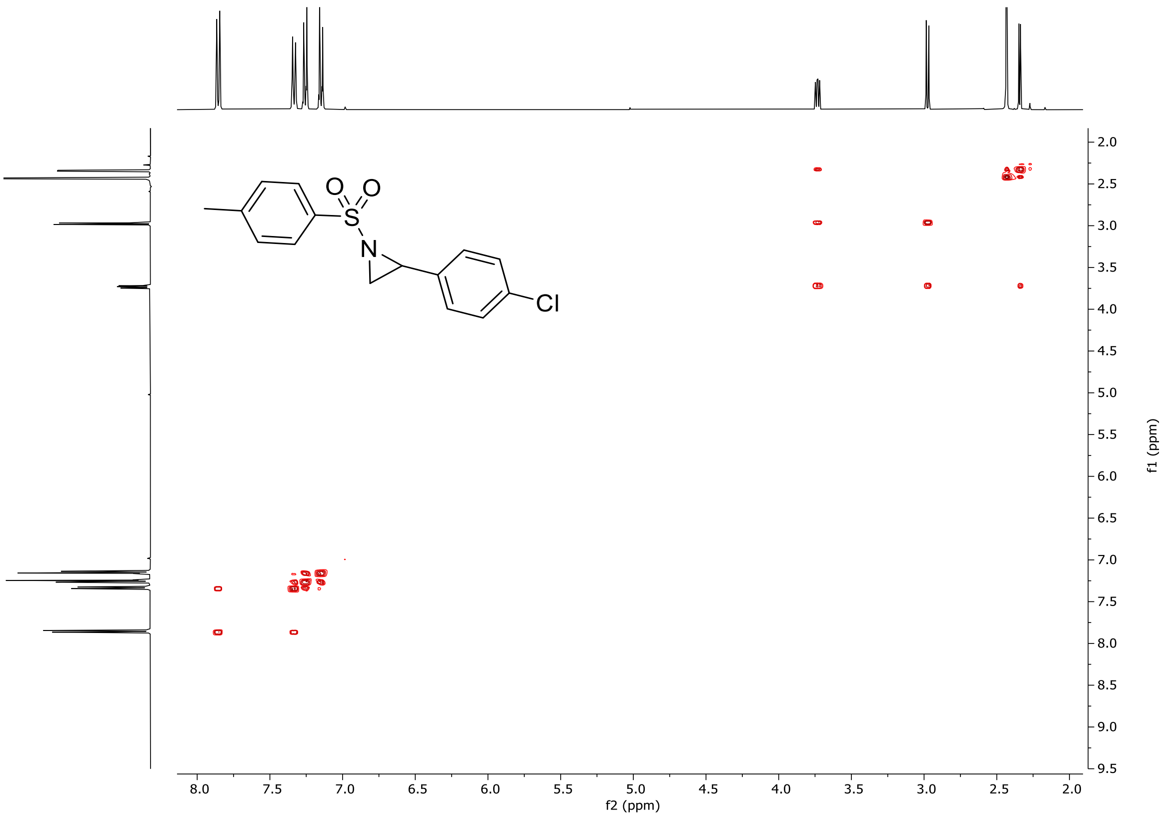

4k: ^{1}H-^{13}C HSQC spectrum in $CDCl_3$

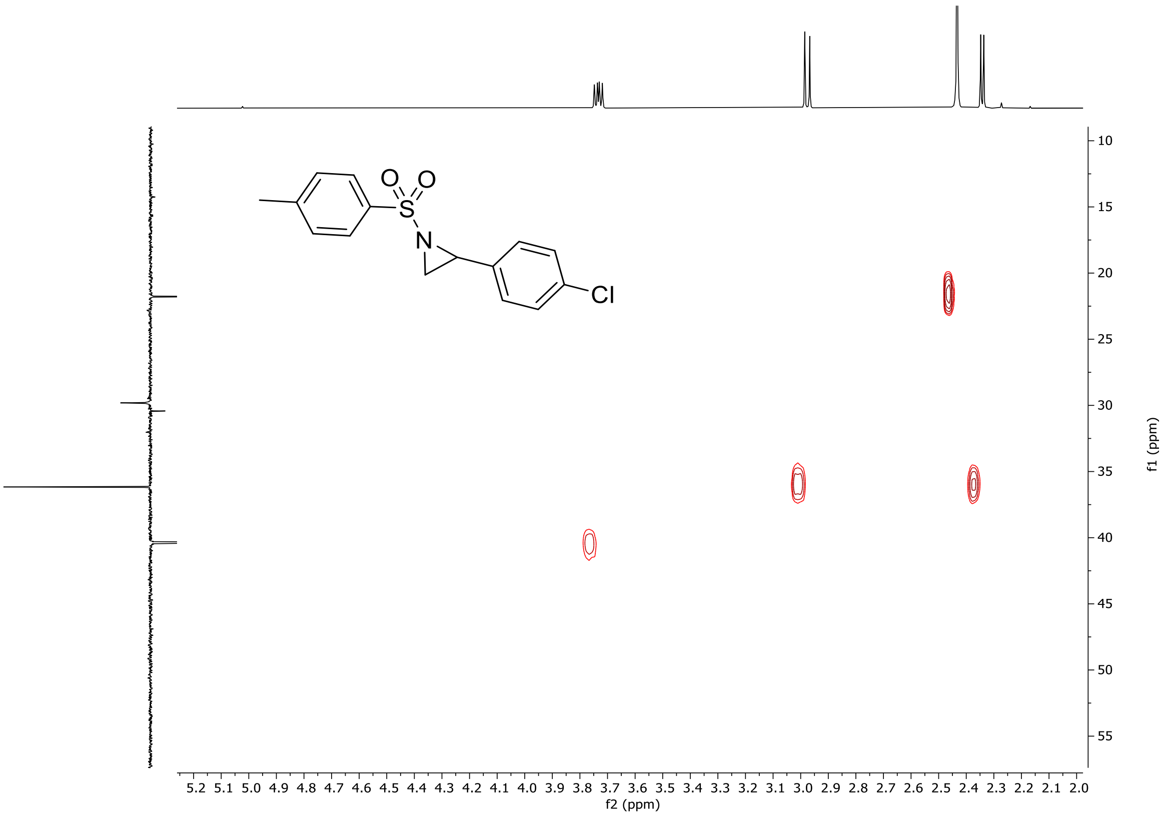

4l: ^{1}H NMR, 400 MHz in $CDCl_3$

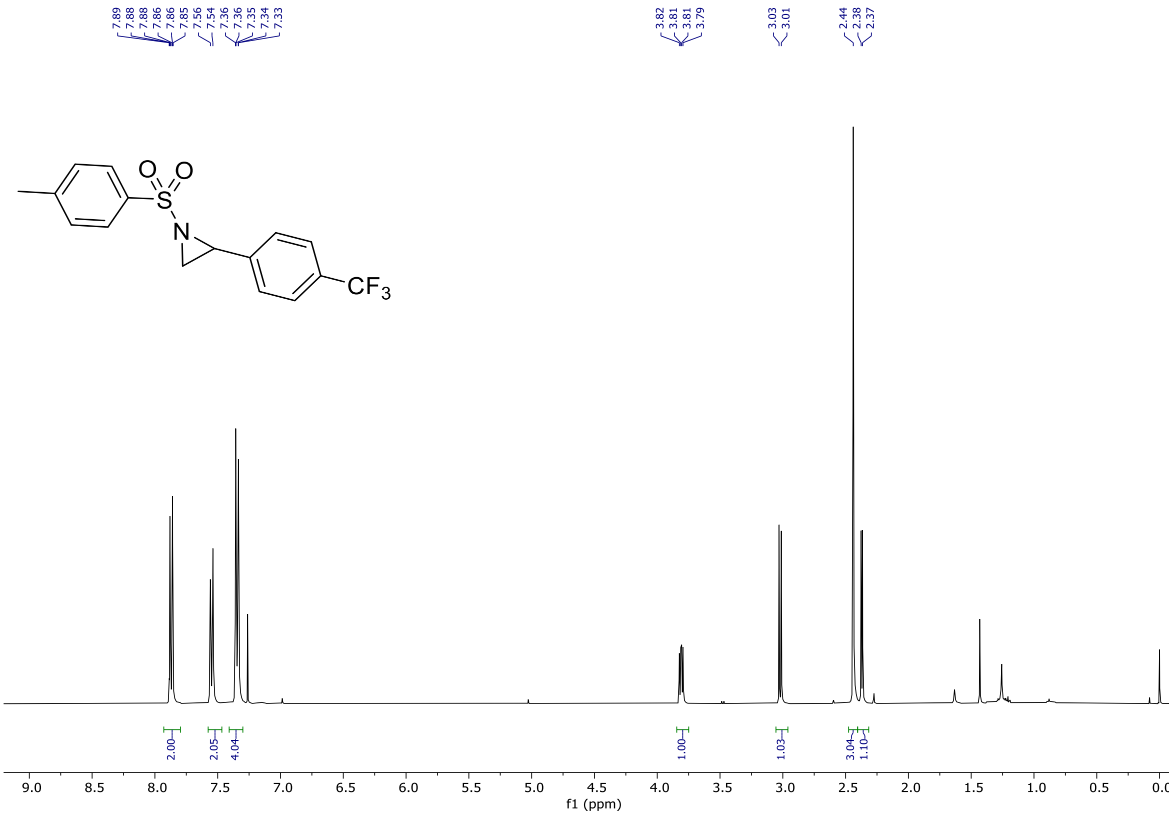

4l: $^{13}C\{^{1}H\}$-APT NMR, 101 MHz in $CDCl_3$

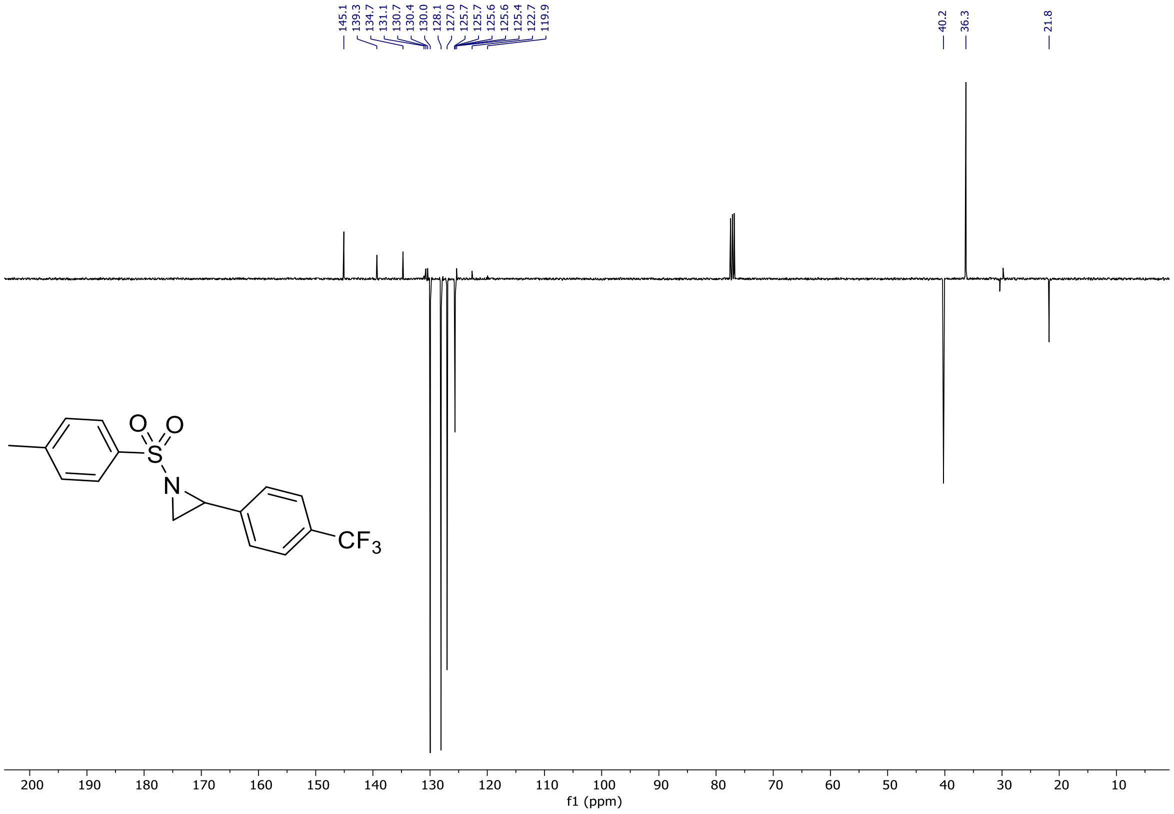

4l: ^{19}F NMR, 376 MHz in CDCl$_3$

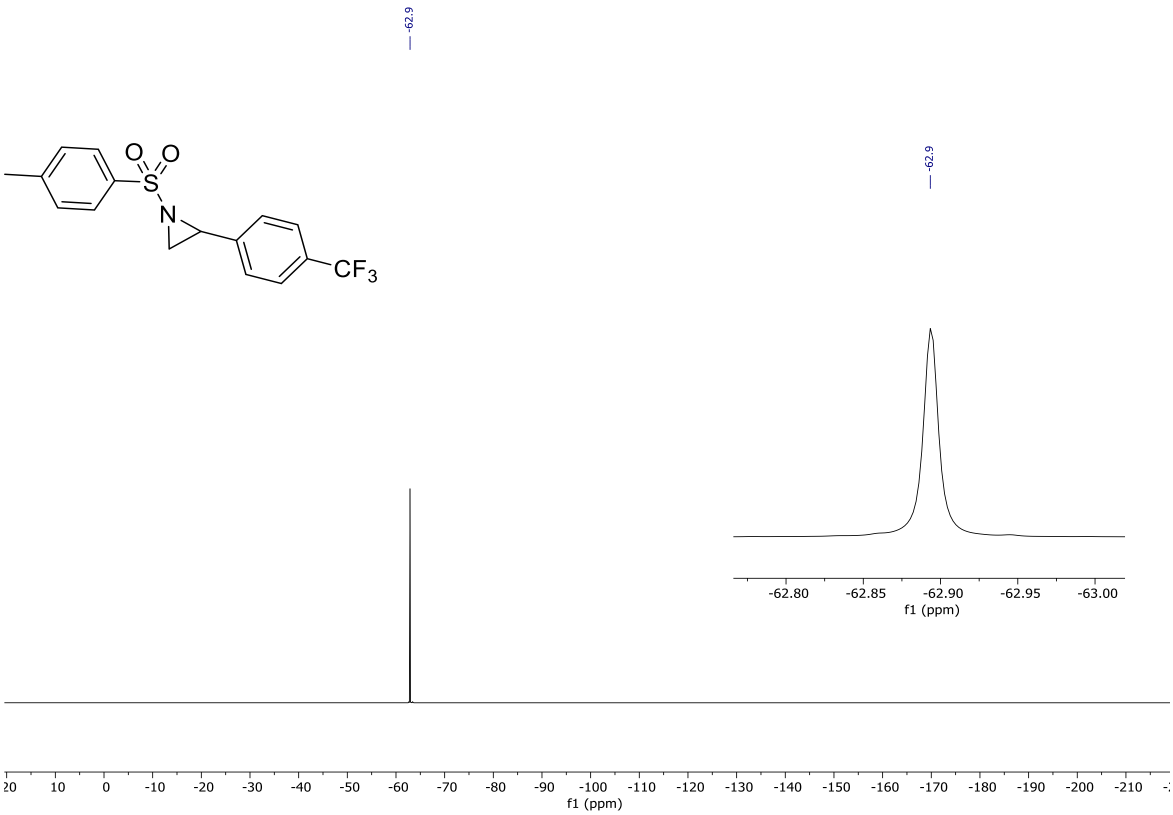

4l: ^{1}H-^{1}H COSY spectrum in CDCl$_3$

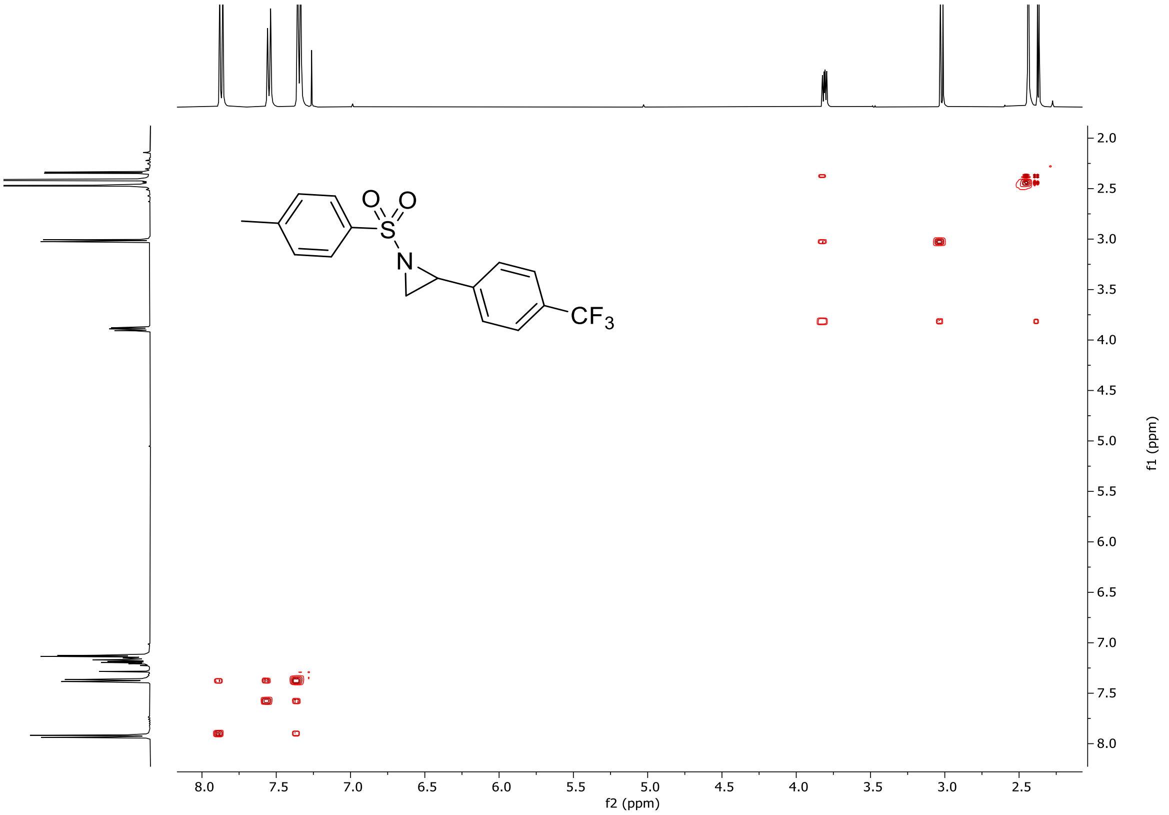

4l: ^{1}H-^{13}C HSQC spectrum in $CDCl_3$

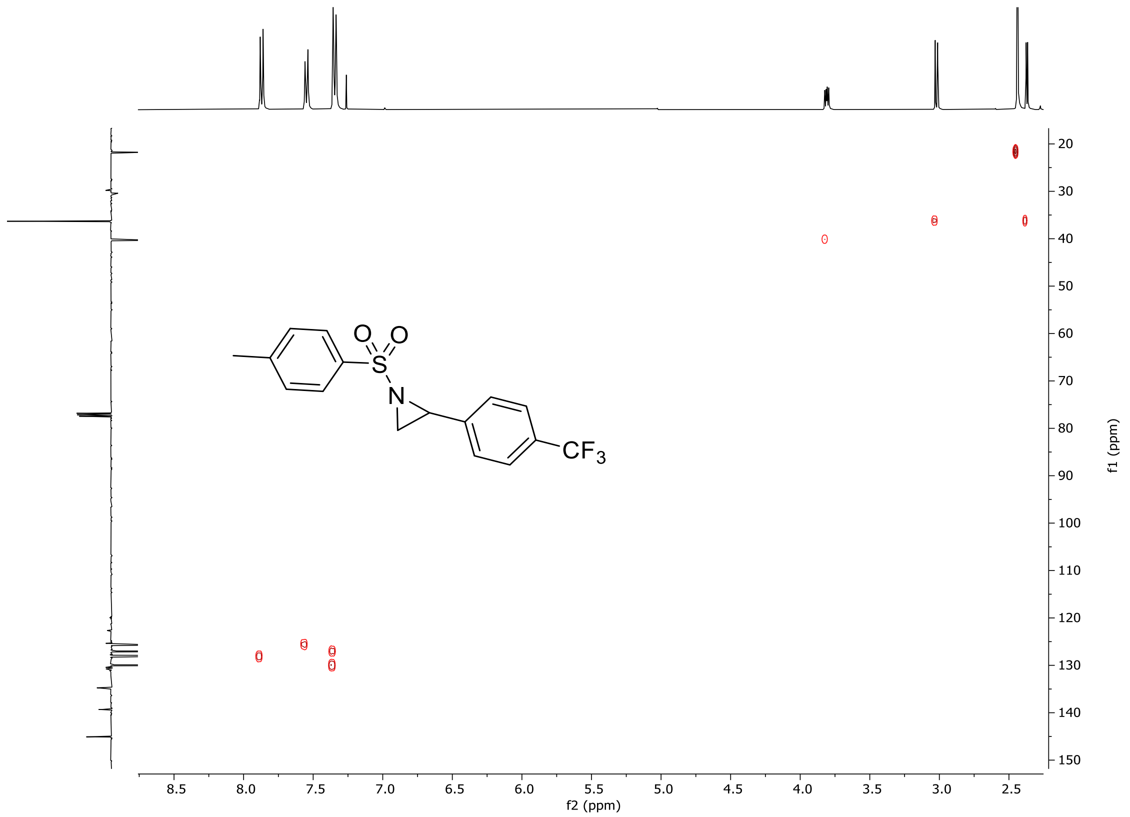

4m: ^{1}H NMR, 400 MHz in $CDCl_3$

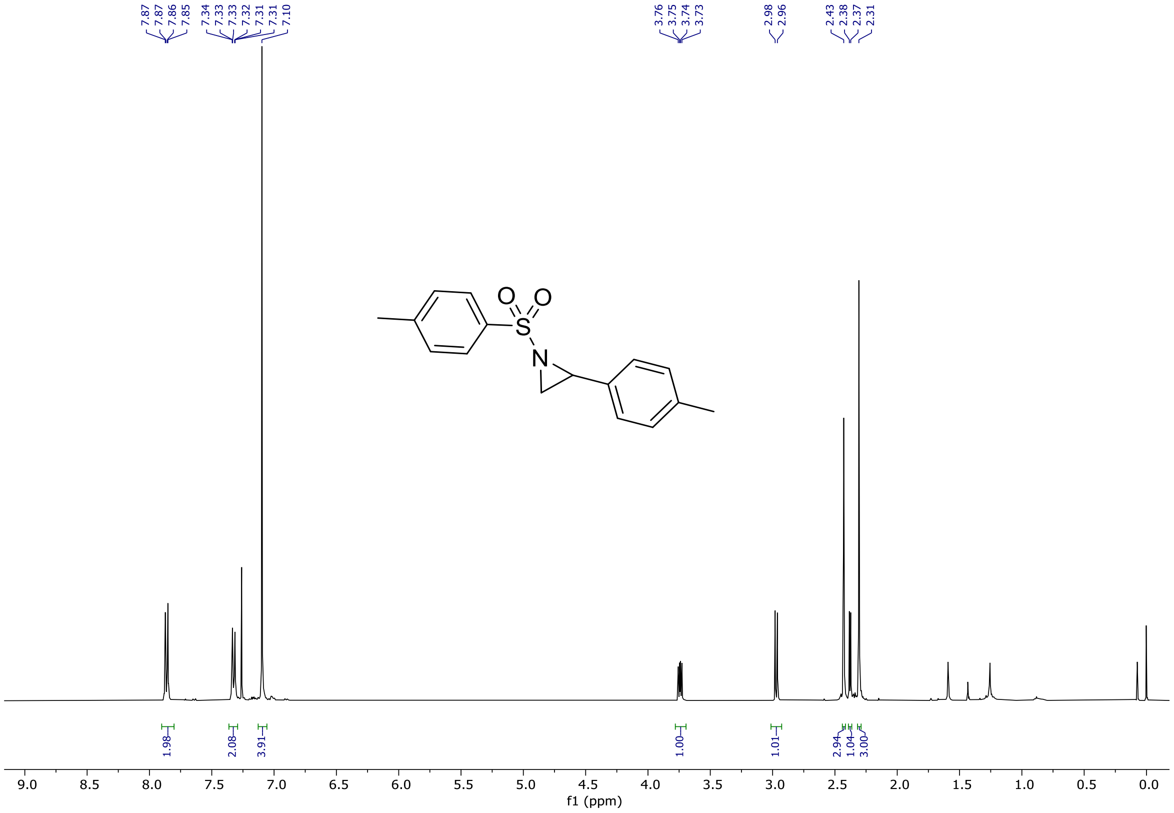

4m: $^{13}C\{^{1}H\}$-APT NMR, 101 MHz in $CDCl_3$

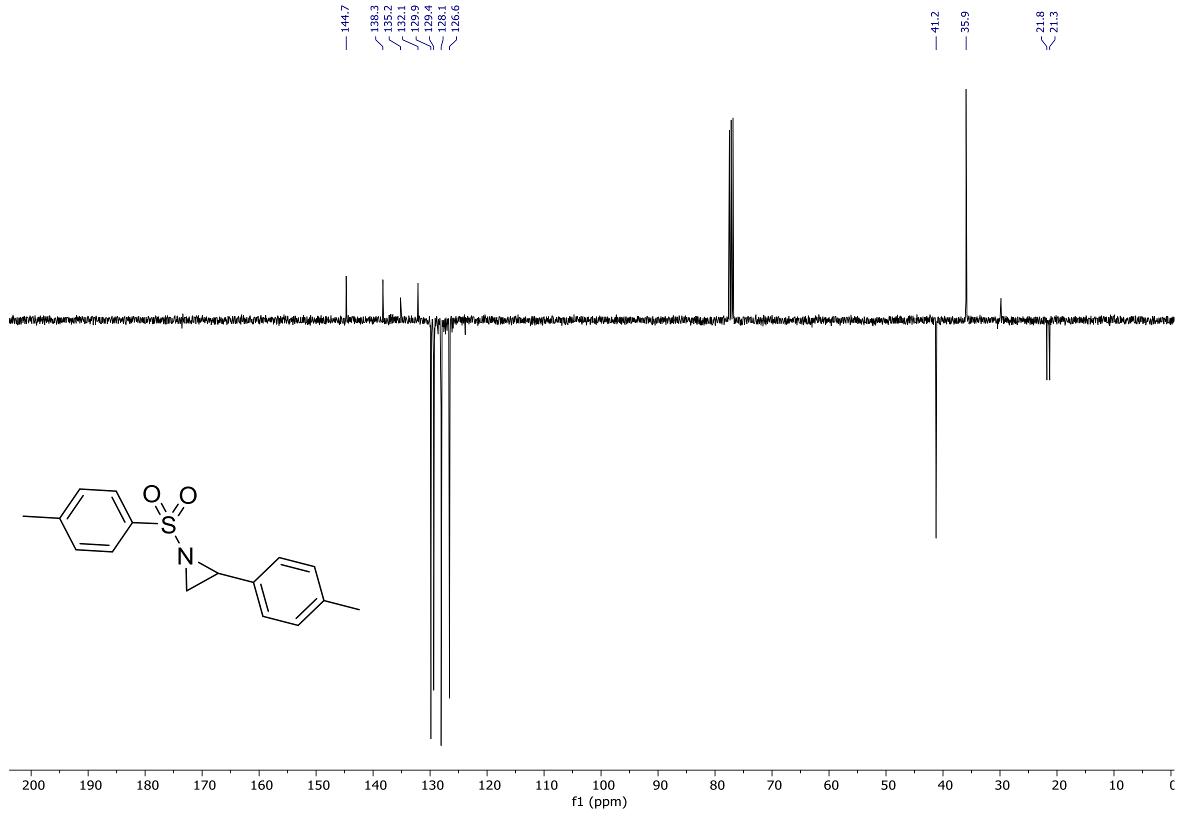

4m: ^{1}H-^{1}H COSY spectrum in $CDCl_3$

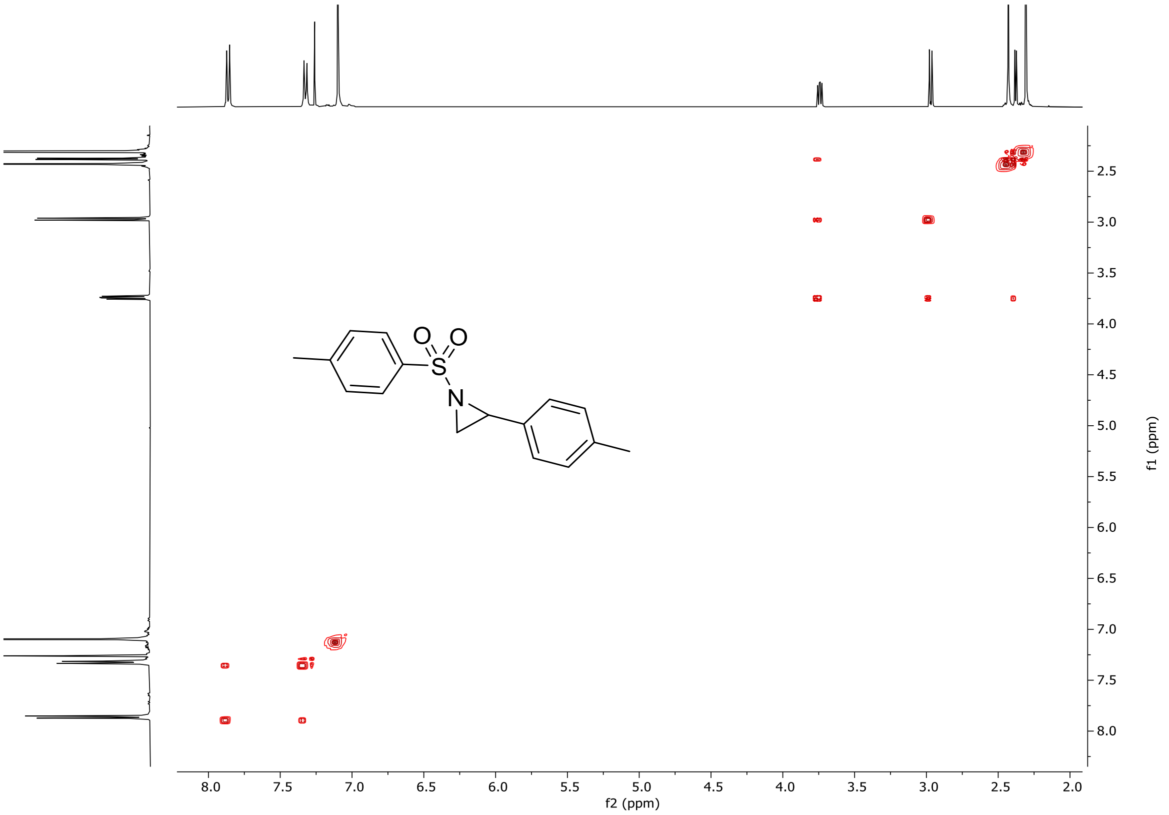

4m: ^{1}H-^{13}C HSQC spectrum in $CDCl_3$

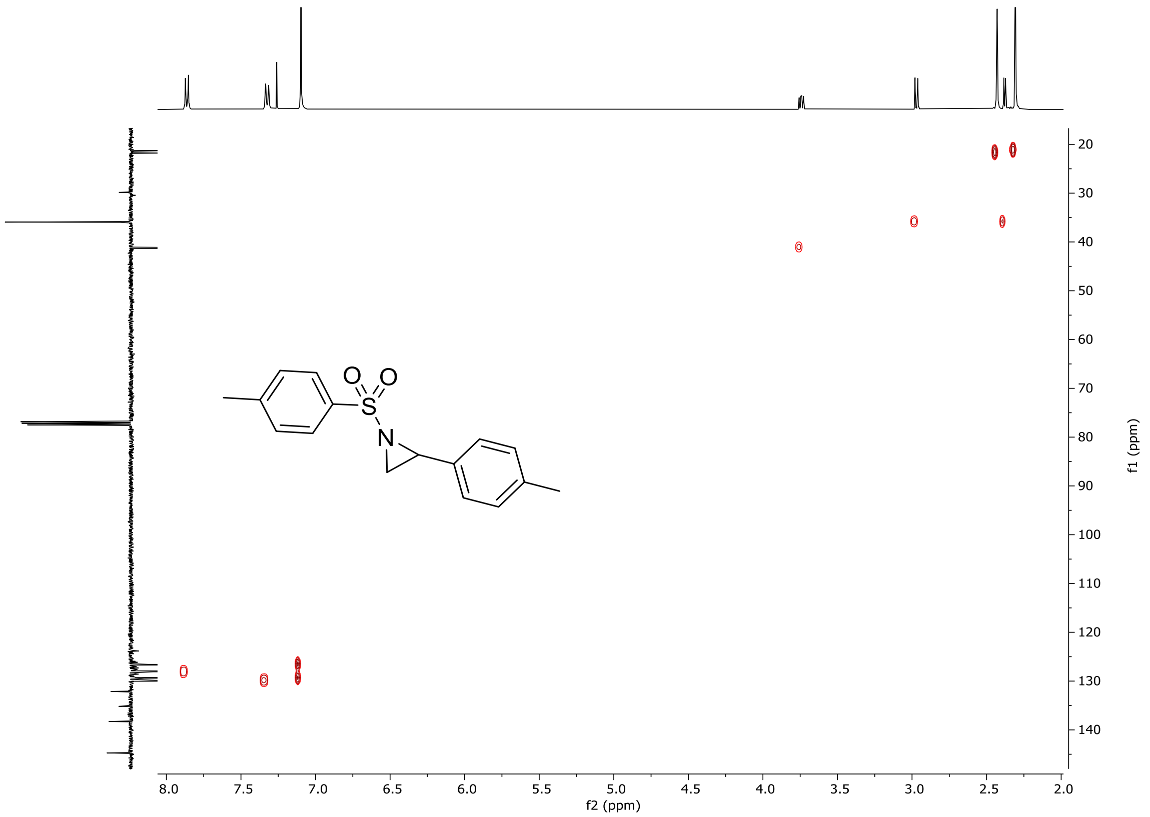

4n: ^{1}H NMR, 500 MHz in $CDCl_3$

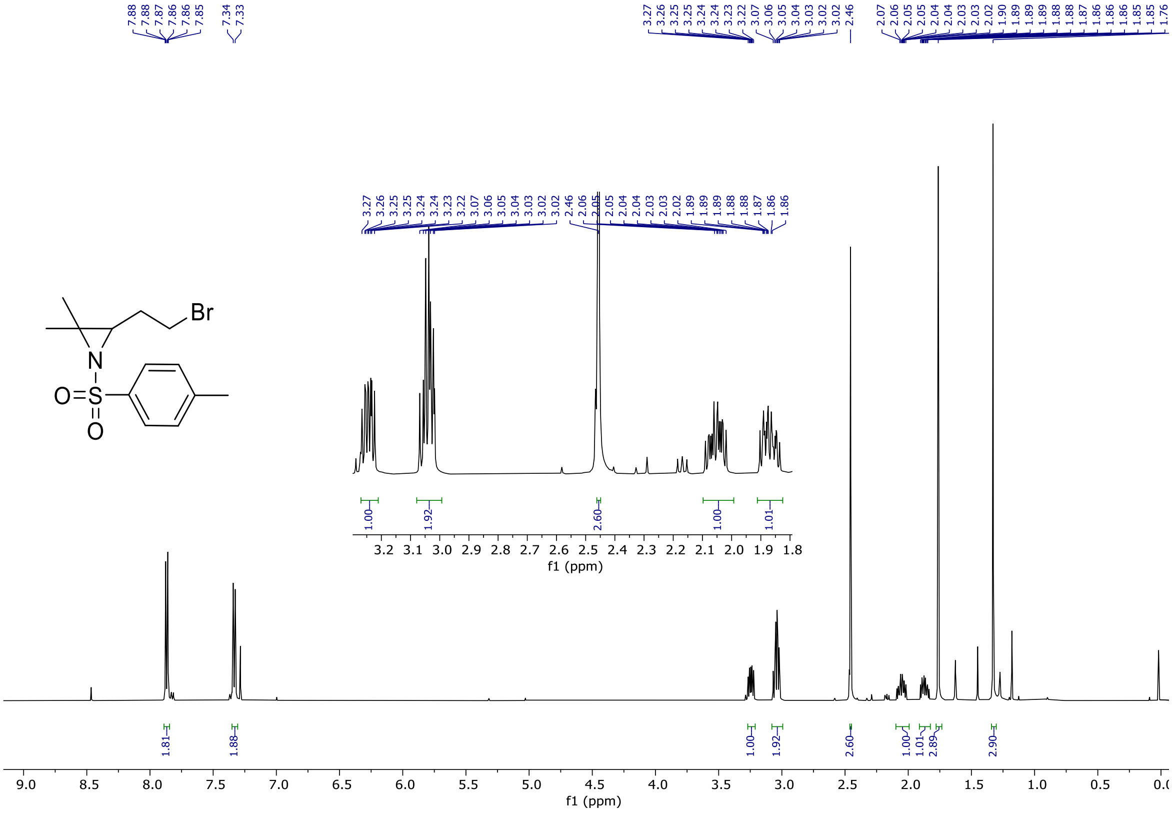

4n: $^{13}C\{^1H\}$-APT NMR, 126 MHz in $CDCl_3$

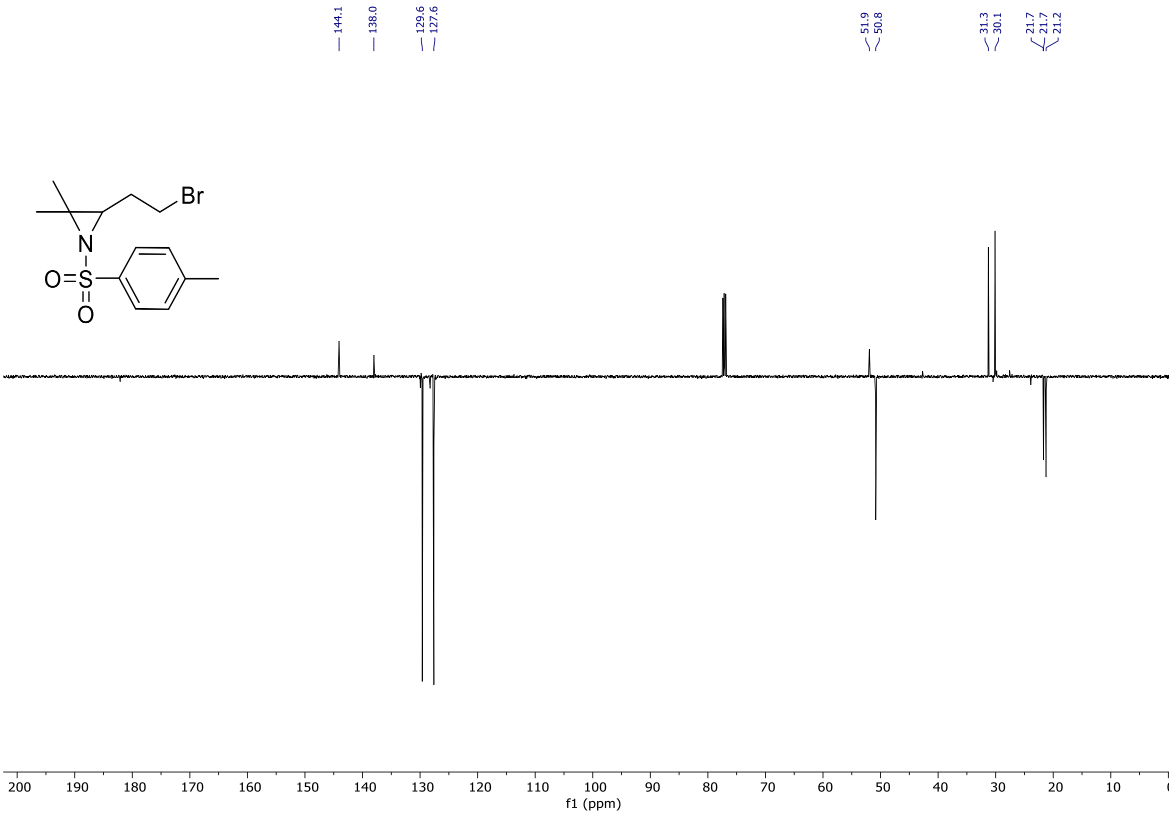

4n: ^1H-^1H COSY spectrum in $CDCl_3$

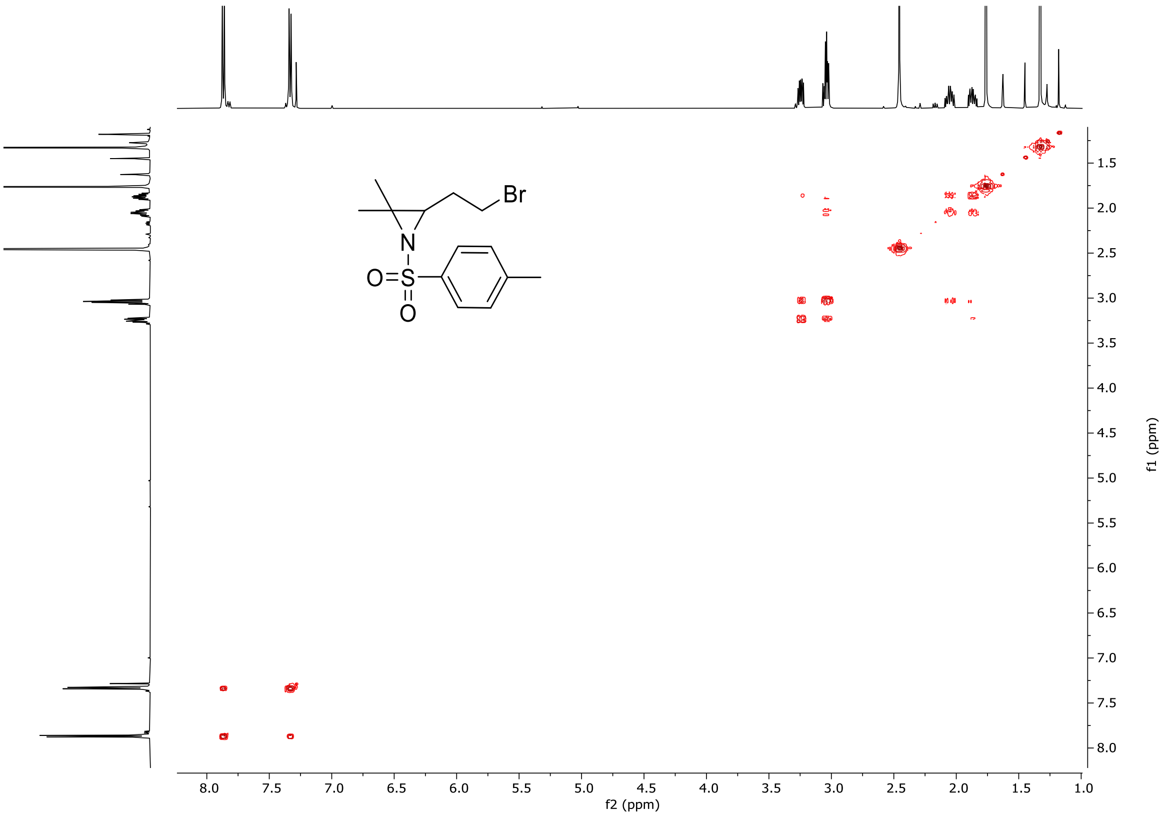

4n: ^{1}H-^{13}C HSQC spectrum in $CDCl_3$

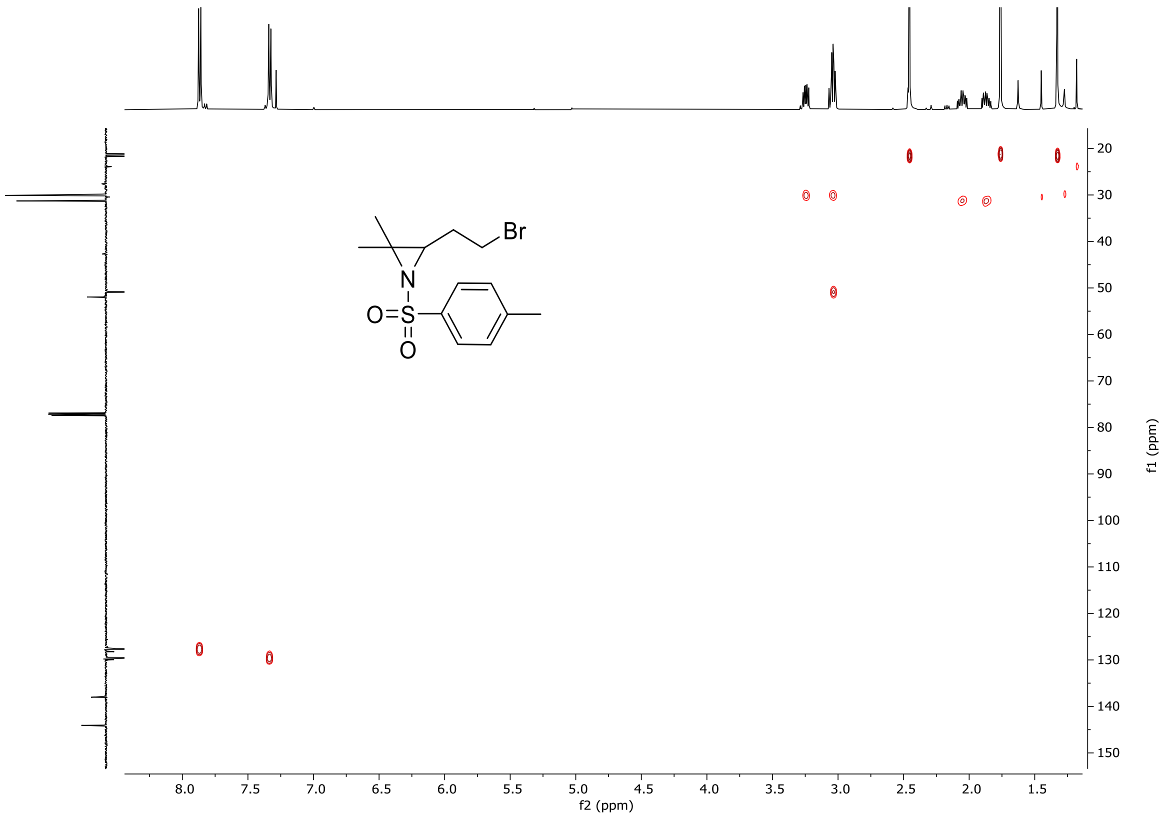

4o: ^{1}H NMR, 400 MHz in $CDCl_3$

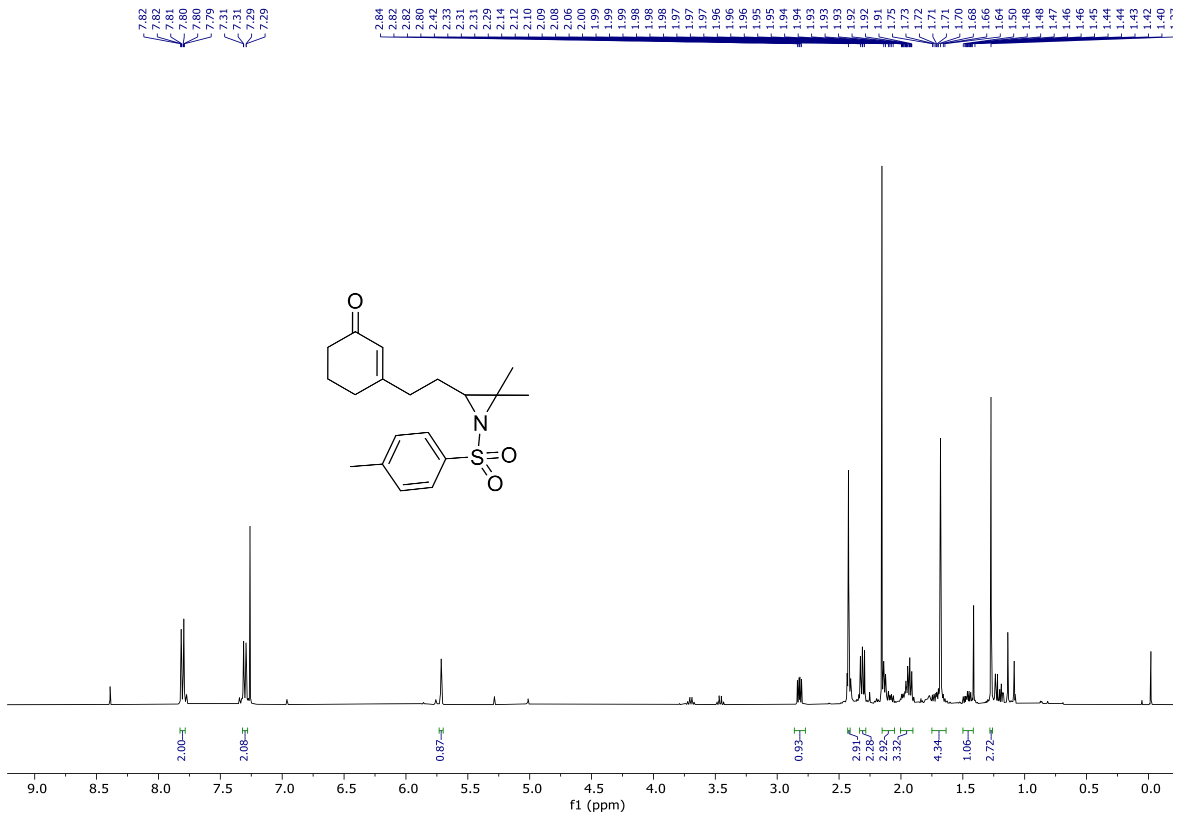

4o: $^{13}C\{^{1}H\}$-APT NMR, 101 MHz in $CDCl_3$

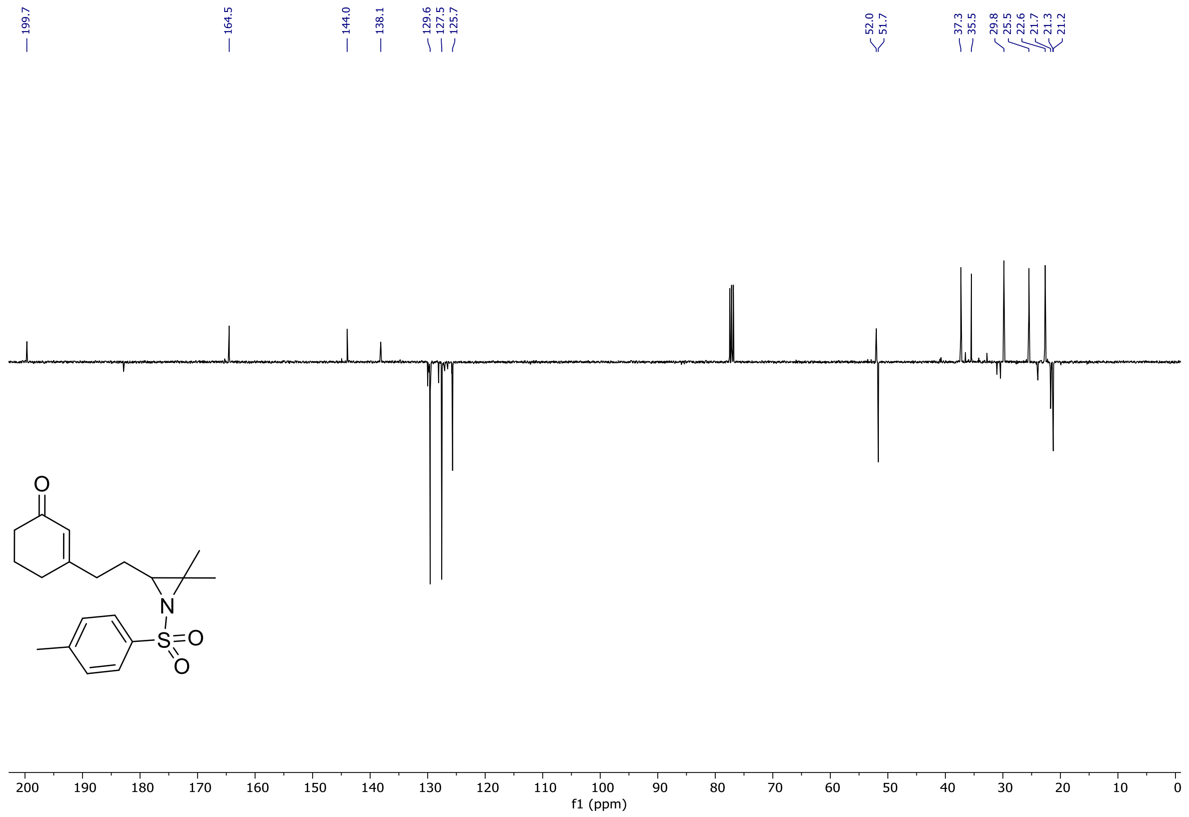

4o: ^{1}H-^{1}H COSY spectrum in $CDCl_3$

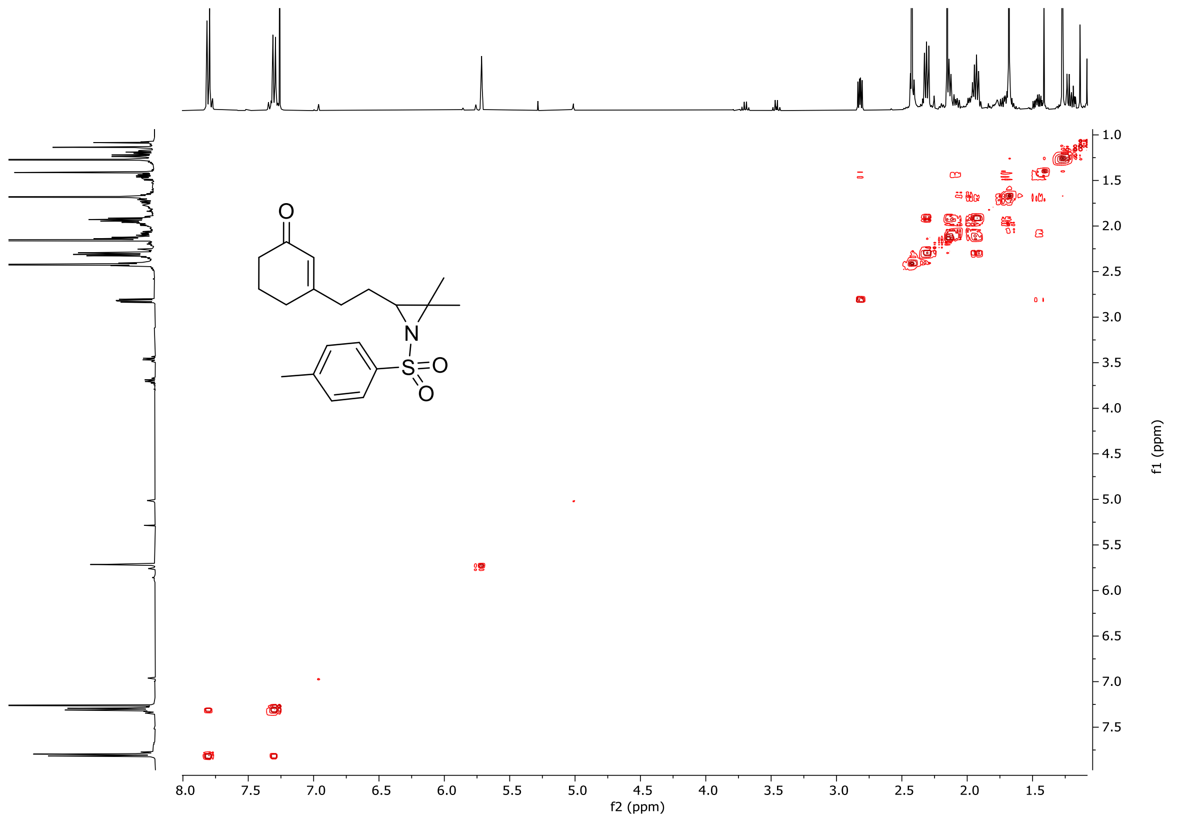

4o: ^{1}H-^{13}C HSQC spectrum in $CDCl_3$

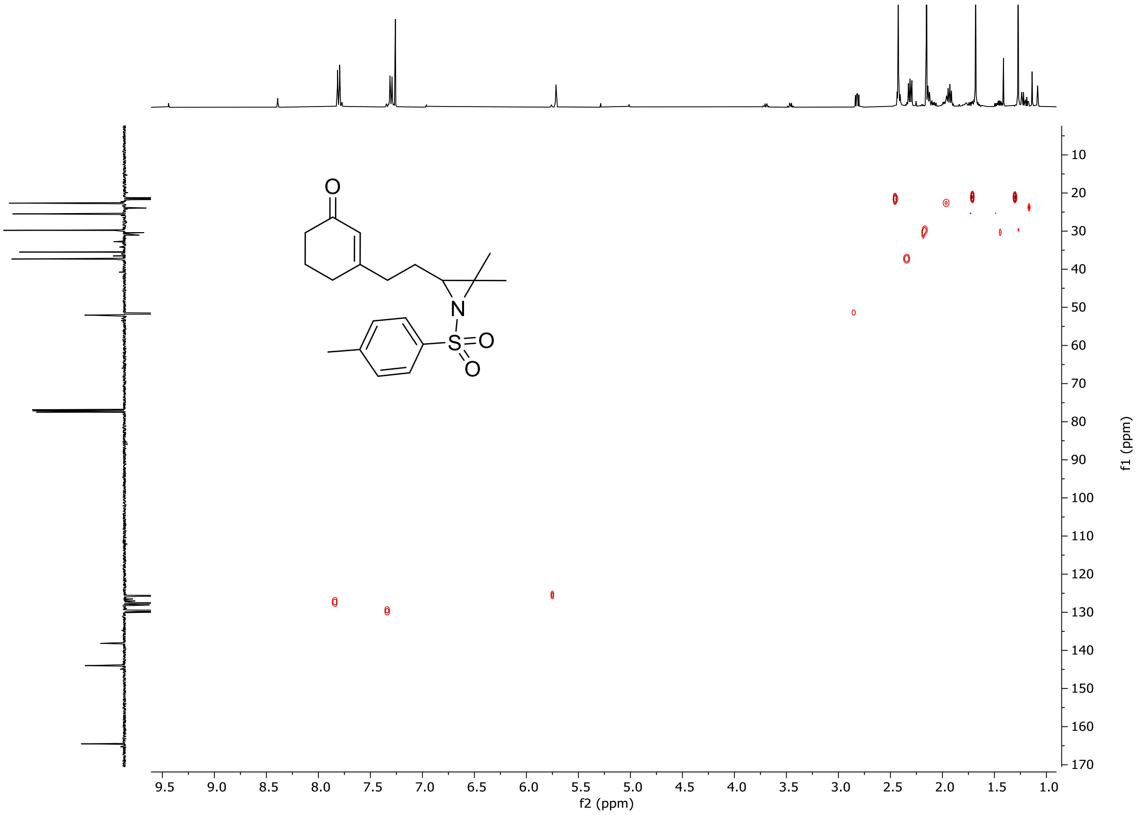

4p: ^{1}H NMR, 400 MHz in $CDCl_3$

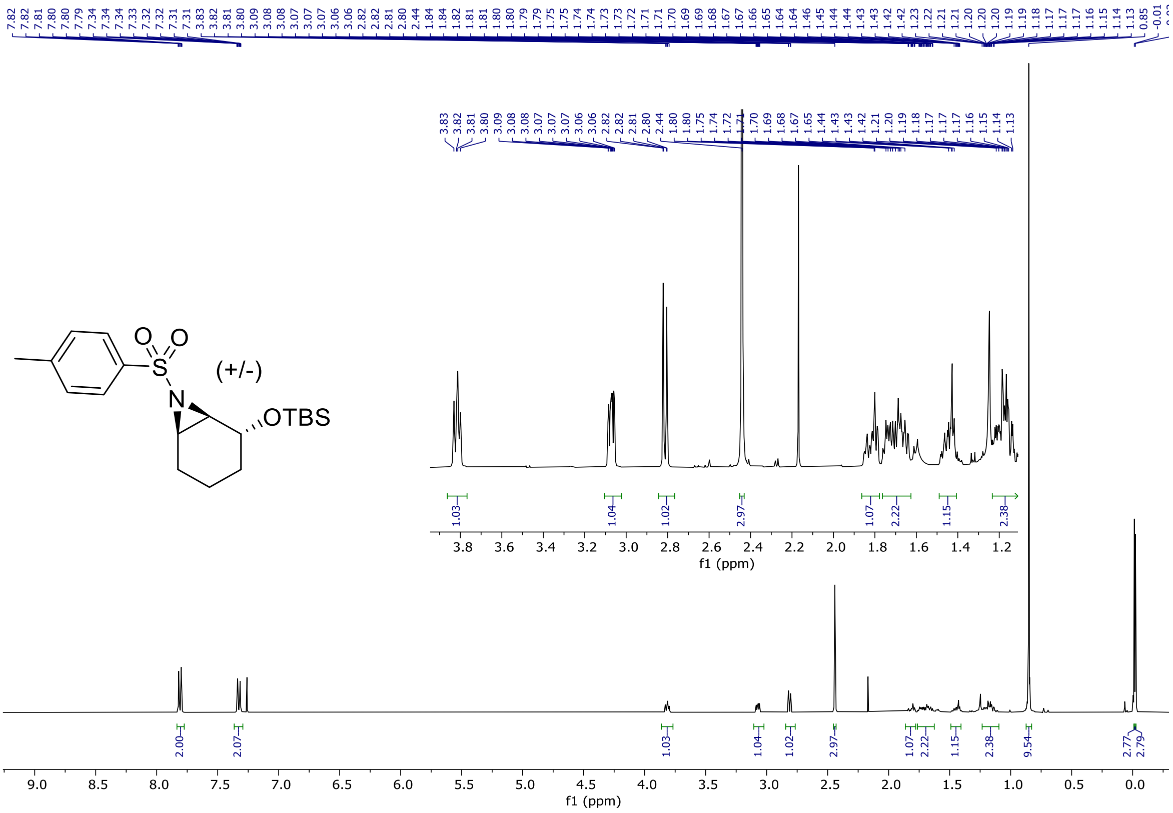

4p: $^{13}C\{^{1}H\}$-APT NMR, 101 MHz in $CDCl_3$

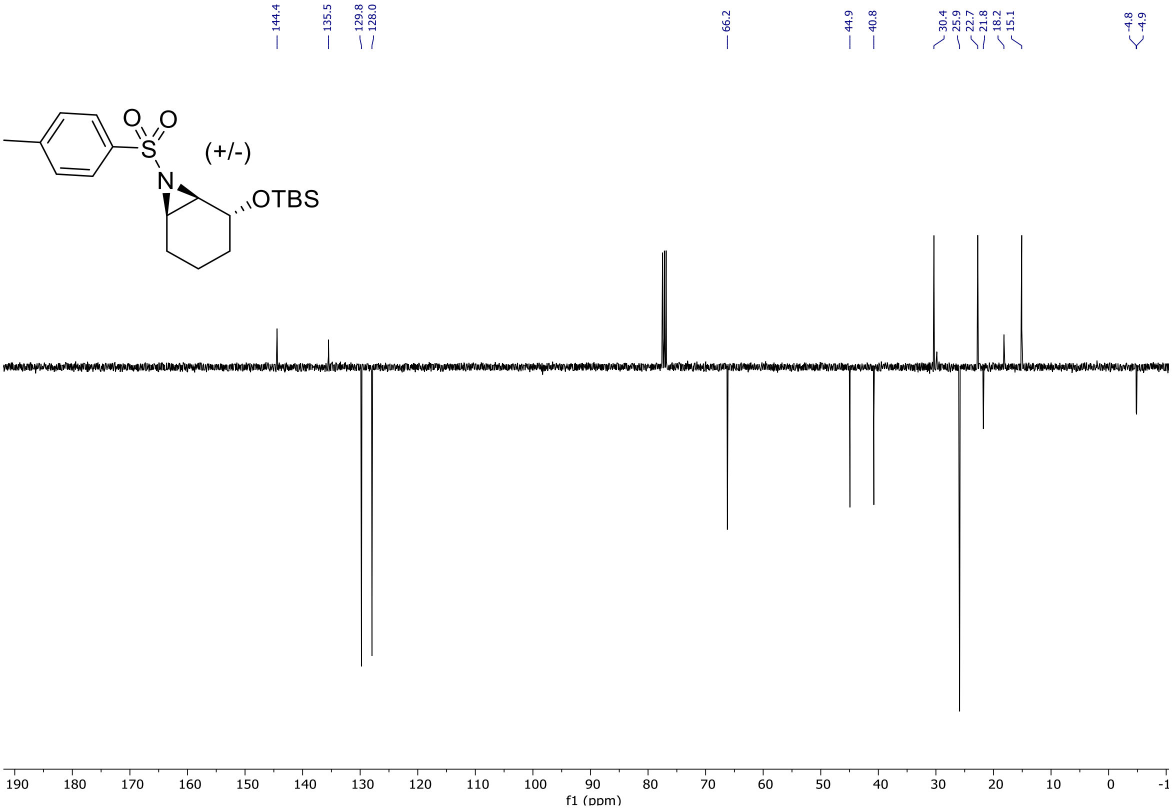

4p: ^{1}H-^{1}H COSY spectrum in $CDCl_3$

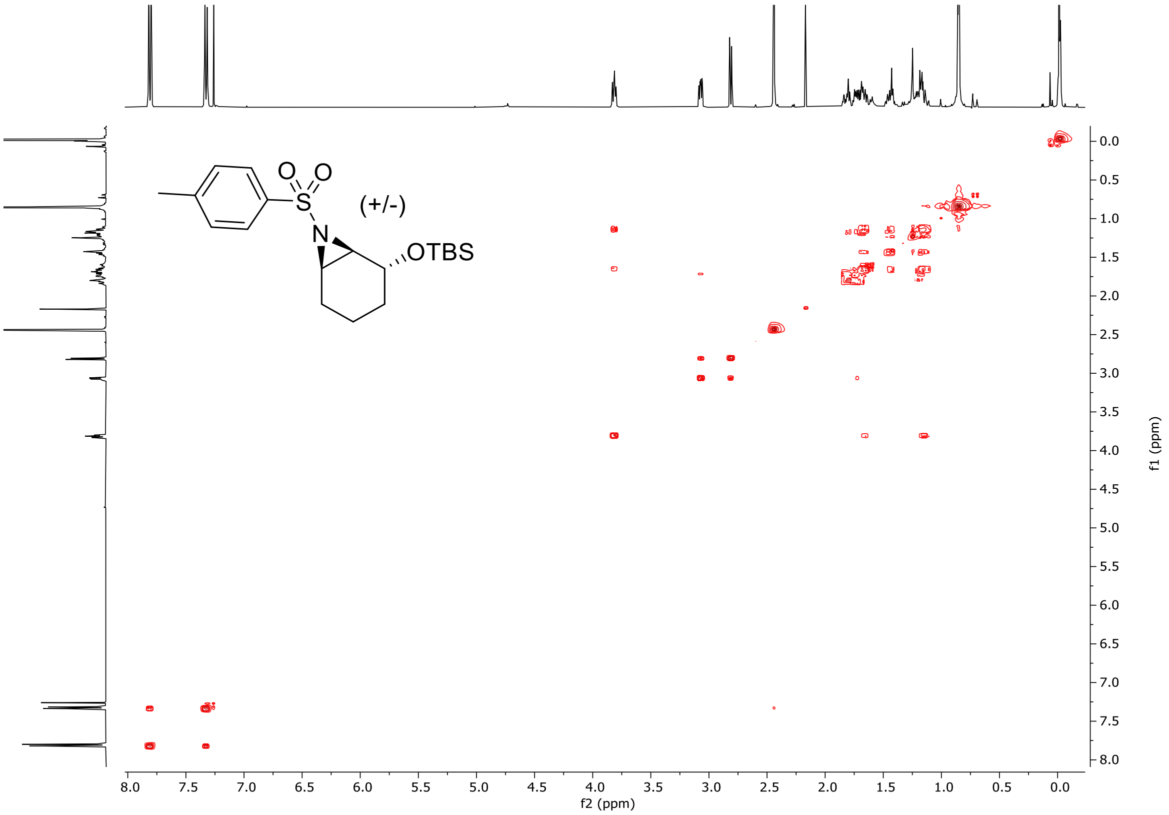

4p: ^{1}H-^{13}C HSQC spectrum in $CDCl_3$

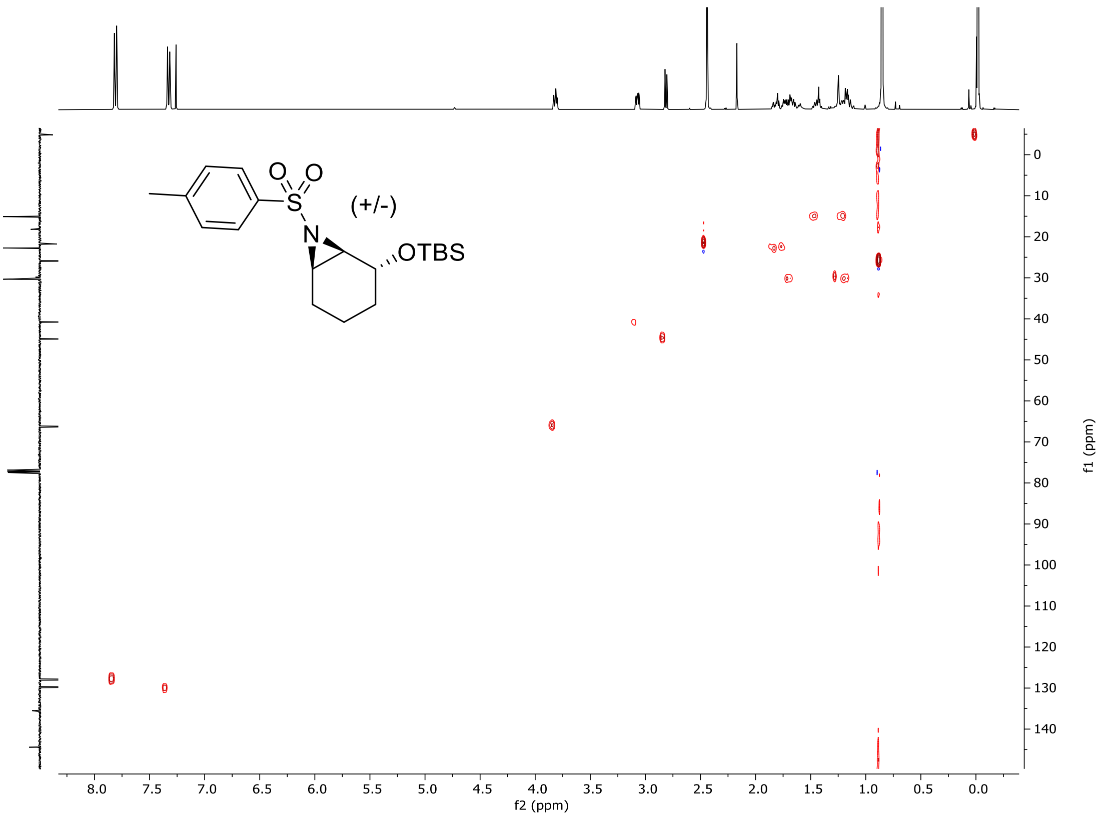

4q: ^{1}H NMR, 400 MHz in $CDCl_3$

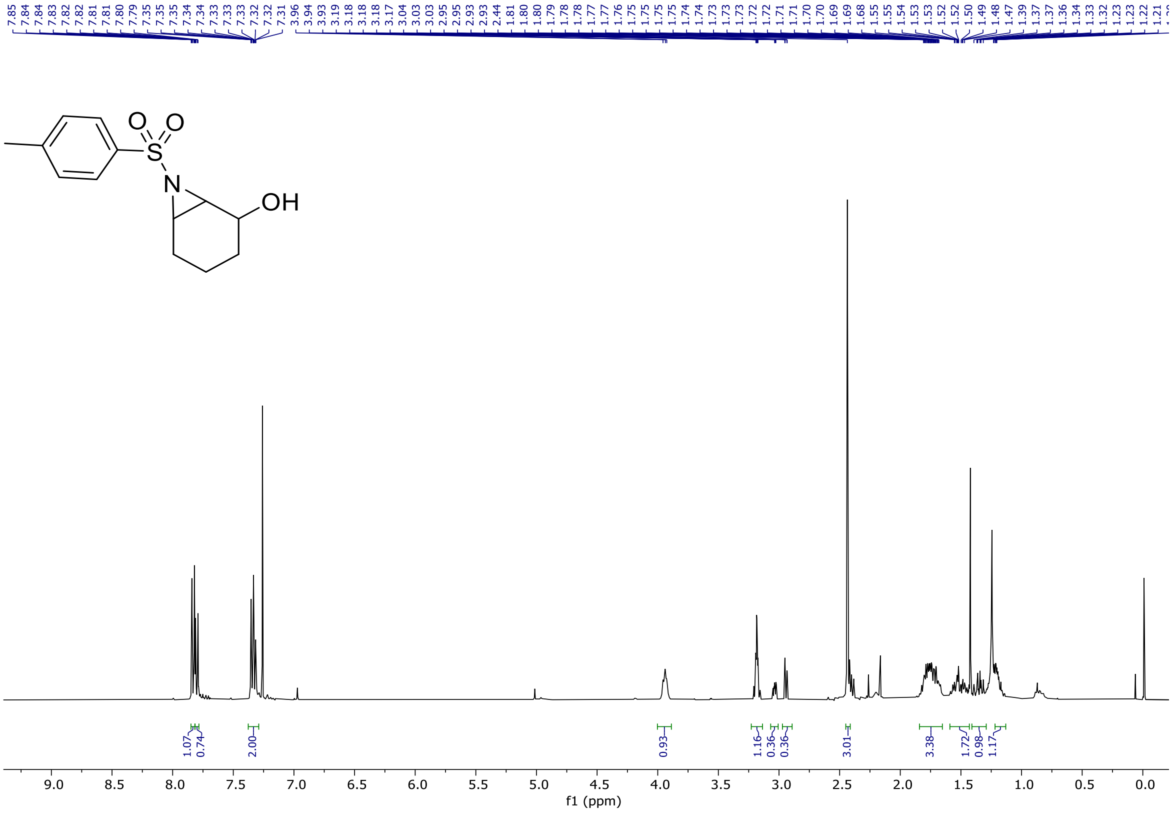

4q: $^{13}C\{^{1}H\}$-APT NMR, 101 MHz in $CDCl_3$

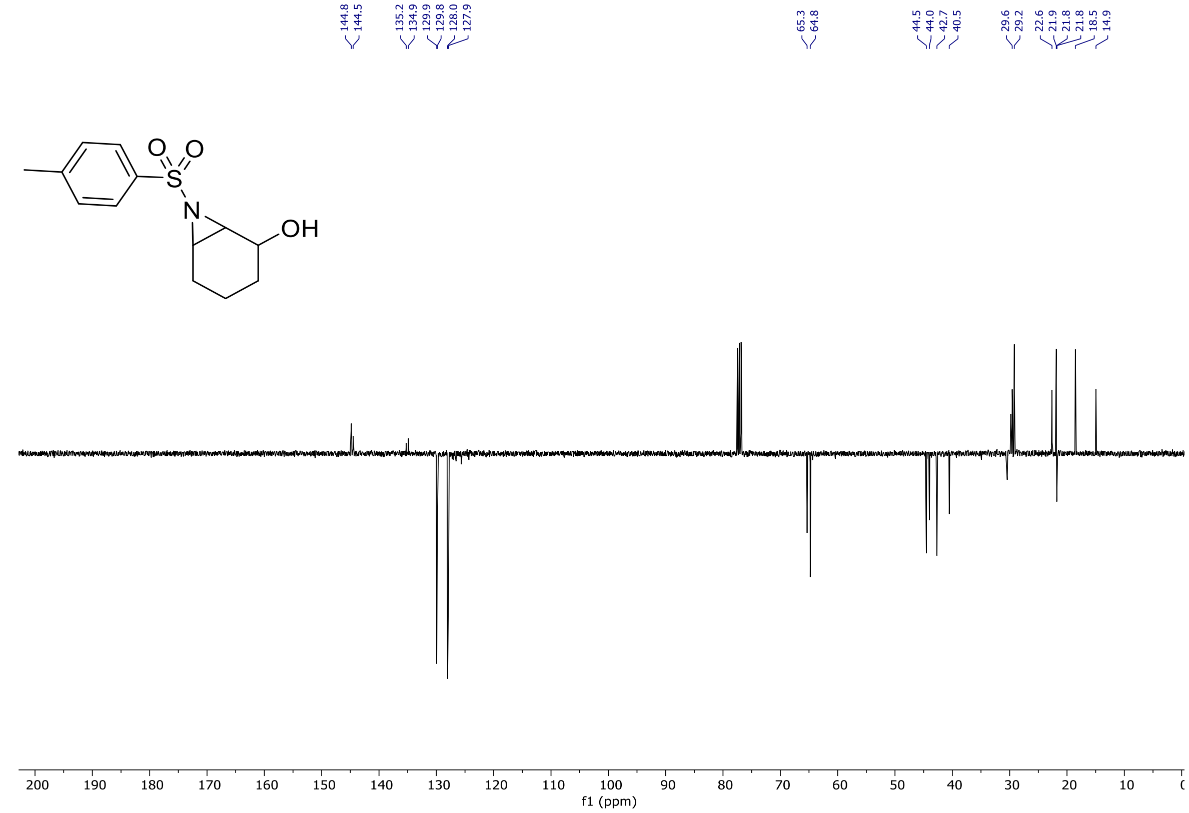

4q: ^{1}H-^{1}H COSY spectrum in $CDCl_3$

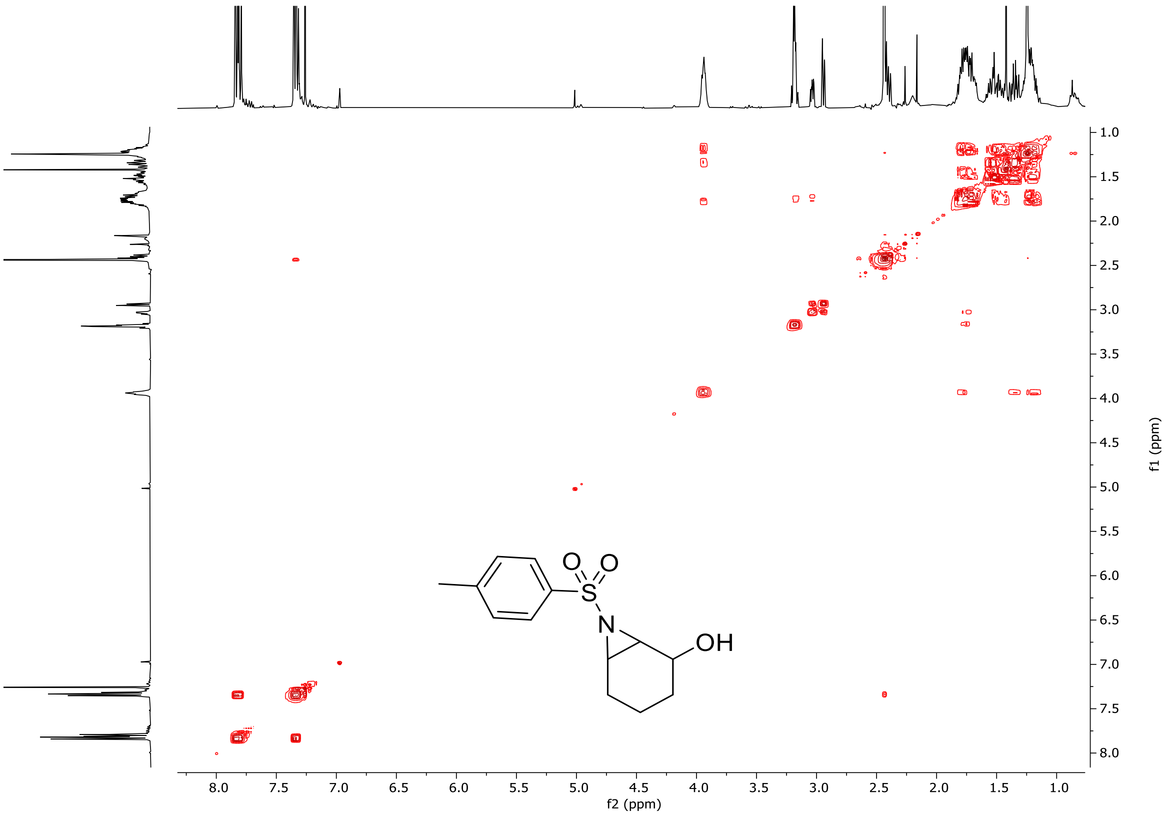

4q: ^1H-^{13}C HSQC spectrum in $CDCl_3$

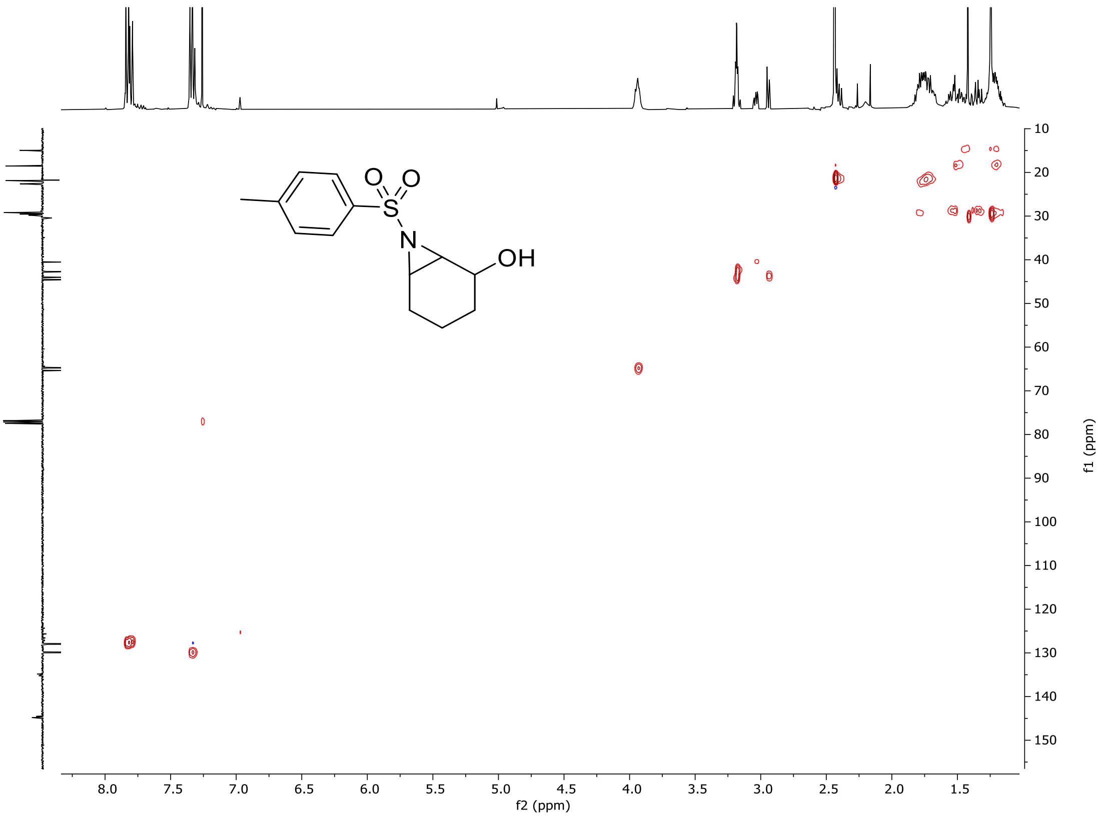

4r: ^1H NMR, 400 MHz in $CDCl_3$

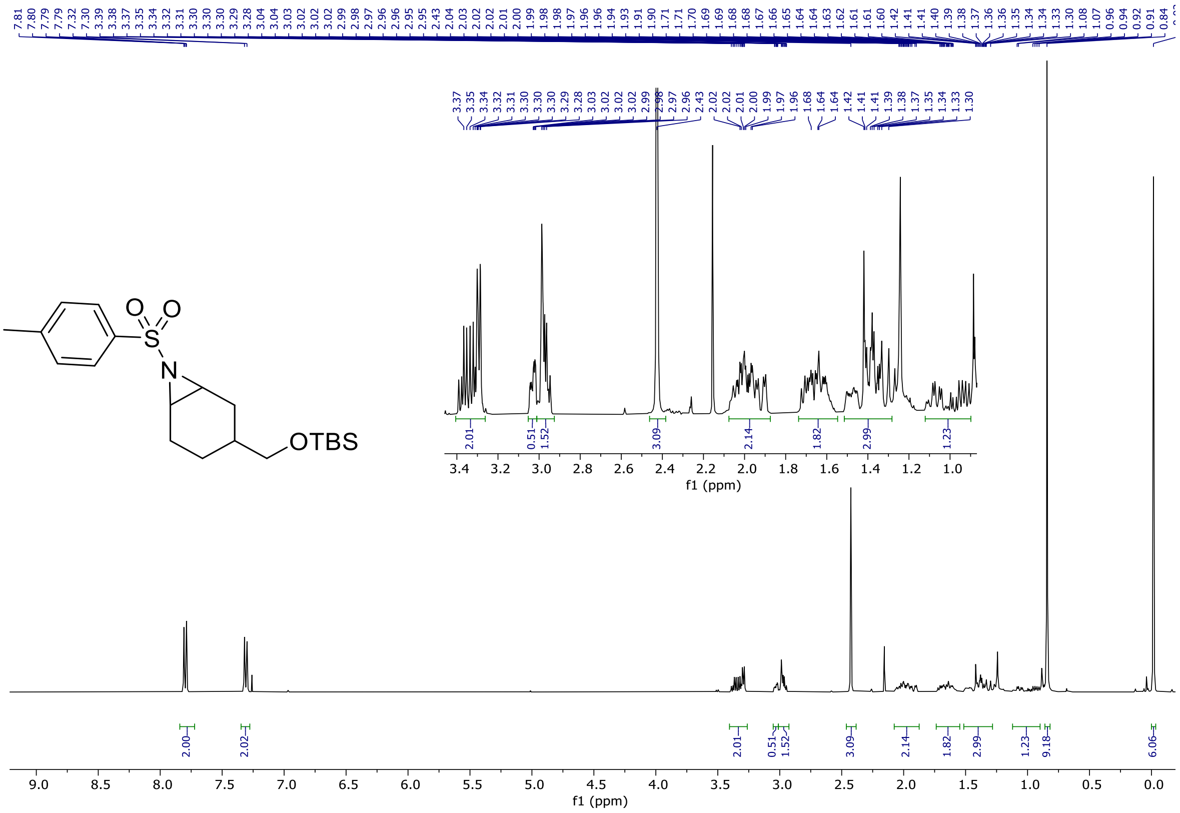

4r: $^{13}C\{^{1}H\}$-APT NMR, 101 MHz in $CDCl_3$

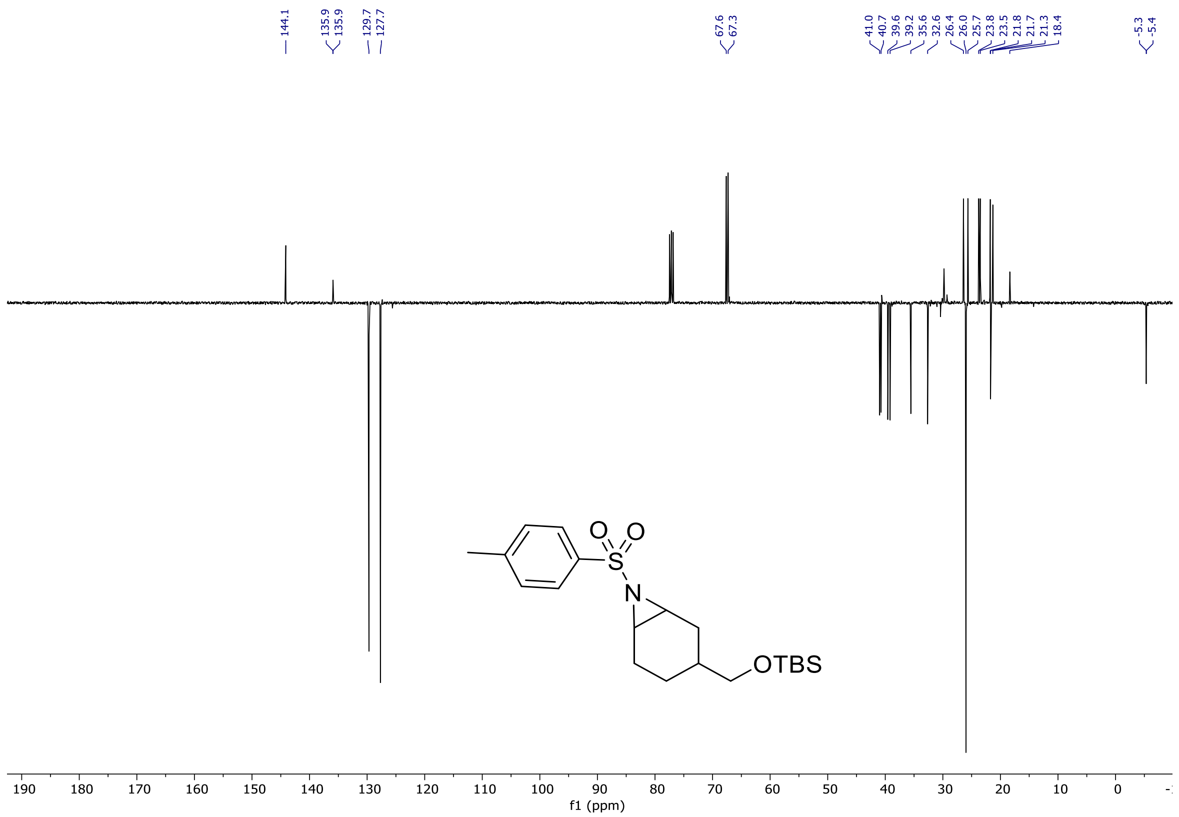

4r: ^{1}H-^{1}H COSY spectrum in $CDCl_3$

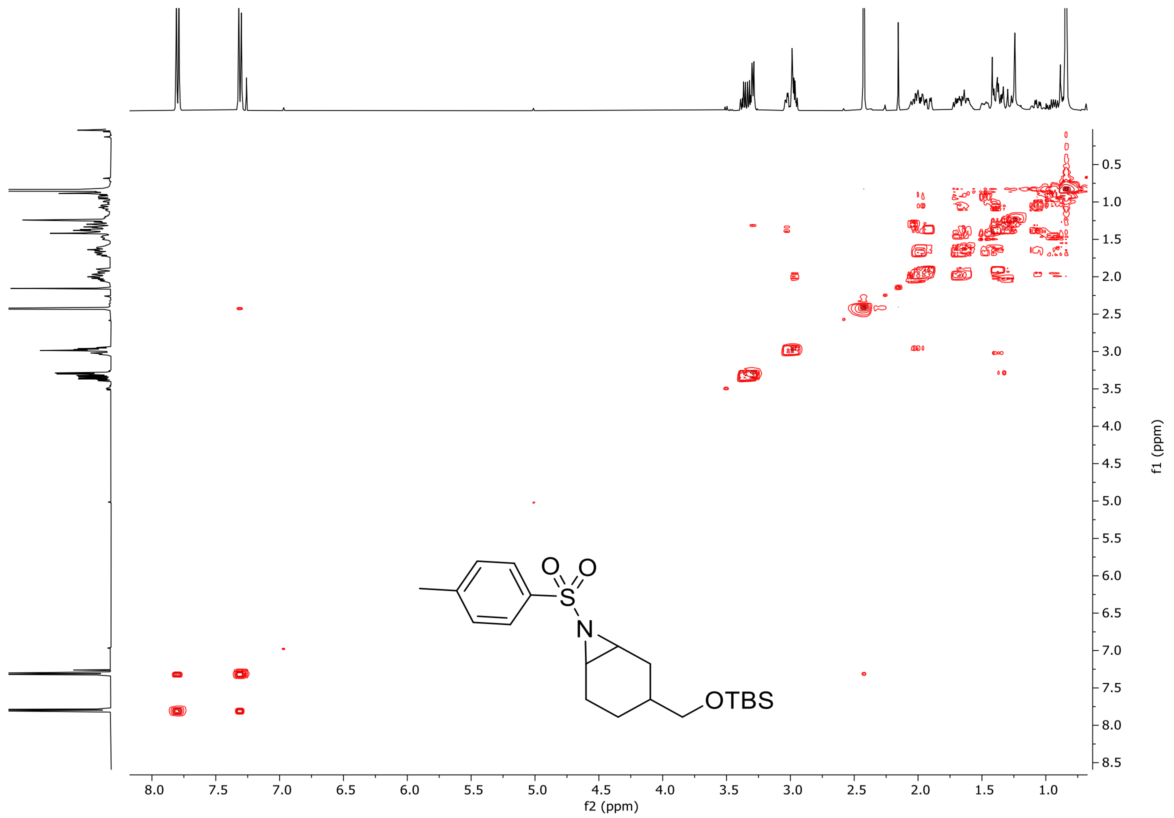

4r: ^{1}H-^{13}C HSQC spectrum in $CDCl_3$

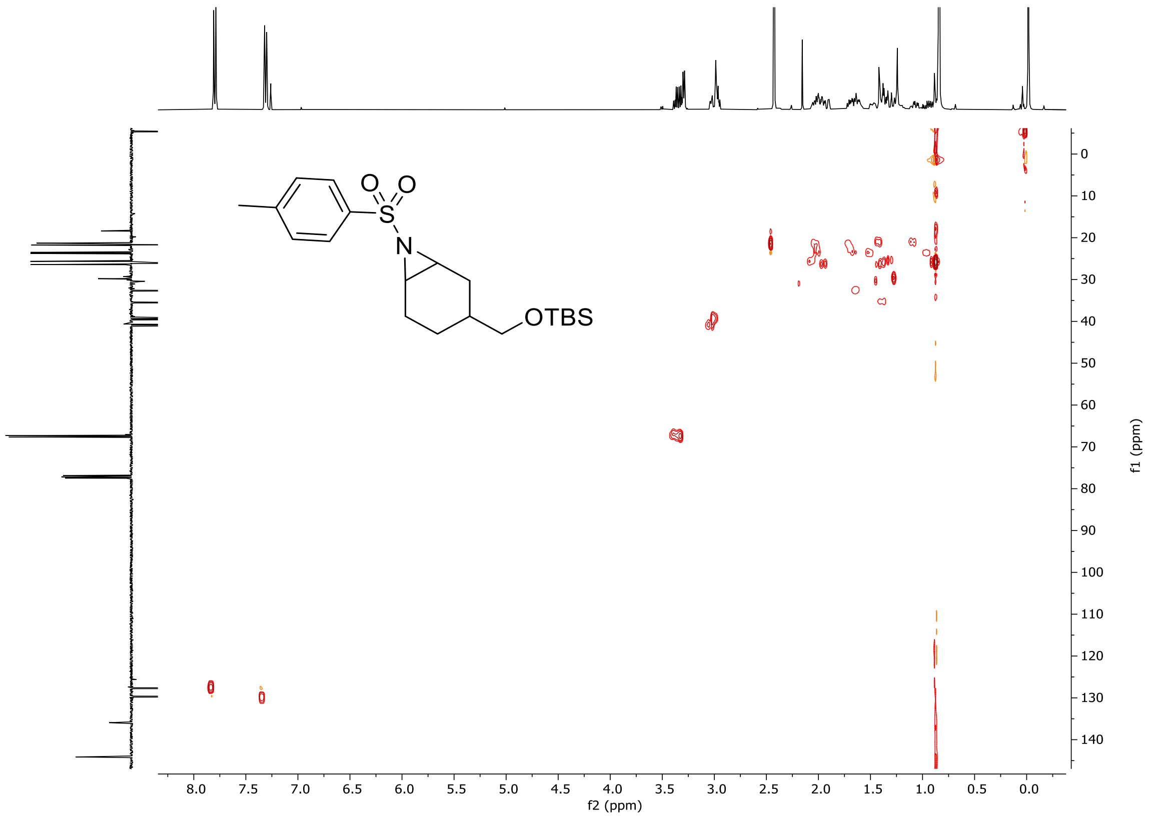

4s: ^{1}H NMR, 400 MHz in $CDCl_3$

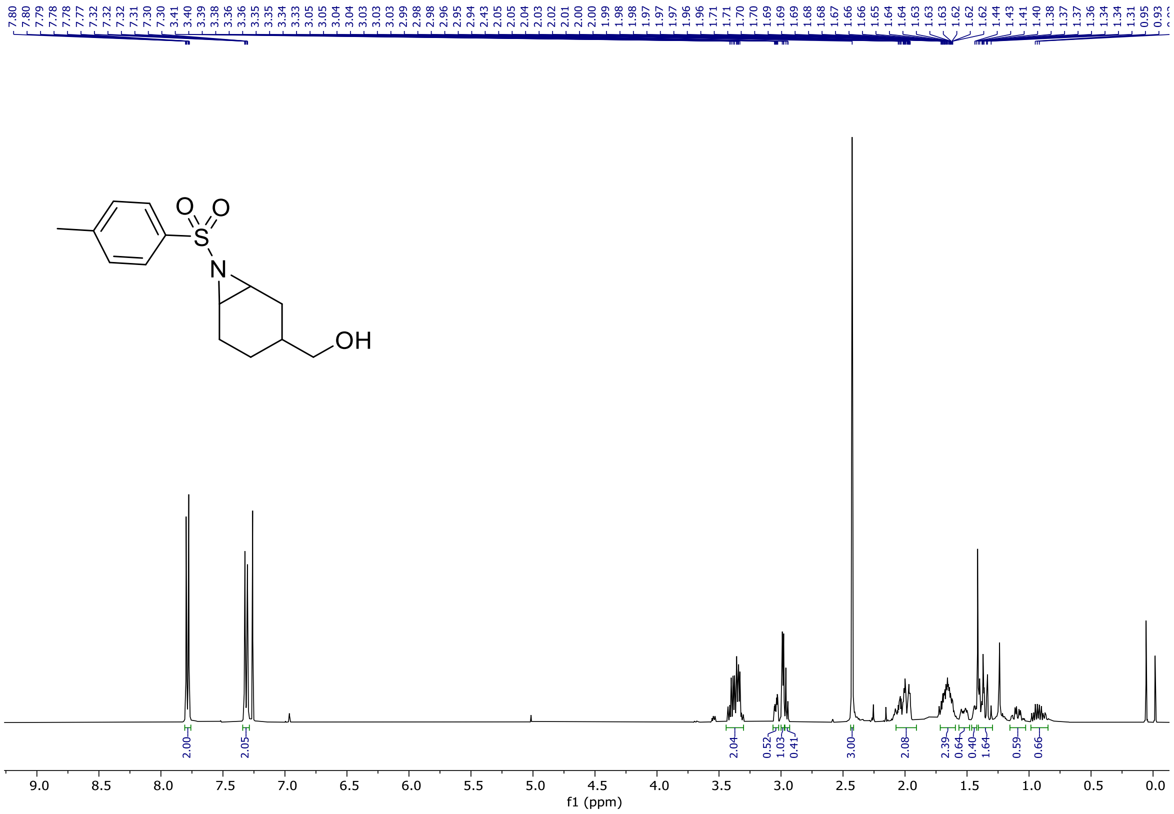

4s: $^{13}C\{^{1}H\}$-APT NMR, 101 MHz in $CDCl_3$

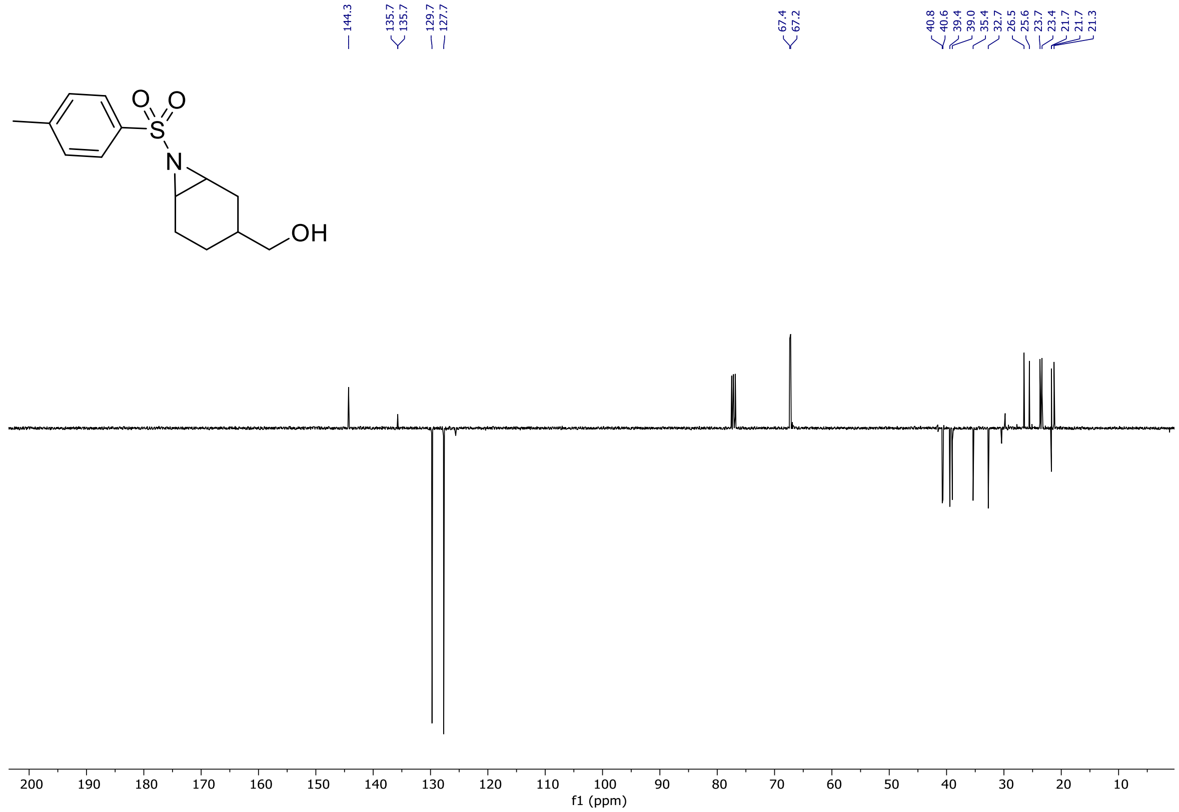

4s: ^{1}H-^{1}H COSY spectrum in $CDCl_3$

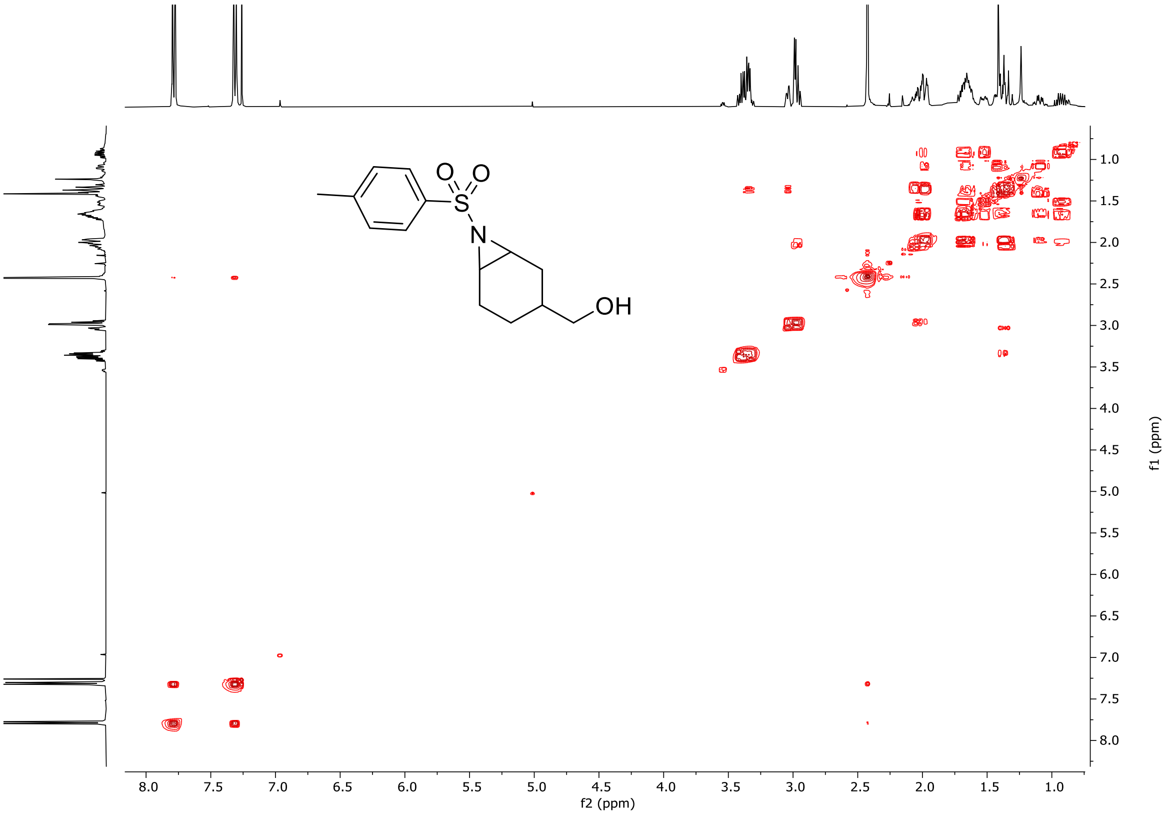

4s: ^{1}H-^{13}C HSQC spectrum in $CDCl_3$

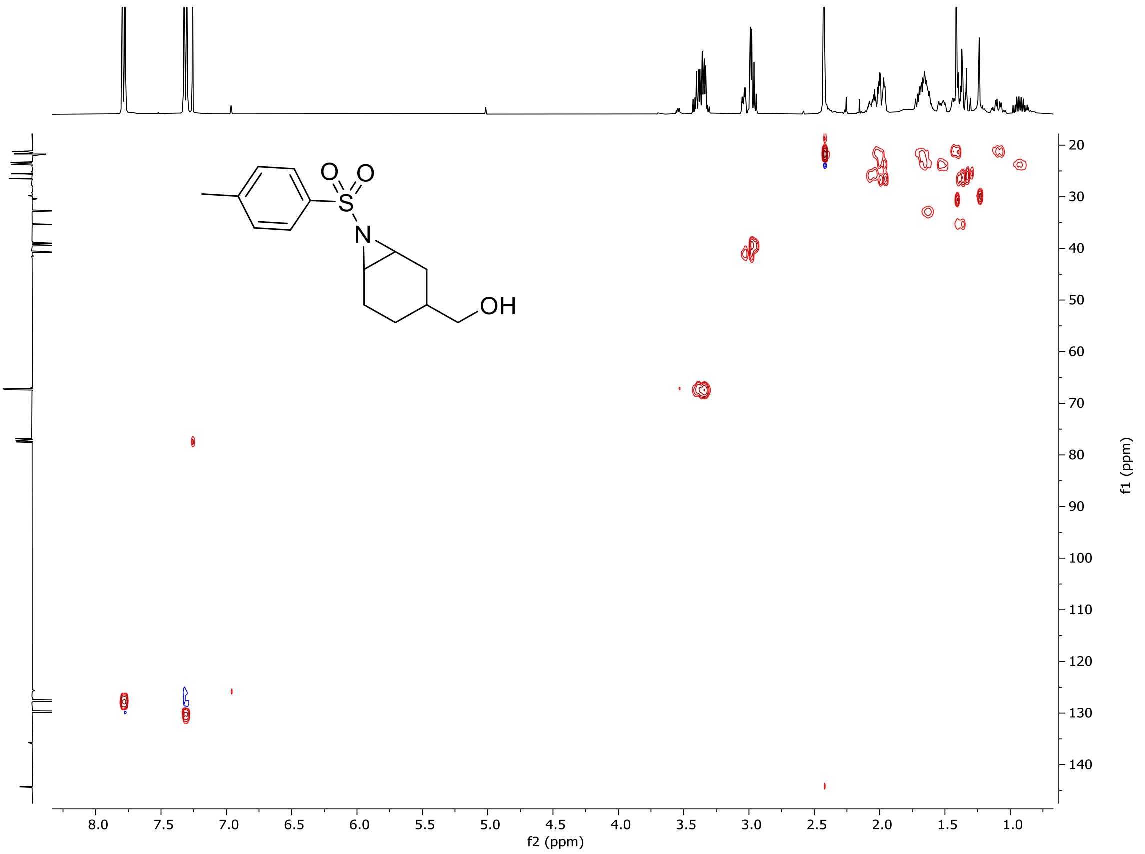

4t: ^{1}H NMR, 400 MHz in $CDCl_3$

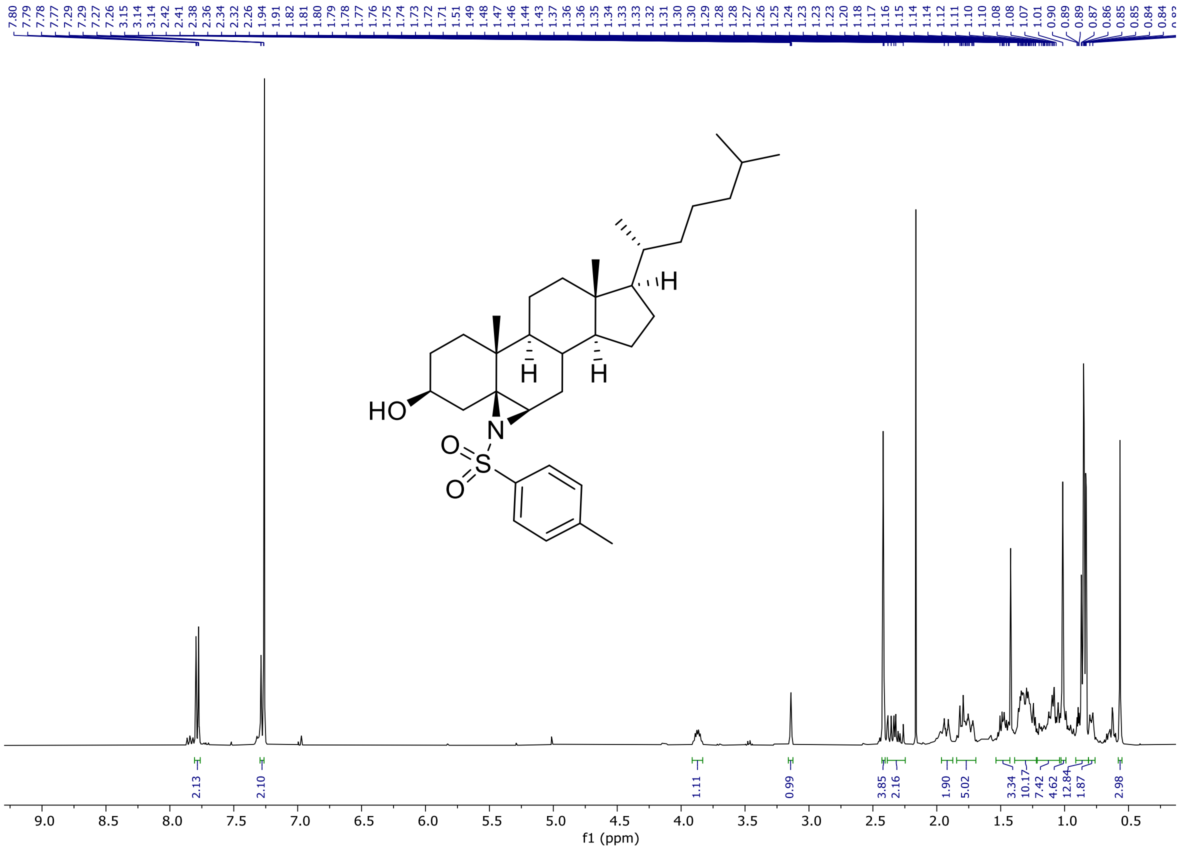

4t: $^{13}C\{^{1}H\}$-APT NMR, 101 MHz in $CDCl_3$

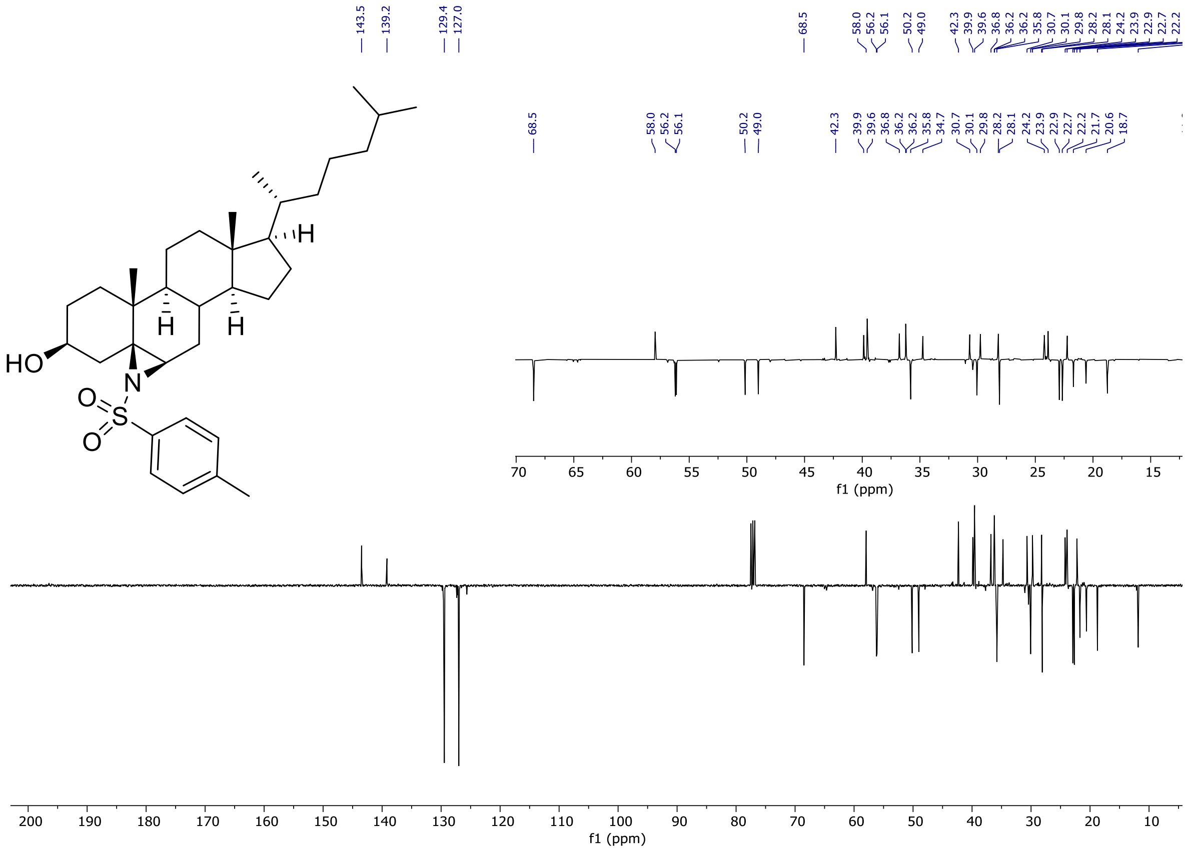

4t: ^{1}H-^{1}H COSY spectrum in $CDCl_3$

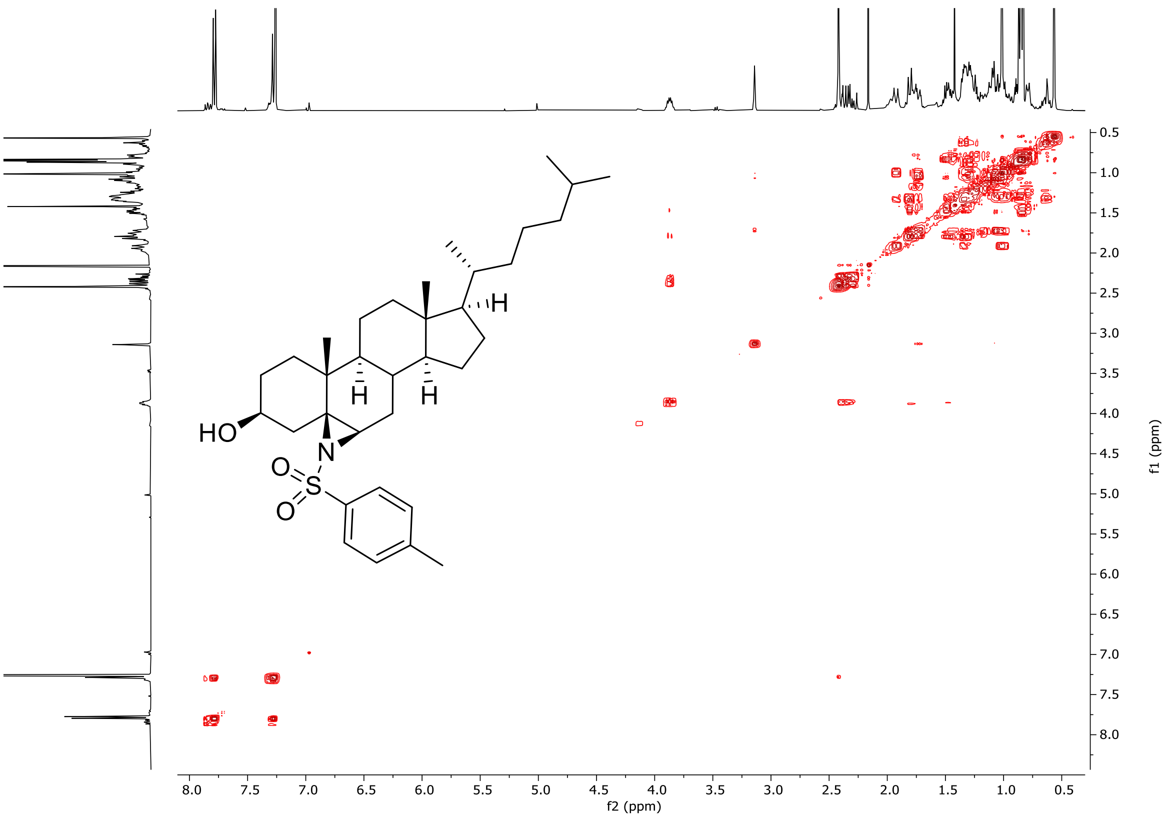

4t: ^{1}H-^{13}C HSQC spectrum in $CDCl_3$

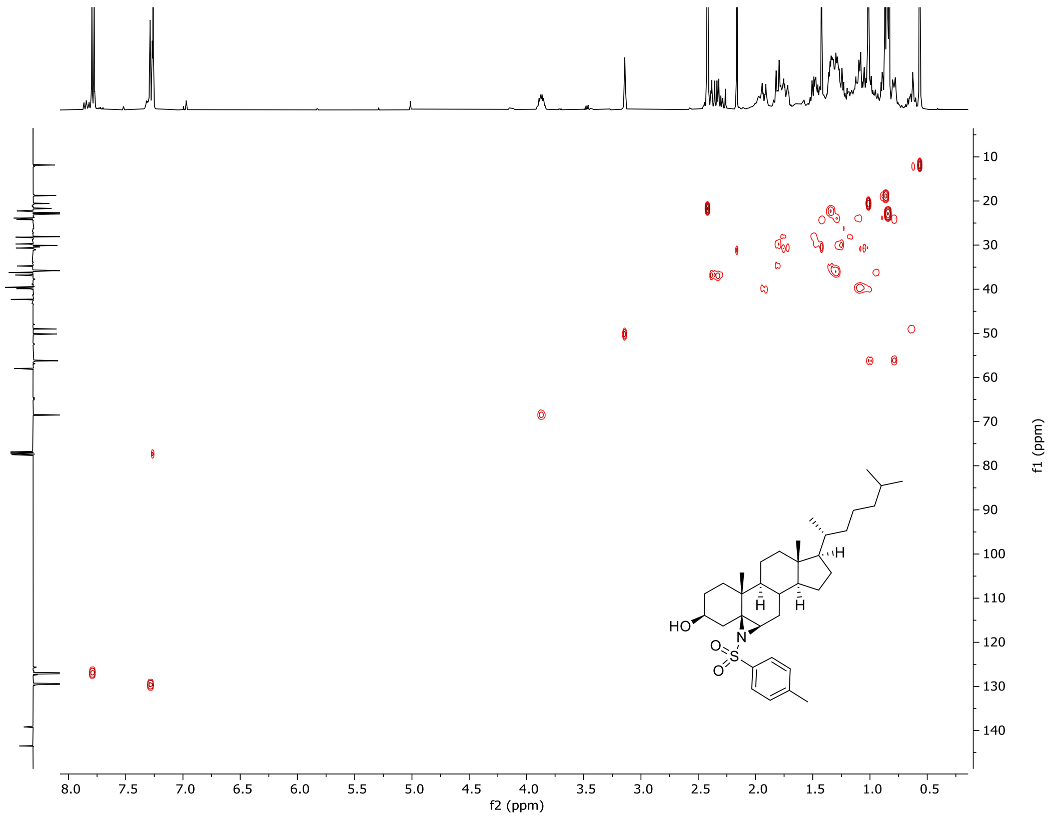

4t: ^{1}H-^{1}H NOESY spectrum in $CDCl_3$

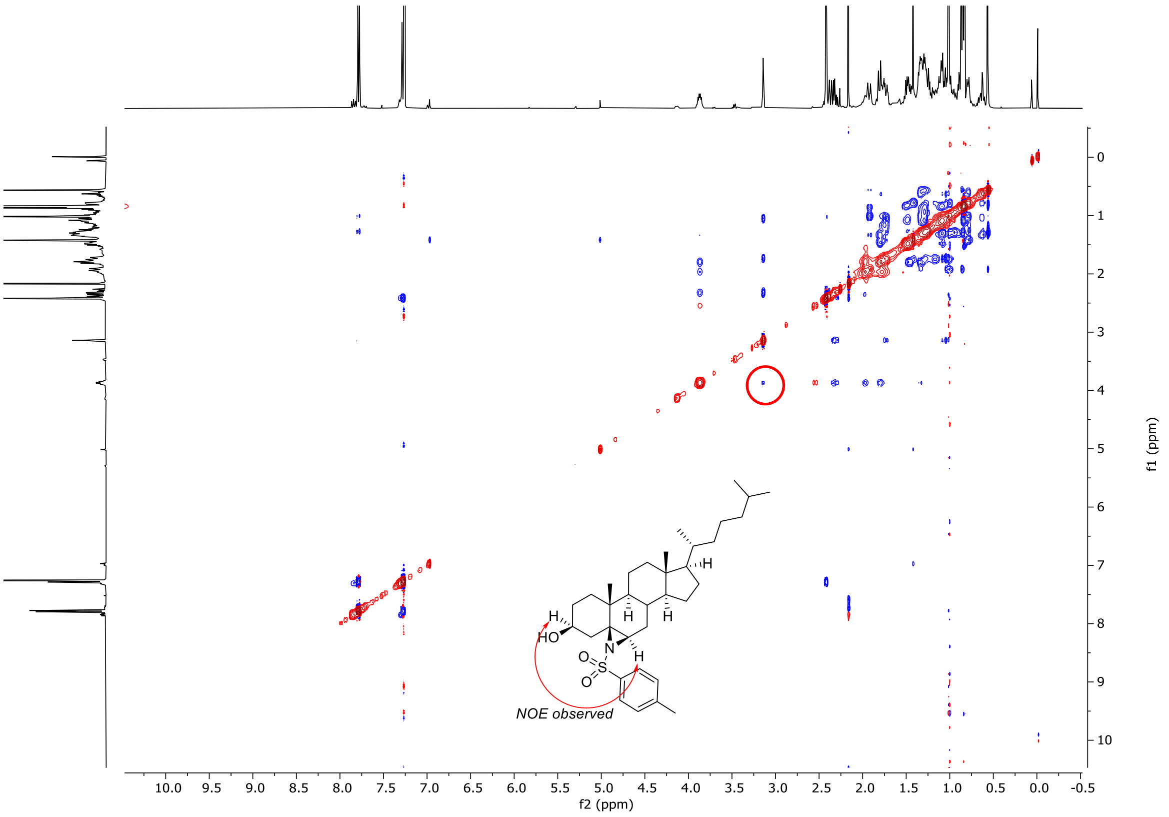

4u: ^{1}H NMR, 500 MHz in CDCl$_3$

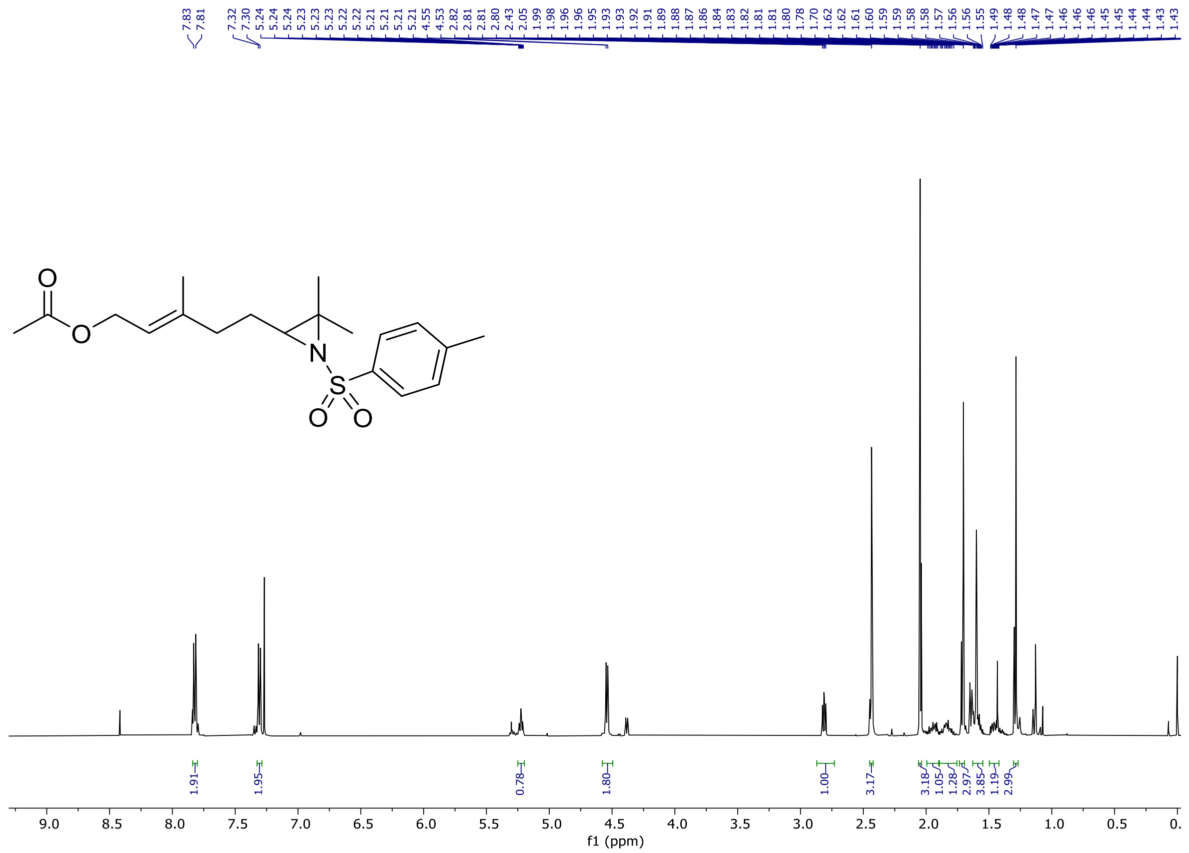

4u: ^{13}C{^{1}H}-APT NMR, 126 MHz in CDCl$_3$

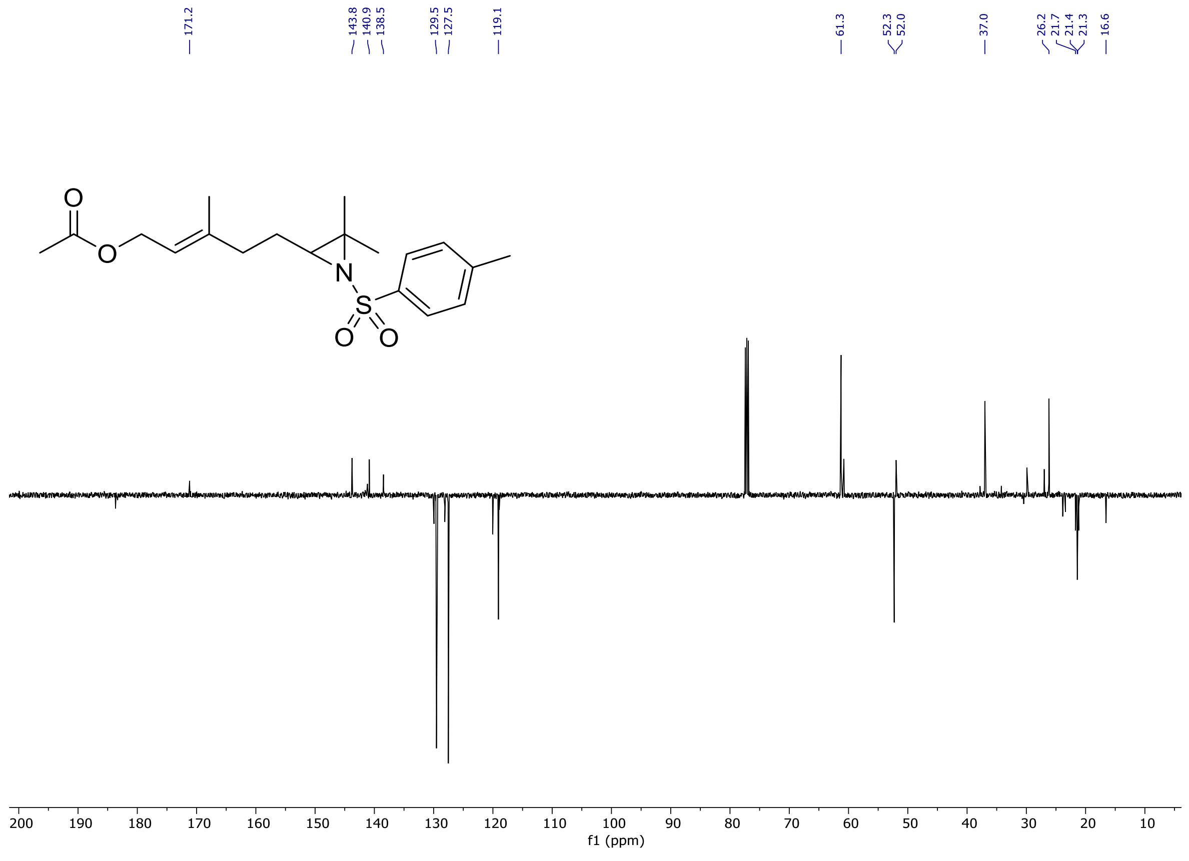

4u: ^{1}H-^{1}H COSY spectrum in $CDCl_3$

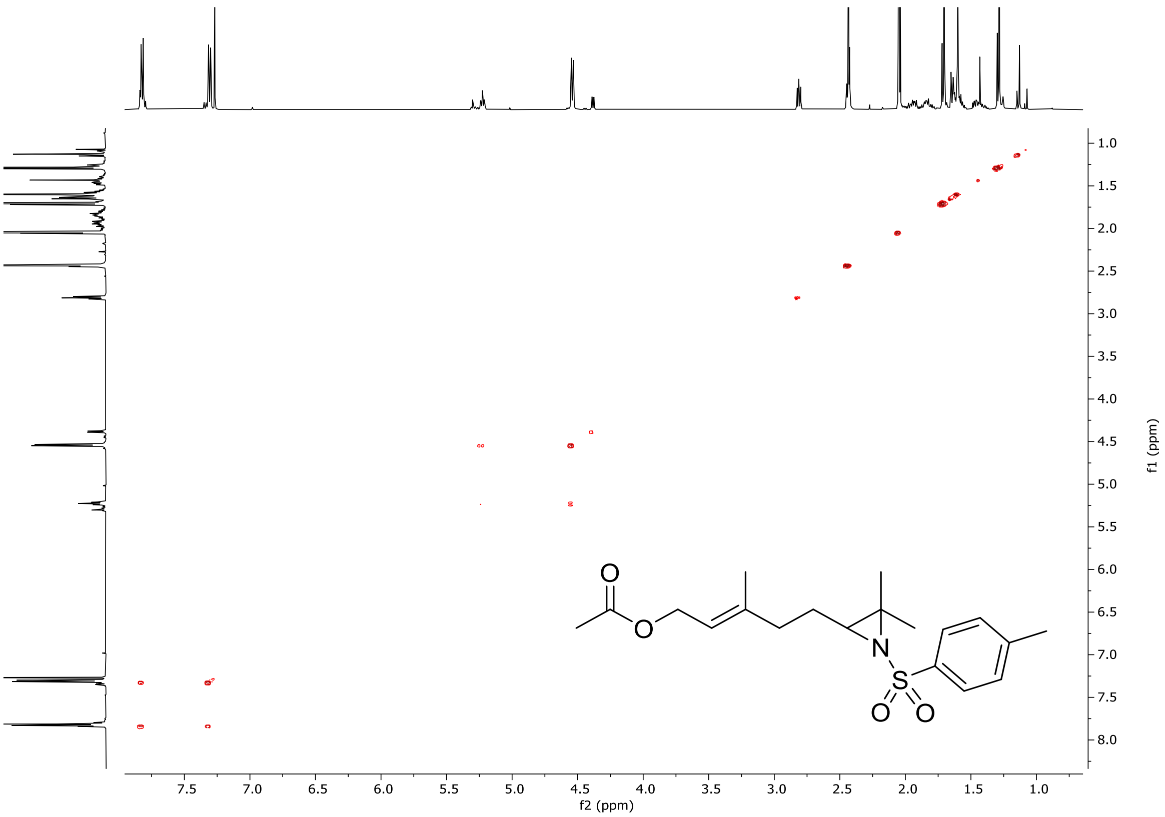

4u: ^{1}H-^{13}C HSQC spectrum in $CDCl_3$

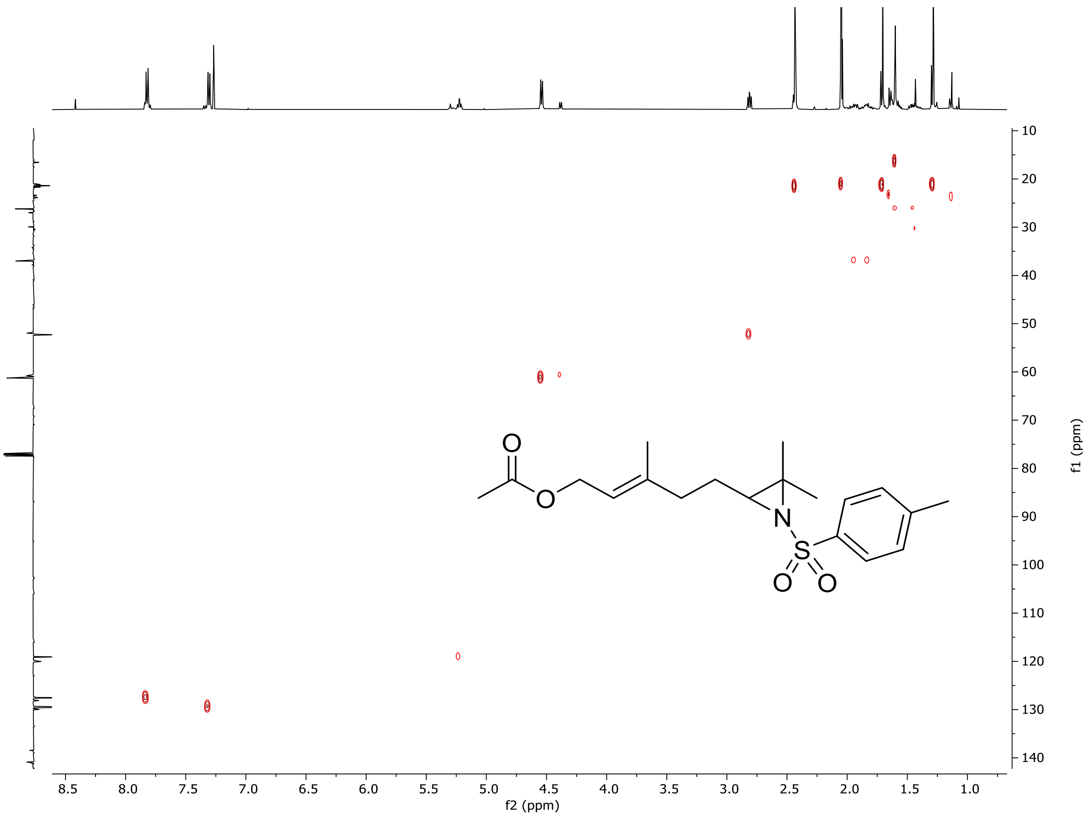

4v: ^{1}H NMR, 400 MHz in $CDCl_3$

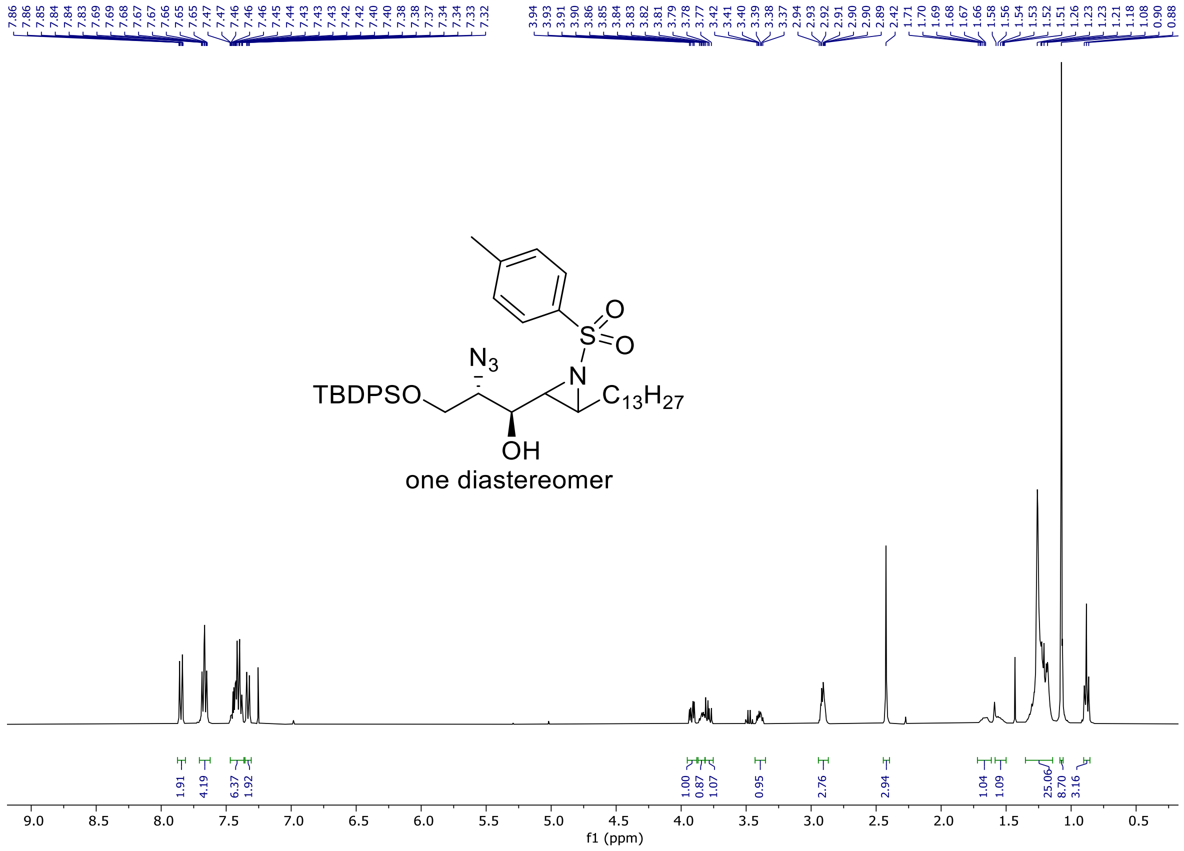

4v: ^{13}C{^{1}H}-APT NMR, 101 MHz in $CDCl_3$

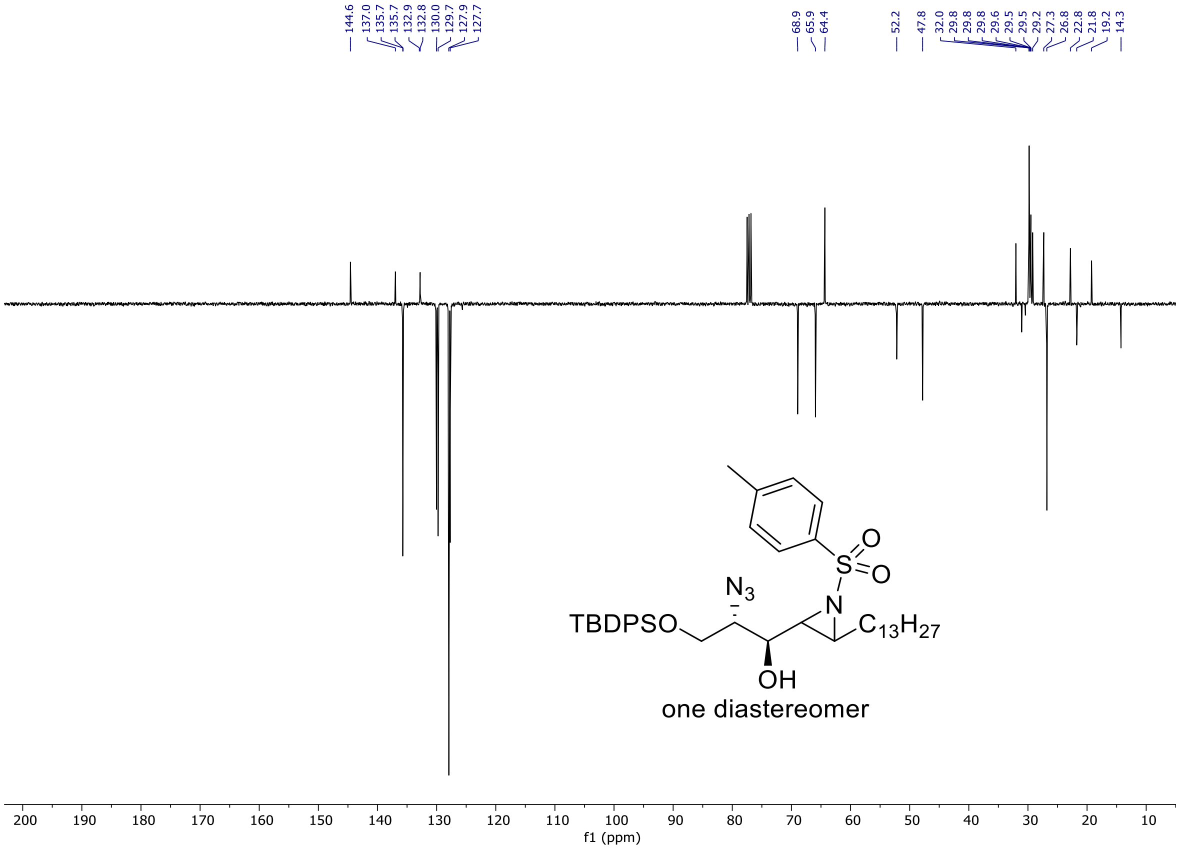

4v: ^{1}H-^{1}H COSY spectrum in $CDCl_3$

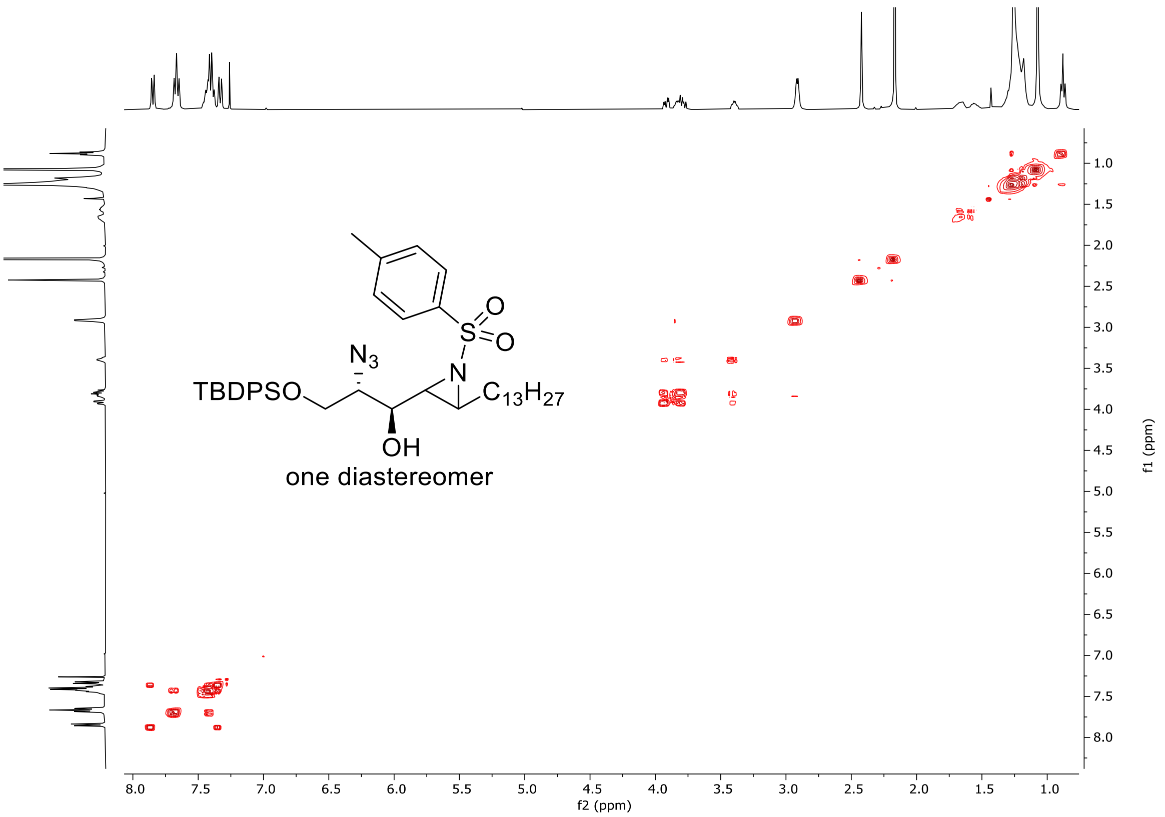

4v: ^{1}H-^{13}C HSQC spectrum in $CDCl_3$

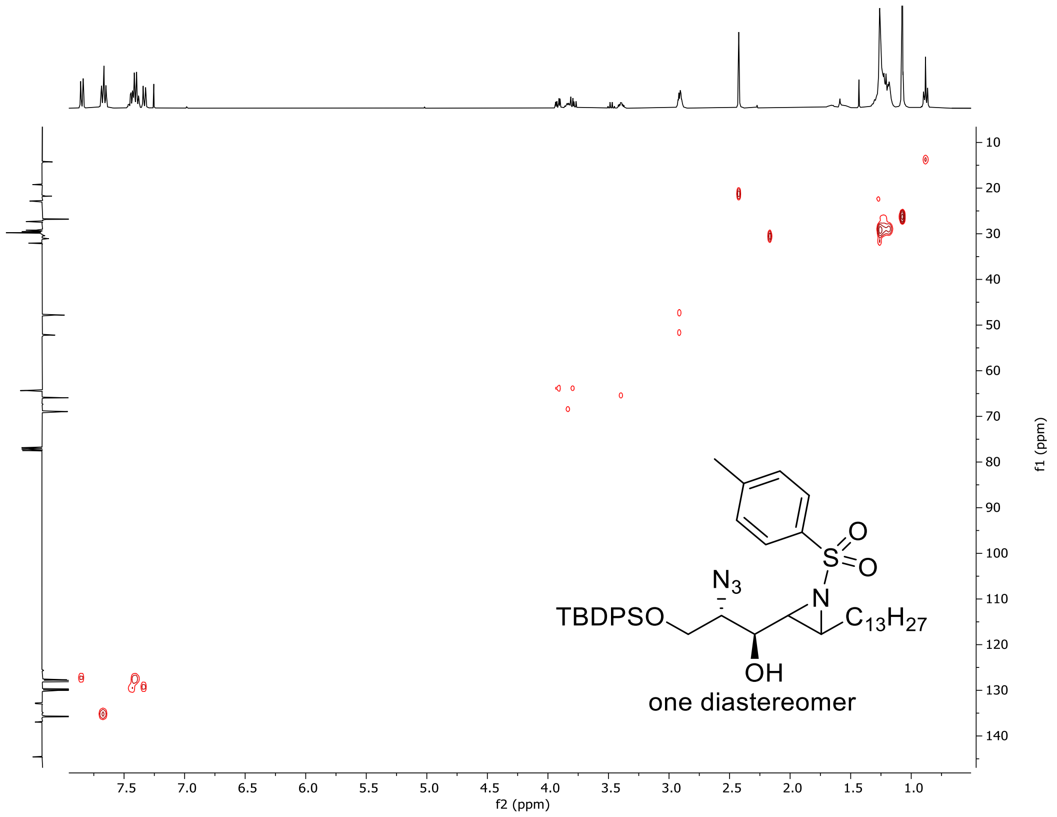

4v: ^{1}H NMR, 400 MHz in $CDCl_3$

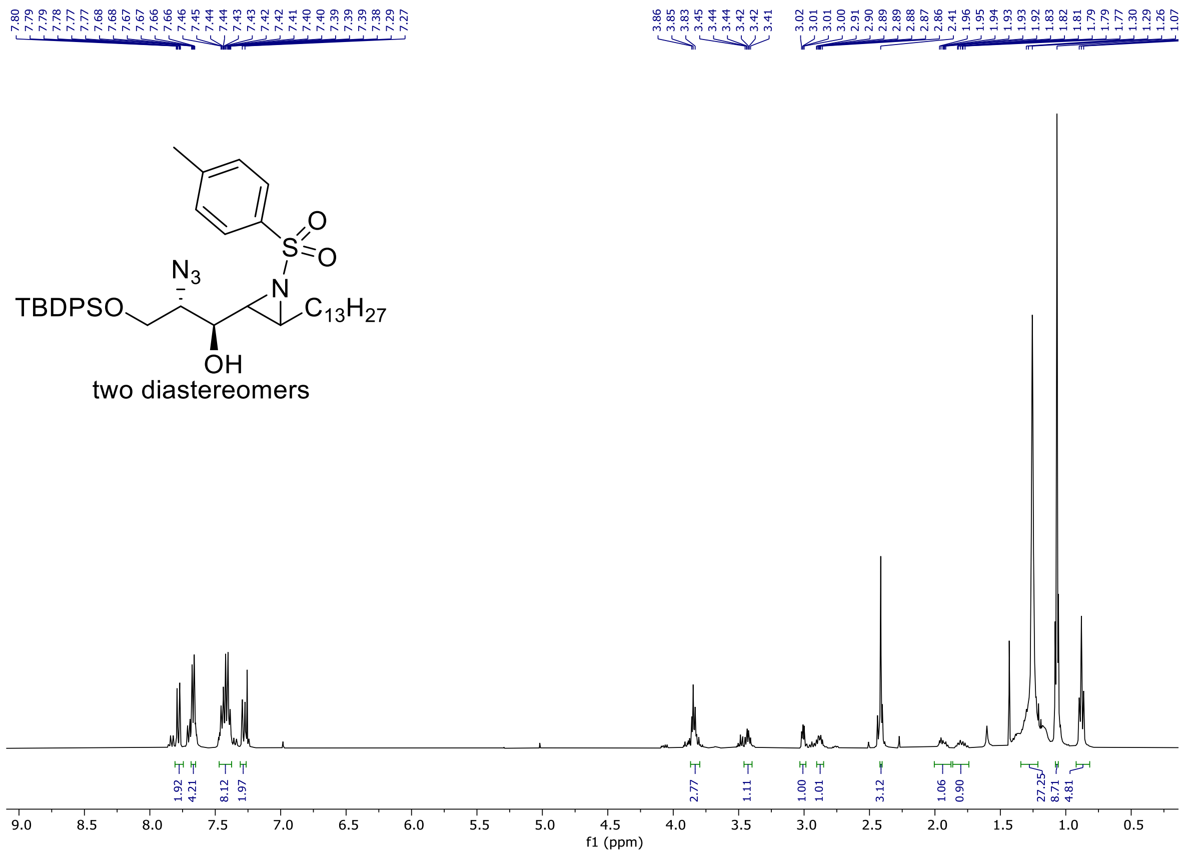

4v: ^{13}C{^{1}H}-APT NMR, 101 MHz in $CDCl_3$

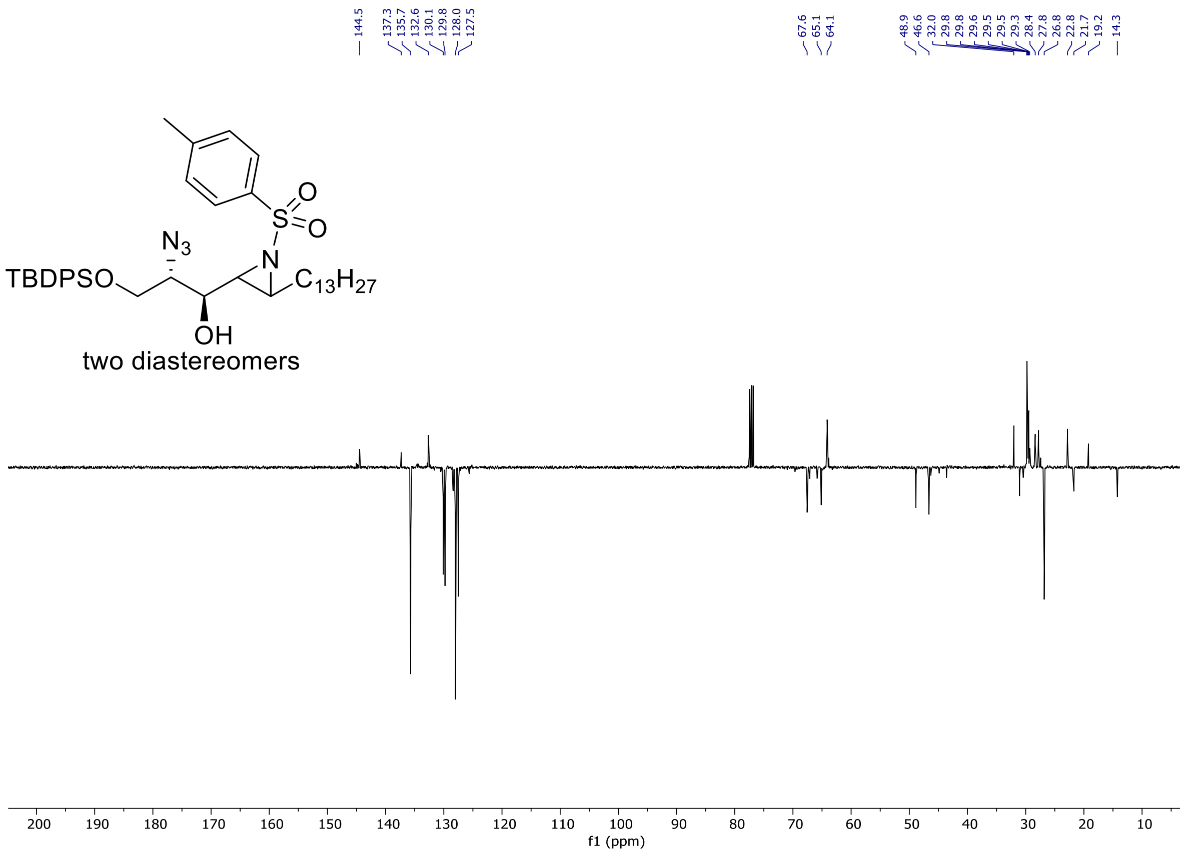

4v: ^{1}H-^{1}H COSY spectrum in $CDCl_3$

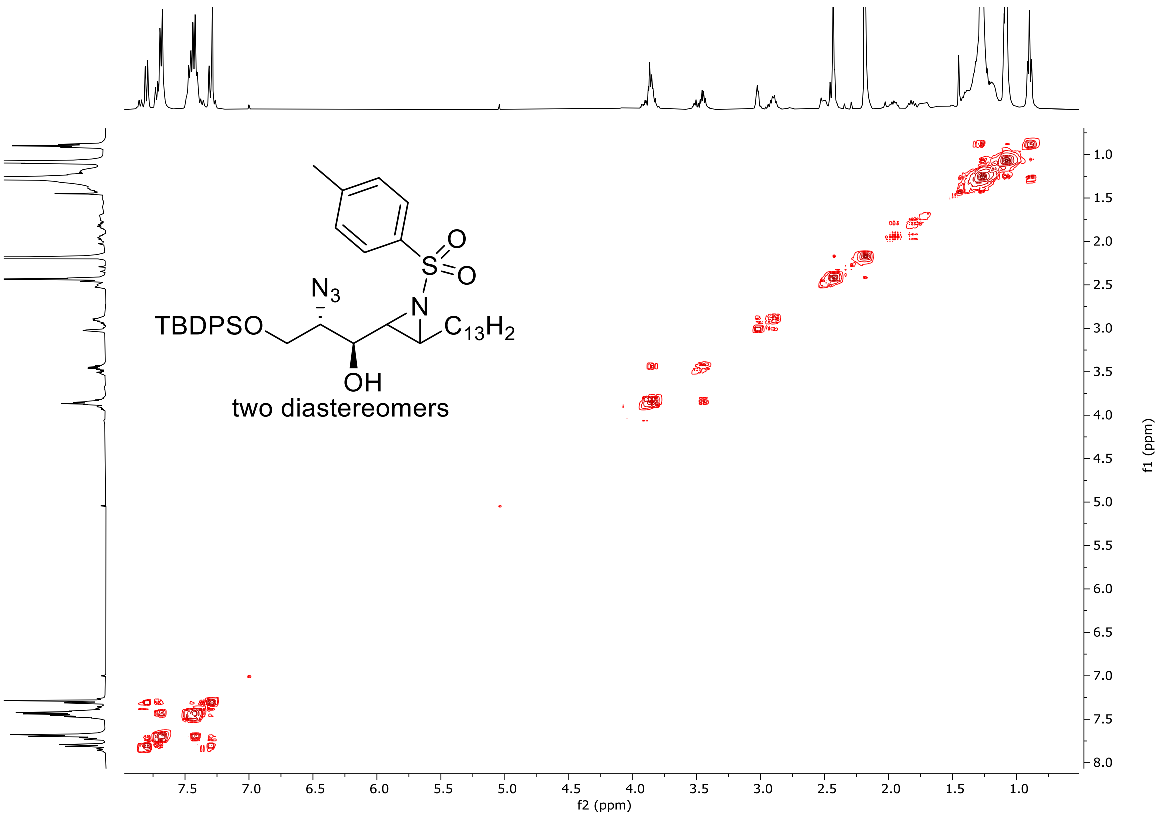

4v: ^{1}H-^{13}C HSQC spectrum in $CDCl_3$

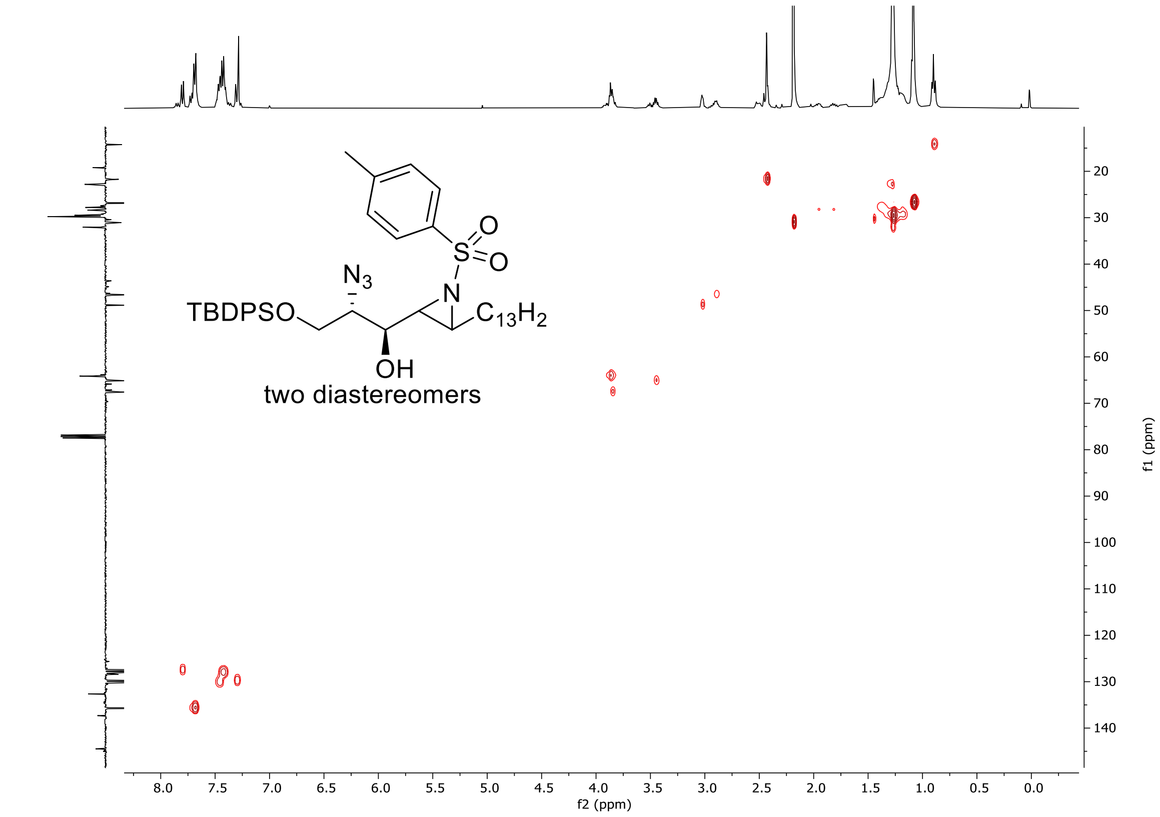

4x: ^{1}H NMR, 400 MHz in $CDCl_3$

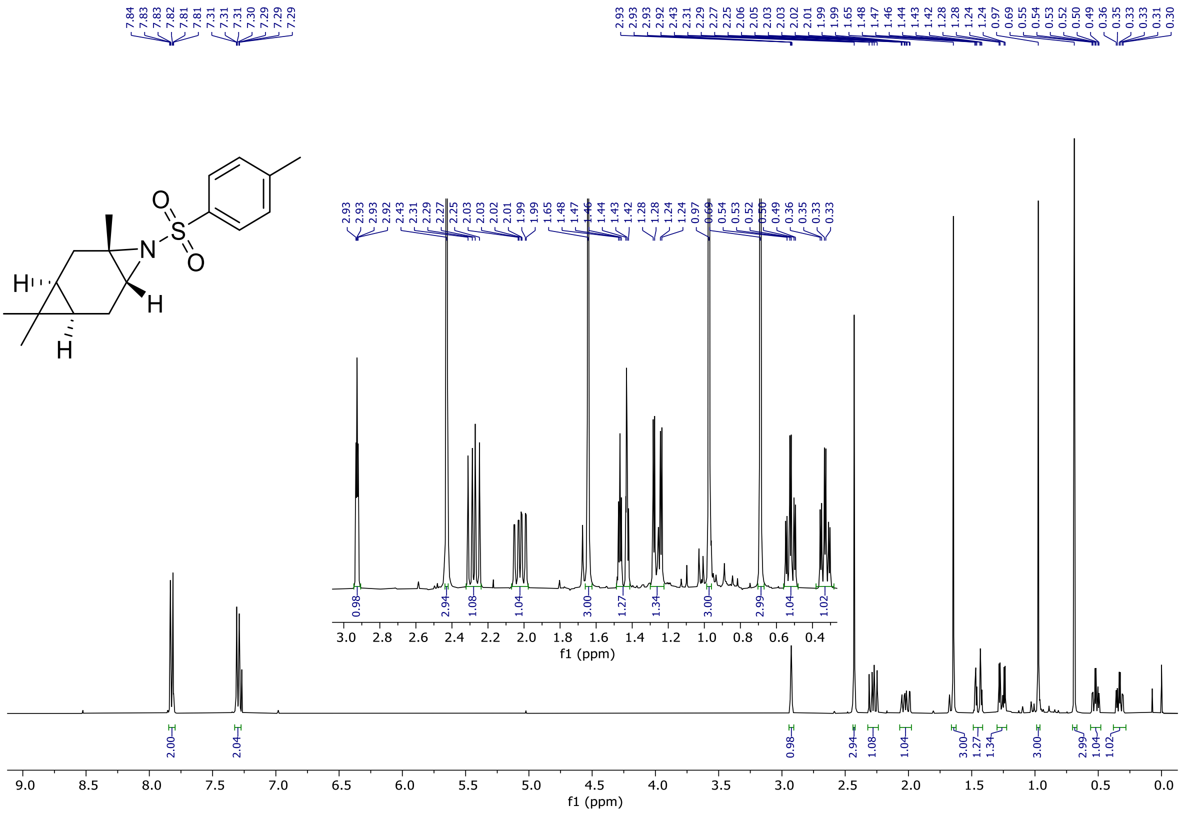

4x: ^{13}C{^{1}H}-APT NMR, 101 MHz in $CDCl_3$

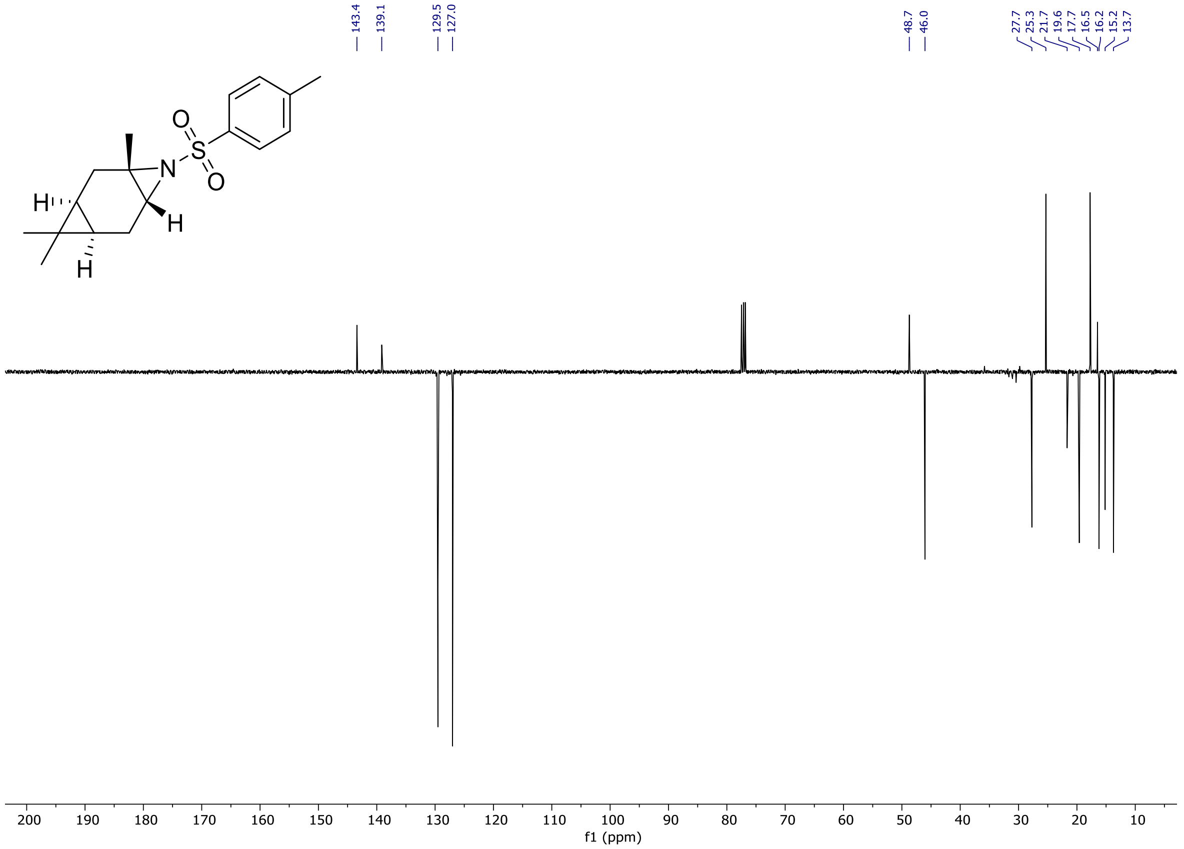

4x: ^{1}H-^{1}H COSY spectrum in $CDCl_3$

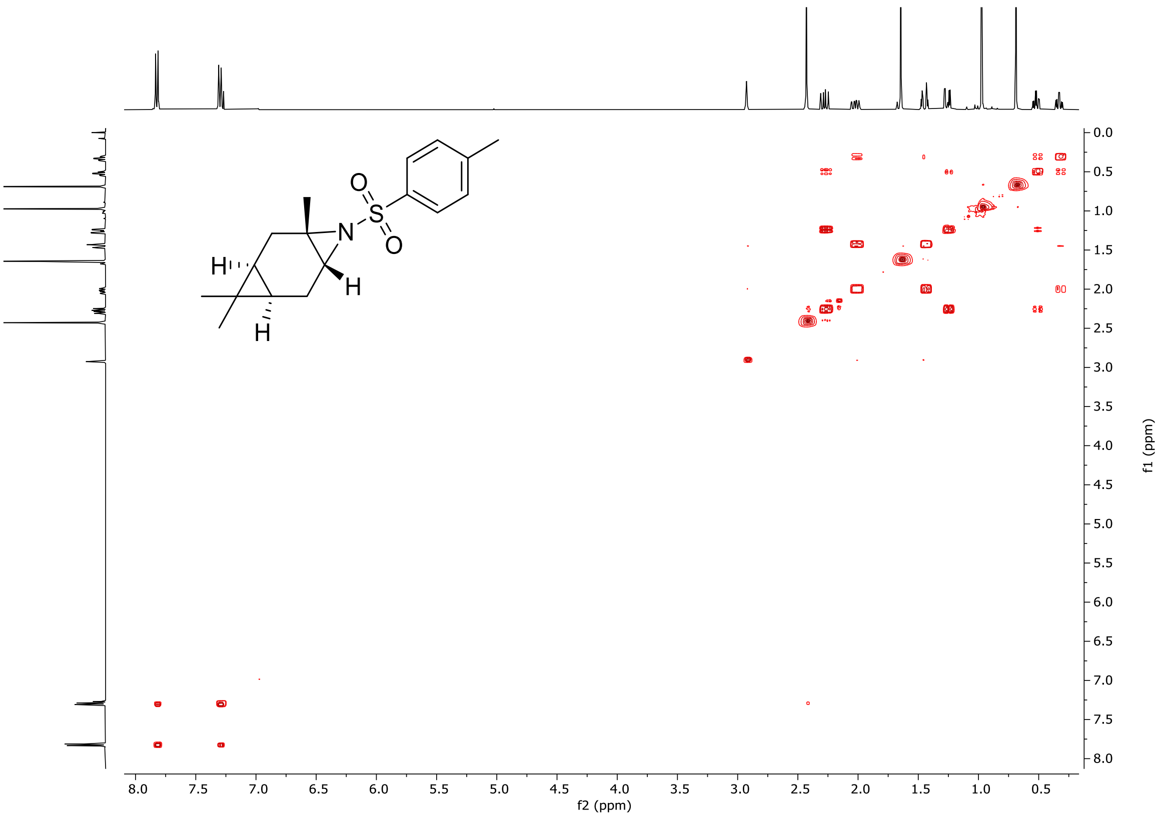

4x: ^{1}H-^{13}C HSQC spectrum in $CDCl_3$

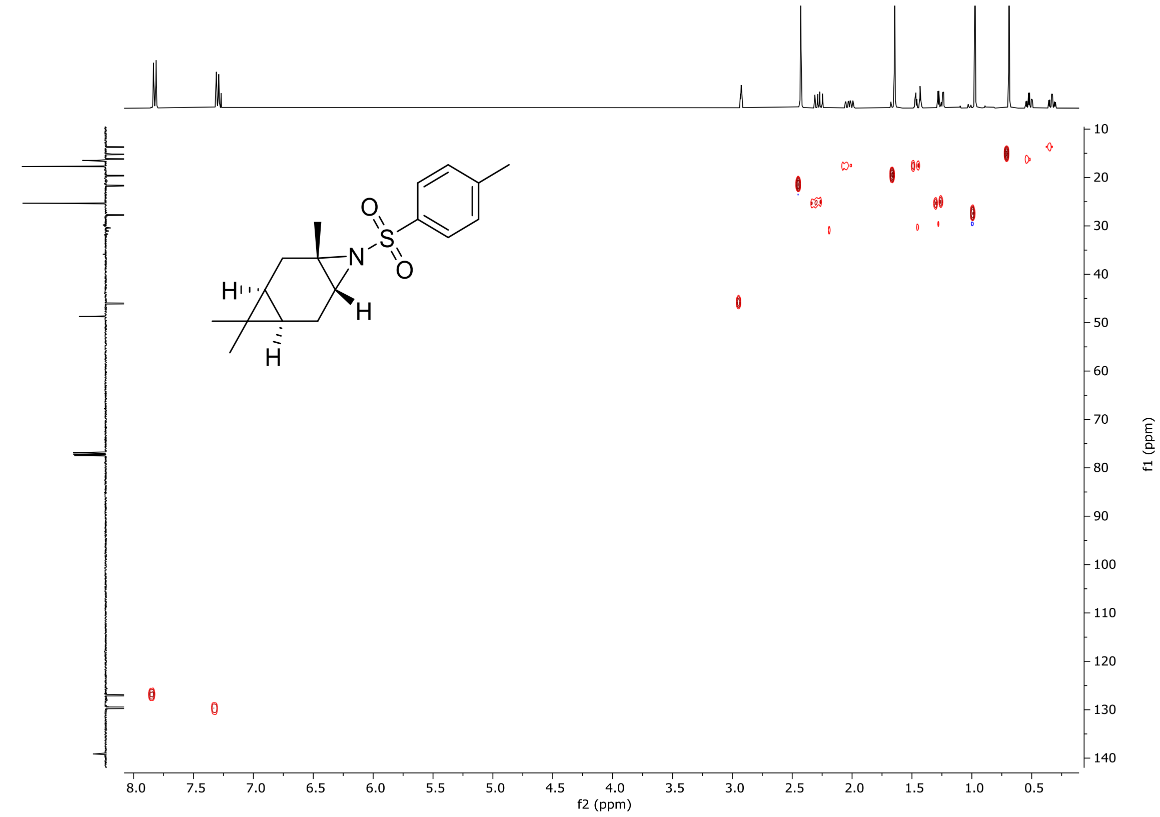

4y: ^{1}H NMR, 500 MHz in $CDCl_3$

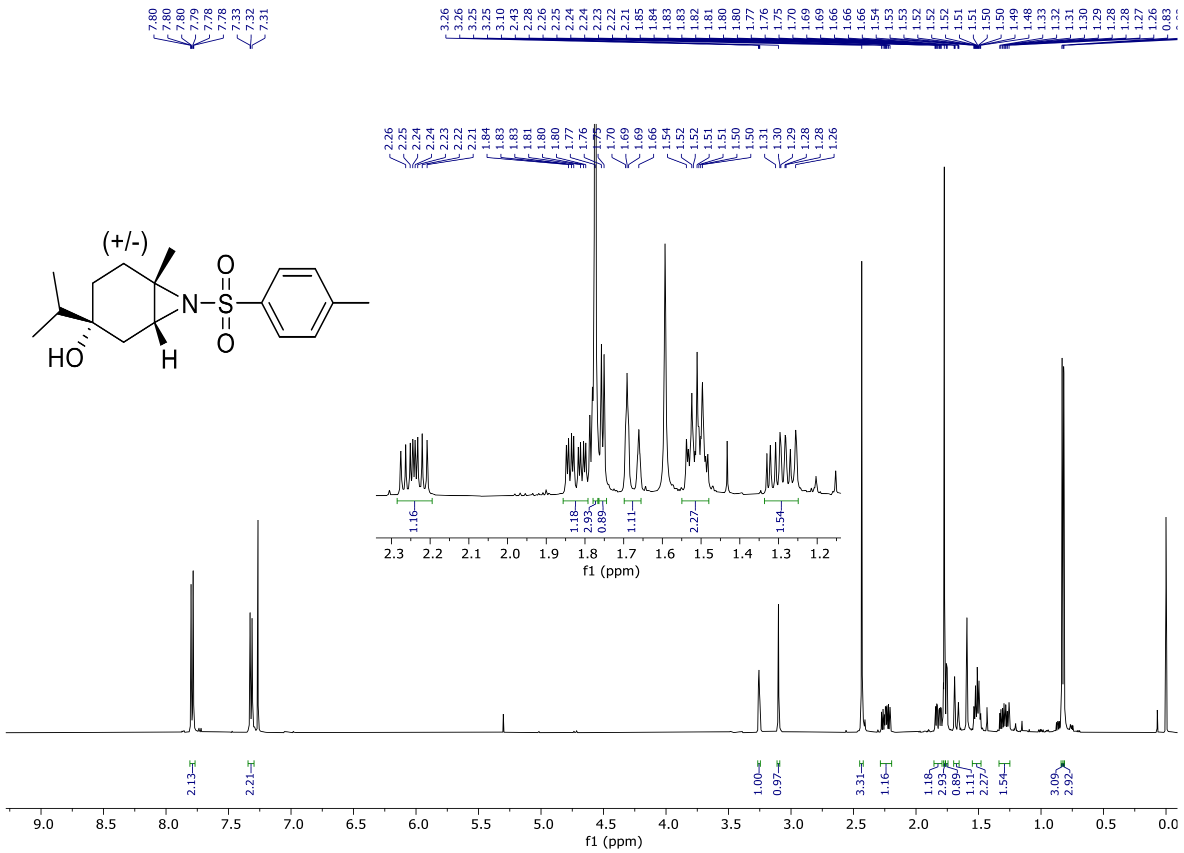

4y: $^{13}C\{^{1}H\}$-APT NMR, 126 MHz in $CDCl_3$

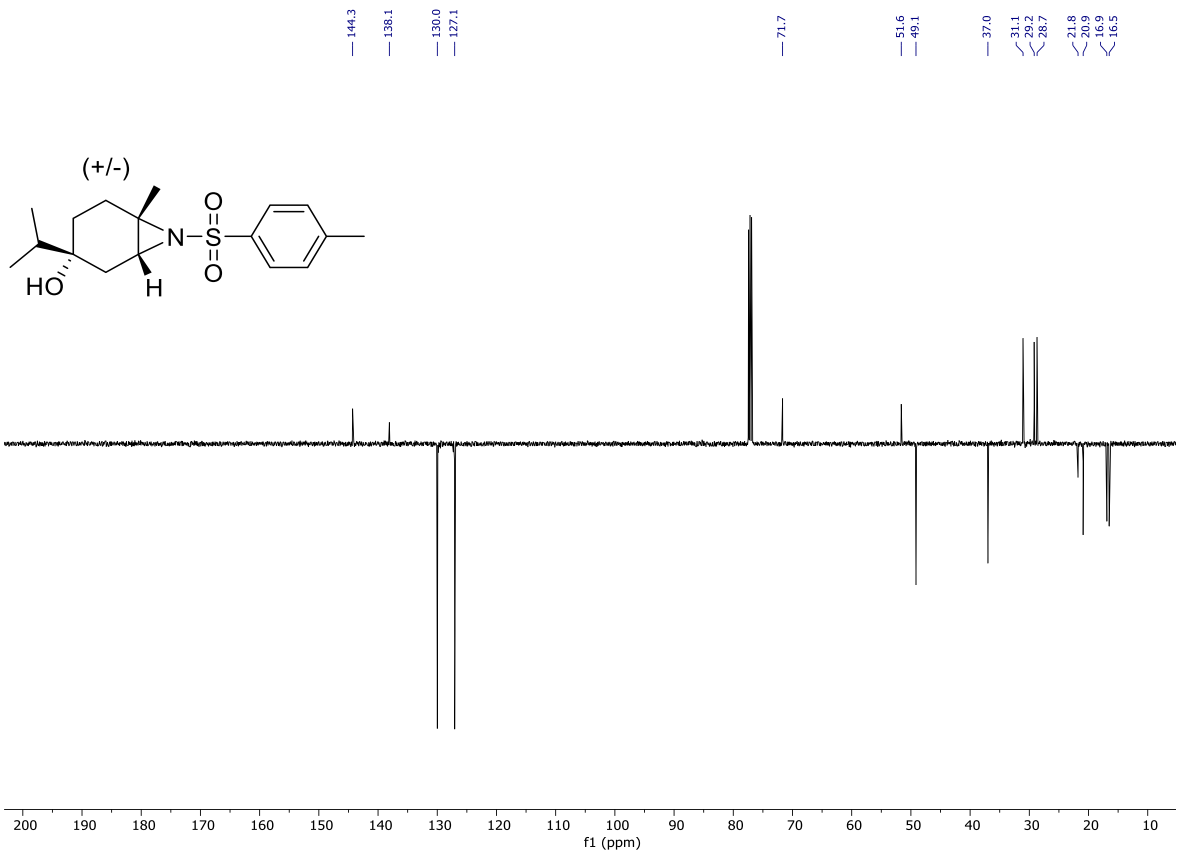

4y: ^{1}H-^{1}H COSY spectrum in $CDCl_3$

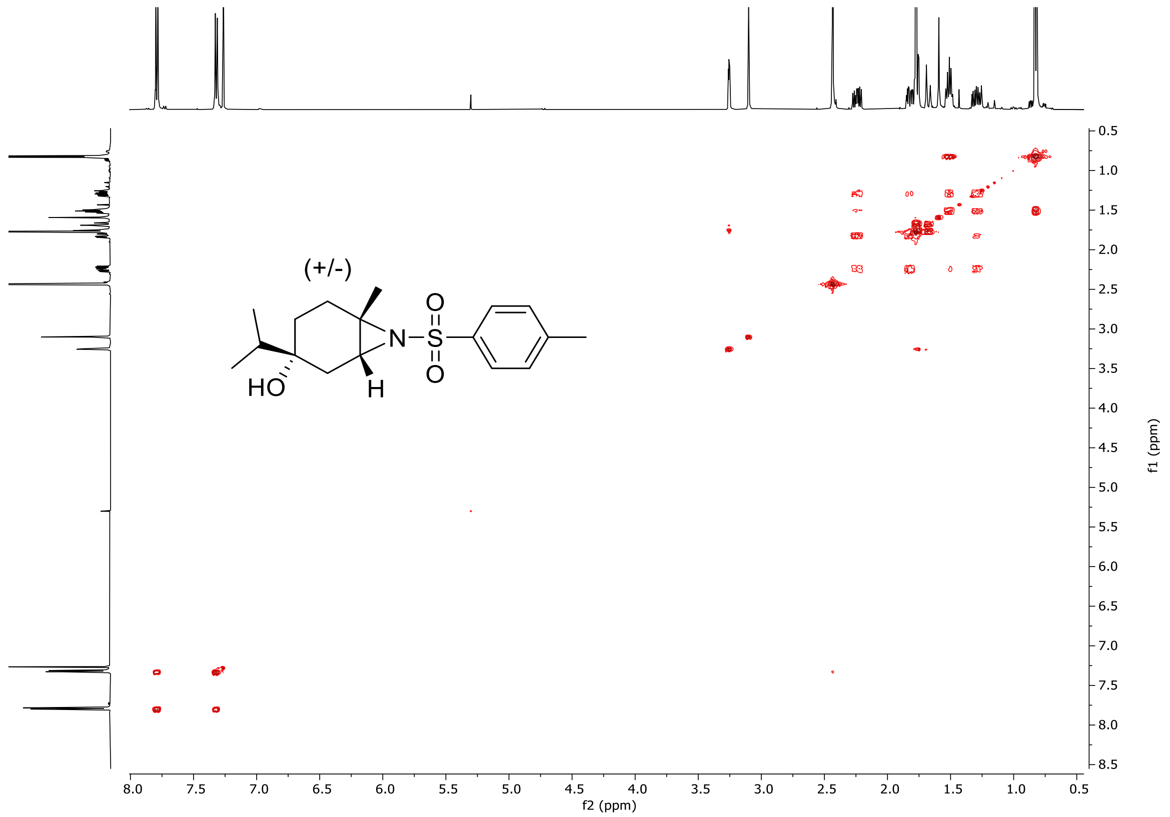

4y: ^{1}H-^{13}C HSQC spectrum in $CDCl_3$

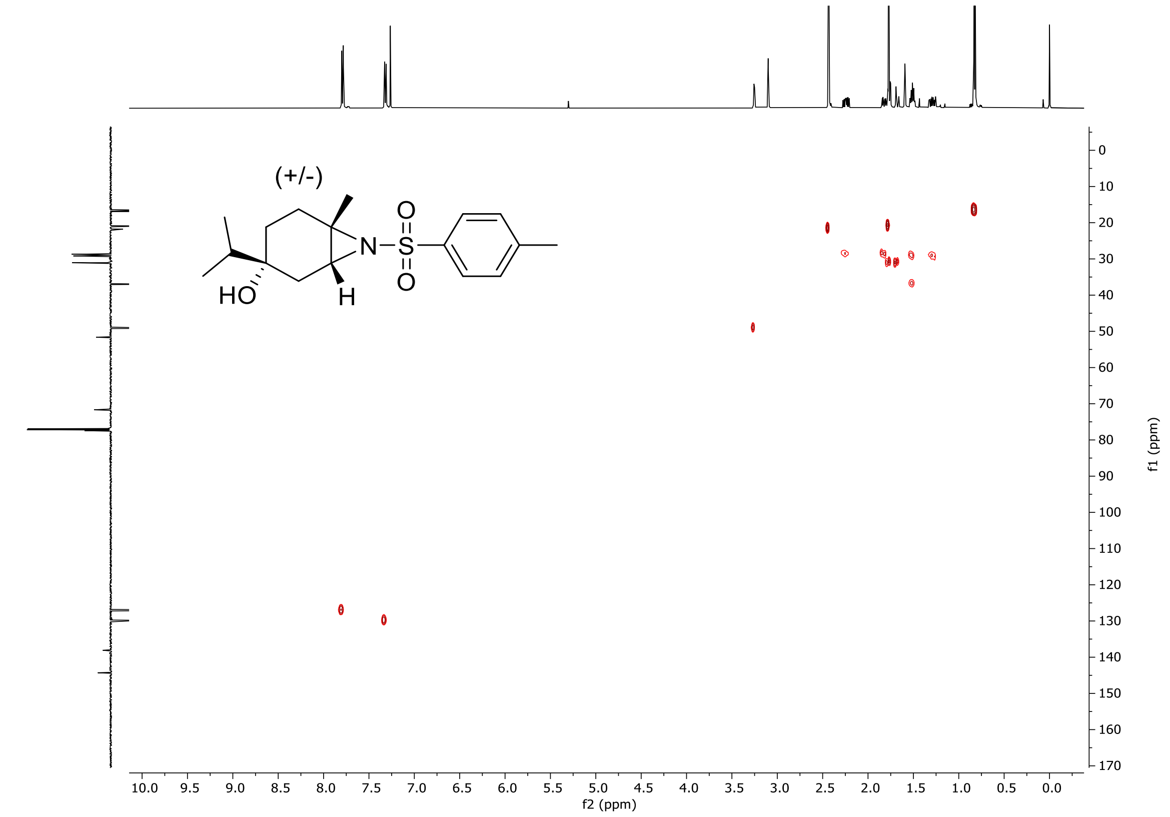

4y: ^{1}H-^{1}H NOESY spectrum in $CDCl_3$

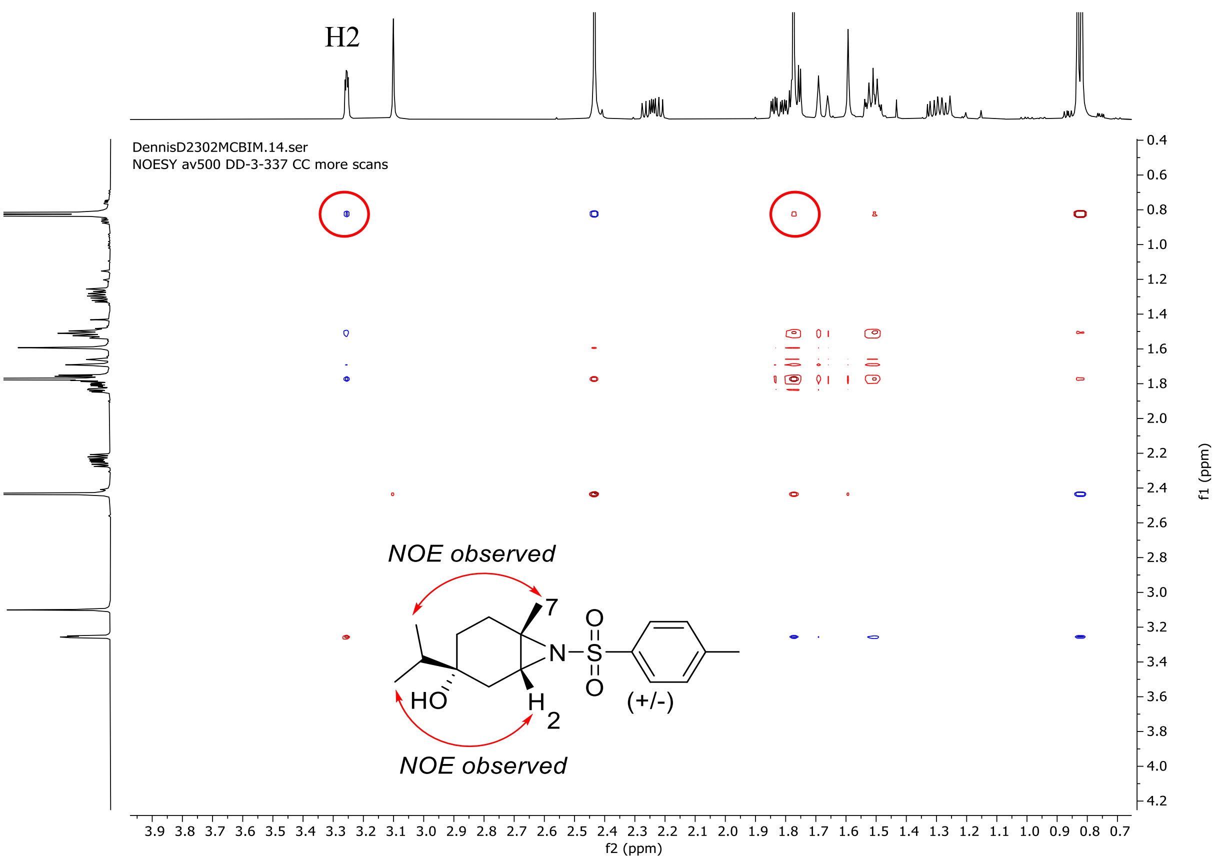

4z: ^{1}H NMR, 400 MHz in $CDCl_3$

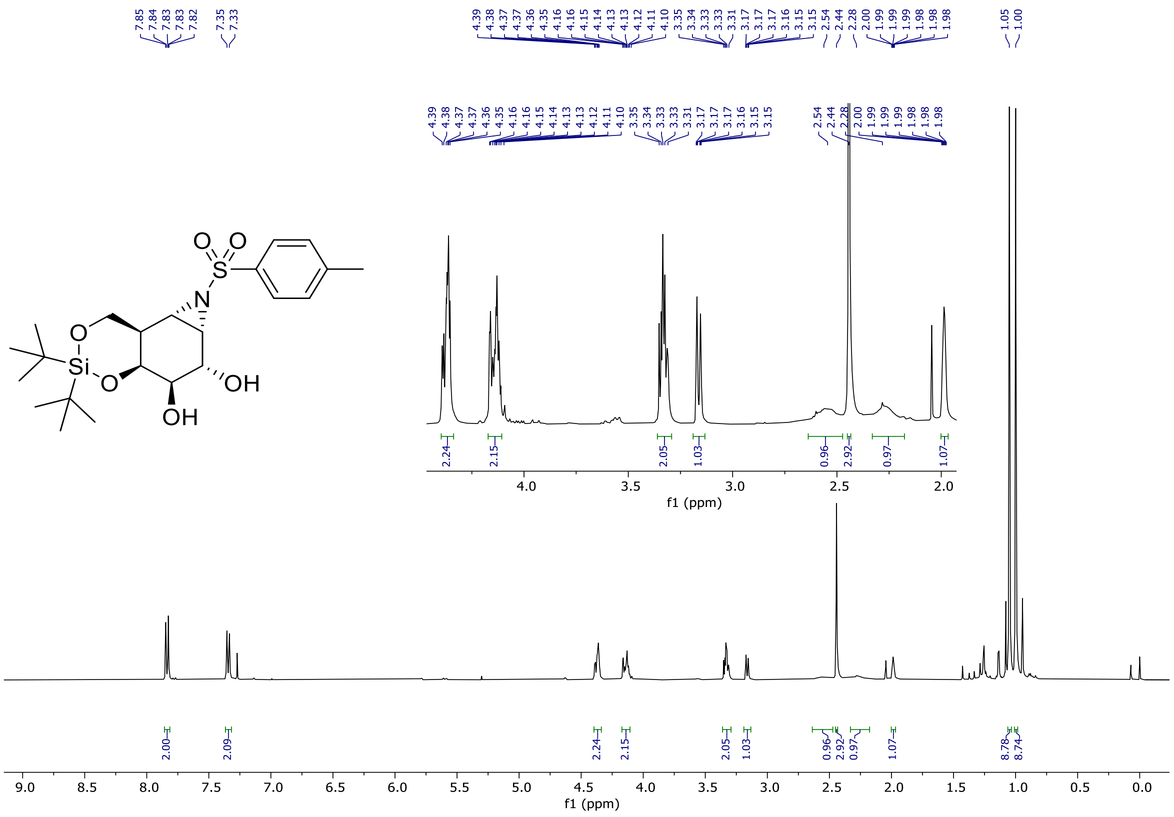

4z: $^{13}C\{^{1}H\}$-APT NMR, 101 MHz in $CDCl_3$

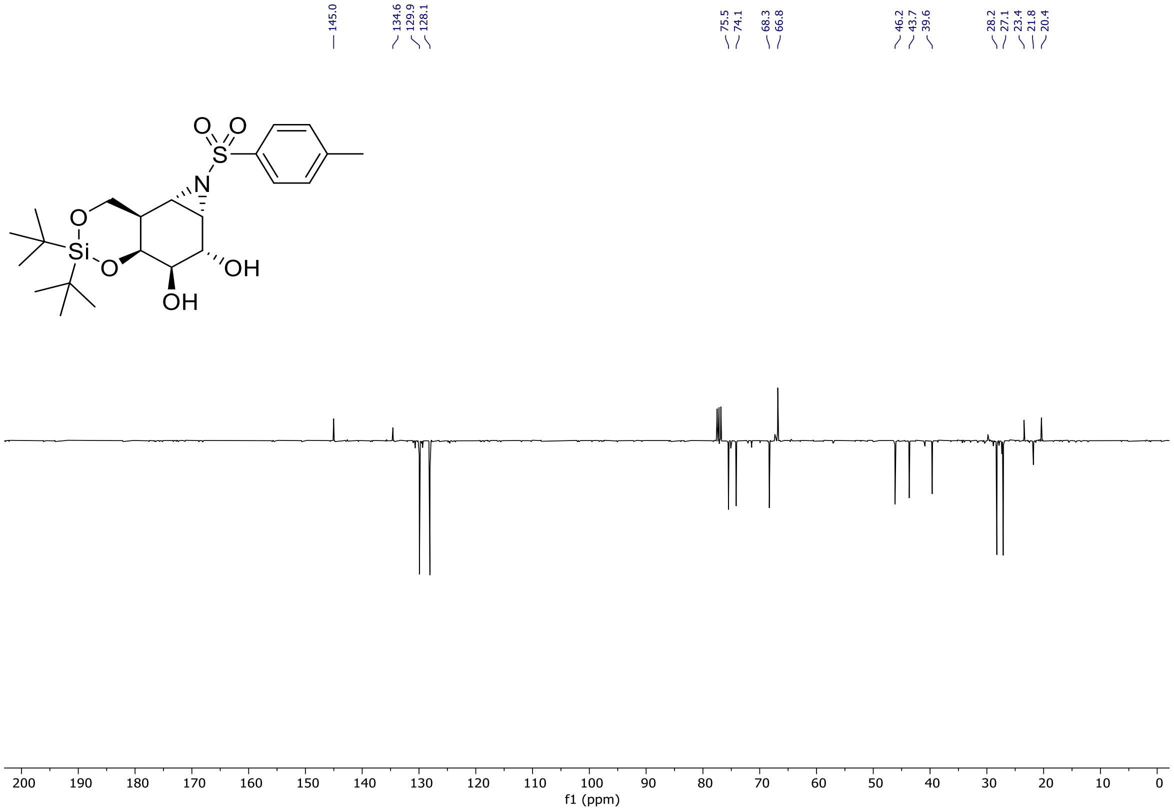

4z: ^{1}H-^{1}H COSY spectrum in $CDCl_3$

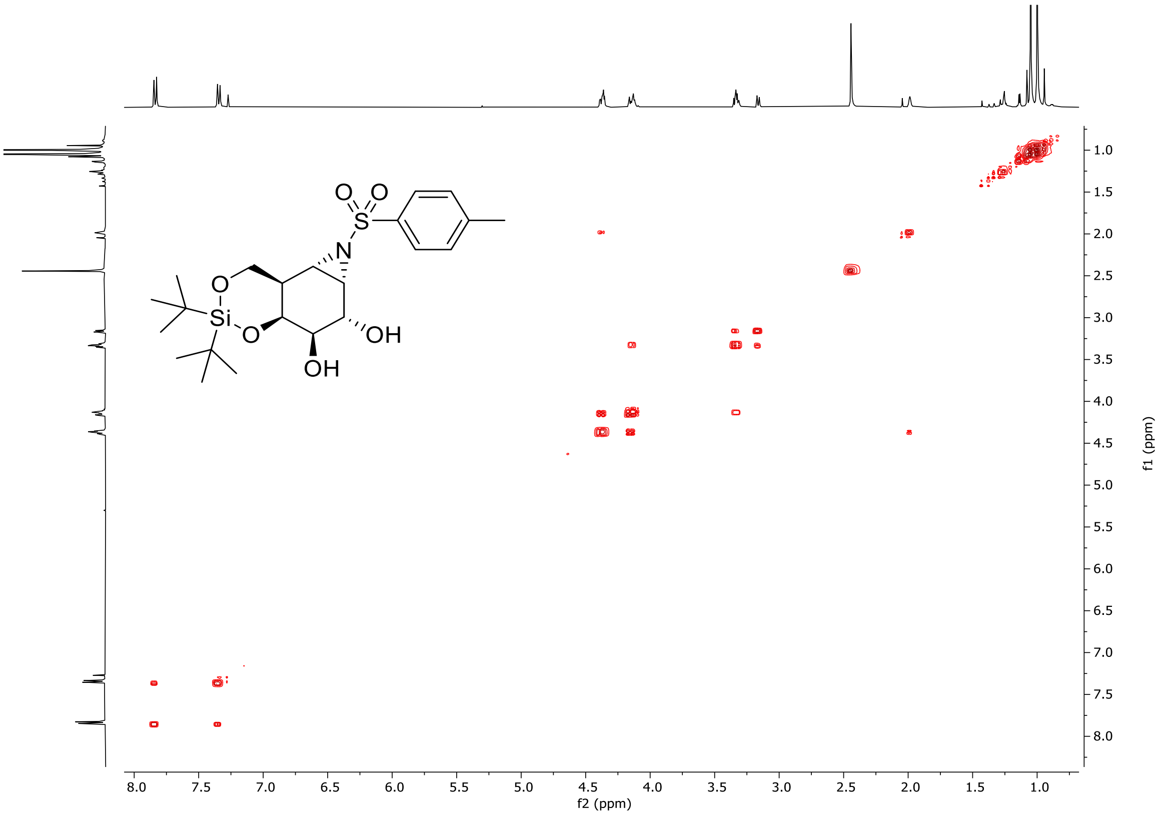

4z: ^{1}H-^{13}C HSQC spectrum in $CDCl_3$

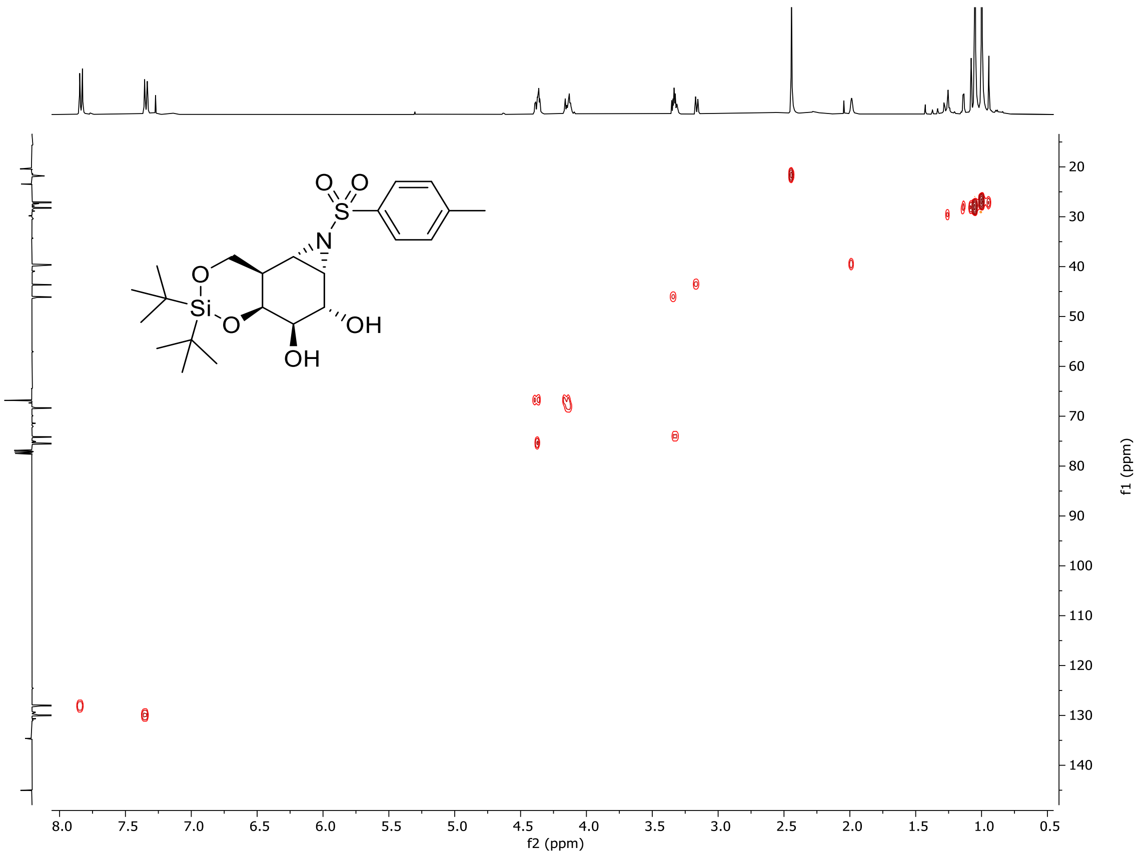

Supplement: Supplementary file 1 — jo3c02709_si_001.pdf [file jo3c02709_si_001.pdf]
